# Inflammatory alterations mediate tau-associated neurodegeneration

**DOI:** 10.1093/braincomms/fcag242

**Published:** 2026-06-24

**Authors:** Patrick J Lao, Seonjoo Lee, Daniel Talmasov, Dina Dass, Ndubisi Chikwem, Aubrey Johnson, Anna Smith, Diana Guzman, Amarachukwu Okafor, Hannah Houlihan, Lauren Heuer, Thairi Sanchez, Andrea Maldonado, Catherine Palacios, Samantha Rossano, Howard Andrews, Christiane Reitz, William C Kreisl, James M Noble, Yasir H Qureshi, Scott A Small

**Affiliations:** Taub Institute for Research on Alzheimer’s Disease and the Aging Brain, G.H. Sergievsky Center, Department of Neurology, Columbia University Irving Medical Center, NewYork, NY 10032, USA; Department of Psychiatry, Columbia University Irving Medical Center, New York, NY, USA; Taub Institute for Research on Alzheimer’s Disease and the Aging Brain, G.H. Sergievsky Center, Department of Neurology, Columbia University Irving Medical Center, NewYork, NY 10032, USA; Taub Institute for Research on Alzheimer’s Disease and the Aging Brain, G.H. Sergievsky Center, Department of Neurology, Columbia University Irving Medical Center, NewYork, NY 10032, USA; Taub Institute for Research on Alzheimer’s Disease and the Aging Brain, G.H. Sergievsky Center, Department of Neurology, Columbia University Irving Medical Center, NewYork, NY 10032, USA; Taub Institute for Research on Alzheimer’s Disease and the Aging Brain, G.H. Sergievsky Center, Department of Neurology, Columbia University Irving Medical Center, NewYork, NY 10032, USA; Taub Institute for Research on Alzheimer’s Disease and the Aging Brain, G.H. Sergievsky Center, Department of Neurology, Columbia University Irving Medical Center, NewYork, NY 10032, USA; Taub Institute for Research on Alzheimer’s Disease and the Aging Brain, G.H. Sergievsky Center, Department of Neurology, Columbia University Irving Medical Center, NewYork, NY 10032, USA; Taub Institute for Research on Alzheimer’s Disease and the Aging Brain, G.H. Sergievsky Center, Department of Neurology, Columbia University Irving Medical Center, NewYork, NY 10032, USA; Taub Institute for Research on Alzheimer’s Disease and the Aging Brain, G.H. Sergievsky Center, Department of Neurology, Columbia University Irving Medical Center, NewYork, NY 10032, USA; Taub Institute for Research on Alzheimer’s Disease and the Aging Brain, G.H. Sergievsky Center, Department of Neurology, Columbia University Irving Medical Center, NewYork, NY 10032, USA; Taub Institute for Research on Alzheimer’s Disease and the Aging Brain, G.H. Sergievsky Center, Department of Neurology, Columbia University Irving Medical Center, NewYork, NY 10032, USA; Taub Institute for Research on Alzheimer’s Disease and the Aging Brain, G.H. Sergievsky Center, Department of Neurology, Columbia University Irving Medical Center, NewYork, NY 10032, USA; Taub Institute for Research on Alzheimer’s Disease and the Aging Brain, G.H. Sergievsky Center, Department of Neurology, Columbia University Irving Medical Center, NewYork, NY 10032, USA; Taub Institute for Research on Alzheimer’s Disease and the Aging Brain, G.H. Sergievsky Center, Department of Neurology, Columbia University Irving Medical Center, NewYork, NY 10032, USA; Taub Institute for Research on Alzheimer’s Disease and the Aging Brain, G.H. Sergievsky Center, Department of Neurology, Columbia University Irving Medical Center, NewYork, NY 10032, USA; Taub Institute for Research on Alzheimer’s Disease and the Aging Brain, G.H. Sergievsky Center, Department of Neurology, Columbia University Irving Medical Center, NewYork, NY 10032, USA; Taub Institute for Research on Alzheimer’s Disease and the Aging Brain, G.H. Sergievsky Center, Department of Neurology, Columbia University Irving Medical Center, NewYork, NY 10032, USA; Taub Institute for Research on Alzheimer’s Disease and the Aging Brain, G.H. Sergievsky Center, Department of Neurology, Columbia University Irving Medical Center, NewYork, NY 10032, USA; Taub Institute for Research on Alzheimer’s Disease and the Aging Brain, G.H. Sergievsky Center, Department of Neurology, Columbia University Irving Medical Center, NewYork, NY 10032, USA; Taub Institute for Research on Alzheimer’s Disease and the Aging Brain, G.H. Sergievsky Center, Department of Neurology, Columbia University Irving Medical Center, NewYork, NY 10032, USA

**Keywords:** TSPO PET, microglia, amyloid, tau, neurodegeneration

## Abstract

Microglia monitor and respond to the brain’s microenvironment to maintain homeostasis. However, in Alzheimer’s disease and related dementias, chronically pro-inflammatory microglia may contribute to pathology. We hypothesized that inflammatory alterations, measured as microglia density via 18 kDa translocator PET, would be elevated with a topography similar to tau, be most strongly associated with tau compared to amyloid and neurodegeneration, and mediate pathways among amyloid, tau and neurodegeneration. Participants (21 cognitively unimpaired, 25 cognitively impaired) from the Longitudinal Imaging of Microglial Activation in Different Clinical Variants of Alzheimer’s Disease study underwent baseline amyloid PET (Florbetaben standard uptake value ratio), tau PET (MK6240 standard uptake value ratio), 18 kDa translocator PET (ER176 standard uptake value ratio) and structural MRI (grey matter volume). Biomarkers were quantified in 13 *a priori* regions of interest. Cognitive assessments and consensus diagnoses were performed at the Columbia Alzheimer’s Disease Research Center with biomarker information when available to define cognitive impairment. We evaluated cross-sectional regional colocalization of microglia density and amyloid, tau and neurodegeneration biomarker elevations in cognitively impaired individuals compared to amyloid-negative cognitively unimpaired individuals, microglia density associations with amyloid, tau and neurodegeneration biomarkers and microglia density mediation pathways among amyloid, tau and neurodegeneration. Exploratory analyses were stratified by amyloid positivity. Across all cognitively impaired individuals with different underlying brain microenvironments to which microglia are sensitive, higher microglia density colocalized with greater tau (10 regions) more often than with amyloid (8 regions) and neurodegeneration (4 regions), was associated with greater tau (β = 0.29–0.67 in cingulate, lingual and parietal regions) and neurodegeneration (β = −3.6 to −0.14 in limbic and medial temporal regions), and mediated tau-associated neurodegeneration (β = −0.44 to −0.26 in limbic, temporal and parietal regions). In the context of amyloid-positivity, microglia may also mediate amyloid-associated tau (β = 0.24–0.25 in parietal regions) and tau spreading (β = 0.09–0.12 across progressive Braak stage regions), whereas amyloid may not be necessary for tau-associated neurodegeneration, particularly in limbic regions (β = −0.46 to −0.37 in amyloid-negative individuals with cognitive impairment alone). Glia may represent a promising target for intervening on tau-associated neurodegeneration across individuals with cognitive impairment.

## Teaser

Lao *et al.* report that microglia density was elevated with similar topography to tau, and greater microglia density was associated with greater neurodegeneration and tau. In the hippocampus, amygdala, temporal cortex and parietal cortex, greater microglia density mediated tau-associated neurodegeneration. Results were dependent on amyloid positivity status.

## Graphical Abstract

**Graphical Abstract:**
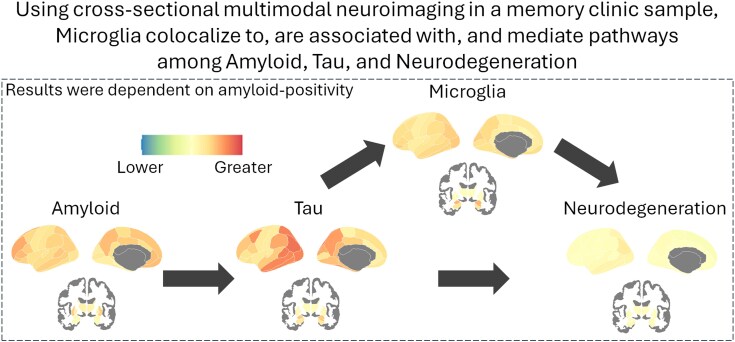

## Introduction

A comprehensive framework for Alzheimer’s disease (AD) that integrates proteopathic processes (such as the misfolding, aggregation and spreading of amyloid and tau) with immunopathic mechanisms (including molecular and cellular components) could uncover alternative modifiable targets for preventing neurodegeneration and cognitive decline, beyond the traditional focus on amyloid and tau (for reviews, see^1,2^). Microglia are one of the brain’s resident immune cells, continuously monitoring, clearing lipid debris and responding to stimuli, including infection, toxins and injury, thereby contributing to neuroprotection and repair. However, microglial function depends on the brain’s microenvironment, which can vary significantly under different physiological conditions. Studies have suggested an overall biphasic role in Alzheimer’s disease and related dementias (ADRD) progression in which microglia performing their surveillance and clearance functions protect and maintain the brain,^3^ but after chronic activation (e.g. shift in expression of surface receptors and effector molecules towards pro-inflammatory states) or dysfunction (e.g. loss of phagocytic and degradative capability), microglia may promote disease pathology themselves and through their cross-talk with neurons, endothelium and other inflammatory cells.^4^

Our objective was to leverage multimodal neuroimaging in a memory clinic sample to investigate the disease-promoting role of microglia in relation to the brain microenvironment (i.e. amyloid, tau and neurodegeneration). TSPO (18 kDa translocator protein) is primarily expressed in microglia, but also in astrocytes and infiltrating macrophages, although at a lower level, and even in non-immune cell types such as endothelial cells.^5-7^ TSPO PET imaging reflects microglial density and recruitment more than function in humans^8^ and correlates with functional and microglia-specific inflammatory markers, including sTREM2^9^ and CD68.^7^ Therefore, we utilize TSPO PET imaging to broadly map microglia-associated ‘inflammatory alterations’ across the brain. In samples with mild cognitive impairment (MCI) and late-onset AD, greater TSPO was associated with tau spreading across Braak stage regions, neurodegeneration and cognition.^9,10^ In early-onset MCI, a younger, more homogeneous group with less age-related copathology, a study demonstrated a regional distribution of elevated TSPO similar to that of tau and neurodegeneration, a stronger association with tau compared to amyloid and neurodegeneration and a correlation to memory impairment.^11^ In other neurodegenerative disorders, TSPO was elevated in key motor regions in Progressive Apraxia of Speech with Parkinson-plus features, correlated with worse Parkinsonism, and correlated with greater tau.^12^ Similarly, TSPO followed the distribution of tau across functionally connected brain regions in individuals with 4R tauopathies.^13^ TSPO studies across various neurodegenerative disorders have revealed consistent increases in key brain regions that correlated with symptomatology, suggesting that greater microglia density can be disease-promoting.^14,15^

Here, we sought to generalize microglia associations across various ADRD diagnoses that represent different brain microenvironments using simplified quantification for the novel TSPO radiotracer, ER176. Identifying common microglia-associated inflammatory alterations can inform therapeutic strategies for application across ADRD diagnoses. We hypothesized that within a cohort characterized at the Columbia Alzheimer’s Disease Research Center (ADRC) comprising individuals with MCI, AD and posterior cortical atrophy (PCA) among other clinical variants, ER176 would be (i) elevated in ADRD across key brain regions similarly to tau, (ii) most strongly associated with tau compared to amyloid and neurodegeneration and (iii) a mediator of associations between amyloid, tau and neurodegeneration.

## Materials and methods

### Participants

Participants in the Longitudinal Imaging of Microglial Activation in Different Clinical Variants of Alzheimer’s Disease study (R01AG063888) were co-enrolled in the Columbia Alzheimer’s Disease Research Center (P30AG066462). Participants underwent baseline amyloid PET, tau PET, TSPO PET, structural MRI and ADRC cognitive testing. Longitudinal data collection is ongoing. Briefly, participants were evaluated with the Mini-Mental State Examination (MMSE),^16^ and domain-specific tests including the Selective Reminding Test,^17^ Trail Making Test^18^ and categorical fluency.^19^ Domain-specific cognitive test scores were transformed into z-scores using age-, sex- and education-adjusted normative data derived for the National Alzheimer’s Coordinating Center Uniform Dataset (NACC; see Supplementary Materials for individual tests included in each cognitive domain score).^20^ At Columbia ADRC case consensus, participants were categorized as cognitively unimpaired (*n* = 21), amnestic or non-amnestic MCI (*n* = 8), amnestic multi-domain dementia syndrome referred to as ‘AD dementia’ (*n* = 8), PCA (*n* = 6), logopenic variant of primary progressive aphasia (lvPPA; *n* = 1), limbic-predominant age-related TDP43 encephalopathy (LATE; *n* = 1) and frontotemporal dementia (FTD; *n* = 1). All cognitively unimpaired older adults were amyloid-negative. Biomarker data were used in case consensus when available. All participants (or their legally authorized representatives) provided informed consent according to the Declaration of Helsinki. The Institutional Review Board of Columbia University Irving Medical Center gave ethical approval for this work.

The novel third-generation TSPO PET radiotracer ER176 exhibits favourable pharmacokinetic properties.^21,22^ Notably, ER176 has high signal-to-noise, no radiometabolites that enter the brain and is less sensitive to the single-nucleotide polymorphism in the TSPO gene (rs6971) that precluded the use of TSPO PET radiotracers for ∼ 10% of the population^23^; however, TSPO binding affinity still needs to be statistically corrected for. Briefly, genomic DNA from each subject was used to genotype the rs6971 single-nucleotide polymorphism (SNP) using a TaqMan assay.^24^ Participants included high-affinity binders (54%), mixed-affinity binders (33%) and low-affinity binders (13%), aligning with previously reported frequencies.^24^ APOE genotyping was determined using the KASPar® PCR SNP genotyping system (LGC Genomics) for the rs7412 and rs429358 SNPs. Genotype data for these two SNPs were used to unambiguously define ε2, ε3 and ε4 alleles. APOE information was missing in three participants (one cognitively unimpaired adult, two ADRD). Participants with at least one ε4 allele were categorized as APOE4 carriers.

### Neuroimaging

Structural T1 MRI was performed in a 3T GE Signa Premier scanner (repetition time [TR]: 6.6 ms, echo time [TE]: 3 ms, voxel size =1 × 1 × 1 mm^3^). Regions of interest were defined using the Hammers-N30R83-1 MM atlas in the PNEURO module of PMOD 3.9 (PMOD Technologies^25^), except for the entorhinal cortex, which was defined using the Desikan–Killiany atlas in FreeSurfer 6.0 (Massachusetts General Hospital, Harvard Medical School; http://surfer.nmr.mgh.harvard.edu). Grey matter volumes were calculated as the bilateral average for 13 *a priori* regions segmented in native MRI space (prefrontal cortex, insula, cingulate gyrus, fusiform, lingual gyrus, entorhinal cortex, middle inferior temporal gyrus, superior temporal gyrus, inferior parietal cortex, superior parietal cortex, amygdala, hippocampus and striatum [see Supplementary Materials for region of interest or ROI definitions]).

All PET scans were performed in a Siemens Biograph64 mCT/PET scanner, reconstructed with OSEM (voxel size = 1 × 1 × 2 mm^3^) and corrected for radioactive decay, attenuation, scatter, random events and scanner deadtime and normalization. All preprocessing steps (i.e. frame realignment, coregistration with segmented MRI, standard uptake value ratio (SUVR) calculation) and partial volume correction using the voxelwise Geometric Transfer Method were performed in PMOD.^26^ Amyloid PET was performed using Florbetaben (8.1 mCi; FBB), and FBB SUVR was calculated using 90–110 min data and the whole cerebellum as the reference region. Tau PET was performed using MK-6240 (5 mCi), and MK-6240 SUVR was calculated using 90–110 min data and inferior cerebellar grey matter as the reference region. Tau PET was missing in 5 participants (1 cognitively unimpaired adult, 4 ADRD). TSPO PET was performed using ER176 (up to 20 mCi), and ER176 SUVR was calculated using 60–90 min data and the whole cerebellum as a pseudo-reference region, as previously assessed on the imaging^27,28^ and pathological levels.^7,29^ TSPO PET was missing in one participant (one ADRD). All PET SUVRs were averaged bilaterally in the same regions of interest as grey matter volumes.

### Statistical analysis

Demographic characteristics, biomarker levels and clinical characteristics were compared between amyloid-negative cognitively unimpaired adults and individuals with ADRD. For descriptive purposes, amyloid positivity was rated visually according to vendor instructions.^30^ Tau positivity across Braak I/II was categorized as two standard deviations above the mean of MK6240 SUVR in cognitively unimpaired adults, following previous reports.^31^ Given that every individual has microglia, TSPO positivity across a composite brain region was simplistically defined as above the mean in amyloid-negative cognitively unimpaired adults by binding affinity groups (Supplementary Materials; Supplementary Fig. S1). For analytic purposes, all biomarkers were used continuously. To assess regional colocalization of biomarker elevations, we compared participants with ADRD to amyloid-negative cognitively unimpaired adults in separate ANCOVAs per biomarker and counted the number of regions where elevated TSPO and elevated amyloid, tau and neurodegeneration were colocalized. To assess regional biomarker associations, we included amyloid, tau and neurodegeneration by ROI interactions as simultaneous predictors of TSPO in a single general linear model. To assess regional mechanistic pathways, we included mediations from amyloid to TSPO to tau within ROI (i.e. TSPO-mediated amyloid-associated tau); tau in earlier Braak stages to composite TSPO to tau in subsequent Braak stages (i.e. TSPO-mediated tau spreading) and tau to TSPO to neurodegeneration within ROI (i.e. TSPO-mediated tau-associated neurodegeneration). To support causal inference from cross-sectional mediations, we also assessed alternative directions where TSPO may initiate key pathways (TSPO to amyloid to tau; TSPO to earlier Braak tau to later Braak tau; TSPO to tau to neurodegeneration). We performed exploratory analyses, stratifying by amyloid positivity, to understand the different brain microenvironments in which associations were present. All models were adjusted for age, sex, body mass index (BMI) and APOE4 status.^32^ Models with ER176 SUVR were further adjusted for TSPO binding affinity and models with grey matter volume were further adjusted for intracranial total volume. All estimates were standardized for a measure of effect size and to facilitate comparison across models. We ran three sets of sensitivity analyses. We repeated all analyses using a generalized linear model following a gamma distribution and log link or mediations using a log transform to reduce potential bias from very high biomarker values, particularly in the amyloid-positive individuals with ADRD. We reassessed regional biomarker associations, including amyloid, tau and neurodegeneration in separate, univariate general linear models to address collinearity. We further repeated all analyses using bilateral regions across the brain to assess the robustness of our findings, which were limited to 13 *a priori* regions to reduce multiple comparisons. Statistical models were adjusted for multiple comparisons using the multivariate t distribution for ANCOVAs and linear models or the False Detection Rate method for mediations and run in R 4.4.1.

## Results

### Participant characteristics

Amyloid-negative cognitively unimpaired adults and individuals with ADRD were similar in age, sex, BMI, race, ethnicity, TSPO affinity and APOE genotype (Table 1). By definition, individuals with ADRD had lower MMSE and cognitive domain scores, with the greatest impairment in visuospatial ability, followed by delayed episodic memory, immediate episodic memory, attention, executive function and language (Supplementary Table S1). As expected, individuals with ADRD were more likely to be amyloid, tau and TSPO-positive. Out of 21 individuals without cognitive impairment, 48% were amyloid-negative, tau-negative, TSPO-negative, while 48% were amyloid-negative, tau-negative, TSPO-positive (one cognitively unimpaired adult was missing their tau PET). This is a result of using the mean TSPO in cognitively unimpaired adults as a threshold for context, but analytic models use all biomarkers continuously. Out of 25 individuals with ADRD, 44% were amyloid-positive, tau-positive and TSPO-positive; 4% were amyloid-positive, tau-positive and TSPO-negative; 8% were amyloid-negative, tau-positive and TSPO-positive and 20% were amyloid-negative, tau-negative and TSPO-positive (for biomarker positivity profile by ADRD diagnosis, see Supplementary Fig. S1).

**Table 1:** Participant characteristics

	Cognitively unimpaired	ADRD	Test statistic, *P*-value
*N* (%)	21 (45.7)	25 (54.3)	–
Age	54–80 70.3 ± 5.9	53–86 70.1 ± 8.3	F(1,44) < 0.01, *P* = 0.92
Sex	10 (47.6%) women 11 (52.4%) men	11 (44%) women 14 (56%) men	χ^2^(1) < 0.01, *P* > 0.99
BMI	21–31.3 25.1 ± 3.1	19.1–40.6 26.7 ± 5.5	*F*(1,43) = 1.4, *P* = 0.25
Education	12–20 16.3 ± 2.1	11–22 16.5 ± 3.4	*F*(1,44) = 0.03, *P* = 0.86
Race	17 (81%) White 3 (14.3%) Black 1 (4.8%) Mixed	23 (92%) White 1 (4%) Black 1 (4%) Asian	χ^2^(3) = 3.6, *P* = 0.31
Ethnicity	19 (90.5%) Non-Hispanic 2 (9.5%) Hispanic	23 (92%) Non-Hispanic 2 (8%) Hispanic	χ^2^(1) < 0.01, *P* > 0.99
APOE	13 (65%) E3/E3 7 (35%) E3/E4	1 (4.3%) E3/E2 11 (47.8%) E3/E3 7 (30.4%) E3/E4 2 (8.7%) E4/E2 2 (8.7%) E4/E4	χ^2^(2) = 0.4, *P* = 0.81
TSPO genotype	2 (9.5%) Low affinity 7 (33.3%) Mixed affinity 12 (57.1%) High affinity	4 (16%) Low affinity 8 (32%) Mixed affinity 13 (52%) High affinity	χ^2^(4) = 5, *P* = 0.29
Amyloid positivity	21 (100%) Amyloid negative 0 (0%) Amyloid positive	10 (40%) Amyloid negative 15 (60%) Amyloid positive	**χ^2^(1)** **=** **16.1, *P*** **=** **6e−05**
Tau positivity	19 (95%) Tau negative 1 (5%) Tau positive	5 (23.8%) Tau negative 16 (76.2%) Tau positive	**χ^2^(1)** **=** **18.6, *P*** **=** **2e−05**
TSPO positivity	10 (47.6%) TSPO negative 11 (52.4%) TSPO positive	3 (12.5%) TSPO negative 21 (87.5%) TSPO positive	**χ^2^(1)** **=** **5.1, *P*** **=** **0.02**

Demographic characteristics for cognitively unimpaired adults and individuals with Alzheimer’s disease and related dementias (ADRD). Bold indicates significant (*P* < 0.05).

### Colocalization of TSPO with amyloid, tau and neurodegeneration

**Figure 1:**
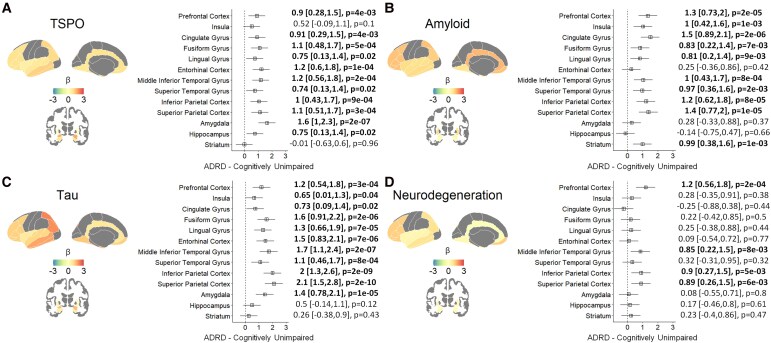
**Regional biomarker elevation.** Standardized effect size (beta) for the difference between participants with Alzheimer’s disease and related dementias (ADRD) and cognitively unimpaired adults across each of the 13 *a priori* regions for (A) 18 kDa translocator protein (TSPO), (B) amyloid, (C) tau and (D) neurodegeneration. Note that neurodegeneration is an inverted grey matter volume for direct visual comparison to other biomarkers. General linear model results are corrected for multiple comparisons. TSPO *N* = 41, amyloid *N* = 42, tau *N* = 38, neurodegeneration *N* = 39.

Comparing individuals with ADRD to amyloid-negative cognitively unimpaired adults, elevated TSPO was present in 11 regions (Fig. 1A). The spatial colocalization of elevated TSPO with elevated tau (10 regions; Fig. 1A and C) was greater than that with amyloid (8 regions; Fig. 1A and B) and neurodegeneration (4 regions; Fig. 1A and D). The middle inferior temporal gyrus, prefrontal cortex, inferior parietal cortex and superior parietal cortex had elevated TSPO, amyloid, tau and neurodegeneration. The cingulate gyrus, fusiform gyrus, lingual gyrus and superior temporal gyrus had elevated TSPO, amyloid and tau. The entorhinal cortex and amygdala had elevated TSPO and tau. The hippocampus had elevated TSPO alone.

### Associations of TSPO with amyloid, tau and neurodegeneration

**Figure 2:**
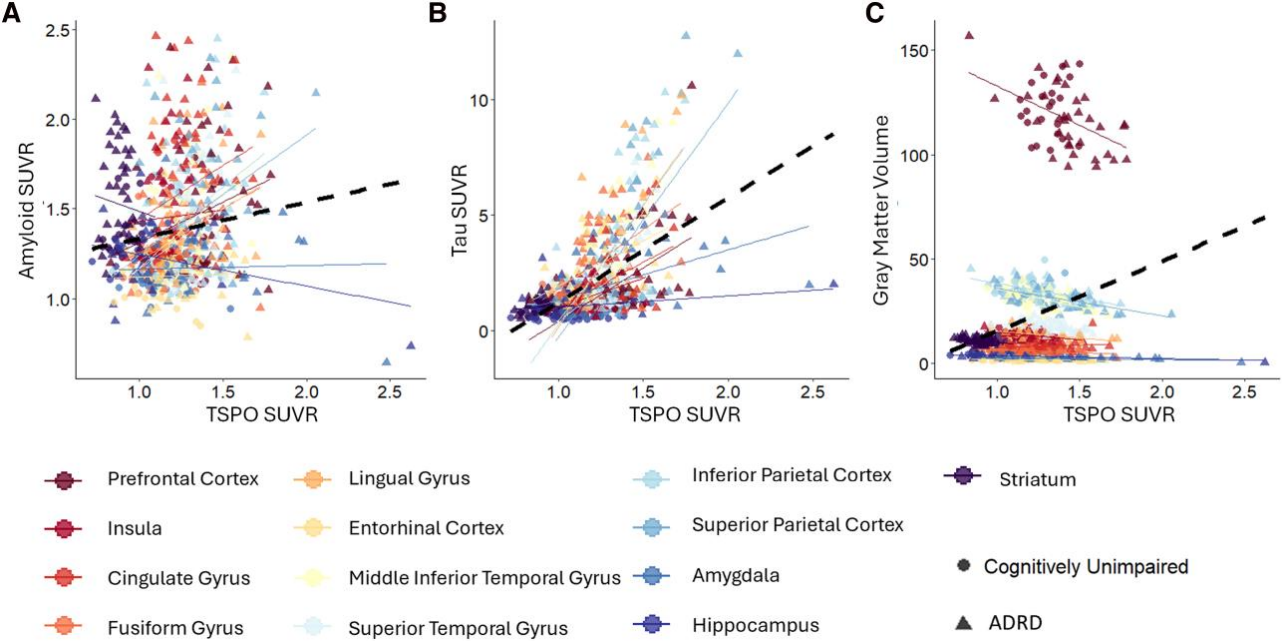
**Regional biomarker scatterplots.** Regional (straight lines) and overall (dotted lines) associations of 18 kDa translocator protein (TSPO) with (A) amyloid, (B) tau and (C) grey matter volume across cognitively unimpaired adults (circles) and individuals with Alzheimer’s disease and related dementias (ADRD; triangles), indicating spatially specific associations between biomarker pairs. For example, with grey matter volume, the overall positive association indicates that larger regions have greater TSPO, but within regions, greater TSPO is associated with lower grey matter volume. General linear model results with all biomarkers entered simultaneously (*N* = 37) are corrected for multiple comparisons and presented in Table 2. Greater TSPO was associated with greater tau in cingulate (standardized slope = 0.67 [0.31, 1], *P* = 0.004), lingual (0.35 [0.14, 0.57], *P* = 0.02), inferior parietal (0.3 [0.11, 0.48], *P* = 0.02) and superior parietal (0.29 [0.11, 0.46], *P* = 0.01) regions (B) and with lower grey matter volume in amygdala (−3.6 [−4.5, −2.6], *P* < 0.001), hippocampus (−2.3 [−2.9, −1.7], *P* < 0.001), entorhinal (−1.6 [−2.6, −0.52], *P* = 0.04), fusiform (−0.61 [−0.98, −0.24], *P* = 0.01) and middle inferior temporal (−0.14 [−0.23, −0.04], *P* = 0.05) regions (C). SUVR = standard uptake value ratio.

Across amyloid-negative cognitively unimpaired adults and individuals with ADRD, TSPO was not associated with amyloid (Fig. 2A). Greater TSPO was associated with greater tau in cingulate (standardized slope = 0.67 [0.31, 1], *P* = 0.004), lingual (0.35 [0.14, 0.57], *P* = 0.02), inferior parietal (0.3 [0.11, 0.48], *P* = 0.02) and superior parietal (0.29 [0.11, 0.46], *P* = 0.01; Fig. 2B) regions and with greater neurodegeneration (i.e. lower grey matter volume) in amygdala (−3.6 [−4.5, −2.6], *P* < 0.001), hippocampus (−2.3 [−2.9, −1.7], *P* < 0.001), entorhinal (−1.6 [−2.6, −0.52], *P* = 0.04), fusiform (−0.61 [−0.98, −0.24], *P* = 0.01) and middle inferior temporal (−0.14 [−0.23, −0.04], *P* = 0.05) regions (Fig. 2C; Table 2).

**Table 2:** Regional biomarker associations

	Amyloid	Tau	Grey matter volume
Prefrontal cortex	−0.26 [−1.3, 0.81], *P* > 0.99	0.29 [0.07, 0.5], *P* = 0.11	−0.03 [−0.06, −0.01], *P* = 0.18
Insula	−0.3 [−1.6, 1], *P* > 0.99	0.1 [−0.4, 0.61], *P* > 0.99	−0.27 [−0.47, −0.06], *P* = 0.14
Cingulate gyrus	−0.53 [−1.5, 0.5], *P* = 0.99	**0.67 [0.31, 1], *P*** **=** **4e−03**	0.11 [−0.03, 0.26], *P* = 0.8
Fusiform gyrus	0.3 [−1.2, 1.8], *P* > 0.99	0.2 [−0.08, 0.48], *P* = 0.9	**−0.61 [−0.98, −0.24], *P*** **=** **0.01**
Lingual gyrus	−0.83 [−2.4, 0.76], *P* = 0.99	**0.35 [0.14, 0.57], *P*** **=** **0.02**	−0.28 [−0.49, −0.08], *P* = 0.07
Entorhinal cortex	−1.5 [−3.9, 0.87], *P* = 0.95	0.35 [0.04, 0.65], *P* = 0.28	**−1.6 [−2.6, −0.52], *P*** **=** **0.04**
Middle inferior temporal gyrus	−0.15 [−1.5, 1.2], *P* > 0.99	0.26 [0.03, 0.48], *P* = 0.27	**−0.14 [−0.23, −0.04], *P*** **=** **0.05**
Superior temporal gyrus	−0.31 [−1.7, 1], *P* > 0.99	0.26 [−0.05, 0.56], *P* = 0.76	−0.19 [−0.37, −0.01], *P* = 0.35
Inferior parietal cortex	−0.63 [−1.8, 0.56], *P* = 0.99	**0.3 [0.11, 0.48], *P*** **=** **0.02**	−0.06 [−0.15, 0.02], *P* = 0.88
Superior parietal cortex	−0.7 [−1.9, 0.46], *P* = 0.97	**0.29 [0.11, 0.46], *P*** **=** **0.01**	−0.1 [−0.19, 0], *P* = 0.44
Amygdala	0.71 [−1.6, 3.1], *P* > 0.99	0.21 [−0.11, 0.52], *P* = 0.93	**−3.6 [−4.5, −2.6], *P*** **=** **2e−12**
Hippocampus	−1.2 [−3.8, 1.4], *P* > 0.99	−0.1 [−0.88, 0.68], *P* > 0.99	**−2.3 [−2.9, −1.7], *P*** **=** **3e−12**
Striatum	−0.12 [−1.7, 1.5], *P* > 0.99	−0.37 [−2, 1.3], *P* > 0.99	−0.16 [−0.4, 0.08], *P* = 0.94

Standardized estimates (beta) for biomarker regressions across cognitively unimpaired adults and individuals with Alzheimer’s disease and related dementias (ADRD). Amyloid, tau and neurodegeneration across regions of interest are simultaneous predictors of TSPO. General linear model results with all biomarkers entered simultaneously (*N* = 37) are corrected for multiple comparisons. Bold indicates significance (*P* < 0.05).

### Mediations of TSPO among amyloid, tau and neurodegeneration

Assessing specific pathways, TSPO mediated the association between tau and neurodegeneration in the middle inferior temporal gyrus (standardized indirect effect: −0.44 [−0.67, −0.2], *P* = 0.003), hippocampus (−0.31 [−0.55, −0.07], *P* = 0.05), amygdala (−0.31 [−0.54, −0.08], *P* = 0.05) and superior parietal cortex (−0.26 [−0.47, −0.05], *P* = 0.05; Table 3), while the alternative mediation (tau mediated TSPO-associated neurodegeneration) was not present. Early tau mediated the association between composite TSPO and intermediate tau (0.47 [0.24, 0.71], *P* < 0.001), while intermediate tau mediated the association between composite TSPO and late tau (0.59 [0.36, 0.83], *P* < 0.001; Supplementary Table S2).

**Table 3:** Pathway mediations

	Exposure->Mediator	Mediator->Outcome	Exposure->Outcome	Exposure->Mediator->Outcome	Total	Proportion mediated
	Amyloid->TSPO	TSPO->Tau	Amyloid->Tau	Amyloid->TSPO->Tau		
Prefrontal cortex	0.21 [−0.07, 0.49], *P* = 0.2	**0.37 [0.16, 0.58], *P*** **=** **8e−04**	**0.53 [0.31, 0.74], *P*** **=** **5e−06**	0.08 [−0.04, 0.19], *P* = 0.24	**0.6 [0.37, 0.84], *P*** **=** **2e−06**	12.8%
Insula	−0.04 [−0.4, 0.32], *P* = 0.89	0.17 [−0.04, 0.39], *P* = 0.12	**0.51 [0.27, 0.75], *P*** **=** **7e−05**	−0.01 [−0.07, 0.06], *P* = 0.92	**0.5 [0.25, 0.75], *P*** **=** **1e−04**	1.4%
Cingulate gyrus	0.3 [−0.05, 0.65], *P* = 0.2	**0.27 [0.12, 0.42], *P*** **=** **8e−04**	**0.43 [0.25, 0.61], *P*** **=** **8e−06**	0.08 [−0.02, 0.18], *P* = 0.24	**0.51 [0.31, 0.71], *P*** **=** **2e−06**	15.8%
Fusiform gyrus	0.01 [−0.36, 0.38], *P* = 0.95	**0.36 [0.14, 0.57], *P*** **=** **1e−03**	**0.47 [0.22, 0.72], *P*** **=** **3e−04**	0 [−0.13, 0.14], *P* = 0.95	**0.47 [0.19, 0.75], *P*** **=** **1e−03**	0.9%
Lingual gyrus	0.28 [0, 0.57], *P* = 0.17	**0.5 [0.34, 0.66], *P*** **=** **2e−08**	**0.35 [0.2, 0.5], *P*** **=** **2e−05**	0.14 [−0.01, 0.29], *P* = 0.21	**0.49 [0.28, 0.7], *P*** **=** **7e−06**	28.8%
Entorhinal cortex	−0.27 [−0.63, 0.1], *P* = 0.2	**0.57 [0.34, 0.81], *P*** **=** **4e−06**	**0.45 [0.17, 0.72], *P*** **=** **1e−03**	−0.15 [−0.37, 0.06], *P* = 0.24	0.29 [−0.05, 0.63], *P* = 0.1	52.3%
Middle inferior temporal gyrus	0.31 [−0.05, 0.68], *P* = 0.2	**0.48 [0.31, 0.65], *P*** **=** **2e−07**	**0.4 [0.2, 0.6], *P*** **=** **1e−04**	0.15 [−0.03, 0.33], *P* = 0.24	**0.55 [0.29, 0.81], *P*** **=** **6e−05**	27.2%
Superior temporal gyrus	0.24 [−0.08, 0.55], *P* = 0.2	**0.31 [0.14, 0.47], *P*** **=** **8e−04**	**0.45 [0.26, 0.65], *P*** **=** **1e−05**	0.07 [−0.03, 0.18], *P* = 0.24	**0.53 [0.31, 0.74], *P*** **=** **5e−06**	13.9%
Inferior parietal cortex	**0.38 [0.12, 0.64], *P*** **=** **0.02**	**0.46 [0.28, 0.64], *P*** **=** **3e−06**	**0.47 [0.3, 0.65], *P*** **=** **2e−06**	0.18 [0.04, 0.32], *P* = 0.09	**0.65 [0.44, 0.86], *P*** **=** **1e−08**	27.2%
Superior parietal cortex	**0.37 [0.12, 0.61], *P*** **=** **0.02**	**0.48 [0.27, 0.68], *P*** **=** **1e−05**	**0.48 [0.29, 0.67], *P*** **=** **3e−06**	0.17 [0.04, 0.31], *P* = 0.09	**0.65 [0.44, 0.87], *P*** **=** **1e−08**	26.7%
Amygdala	−0.27 [−0.61, 0.08], *P* = 0.2	**0.46 [0.24, 0.68], *P*** **=** **9e−05**	**0.41 [0.17, 0.65], *P*** **=** **8e−04**	−0.12 [−0.29, 0.05], *P* = 0.24	0.29 [0, 0.57], *P* = 0.06	43%
Hippocampus	**−0.65 [−0.99, −0.32], *P*** **=** **1e−03**	**0.29 [0.04, 0.54], *P*** **=** **0.03**	−0.03 [−0.32, 0.27], *P* = 0.85	−0.19 [−0.38, 0], *P* = 0.21	−0.22 [−0.49, 0.06], *P* = 0.12	86.6%
Striatum	−0.05 [−0.36, 0.25], *P* = 0.86	−0.03 [−0.29, 0.23], *P* = 0.82	**0.51 [0.27, 0.76], *P*** **=** **7e−05**	0 [−0.02, 0.02], *P* = 0.92	**0.51 [0.27, 0.76], *P*** **=** **7e−05**	0.3%

	Tau->TSPO	TSPO->Tau	Tau->Tau	Tau->TSPO->Tau		
Early to middle Braak	**0.52 [0.28, 0.77], *P*** **=** **3e−05**	**0.15 [0.02, 0.28], *P*** **=** **0.05**	**0.8 [0.66, 0.93], *P*** **<** **1e−25**	0.08 [0, 0.16], *P* = 0.09	**0.88 [0.75, 1], *P*** **<** **1e−25**	9%
Middle to late Braak	**0.63 [0.39, 0.87], *P*** **=** **5e−07**	0.07 [−0.03, 0.17], *P* = 0.18	**0.87 [0.77, 0.98], *P*** **<** **1e−25**	0.04 [−0.02, 0.11], *P* = 0.19	**0.92 [0.83, 1], *P*** **<** **1e−25**	4.7%

	Tau->TSPO	TSPO->Grey matter volume	Tau->Grey matter volume	Tau->TSPO->Grey matter volume		
Prefrontal cortex	**0.48 [0.23, 0.74], *P*** **=** **4e−04**	**−0.38 [−0.64, −0.12], *P*** **=** **9e−03**	−0.12 [−0.4, 0.16], *P* = 0.44	−0.18 [−0.34, −0.02], *P* = 0.06	**−0.3 [−0.56, −0.04], *P*** **=** **0.03**	61%
Insula	0.22 [−0.15, 0.6], *P* = 0.26	**−0.35 [−0.56, −0.15], *P*** **=** **3e−03**	−0.28 [−0.54, −0.02], *P* = 0.09	−0.08 [−0.22, 0.06], *P* = 0.29	**−0.36 [−0.65, −0.07], *P*** **=** **0.02**	22.2%
Cingulate gyrus	**0.74 [0.36, 1.1], *P*** **=** **3e−04**	0.35 [−0.03, 0.73], *P* = 0.09	−0.4 [−0.98, 0.18], *P* = 0.29	0.26 [−0.05, 0.57], *P* = 0.14	−0.14 [−0.65, 0.38], *P* = 0.6	188.9%
Fusiform gyrus	**0.49 [0.17, 0.81], *P*** **=** **4e−03**	**−0.43 [−0.69, −0.17], *P*** **=** **3e−03**	−0.14 [−0.44, 0.17], *P* = 0.44	−0.21 [−0.4, −0.02], *P* = 0.06	**−0.35 [−0.66, −0.04], *P*** **=** **0.03**	60.1%
Lingual gyrus	**0.86 [0.61, 1.1], *P*** **=** **9e−11**	**−0.31 [−0.59, −0.04], *P*** **=** **0.04**	−0.16 [−0.49, 0.17], *P* = 0.44	−0.27 [−0.52, −0.02], *P* = 0.06	**−0.42 [−0.67, −0.18], *P*** **=** **1e−03**	63.1%
Entorhinal cortex	**0.55 [0.28, 0.82], *P*** **=** **2e−04**	−0.23 [−0.49, 0.02], *P* = 0.09	**−0.36 [−0.63, −0.08], *P*** **=** **0.04**	−0.13 [−0.28, 0.03], *P* = 0.14	**−0.48 [−0.72, −0.24], *P*** **=** **2e−04**	26.5%
Middle inferior temporal gyrus	**0.82 [0.54, 1.1], *P*** **=** **4e−08**	**−0.53 [−0.75, −0.32], *P*** **=** **6e−06**	−0.13 [−0.39, 0.13], *P* = 0.44	**−0.44 [−0.67, −0.2], *P*** **=** **3e−03**	**−0.56 [−0.81, −0.32], *P*** **=** **3e−05**	**77**.**5%**
Superior temporal gyrus	**0.62 [0.3, 0.93], *P*** **=** **3e−04**	−0.22 [−0.44, 0.01], *P* = 0.09	−0.36 [−0.67, −0.04], *P* = 0.08	−0.13 [−0.29, 0.02], *P* = 0.14	**−0.49 [−0.77, −0.21], *P*** **=** **1e−03**	27.1%
Inferior parietal cortex	**0.74 [0.53, 0.94], *P*** **=** **1e−11**	−0.19 [−0.51, 0.12], *P* = 0.25	**−0.45 [−0.79, −0.11], *P*** **=** **0.04**	−0.14 [−0.38, 0.09], *P* = 0.28	**−0.59 [−0.85, −0.33], *P*** **=** **3e−05**	23.9%
Superior parietal cortex	**0.67 [0.47, 0.86], *P*** **=** **2e−10**	**−0.39 [−0.68, −0.1], *P*** **=** **0.02**	−0.3 [−0.59, −0.01], *P* = 0.09	**−0.26 [−0.47, −0.05], *P*** **=** **0.05**	**−0.56 [−0.8, −0.32], *P*** **=** **3e−05**	**46**.**3%**
Amygdala	**0.51 [0.18, 0.84], *P*** **=** **3e−03**	**−0.61 [−0.83, −0.4], *P*** **=** **2e−07**	−0.06 [−0.32, 0.2], *P* = 0.63	**−0.31 [−0.54, −0.08], *P*** **=** **0.05**	**−0.38 [−0.69, −0.07], *P*** **=** **0.02**	**82**.**9%**
Hippocampus	**0.54 [0.14, 0.93], *P*** **=** **9e−03**	**−0.57 [−0.73, −0.41], *P*** **=** **3e−11**	**−0.37 [−0.59, −0.15], *P*** **=** **0.01**	**−0.31 [−0.55, −0.07], *P*** **=** **0.05**	**−0.68 [−0.98, −0.37], *P*** **=** **4e−05**	**45**.**6%**
Striatum	−0.07 [−0.39, 0.26], *P* = 0.69	−0.12 [−0.39, 0.15], *P* = 0.38	−0.27 [−0.57, 0.02], *P* = 0.13	0.01 [−0.04, 0.05], *P* = 0.71	−0.26 [−0.56, 0.03], *P* = 0.09	3.1%

Standardized pathway estimates (beta) in all participants with Alzheimer’s disease and related dementias (ADRD) with TSPO as the mediator. Mediation model (*N* = 37) results are corrected for multiple comparisons. Note: Proportion mediated may exceed 100% in some regions, indicating unstable estimates or inconsistent mediation, where the direct and indirect effects have opposite signs. Bold indicates significance (*P* < 0.05).

### Exploratory analysis: stratification by amyloid positivity

In sensitivity analyses, we assessed the role of TSPO in the context of amyloid-positive ADRD and amyloid-negative ADRD separately (Supplementary Table S1). Amyloid-positive ADRD nor amyloid-negative ADRD differed from cognitively unimpaired adults in demographic characteristics. While not significant, it is interesting to note that 64% of amyloid-positive ADRD were APOE4 carriers and 78% of amyloid-negative ADRD were not APOE4 carriers. By definition, amyloid-positive ADRD were more likely to be amyloid-positive compared to amyloid-negative cognitively unimpaired adults (100% versus 0%); they were also more likely to be tau-positive (100% versus 5%), but not TSPO-positive (86% versus 52%). Similarly, amyloid-negative ADRD were not different from amyloid-negative cognitively unimpaired adults in amyloid positivity by definition (0% versus 0%), and were not different in tau positivity (29% versus 5%) or TSPO positivity (90% versus 52%). Amyloid-positive ADRD had the greatest impairment in visuospatial ability, followed by attention, delayed episodic memory, executive function, immediate episodic memory and language, whereas amyloid-negative ADRD had the greatest impairment in delayed episodic memory, followed by immediate episodic memory, visuospatial ability, executive function, attention and language.

Compared to amyloid-negative cognitively unimpaired adults, elevated TSPO was present in 9 regions in amyloid-positive ADRD, whereas elevated TSPO was present in 11 regions in amyloid-negative ADRD (Table 4). In amyloid-positive ADRD, elevated TSPO colocalized with elevated tau in nine regions, amyloid in nine regions and neurodegeneration in four regions. Specifically, elevated TSPO was spatially colocalized with elevated amyloid, tau and neurodegeneration in the middle inferior temporal gyrus, inferior parietal cortex, superior parietal cortex and prefrontal cortex and with elevated amyloid and tau in the amygdala, entorhinal cortex, fusiform gyrus, lingual gyrus and cingulate gyrus. There were no regions where elevated TSPO was present by itself nor any regions that colocalized with elevated amyloid alone, elevated tau alone or elevated neurodegeneration alone. In amyloid-negative ADRD, elevated TSPO colocalized with elevated tau in five regions (amygdala, hippocampus, middle inferior temporal gyrus, fusiform gyrus, entorhinal cortex), but not with elevated amyloid or neurodegeneration.

**Table 4:** Biomarker elevation by amyloid positivity

A+ ADRD	TSPO	Amyloid	Tau	Neurodegeneration
Prefrontal cortex	**0.86 [0.14, 1.6], *P*** **=** **0.02**	**3.2 [2.5, 3.9], *P*** **=** **2e−17**	**2.3 [1.5, 3], *P*** **=** **2e−09**	**1.9 [1.2, 2.7], *P*** **=** **4e−07**
Insula	0.42 [−0.3, 1.1], *P* = 0.25	**2.5 [1.8, 3.3], *P*** **=** **6e−12**	**1.2 [0.48, 1.9], *P*** **=** **1e−03**	0.22 [−0.5, 0.95], *P* = 0.55
Cingulate gyrus	**0.89 [0.16, 1.6], *P*** **=** **0.02**	**3.5 [2.8, 4.3], *P*** **=** **1e−20**	**1.4 [0.71, 2.2], *P*** **=** **1e−04**	−0.2 [−0.93, 0.53], *P* = 0.59
Fusiform gyrus	**1.4 [0.69, 2.2], *P*** **=** **1e−04**	**2.1 [1.4, 2.9], *P*** **=** **5e−09**	**2.9 [2.1, 3.6], *P*** **=** **6e−14**	0.12 [−0.6, 0.85], *P* = 0.74
Lingual gyrus	**1.1 [0.34, 1.8], *P*** **=** **4e−03**	**1.8 [1.1, 2.6], *P*** **=** **4e−07**	**2.5 [1.8, 3.3], *P*** **=** **5e−11**	0.28 [−0.44, 1], *P* = 0.44
Entorhinal cortex	**1.8 [1, 2.5], *P*** **=** **2e−06**	**0.77 [0.07, 1.5], *P*** **=** **0.03**	**2.6 [1.8, 3.3], *P*** **=** **2e−11**	0.03 [−0.69, 0.76], *P* = 0.93
Middle inferior temporal gyrus	**1.5 [0.8, 2.3], *P*** **=** **4e−05**	**2.5 [1.8, 3.2], *P*** **=** **1e−11**	**3.3 [2.5, 4.1], *P*** **=** **3e−17**	**1 [0.3, 1.8], *P*** **=** **6e−03**
Superior temporal gyrus	0.68 [−0.04, 1.4], *P* = 0.06	**2.4 [1.7, 3.1], *P*** **=** **5e−11**	**2.1 [1.4, 2.8], *P*** **=** **3e−08**	0.38 [−0.35, 1.1], *P* = 0.31
Inferior parietal cortex	**1.2 [0.5, 2], *P*** **=** **1e−03**	**3 [2.2, 3.7], *P*** **=** **2e−15**	**3.9 [3.1, 4.6], *P*** **=** **2e−22**	**1.5 [0.73, 2.2], *P*** **=** **9e−05**
Superior parietal cortex	**1.2 [0.49, 1.9], *P*** **=** **1e−03**	**3.2 [2.4, 3.9], *P*** **=** **4e−17**	**4.1 [3.3, 4.8], *P*** **=** **2e−24**	**1.3 [0.56, 2], *P*** **=** **6e−04**
Amygdala	**2.1 [1.4, 2.9], *P*** **=** **2e−08**	**1 [0.33, 1.7], *P*** **=** **4e−03**	**2.6 [1.8, 3.3], *P*** **=** **1e−11**	0.01 [−0.72, 0.73], *P* = 0.99
Hippocampus	0.51 [−0.22, 1.2], *P* = 0.17	0.33 [−0.37, 1], *P* = 0.36	**0.81 [0.08, 1.5], *P*** **=** **0.03**	0.07 [−0.66, 0.8], *P* = 0.85
Striatum	−0.2 [−0.92, 0.53], *P* = 0.59	**2.4 [1.7, 3.1], *P*** **=** **5e−11**	0.47 [−0.26, 1.2], *P* = 0.2	0.18 [−0.54, 0.91], *P* = 0.62

A− ADRD	TSPO	Amyloid	Tau	Neurodegeneration
Prefrontal cortex	**1.4 [0.57, 2.2], *P*** **=** **8e−04**	−0.33 [−1.1, 0.46], *P* = 0.41	0.72 [−0.16, 1.6], *P* = 0.11	0.4 [−0.44, 1.2], *P* = 0.35
Insula	**0.94 [0.14, 1.7], *P*** **=** **0.02**	−0.41 [−1.2, 0.38], *P* = 0.31	0.84 [−0.04, 1.7], *P* = 0.06	0.47 [−0.37, 1.3], *P* = 0.27
Cingulate gyrus	**1.4 [0.56, 2.2], *P*** **=** **8e−04**	−0.28 [−1.1, 0.51], *P* = 0.48	0.32 [−0.56, 1.2], *P* = 0.48	−0.28 [−1.1, 0.56], *P* = 0.52
Fusiform gyrus	**1.2 [0.39, 2], *P*** **=** **3e−03**	−0.49 [−1.3, 0.3], *P* = 0.22	**1.4 [0.53, 2.3], *P*** **=** **2e−03**	0.44 [−0.39, 1.3], *P* = 0.3
Lingual gyrus	0.71 [−0.08, 1.5], *P* = 0.08	−0.03 [−0.82, 0.76], *P* = 0.94	0.62 [−0.26, 1.5], *P* = 0.16	0.29 [−0.54, 1.1], *P* = 0.49
Entorhinal cortex	**1.1 [0.26, 1.9], *P*** **=** **9e−03**	−0.37 [−1.2, 0.43], *P* = 0.36	**2.7 [1.8, 3.6], *P*** **=** **5e−09**	0.26 [−0.58, 1.1], *P* = 0.55
Middle inferior temporal gyrus	**1.3 [0.47, 2.1], *P*** **=** **2e−03**	−0.29 [−1.1, 0.5], *P* = 0.47	**0.92 [0.05, 1.8], *P*** **=** **0.04**	0.78 [−0.06, 1.6], *P* = 0.07
Superior temporal gyrus	**1.2 [0.39, 2], *P*** **=** **3e−03**	−0.41 [−1.2, 0.38], *P* = 0.31	0.77 [−0.1, 1.6], *P* = 0.08	0.34 [−0.49, 1.2], *P* = 0.42
Inferior parietal cortex	**1.3 [0.51, 2.1], *P*** **=** **1e−03**	−0.35 [−1.1, 0.44], *P* = 0.38	0.57 [−0.3, 1.4], *P* = 0.2	0.29 [−0.55, 1.1], *P* = 0.5
Superior parietal cortex	**1.5 [0.72, 2.3], *P*** **=** **2e−04**	−0.09 [−0.88, 0.7], *P* = 0.82	0.78 [−0.1, 1.7], *P* = 0.08	0.51 [−0.33, 1.3], *P* = 0.23
Amygdala	**1.7 [0.93, 2.5], *P*** **=** **2e−05**	−0.75 [−1.5, 0.05], *P* = 0.06	**1.7 [0.78, 2.5], *P*** **=** **2e−04**	0.27 [−0.56, 1.1], *P* = 0.52
Hippocampus	**1.4 [0.63, 2.2], *P*** **=** **5e−04**	**−1.2 [−2, −0.37], *P*** **=** **4e−03**	**1.6 [0.68, 2.4], *P*** **=** **5e−04**	0.39 [−0.44, 1.2], *P* = 0.35
Striatum	0.32 [−0.48, 1.1], *P* = 0.43	−0.34 [−1.1, 0.45], *P* = 0.4	0.62 [−0.26, 1.5], *P* = 0.17	0.4 [−0.43, 1.2], *P* = 0.34

Standardized effect size (beta) for the difference between amyloid-positive Alzheimer’s disease and related dementias (A+ ADRD) and cognitively unimpaired adult subgroups (top) and between amyloid-negative ADRD (A− ADRD) and cognitively unimpaired adult subgroups (bottom) across each of the 13 *a priori* regions. Note that neurodegeneration is an inverted grey matter volume. General linear model results are corrected for multiple comparisons. For A+ ADRD, TSPO *N* = 32, amyloid *N* = 33, tau *N* = 31, neurodegeneration *N* = 31. For A− ADRD, TSPO *N* = 29, amyloid *N* = 29, tau *N* = 26, neurodegeneration *N* = 27. Bold indicates significance (*P* < 0.05).

For regional biomarker associations, greater TSPO was associated with greater tau in cingulate gyrus (0.8 [0.43, 1.2], *P* < 0.001), entorhinal cortex (0.49 [0.17, 0.81], *P* = 0.04), inferior parietal cortex (0.44 [0.24, 0.63], *P* < 0.001), lingual gyrus (0.42 [0.19, 0.65], *P* = 0.004), prefrontal cortex (0.42 [0.2, 0.64], *P* = 0.003), superior parietal cortex (0.42 [0.24, 0.6], *P* < 0.001) and middle inferior temporal gyrus (0.38 [0.14, 0.62], *P* = 0.03) and with greater neurodegeneration in the amygdala (−2.5 [−4, −1.1], *P* = 0.007) in amyloid-positive ADRD, but only associated with greater neurodegeneration in amygdala (−3 [−4.3, −1.7], *P* < 0.001) and hippocampus (−2.5 [−3.4, −1.5], *P* < 0.001) in amyloid-negative ADRD (Table 5). When accounting for specific pathways, TSPO mediated amyloid-associated tau in the superior (0.25 [0.07, 0.43], *P* = 0.05) and inferior (0.24 [0.06, 0.42], *P* = 0.05) parietal cortex as well as tau spreading across progressive Braak regions (early to middle: 0.12 [0.02, 0.21], *P* = 0.03; middle to late: 0.09 [0, 0.18], *P* = 0.05) in amyloid-positive ADRD (Table 6; Supplementary Table S3). Testing the alternative pathways, earlier-stage tau mediated associations between TSPO and later-stage tau (middle to late: 0.62 [0.39, 0.84], *P* < 0.001; early to middle: 0.51 [0.28, 0.74], *P* < 0.001; Supplementary Table S4). In amyloid-negative ADRD, TSPO mediated tau-associated neurodegeneration in the hippocampus (−0.46 [−0.71, −0.21], *P* = 0.004) and amygdala (−0.37 [−0.62, −0.13], *P* = 0.02; Table 6). Notably, the hippocampus was a unique region for TSPO, tau and neurodegeneration in the absence of amyloid positivity, reflected in the negative TSPO mediation between amyloid and tau (−0.51 [−0.82, −0.2], *P* = 0.02; Table 6) and colocalization of elevated TSPO with reduced amyloid in the hippocampus (Table 4), suggesting that TSPO mediated tau-associated neurodegeneration even in the absence of elevated amyloid.

**Table 5:** Regional biomarker associations by amyloid positivity

A+ ADRD	Amyloid	Tau	Grey matter volume
Prefrontal cortex	−0.13 [−1.3, 1], *P* > 0.99	**0.42 [0.2, 0.64], *P*** **=** **3e−03**	−0.02 [−0.05, 0.01], *P* = 0.92
Insula	−0.49 [−1.9, 0.9], *P* > 0.99	0.33 [−0.19, 0.86], *P* = 0.95	−0.15 [−0.4, 0.1], *P* = 0.97
Cingulate gyrus	−0.16 [−1.3, 0.96], *P* > 0.99	**0.8 [0.43, 1.2], *P*** **=** **3e−04**	−0.05 [−0.31, 0.2], *P* > 0.99
Fusiform gyrus	0.06 [−1.6, 1.7], *P* > 0.99	0.4 [0.09, 0.71], *P* = 0.13	−0.2 [−0.7, 0.29], *P* > 0.99
Lingual gyrus	−0.87 [−2.5, 0.77], *P* = 0.99	**0.42 [0.19, 0.65], *P*** **=** **4e−03**	−0.22 [−0.46, 0.02], *P* = 0.6
Entorhinal cortex	−1.2 [−3.9, 1.4], *P* > 0.99	**0.49 [0.17, 0.81], *P*** **=** **0.04**	−1 [−2.2, 0.07], *P* = 0.57
Middle inferior temporal gyrus	−0.08 [−1.5, 1.3], *P* > 0.99	**0.38 [0.14, 0.62], *P*** **=** **0.03**	−0.1 [−0.21, 0], *P* = 0.54
Superior temporal gyrus	−0.17 [−1.6, 1.3], *P* > 0.99	0.41 [0.09, 0.72], *P* = 0.15	−0.08 [−0.28, 0.12], *P* > 0.99
Inferior parietal cortex	−0.46 [−1.7, 0.79], *P* > 0.99	**0.44 [0.24, 0.63], *P*** **=** **1e−04**	−0.01 [−0.1, 0.09], *P* > 0.99
Superior parietal cortex	−0.46 [−1.7, 0.75], *P* > 0.99	**0.42 [0.24, 0.6], *P*** **=** **7e−05**	−0.02 [−0.12, 0.08], *P* > 0.99
Amygdala	1.8 [−0.68, 4.3], *P* = 0.88	0.4 [0.07, 0.74], *P* = 0.21	**−2.5 [−4, −1.1], *P*** **=** **7e−03**
Hippocampus	1.8 [−1.2, 4.8], *P* = 0.97	0.15 [−0.66, 0.96], *P* > 0.99	−0.91 [−1.7, −0.09], *P* = 0.31
Striatum	−0.22 [−1.9, 1.4], *P* > 0.99	−0.46 [−2.3, 1.4], *P* > 0.99	−0.16 [−0.48, 0.16], *P* = 0.99

A− ADRD	Amyloid	Tau	Grey matter volume
Prefrontal cortex	0.92 [−1.3, 3.2], *P* > 0.99	1 [−0.62, 2.6], *P* = 0.96	−0.02 [−0.06, 0.01], *P* = 0.86
Insula	0.18 [−2.5, 2.9], *P* > 0.99	0.89 [−1.7, 3.4], *P* > 0.99	−0.26 [−0.51, −0.01], *P* = 0.44
Cingulate gyrus	0.25 [−1.8, 2.3], *P* > 0.99	0.94 [−0.62, 2.5], *P* = 0.97	0.16 [−0.01, 0.34], *P* = 0.6
Fusiform gyrus	1.1 [−1.8, 4.1], *P* > 0.99	0.33 [−0.91, 1.6], *P* > 0.99	−0.63 [−1.1, −0.16], *P* = 0.11
Lingual gyrus	2.1 [−1.9, 6.1], *P* = 0.99	2 [0.1, 4], *P* = 0.4	−0.13 [−0.39, 0.13], *P* = 0.99
Entorhinal cortex	−3 [−7.3, 1.2], *P* = 0.9	0.52 [−0.18, 1.2], *P* = 0.86	−0.78 [−2.2, 0.65], *P* = 0.99
Middle inferior temporal gyrus	1.9 [−1.3, 5], *P* = 0.97	1.5 [−0.1, 3.2], *P* = 0.58	−0.11 [−0.22, 0.01], *P* = 0.57
Superior temporal gyrus	1.1 [−1.9, 4.1], *P* > 0.99	0.84 [−0.9, 2.6], *P* > 0.99	−0.15 [−0.36, 0.06], *P* = 0.9
Inferior parietal cortex	0.15 [−2.4, 2.7], *P* > 0.99	0.46 [−1.3, 2.2], *P* > 0.99	−0.05 [−0.15, 0.05], *P* = 0.99
Superior parietal cortex	0.93 [−1.4, 3.2], *P* > 0.99	1.1 [−0.61, 2.8], *P* = 0.95	−0.07 [−0.19, 0.05], *P* = 0.97
Amygdala	1.8 [−2.8, 6.4], *P* > 0.99	1.9 [0.24, 3.5], *P* = 0.27	**−3 [−4.3, −1.7], *P*** **=** **5e−05**
Hippocampus	−0.78 [−5.7, 4.1], *P* > 0.99	0.51 [−1.5, 2.5], *P* > 0.99	**−2.5 [−3.4, −1.5], *P*** **=** **9e−06**
Striatum	2.2 [−1.1, 5.5], *P* = 0.94	0.33 [−2.3, 3], *P* > 0.99	−0.2 [−0.49, 0.1], *P* = 0.93

Standardized estimates (beta) for biomarker regressions across cognitively unimpaired adults and amyloid-positive Alzheimer’s disease and related dementias (A+ ADRD) subgroups (top) and across cognitively unimpaired adults and amyloid-negative ADRD (A− ADRD) subgroups (bottom). Amyloid, tau and neurodegeneration across regions of interest are simultaneous predictors of TSPO. General linear model (A+ ADRD *N* = 30, A− ADRD *N* = 26) results are adjusted for multiple comparisons. Bold indicates significance (*P* < 0.05).

**Table 6:** Pathway mediations by amyloid positivity

A+ ADRD	Exposure->Mediator->Outcome	A− ADRD	Exposure->Mediator->Outcome
	Amyloid->TSPO->Tau		Amyloid->TSPO->Tau
Prefrontal cortex	0.15 [−0.01, 0.31], *P* = 0.16	Prefrontal cortex	−0.02 [−0.12, 0.09], *P* > 0.99
Insula	0.01 [−0.09, 0.11], *P* = 0.92	Insula	0 [−0.17, 0.17], *P* > 0.99
Cingulate gyrus	0.16 [0.01, 0.31], *P* = 0.1	Cingulate gyrus	0 [−0.04, 0.05], *P* > 0.99
Fusiform gyrus	0.15 [−0.04, 0.34], *P* = 0.21	Fusiform gyrus	−0.05 [−0.2, 0.09], *P* > 0.99
Lingual gyrus	0.12 [−0.07, 0.3], *P* = 0.35	Lingual gyrus	0.04 [−0.13, 0.22], *P* > 0.99
Entorhinal cortex	−0.08 [−0.34, 0.18], *P* = 0.68	Entorhinal cortex	−0.16 [−0.37, 0.04], *P* = 0.35
Middle inferior temporal gyrus	0.27 [0.02, 0.52], *P* = 0.1	Middle inferior temporal gyrus	0 [−0.2, 0.2], *P* > 0.99
Superior temporal gyrus	0.12 [−0.01, 0.26], *P* = 0.17	Superior temporal gyrus	0 [−0.1, 0.1], *P* > 0.99
Inferior parietal cortex	**0.24 [0.06, 0.42], *P*** **=** **0.05**	Inferior parietal cortex	−0.01 [−0.07, 0.05], *P* > 0.99
Superior parietal cortex	**0.25 [0.07, 0.43], *P*** **=** **0.05**	Superior parietal cortex	0.02 [−0.1, 0.14], *P* > 0.99
Amygdala	0.09 [−0.1, 0.27], *P* = 0.51	Amygdala	−0.31 [−0.56, −0.06], *P* = 0.11
Hippocampus	0.01 [−0.06, 0.08], *P* = 0.92	Hippocampus	**−0.51 [−0.82, −0.2], *P*** **=** **0.02**
Striatum	0 [−0.04, 0.04], *P* = 0.94	Striatum	0.2 [−0.03, 0.43], *P* = 0.35

	Tau->TSPO->Tau		Tau->TSPO->Tau
Early to middle Braak	**0.12 [0.02, 0.21], *P*** **=** **0.03**	Early to middle Braak	0.03 [−0.08, 0.14], *P* = 0.92
Middle to late Braak	**0.09 [0, 0.18], *P*** **=** **0.05**	Middle to late Braak	0 [−0.06, 0.06], *P* = 0.92

	Tau->TSPO->Grey matter volume		Tau->TSPO->Grey matter volume
Prefrontal cortex	−0.18 [−0.41, 0.06], *P* = 0.48	Prefrontal cortex	−0.08 [−0.2, 0.04], *P* = 0.44
Insula	−0.09 [−0.23, 0.04], *P* = 0.48	Insula	−0.13 [−0.29, 0.03], *P* = 0.29
Cingulate gyrus	−0.22 [−0.65, 0.2], *P* = 0.64	Cingulate gyrus	0.07 [−0.09, 0.22], *P* = 0.52
Fusiform gyrus	0.03 [−0.18, 0.24], *P* = 0.79	Fusiform gyrus	−0.17 [−0.36, 0.03], *P* = 0.29
Lingual gyrus	−0.28 [−0.64, 0.07], *P* = 0.48	Lingual gyrus	−0.04 [−0.2, 0.12], *P* = 0.71
Entorhinal cortex	−0.08 [−0.25, 0.1], *P* = 0.65	Entorhinal cortex	−0.01 [−0.18, 0.17], *P* = 0.94
Middle inferior temporal gyrus	−0.41 [−0.75, −0.07], *P* = 0.25	Middle inferior temporal gyrus	−0.25 [−0.46, −0.04], *P* = 0.09
Superior temporal gyrus	−0.03 [−0.25, 0.18], *P* = 0.79	Superior temporal gyrus	−0.06 [−0.17, 0.05], *P* = 0.49
Inferior parietal cortex	0.11 [−0.21, 0.44], *P* = 0.65	Inferior parietal cortex	−0.04 [−0.13, 0.05], *P* = 0.52
Superior parietal cortex	−0.13 [−0.44, 0.18], *P* = 0.65	Superior parietal cortex	−0.06 [−0.17, 0.05], *P* = 0.49
Amygdala	−0.11 [−0.25, 0.03], *P* = 0.48	Amygdala	**−0.37 [−0.62, −0.13], *P*** **=** **0.02**
Hippocampus	−0.02 [−0.07, 0.03], *P* = 0.65	Hippocampus	**−0.46 [−0.71, −0.21], *P*** **=** **4e−03**
Striatum	0.01 [−0.03, 0.05], *P* = 0.74	Striatum	−0.04 [−0.13, 0.06], *P* = 0.56

Standardized estimates (beta) in amyloid-positive Alzheimer’s disease and related dementias (A+ ADRD; top) and amyloid-negative Alzheimer’s disease and related dementias (A− ADRD; bottom) with TSPO as the mediator. Mediation model (A+ ADRD *N* = 30, A− ADRD *N* = 26) results are corrected for multiple comparisons. Bold indicates significance (*P* < 0.05).

### Sensitivity analysis: data transformations

After transformation, elevated TSPO was now colocalized with elevated tau in 11 regions total and with elevated neurodegeneration in 5 regions total in all participants (Supplementary Table S5). In amyloid-positive ADRD, elevated TSPO was colocalized with elevated neurodegeneration in seven regions total, while in amyloid-negative ADRD, elevated TSPO was colocalized with elevated neurodegeneration in seven regions total and elevated tau in five regions total (Supplementary Table S5). TSPO-tau associations in the superior parietal cortex and TSPO-neurodegeneration association in the entorhinal cortex and middle inferior temporal cortex did not remain significant after transformation in all participants (Supplementary Table S6). TSPO-tau associations in the entorhinal cortex and middle inferior temporal gyrus did not remain significant in amyloid-positive ADRD (Supplementary Table S6). All mediation results were unchanged after transformation (Supplementary Tables S7 and S8).

### Sensitivity analysis: univariate associations

After assessing TSPO associations with amyloid, tau or neurodegeneration in separate, univariate models (Supplementary Table S9), greater TSPO was additionally associated with greater tau in the hippocampus, amygdala, entorhinal cortex, middle inferior temporal cortex, fusiform gyrus and prefrontal cortex, as well as greater neurodegeneration in the lingual gyrus, insula, superior temporal gyrus, superior parietal cortex, inferior parietal cortex and prefrontal cortex in all participants. Furthermore, greater TSPO was associated with lower amyloid in the hippocampus. In amyloid-positive ADRD, greater TSPO was additionally associated with greater tau in the amygdala, fusiform gyrus and superior temporal gyrus, as well as greater neurodegeneration in the entorhinal cortex, hippocampus, fusiform gyrus, lingual gyrus, middle inferior temporal gyrus, superior parietal cortex and inferior parietal cortex. In amyloid-negative ADRD, greater TSPO was additionally associated with greater neurodegeneration in the entorhinal cortex and the fusiform gyrus. Furthermore, greater TSPO was associated with lower amyloid as well as greater tau in the hippocampus and the amygdala.

### Sensitivity analysis: brain-wide investigation

After investigating regions across the brain (Supplementary Fig. S2), similar patterns of elevated TSPO, tau and amyloid were observed with additional involvement of occipital cortex, sensorimotor cortex and medial structures; however, using individual subregions for neurodegeneration attenuated the difference in prefrontal, temporal and limbic regions, but greater neurodegeneration persisted in parietal regions. Associations of greater TSPO with greater tau extended to sensorimotor and medial structures, while associations of greater TSPO with greater neurodegeneration were only present in subregions of the prefrontal, temporal and parietal cortex, and additionally in the occipital cortex. Greater TSPO was associated with lower amyloid, which was additionally observed in the occipital cortex. TSPO-mediated tau-associated neurodegeneration was most pronounced in the inferior and middle temporal gyrus, amygdala, hippocampus and subregions of the prefrontal cortex (unadjusted *P* < 0.05). In amyloid-positive ADRD, there were widespread areas of greater TSPO, tau and amyloid with limited posterior areas of greater neurodegeneration, while in amyloid-negative ADRD, there were widespread areas of greater TSPO and limited limbic areas of greater tau but lower amyloid, and attenuated differences in neurodegeneration. Greater TSPO was associated with greater tau in more regions than with greater neurodegeneration in amyloid-positive ADRD, while associations between TSPO and neurodegeneration in individual subregions were attenuated, but present in amyloid-negative ADRD with no TSPO and tau associations. TSPO-mediated tau-associated neurodegeneration was present in the amygdala in amyloid-positive ADRD and amyloid-negative ADRD, as well as a few additional subregions in amyloid-positive ADRD (unadjusted *P* < 0.05). Overall, brain-wide associations were convergent with results using the 13 *a priori* regions, with additional regional involvement and with some relationships being focal within composite regions.

## Discussion

Inflammatory alterations, measured with TSPO PET, were colocalized with tau to a greater spatial extent than amyloid and neurodegeneration, were associated with the magnitude of neurodegeneration and tau but not amyloid, and mediated tau-associated neurodegeneration across different clinical variants of ADRD, suggesting a common TSPO-tau mechanism in neurodegenerative disease. Typical Alzheimer’s disease is conceptualized as an amyloid-induced tauopathy, and in the presence of elevated amyloid, TSPO further mediates amyloid-associated tau and tau spreading. Even in amyloid-negative individuals with ADRD, elevated TSPO was widespread and mediated tau-associated neurodegeneration in limbic regions. Therapeutic intervention strategies should consider microglia-associated inflammatory alterations as an alternative or additional target to amyloid to delay or prevent tau accumulation, tau spreading and/or tau-related neurodegeneration.^33-35^

While we observed associations among TSPO, tau and neurodegeneration, specifically that TSPO mediated tau-associated neurodegeneration, the mechanisms remain unclear. Interestingly, we observed hippocampal specificity, particularly in the amyloid-negative context, that persisted when investigating brain-wide mediations. In 5XFAD mice, autoradiography demonstrated an early TSPO elevation in the subiculum of the hippocampus that was greatest in microglia that were in contact with amyloid plaques.^36^ Similar results were observed in autosomal dominant AD brains with PSEN1 mutations.^36^ Immunofluorescence staining suggested that this TSPO-enriched microglia subtype promoted the progression of pathology, neurodegeneration and cognitive decline.^36^ Interestingly, tauopathy mice with the Christchurch mutation (R136S) had lower tau burden and were protected against tau-associated neurodegeneration in the hippocampus through reduced interferon response to tau in mouse and human microglia.^37^ In cognitively unimpaired older adults, hippocampal synaptic density was particularly vulnerable to early tau accumulation.^38^ Even in a mouse model of tauopathy without amyloid, there were unique immune hubs in which depletion of microglia or cytotoxic T cells prevented tau-related neurodegeneration.^39^ Larger studies that allow for subregional analyses within individual ADRD diagnoses and longitudinal studies, including functional measures of microglia and other cell types, will lead to more mechanistic insight in humans.

As a simplistic descriptor on the individual level, we chose to categorize individuals as TSPO-positive if they were above the mean of amyloid-negative cognitively unimpaired adults in a composite region; TSPO positivity was observed in 11 out of 13 amyloid-positive, tau-positive individuals with ADRD, 2 out of 2 amyloid-negative, tau-positive individuals with ADRD and 5 out of 5 amyloid-negative, tau-negative individuals with ADRD. This indicated ‘high’ TSPO in individuals with ADRD, regardless of their amyloid or tau positivity. As one potential explanation, amyloid-negative, tau-negative, TSPO-positive individuals with ADRD could have unmeasured pathology that aligns with their clinical diagnosis (e.g. TDP-43 in LATE) or systemic risk factors that account for elevated TSPO without AD pathology. In a complementary approach, by investigating regional biomarker elevations on the group level, we observed 11 out of 13 regions with elevated TSPO compared to amyloid-negative cognitively unimpaired adults. Interestingly, the hippocampus was the only region to have elevated TSPO alone. There could be discordance between TSPO and AD pathology if TSPO precedes and promotes AD pathology.^9,10^ By investigating regional biomarker associations and mediations, we observed that not all biomarkers that were colocalized together were directly associated with each other, but may have pathway-dependent associations. Studies have demonstrated that inflammatory alterations can precede AD pathology in autosomal dominant AD,^40^ AD in adults with Down syndrome^41^ and late-onset AD.^42^ Longitudinal TSPO PET data capturing conversion to amyloid and/or tau PET positivity is needed.

Multivariate models demonstrated regional differences where TSPO was more strongly associated with tau or neurodegeneration. In univariate models, TSPO-tau and TSPO-neurodegeneration associations were observed in additional regions, many of which overlapped. The stronger of the two biomarker associations in the univariate models was the one that was attenuated, but still significant in the multivariate models, indicating that tau and neurodegeneration may be in the same pathway, but did not fully account for the same variance in TSPO. Further univariate associations were revealed such that greater TSPO was associated with lower amyloid. These unexpected associations were also reflected in specific paths of the mediation analyses. Therefore, complex mediation results were robust to more simplistic model specifications.

Through pathway analyses, we found that TSPO mediated amyloid-associated tau, but only in the amyloid-positive ADRD subgroup. Previous work suggests the link between amyloid and early Braak tau may be an astrocyte-dependent pathway,^43^ although astrocytes may be captured to some extent with TSPO PET and cross-talk between inflammatory cell types is being more widely appreciated. The regional specificity to the middle inferior temporal gyrus and the parietal cortex here could be driven by the two largest diagnostic subgroups, AD and PCA. When assessing the observations with the highest skew, they were in regions including the middle inferior temporal gyrus and prefrontal cortex for amyloid-positive individuals with AD and superior and inferior parietal cortex for amyloid-positive individuals with PCA, as expected for these diagnostic groups.^44^ By transforming the data rather than excluding statistical outliers, many results remained unchanged or minimally changed, but TSPO-tau and TSPO-neurodegeneration associations in the entorhinal cortex and middle inferior temporal gyrus were attenuated, and TSPO-tau associations were further attenuated in the superior parietal cortex.

For tau-spreading pathways, we restricted the analyses to progressive Braak stage regions. We found that TSPO-mediated tau spreading across Braak regions only in the amyloid-positive ADRD subgroup, aligning with previous work in MCI/AD.^9,10^ We further observed the alternative direction in cross-sectional mediations, suggesting a positive feedback loop in which TSPO could promote (TSPO->earlier tau->later tau) and maintain (earlier tau->TSPO->later tau) tau spreading mechanisms. This is potentially supported by microglia depletion studies that prevent tau spreading via endosome-related pathways.^45^ Tau spreading may follow a different spatial and temporal pattern in the amyloid-negative context.^46^

For tau-associated neurodegeneration pathways, TSPO mediations were spatially limited to the amygdala, middle inferior temporal gyrus, superior parietal cortex and hippocampus in the overall group and in the hippocampus and amygdala for the amyloid-negative ADRD subgroup. When investigating brain-wide mediations, TSPO mediations were also present in the amygdala for the amyloid-positive ADRD subgroup without correction for multiple comparisons. Overall, TSPO-mediated amyloid-associated tau and TSPO-mediated tau spreading may depend on elevated amyloid, whereas TSPO-mediated tau-associated neurodegeneration may not.

These findings have clinical implications in the era of anti-amyloid treatment strategies. Interestingly, microglia-mediated clearance mechanisms can be leveraged with anti-amyloid immunotherapy,^47^ opening the possibility of treatment strategies that selectively activate protective microglia functions.^33,48^ Anti-amyloid treatments reduce the amyloid plaque burden but may not necessarily reduce the tau neurofibrillary tangle burden.^49,50^ Therefore, the normalization of amyloid (i.e. treatment-related amyloid clearance [TRAC]^51^) may not prevent TSPO-mediated tau-associated neurodegeneration, as this pathway was present even in the amyloid-negative context. Resolving chronic pro-inflammatory microglia states and/or removing non-AD tauopathy and other pathology may be needed in addition to TRAC to slow or prevent downstream neurodegeneration and cognitive decline. There is a phenomenon known as ‘pseudo-atrophy’ that occurs with anti-amyloid immunotherapy, thought to be related to amyloid plaque clearance from the parenchyma.^52^ TSPO PET has the potential to provide critical insight into whether this reduction in grey matter volume is simply a resolution of inflammatory alterations after amyloid plaque clearance or actual atrophy due to the remaining neurofibrillary tangles.

Limitations of the study include cross-sectional analysis, the mix of ADRD diagnoses (MCI, AD, PCA, lvPPA, LATE, FTD), relatively small sample size, limited regional definitions and lack of other measures of ADRD pathology and inflammation. While this is a cross-sectional analysis, we assessed mediations in the alternative direction. Ongoing longitudinal data collection will allow us to additionally use temporality to infer causality. Non-Alzheimer’s pathology, including cerebrovascular disease, TDP43 and α-synuclein, which we were not able to measure, could be contributing to the TSPO PET signal, particularly across this range of clinical diagnoses. TSPO PET, which we broadly interpret as inflammatory alterations, may provide information that is useful in differential diagnosis through its magnitude of change and spatial distribution, similar to FDG PET for ADRD. We chose *a priori* regions of interest that covered key regions across these diagnoses; however, future studies with sufficient sample sizes for each diagnostic group should further investigate brain-wide associations. Larger studies with broad biomarker characterization, including markers of secreted amyloid, phospho-tau and microglia function, are needed to provide mechanistic insight into these findings. Still, the elevated density/recruitment of microglia in AD-related brain regions, particularly in limbic regions, across different brain microenvironments provides valuable supporting information for future mechanistic investigations.

In conclusion, inflammatory alterations, measured with TSPO PET using a simplified acquisition and quantification scheme, were elevated in a spatial pattern following tau, associated with tau burden accounting for amyloid and neurodegeneration, and mediated tau-associated neurodegeneration across various ADRD diagnoses. Inflammatory alterations may additionally be involved in the link between amyloid and tau, as well as tau spreading, given the presence of elevated amyloid. Future work should focus on understanding the cellular and molecular components of these inflammatory alterations, particularly in the hippocampus, towards a unified framework incorporating the pathogenic and immunogenic pathways in ADRD.

## Supplementary Material

fcag242_Supplementary_Data

## Data Availability

All data produced in the present study are available upon reasonable request to the authors.
