# Supplementary material for: Inflammatory alterations mediate tau-associated neurodegeneration
Source: Brain Commun. 2026 Jun 24;8(4):fcag242. doi: 10.1093/braincomms/fcag242 (PMC13332402; doi:10.1093/braincomms/fcag242)
Supplement: fcag242_Supplementary_Data [file fcag242_supplementary_data.docx]

**Supplemental Materials**

Cognitive domain scores were averaged across individual neuropsychological tests, which were z-scored against age, sex, and education. Delayed Episodic Memory included Selective Reminding Test (Delayed), Craft Story Telling Test (Delayed), Benson Complex Figure Test (Delayed). Immediate Episodic Memory included Selective Reminding Test (Total), Craft Story Telling Test (Immediate). Attention/Processing Speed included Trail Making Test (Part A), Digit Span Test Forward (Total). Executive Function included Trail Making Test (Part B), Digit Span Test Backward (Total). Language included Categorical Fluency (Total), Animal Fluency (Total), Letter Fluency (Total), Multilingual Naming Test (Total). Visuospatial ability included Benson Complex Figure Test (Immediate).

Neuroimaging regions of interest were defined from specific brain atlases, based on availability. All regions of interest were averaged bilaterally. Prefrontal Cortex included Hammers atlas: Frontal Gyrus (Superior, Middle, Inferior), Straight Gyrus, Orbitofrontal Cortex (Anterior, Posterior, Medial, Lateral). Insula included Hammers atlas: Insula. Cingulate Gyrus included Hammers atlas: Cingulate Gyrus. Fusiform Gyrus included Hammers atlas: Fusiform Gyrus. Lingual Gyrus included Hammers atlas: Lingual Gyrus. Entorhinal Cortex included Desikan-Killiany atlas: Entorhinal Cortex. Middle Inferior Temporal Gyrus included Hammers atlas: Temporal Lobe (Medial Anterior, Inferior), Temporal Gyrus (Middle, Inferior). Superior Temporal Gyrus included Hammers atlas: Superior Temporal Gyrus (Anterior, Posterior). Inferior Parietal Cortex included Hammers atlas: Inferior Parietal Gyrus. Superior Parietal Cortex included Hammers atlas: Superior Parietal Gyrus. Amygdala included Hammers atlas: Amygdala. Hippocampus included Hammers atlas: Hippocampus. Striatum included Hammers atlas: Caudate Nucleus, Putamen. Braak I/II included Hippocampus, Hammers atlas: Parahippocampal Gyrus. Braak III/IV included Middle Inferior Temporal Gyrus. Braak V/VI included Prefrontal Cortex, Superior Temporal Gyrus, Inferior Parietal Cortex, Superior Parietal Cortex. Composite (for TSPO and FBB) included Prefrontal Cortex, Cingulate Gyrus, Middle Inferior Temporal Gyrus, Superior Temporal Gyrus, Inferior Parietal Cortex, Superior Parietal Cortex


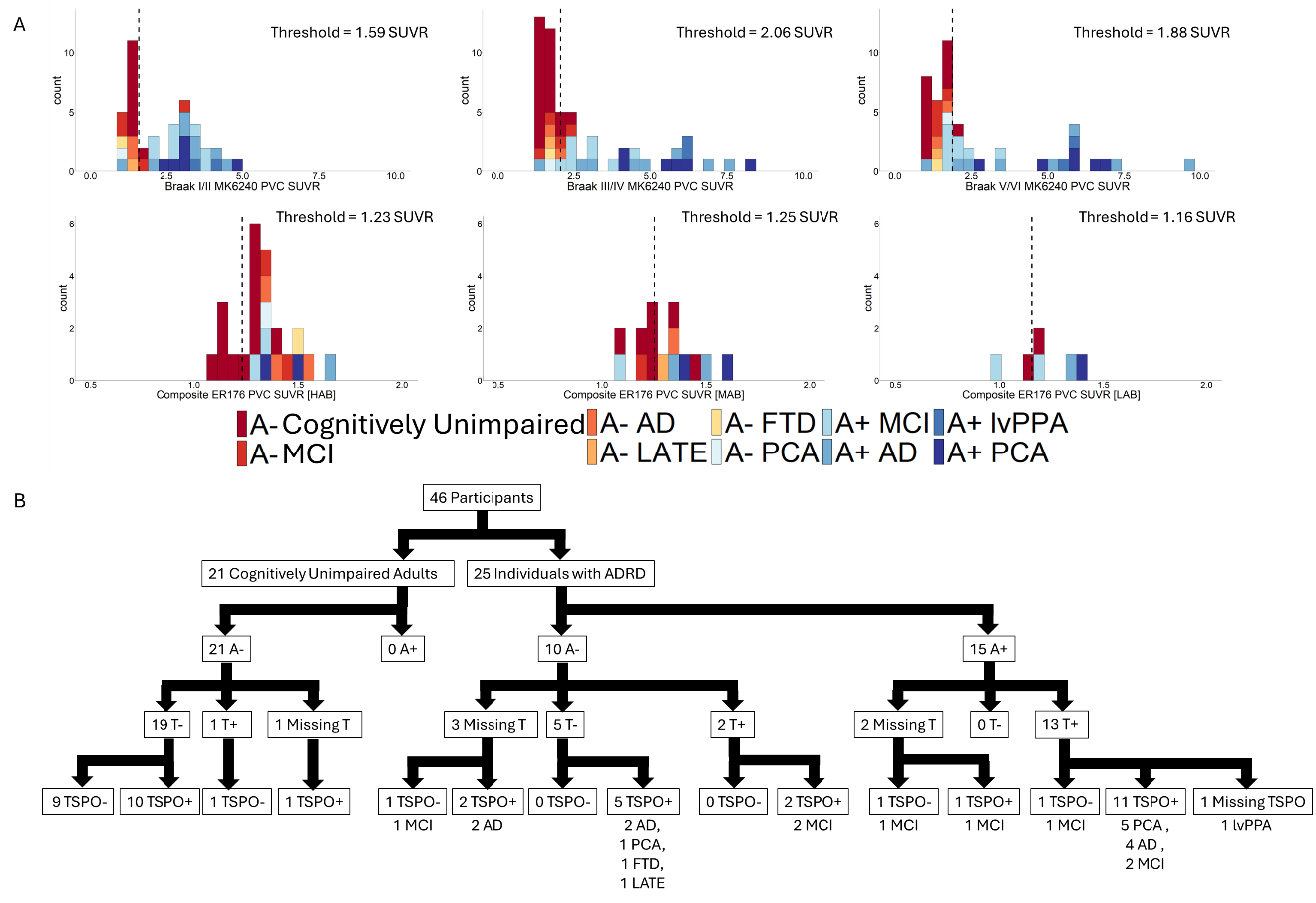


Supplemental Figure 1. A) Histogram of MK6240 partial volume corrected (PVC) standard uptake value ratio (SUVR) across Braak I/II, Braak III/IV, and Braak V/VI (top row; N=40) and ER176 PVC SUVR in a composite brain region of interest across high, mixed, and low affinity binders separately (bottom row; high N=25; mixed N=15; low N=6). The dashed black line indicates mean + 2 standard deviations in cognitively unimpaired adults for tau and mean in cognitively unimpaired adults for TSPO. B) Flow chart of participants by biomarker positivity with Alzheimer’s disease and related dementia (ADRD) diagnosis. Participants included cognitively unimpaired adults (N=21), amnestic or non-amnestic mild cognitive impairment (MCI; N=8), amnestic multi-domain dementia syndrome referred to as “AD dementia” (N=8), PCA (N=6), logopenic variant of primary progressive aphasia (lvPPA; N=1), limbic-predominant age-related TDP43 encephalopathy (LATE; N=1), and frontotemporal dementia (FTD; N=1).


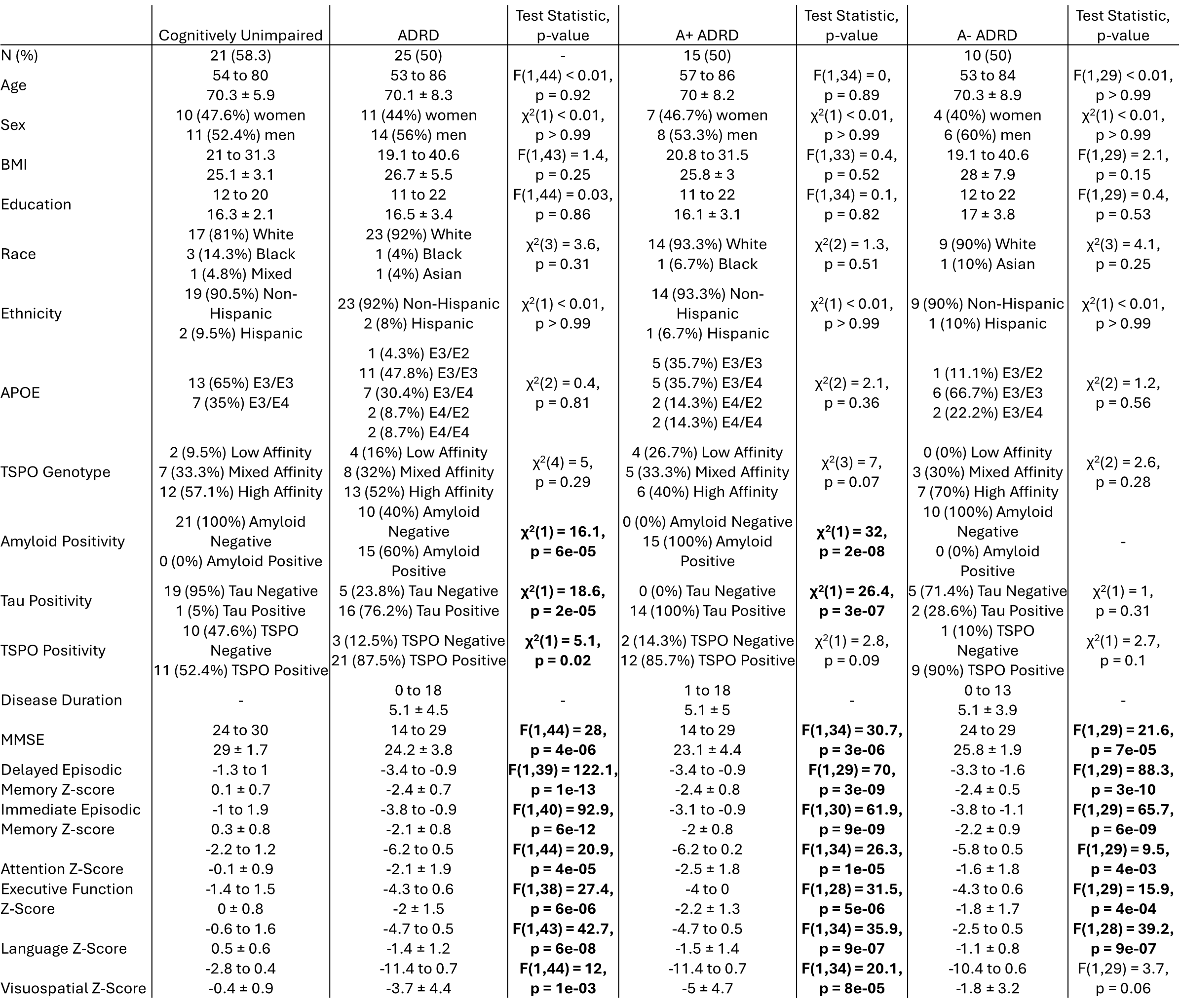


Supplemental Table 1. Demographic characteristics, biomarker positivity, and cognitive scores for cognitively unimpaired adults, all adults with Alzheimer’s disease and related dementia (ADRD), amyloid positive ADRD (A+ ADRD), and amyloid negative ADRD (A- ADRD). Test statistics and p-values are in comparison to cognitively unimpaired adults. Bold indicates significance (p<0.05).


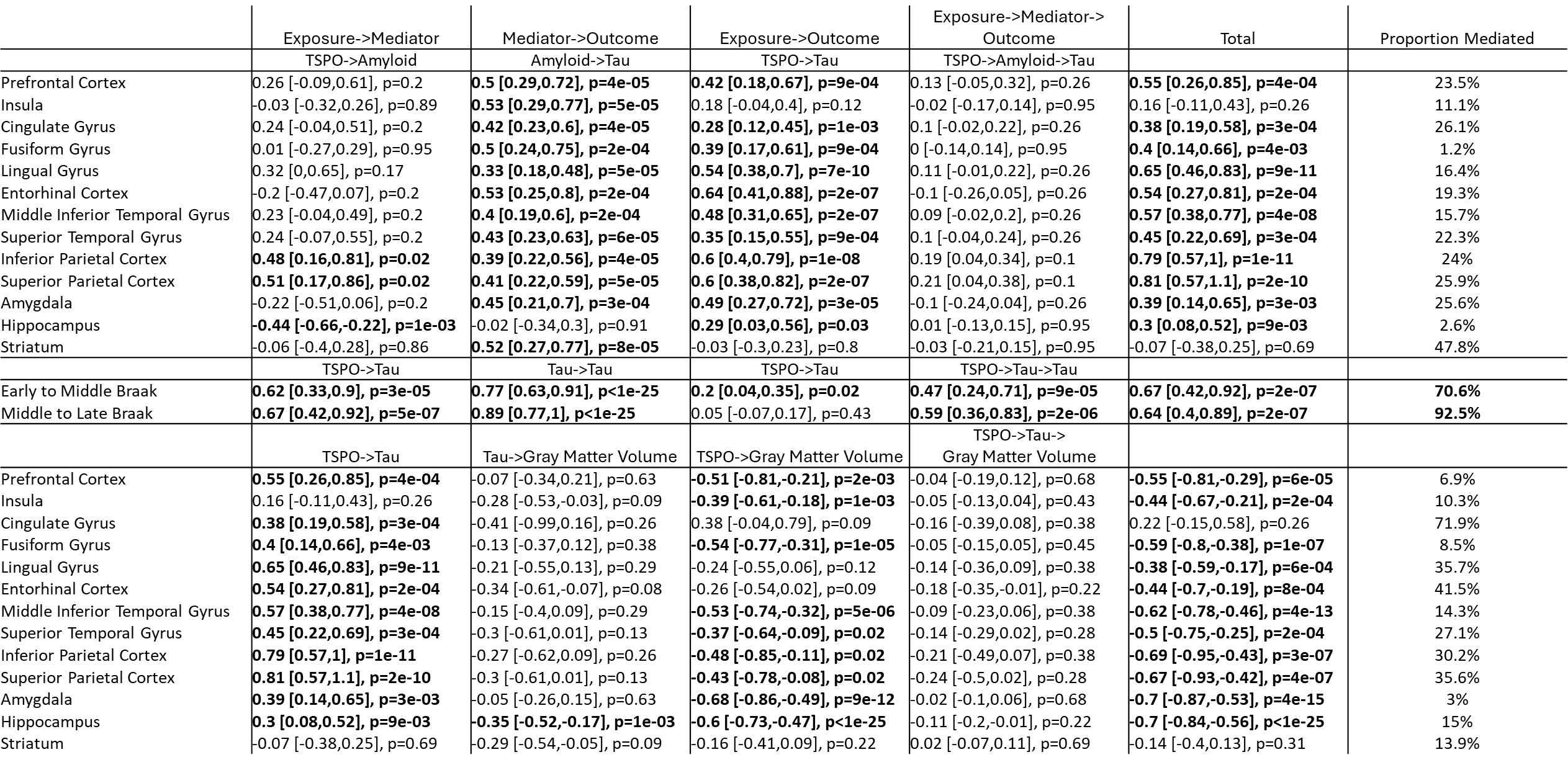


Supplemental Table 2. Standardized pathway estimates (beta) in all participants with Alzheimer’s disease and related dementia (ADRD) with TSPO as the initiator. Mediation model (N=37) results are corrected for multiple comparisons. Note: proportion mediated may exceed 100% in some regions indicating unstable estimates or inconsistent mediation in which the direct and indirect effects have opposite signs. Bold indicates significance (p<0.05).


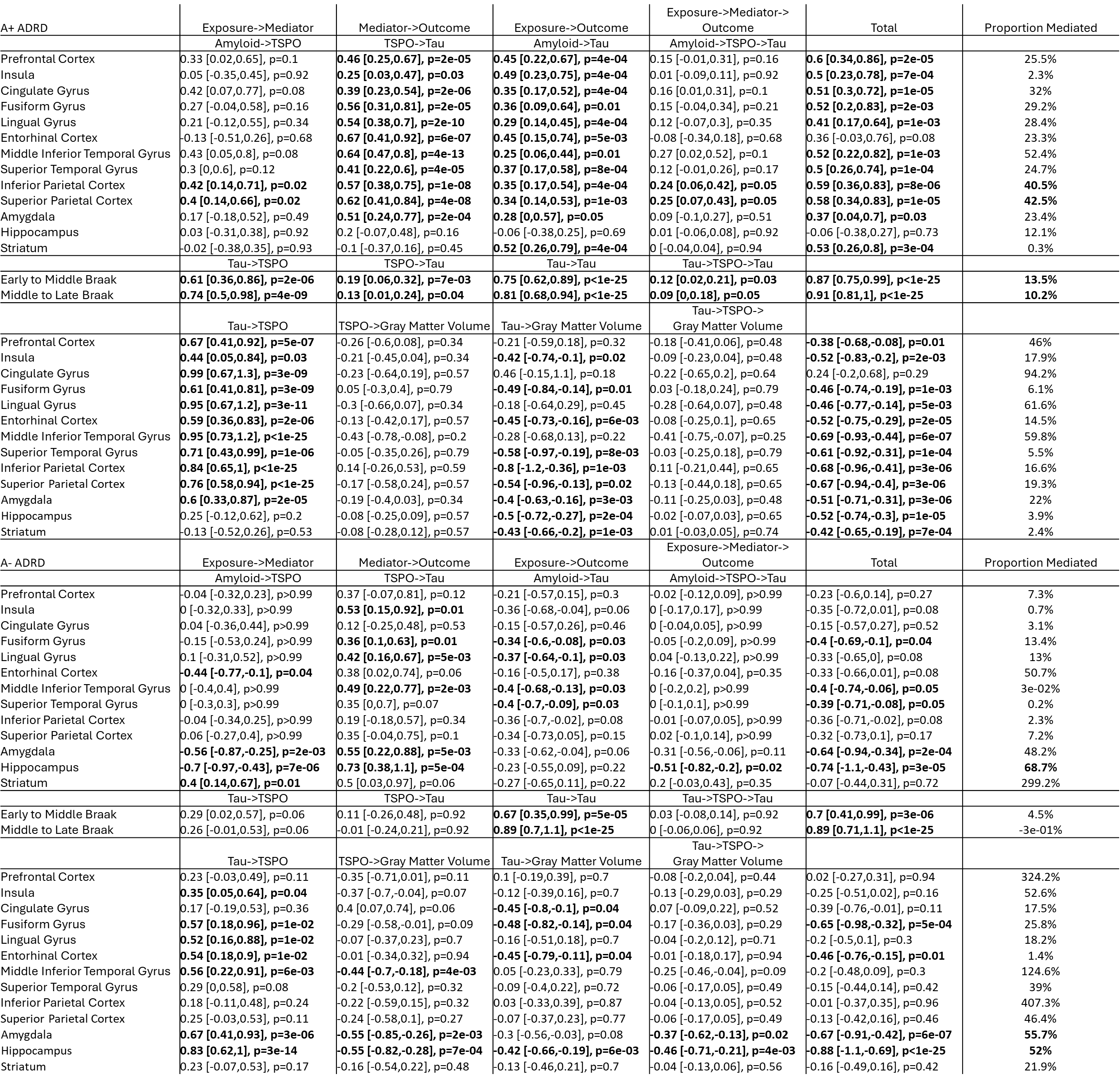


Supplemental Table 3. Standardized estimates (beta) in amyloid-positive Alzheimer’s disease and related dementia (A+ ADRD; top) and amyloid-negative Alzheimer’s disease and related dementia (A- ADRD; bottom) with TSPO as the mediator. Mediation model (A+ ADRD N = 30, A- ADRD N = 26) results are corrected for multiple comparisons. Note: proportion mediated may exceed 100% in some regions indicating unstable estimates or inconsistent mediation in which the direct and indirect effects have opposite signs. Bold indicates significance (p<0.05).


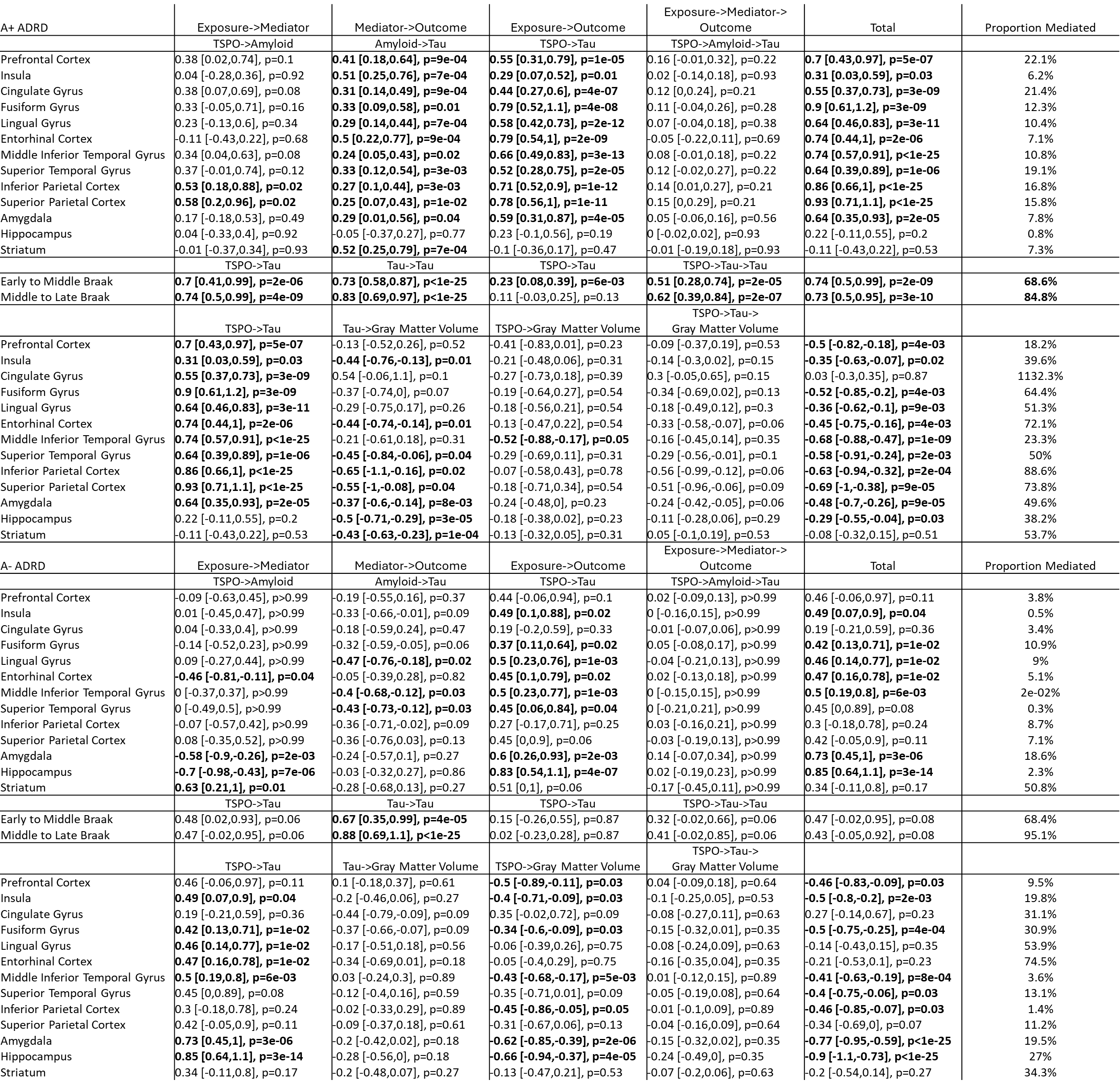


Supplemental Table 4. Standardized pathway estimates (beta) in amyloid positive Alzheimer’s disease and related dementia (A+ ADRD; top) and amyloid negative Alzheimer’s disease and related dementia (A- ADRD; bottom) with TSPO as the initiator. Mediation model (A+ ADRD N = 30, A- ADRD N = 26) results are corrected for multiple comparisons. Note: proportion mediated may exceed 100% in some regions indicating unstable estimates or inconsistent mediation in which the direct and indirect effects have opposite signs. Bold indicates significance (p<0.05).


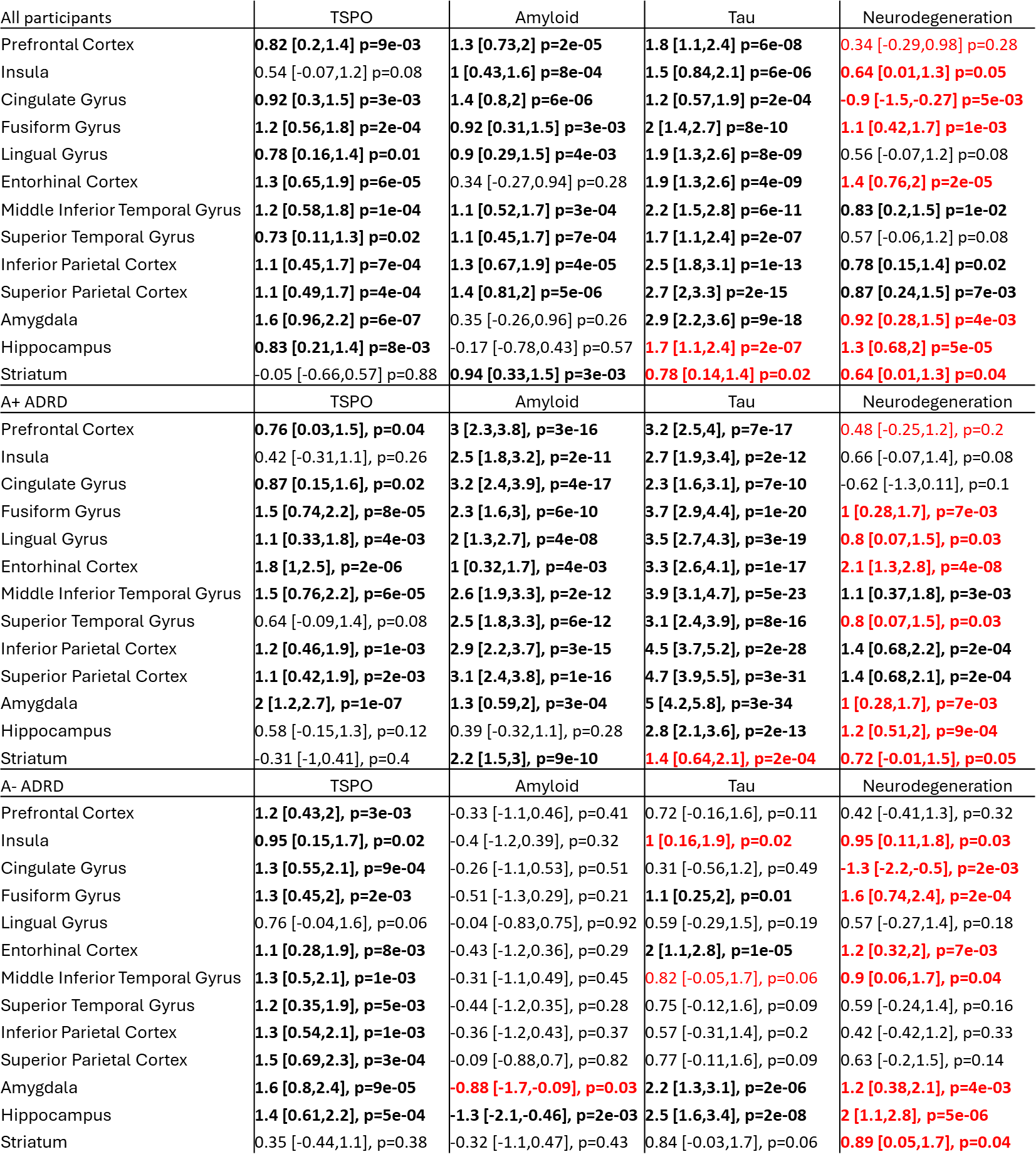


Supplemental Table 5. Standardized effect size (beta) for the difference between all participants with Alzheimer’s disease and related dementia and cognitively unimpaired adult subgroups (top), amyloid-positive Alzheimer’s disease and related dementia (A+ ADRD) and cognitively unimpaired adult subgroups (middle) and between amyloid-negative ADRD (A- ADRD) and cognitively unimpaired adult subgroups (bottom) across each of the 13 a priori regions. Note that Neurodegeneration is inverted gray matter volume for direct comparison to other biomarkers. Generalized linear models were fit with a Gamma distribution and a log link. Results are adjusted for multiple comparisons. Bold indicates significance (p<0.05); red indicates a change in significance from general linear models. For example, bold red indicates that a difference became significant after transformation; unbold red indicates that a difference lost significance after transformation. For all participants, TSPO N = 41, Amyloid N = 42, Tau N = 38, Neurodegeneration N = 39. For A+ ADRD, TSPO N = 32, Amyloid N = 33, Tau N = 31, Neurodegeneration N = 31. For A- ADRD, TSPO N = 29, Amyloid N = 29, Tau N = 26, Neurodegeneration N = 27.


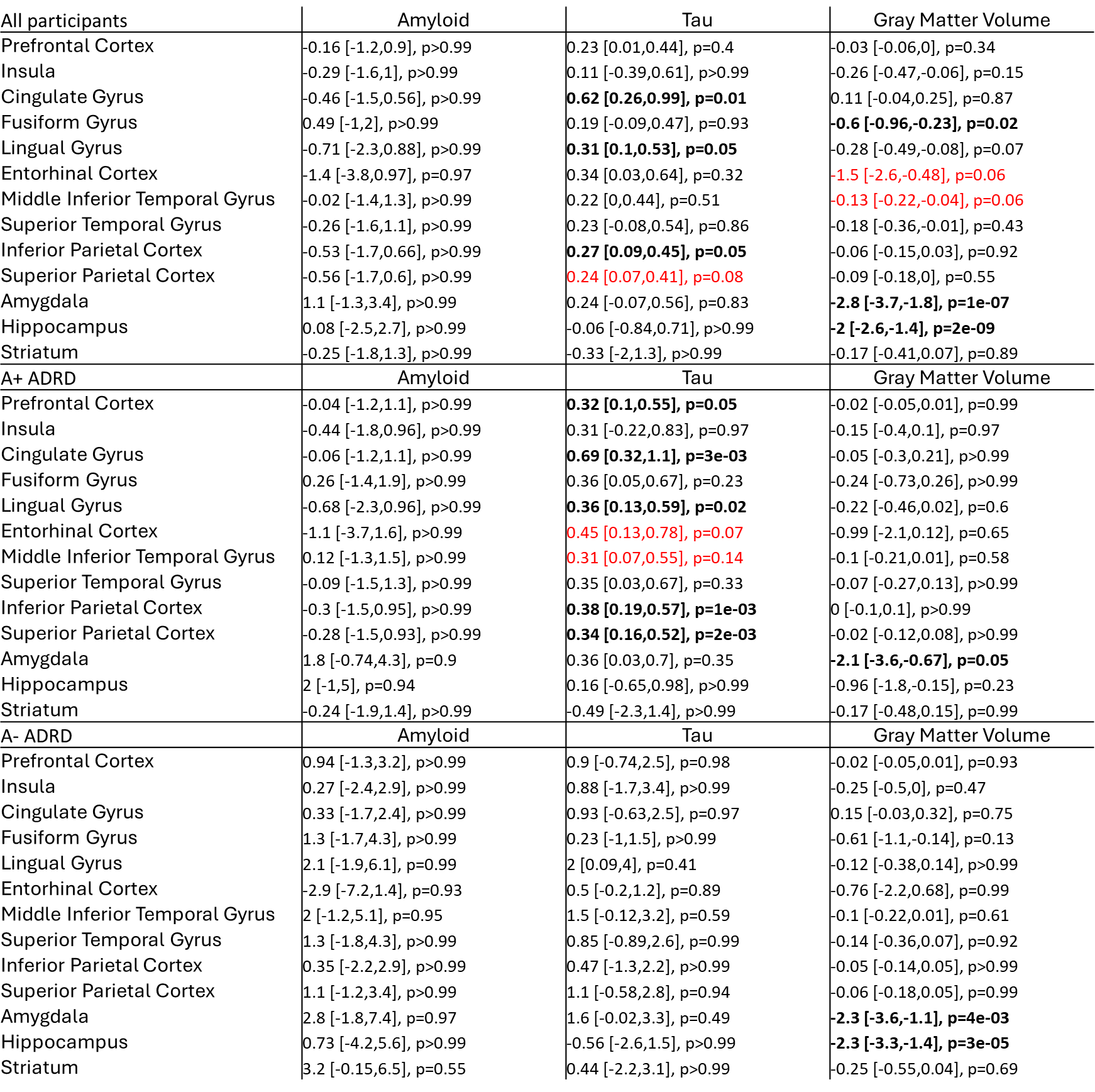


Supplemental Table 6. Standardized estimates (beta) for biomarker regressions across all participants with Alzheimer’s disease and related dementia and cognitively unimpaired adult subgroups (top), amyloid-positive Alzheimer’s disease and related dementia (A+ ADRD) and cognitively unimpaired adult subgroups (middle) and between amyloid-negative ADRD (A- ADRD) and cognitively unimpaired adult subgroups (bottom). Models per region of interest include amyloid, tau, and neurodegeneration as simultaneous predictors of TSPO. Generalized linear models were fit with a Gamma distribution and a log link (all participants N = 37, A+ ADRD N = 30, A- ADRD N = 26), and results are adjusted for multiple comparisons. Bold indicates significance (p<0.05); red indicates a change in significance from general linear models. For example, bold red indicates that a difference became significant after transformation; unbold red indicates that a difference lost significance after transformation.


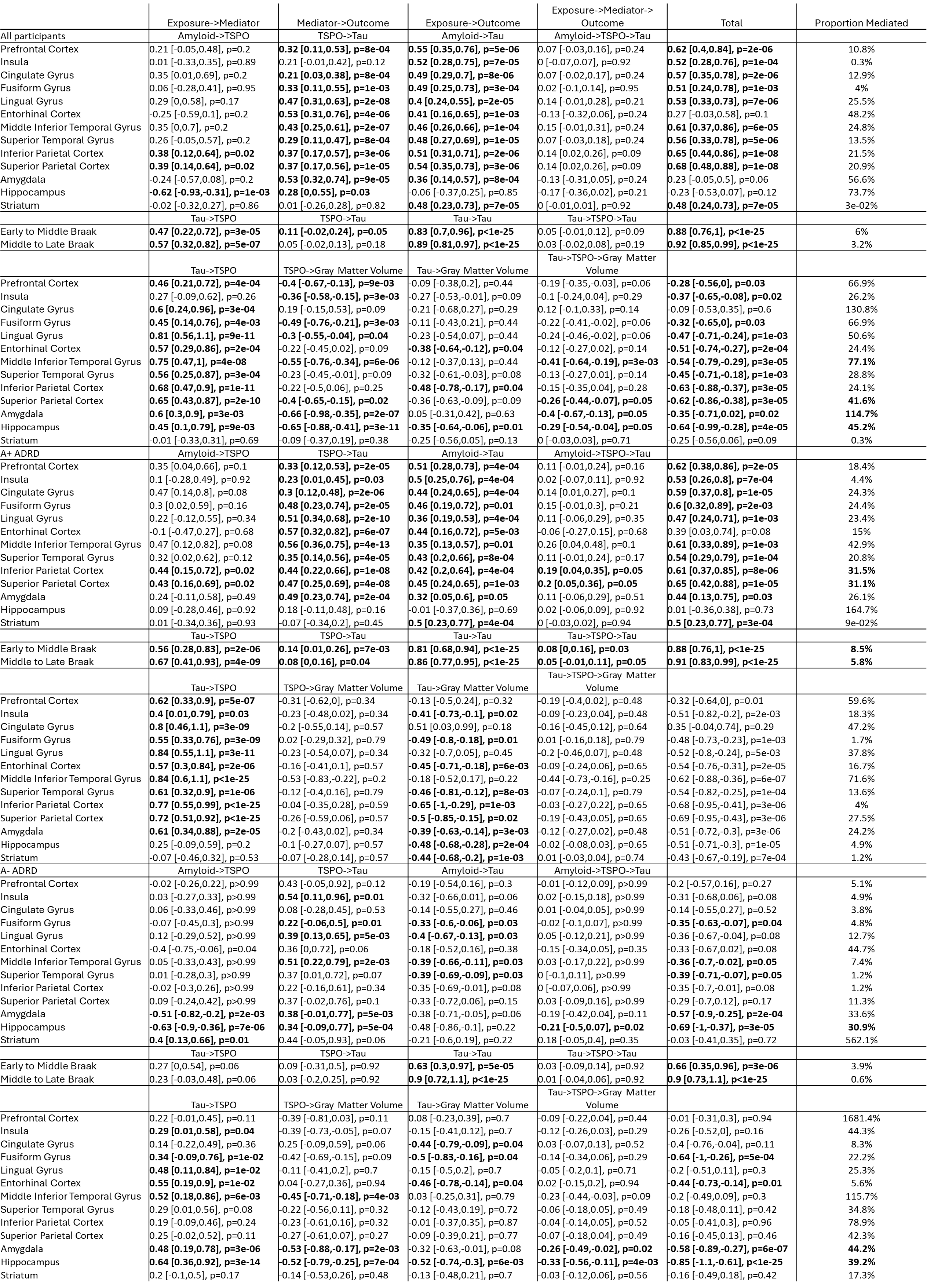


Supplemental Table 7. Standardized estimates (beta) in all participants with Alzheimer’s disease and related dementia and cognitively unimpaired adult subgroups (top), amyloid-positive Alzheimer’s disease and related dementia (A+ ADRD) and cognitively unimpaired adult subgroups (middle) and between amyloid-negative ADRD (A- ADRD) and cognitively unimpaired adult subgroups (bottom) with TSPO as the mediator. Biomarkers were log-transformed. Mediation model (all participants N = 37, A+ ADRD N = 30, A- ADRD N = 26) results are corrected for multiple comparisons. Bold indicates significance (p<0.05); red indicates a change in significance from general linear models. For example, bold red indicates that a difference became significant after transformation; unbold red indicates that a difference lost significance after transformation. Note: proportion mediated may exceed 100% in some regions indicating unstable estimates or inconsistent mediation in which the direct and indirect effects have opposite signs.


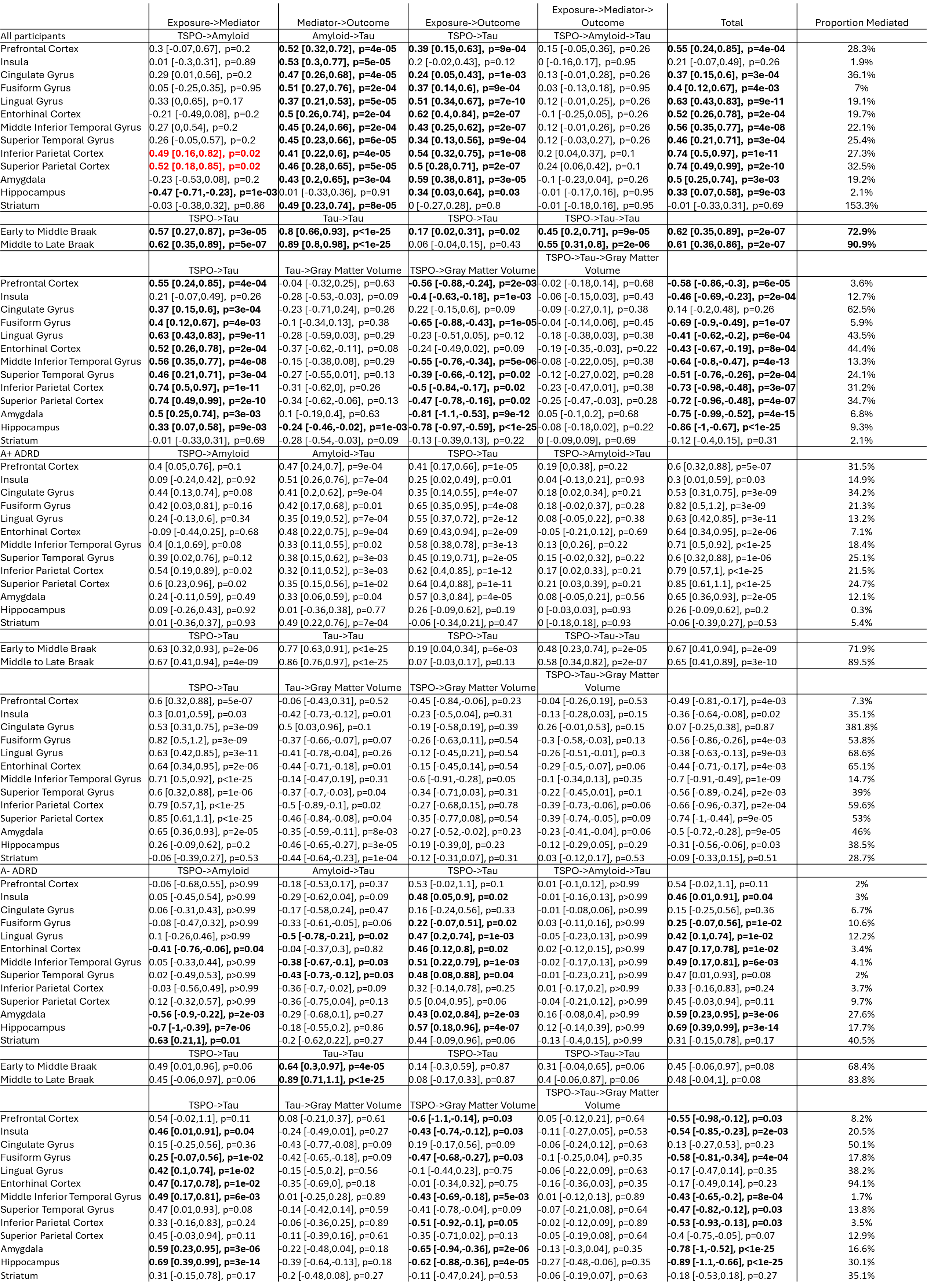


Supplemental Table 8. Standardized pathway estimates (beta) in all participants with Alzheimer’s disease and related dementia and cognitively unimpaired adult subgroups (top), amyloid-positive Alzheimer’s disease and related dementia (A+ ADRD) and cognitively unimpaired adult subgroups (middle) and between amyloid-negative ADRD (A- ADRD) and cognitively unimpaired adult subgroups (bottom) with TSPO as the initiator. Biomarkers were log-transformed. Mediation model (all participants N = 37, A+ ADRD N = 30, A- ADRD N = 26) results are corrected for multiple comparisons. Bold indicates significance (p<0.05); red indicates a change in significance from general linear models. For example, bold red indicates that a difference became significant after transformation; unbold red indicates that a difference lost significance after transformation. Note: proportion mediated may exceed 100% in some regions indicating unstable estimates or inconsistent mediation in which the direct and indirect effects have opposite signs.


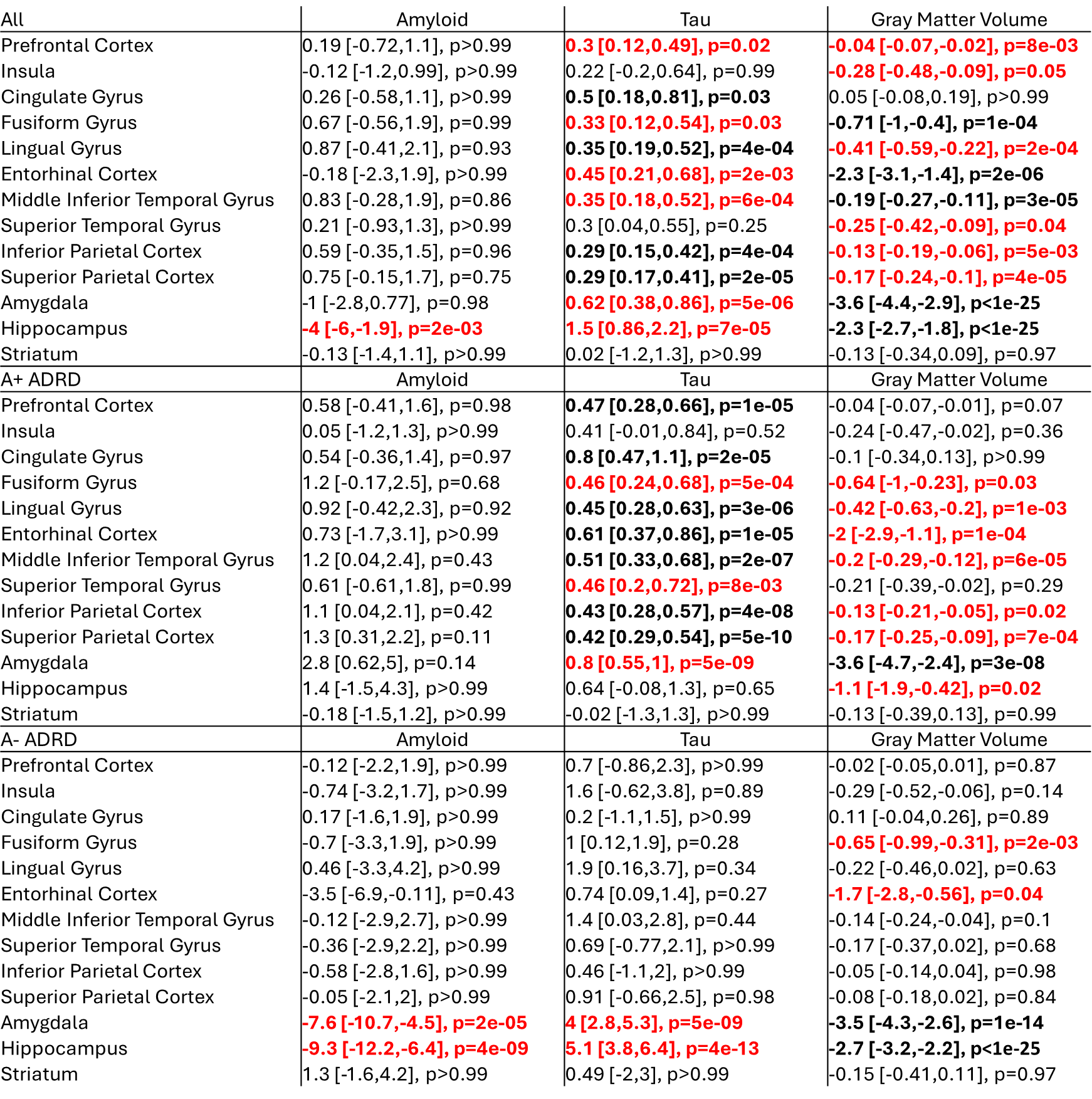


Supplemental Table 9. Standardized estimates (beta) for biomarker regressions across all participants with Alzheimer’s disease and related dementia and cognitively unimpaired adult subgroups (top), amyloid-positive Alzheimer’s disease and related dementia (A+ ADRD) and cognitively unimpaired adult subgroups (middle) and between amyloid-negative ADRD (A- ADRD) and cognitively unimpaired adult subgroups (bottom). Models per region of interest include amyloid, tau, or neurodegeneration as individual predictors of TSPO. Bold indicates significance (p<0.05); red indicates a change in significance from multivariate models. For example, bold red indicates that a difference became in the univariate model; unbold red indicates that a difference lost significance compared to the multivariate model. For all, TSPO-Amyloid N = 41, TSPO-Tau N = 37, TSPO-Neurodegeneration N = 38. For A+ ADRD, TSPO-Amyloid N = 32, TSPO-Tau N = 30, TSPO-Neurodegeneration N = 30. For A- ADRD, TSPO-Amyloid N = 29, TSPO-Tau N = 26, TSPO-Neurodegeneration N = 27.


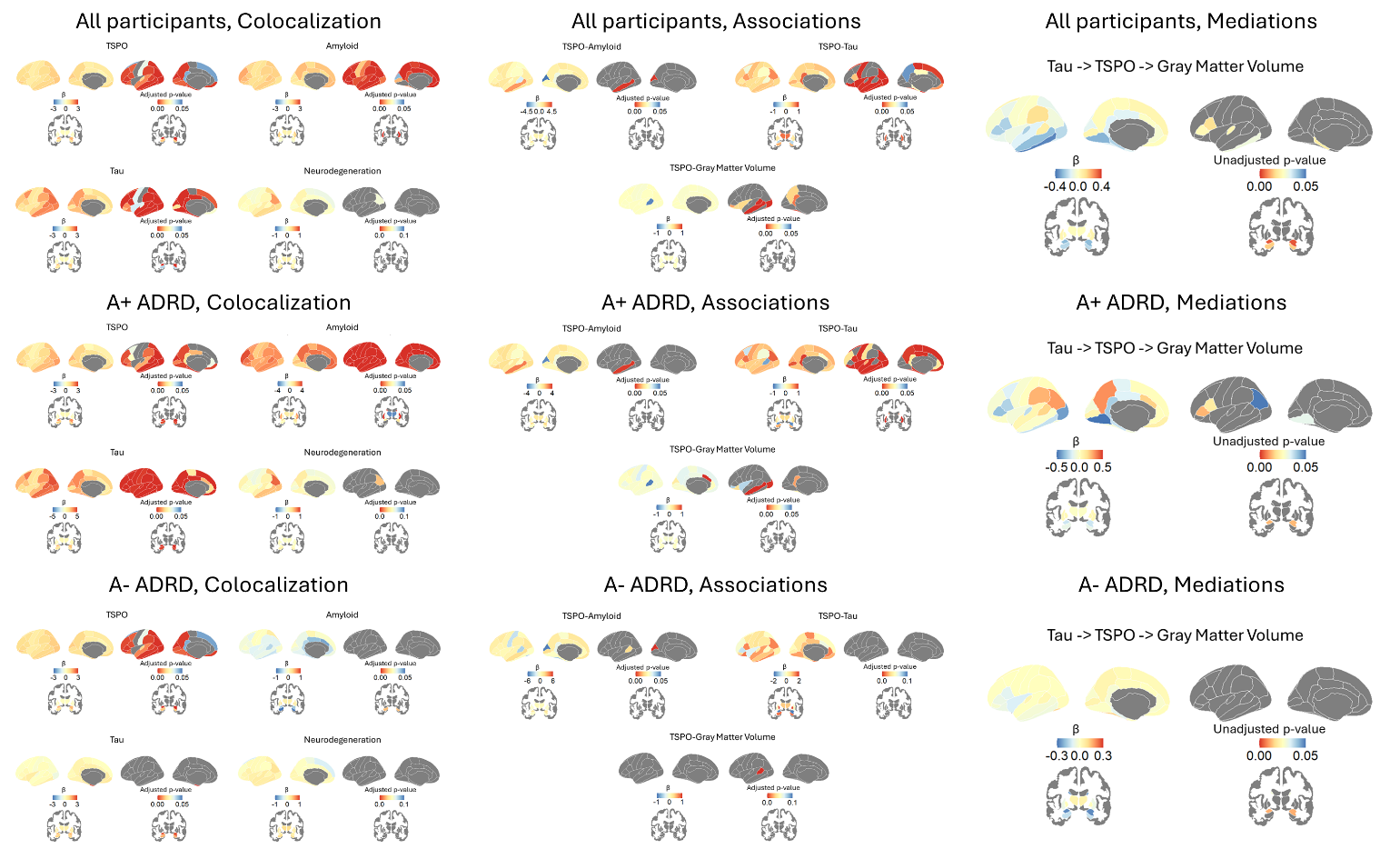


Supplemental Figure 2. Brain-wide investigation of TSPO colocalization, associations, and mediations with amyloid, tau, and neurodegeneration across all participants with Alzheimer’s disease and related dementia and cognitively unimpaired adult subgroups (top), amyloid-positive Alzheimer’s disease and related dementia (A+ ADRD) and cognitively unimpaired adult subgroups (middle) and between amyloid-negative ADRD (A- ADRD) and cognitively unimpaired adult subgroups (bottom). Note that effect size color bar ranges are not uniform across models or groups in order to represent all associations; similarly, unadjusted and adjusted p-values at different thresholds are illustrative in these sensitivity analyses. For colocalization in all participants, TSPO N = 41, Amyloid N = 42, Tau N = 38, Neurodegeneration N = 39; in A+ ADRD, TSPO N = 32, Amyloid N = 33, Tau N = 31, Neurodegeneration N = 31; and in A- ADRD, TSPO N = 29, Amyloid N = 29, Tau N = 26, Neurodegeneration N = 27. For associations and mediations in all participants N = 37; in A+ ADRD N = 30; and in A- ADRD N = 26

R code used in the analyses is as follows:

### Inflammatory alterations mediate tau-associated neurodegeneration

## Hypothesis 1: colocalization

# Compared ADRD to amyloid-negative cognitively unimpaired adults

# count the number of regions that are significantly elevated in TSPO and ATN

# count the number of overlapping regions as support for colocalization

## Hypothesis 2: associations

# Regional associations between TSPO and ATN

# multivariate analyses as support for stronger associations with TSPO and tau

## Hypothesis 3: mediations

# Regional Amyloid->TSPO->Tau

# Earlier Braak Tau->TSPO->Later Braak Tau

# Regional Tau->TSPO->Neurodegeneration

## Exploratory analyses

# Stratify by amyloid positivity

## Sensitivity analyses

# gamma link and log transforms

## Sensitivity analyses

# univariate hypothesis 2

## Sensitivity analyses

# brain-wide hypotheses 1-3

library(readxl)

library(writexl)

library(ggseg)

library(gtsummary)

library(ggpubr)

library(tidyverse)

library(ggseg)

library(RColorBrewer)

library(reshape2)

library(lme4)

library(lmerTest)

library(dplyr)

library(arsenal)

library(sjPlot)

library(ggplot2)

library(ggthemes)

library(gridExtra)

library(blme)

library(interactions)

library(emmeans)

library(Hmisc)

library(ggeffects)

library(kableExtra)

library(lavaan)

library(parameters)

library(lm.beta)

library(patchwork)

### Data available upon request

dat.wide <- read_excel()

### create composite variables

# calculate composite cognitive variables

dat.wide$Delayed_Episodic_Memory_Z_baseline <- (dat.wide$SRT_DR_Z_baseline +

dat.wide$Craft_DV_Z_baseline +

dat.wide$Benson_D_Z_baseline) / 3

dat.wide$Immediate_Episodic_Memory_Z_baseline <- (dat.wide$Craft_IV_Z_baseline +

dat.wide$SRT_TR_Z_baseline) / 2

dat.wide$Attention_Z_baseline <- (dat.wide$Trails_A_Z_baseline +

dat.wide$Numberspan_Forwards_TC_Z_baseline) / 2

dat.wide$Executive_Z_baseline <- (dat.wide$Trails_B_Z_baseline +

dat.wide$Numberspan_Backwards_TC_Z_baseline) / 2

dat.wide$Language_Z_baseline <- (dat.wide$CF_Animals_Z_baseline +

dat.wide$CF_Vegetables_Z_baseline +

dat.wide$CFL_Z_baseline +

dat.wide$MINT_Z_baseline) / 4

dat.wide$Visuospatial_Z_baseline <- (dat.wide$Benson_I_Z_baseline)

boxplot(dat.wide$Delayed_Episodic_Memory_Z_baseline,

dat.wide$Immediate_Episodic_Memory_Z_baseline,

dat.wide$Attention_Z_baseline,

dat.wide$Executive_Z_baseline,

dat.wide$Language_Z_baseline,

dat.wide$Visuospatial_Z_baseline)

# create composite groups based on amyloid positivity and cognitive impairment

dat.wide <- dat.wide %>% mutate(group_baseline = case_when(

Status_baseline == "Control" & Visual_Read_FBB_baseline == "Negative" ~ "A- Control",

Status_baseline == "ADRD" & Visual_Read_FBB_baseline == "Negative" ~ "A- ADRD",

Status_baseline == "ADRD" & Visual_Read_FBB_baseline == "Positive" ~ "A+ ADRD"))

dat.wide$group_baseline <- factor(dat.wide$group_baseline,

levels=c("A- Control", "A+ ADRD", "A- ADRD"))

table(dat.wide$group_baseline)

# create composite subgroups based on amyloid positivity and diagnosis

dat.wide <- dat.wide %>% mutate(subgroup_baseline = case_when(

Diagnosis_baseline == "Control" & Visual_Read_FBB_baseline == "Negative" ~ "A- Control",

Diagnosis_baseline == "MCI" & Visual_Read_FBB_baseline == "Negative" ~ "A- MCI",

Diagnosis_baseline == "MCI" & Visual_Read_FBB_baseline == "Positive" ~ "A+ MCI",

Diagnosis_baseline == "aMCI" & Visual_Read_FBB_baseline == "Positive" ~ "A+ MCI",

Diagnosis_baseline == "AD" & Visual_Read_FBB_baseline == "Negative" ~ "A- AD",

Diagnosis_baseline == "AD" & Visual_Read_FBB_baseline == "Positive" ~ "A+ AD",

Diagnosis_baseline == "PCA" & Visual_Read_FBB_baseline == "Negative" ~ "A- PCA",

Diagnosis_baseline == "PCA" & Visual_Read_FBB_baseline == "Positive" ~ "A+ PCA",

Diagnosis_baseline == "LATE" & Visual_Read_FBB_baseline == "Negative" ~ "A- LATE",

Diagnosis_baseline == "lvPPA" & Visual_Read_FBB_baseline == "Positive" ~ "A+ lvPPA",

Diagnosis_baseline == "FTD" & Visual_Read_FBB_baseline == "Negative" ~ "A- FTD"))

dat.wide$subgroup_baseline <- factor(dat.wide$subgroup_baseline,

levels=c("A- Control", "A- MCI", "A- AD", "A- LATE", "A- FTD", "A- PCA",

"A+ MCI", "A+ AD", "A+ lvPPA", "A+ PCA"))

table(dat.wide$subgroup_baseline)

# biomarker positivity based on controls

dat.wide_controls <- dat.wide[dat.wide$Status_baseline=='Control',]

length(dat.wide_controls$ID)

# 2SD + mean for early Braak

mean_Braak_I_II_MK6240_PVC_baseline <- mean(dat.wide_controls$Braak_I_II_MK6240_PVC_baseline,na.rm=TRUE)

sd_Braak_I_II_MK6240_PVC_baseline <- sd(dat.wide_controls$Braak_I_II_MK6240_PVC_baseline,na.rm=TRUE)

dat.wide$threshold_Braak_I_II_MK6240_PVC_baseline <- mean_Braak_I_II_MK6240_PVC_baseline + (2 * sd_Braak_I_II_MK6240_PVC_baseline)

ggplot(dat.wide, aes(x = Braak_I_II_MK6240_PVC_baseline, fill = subgroup_baseline, colour = subgroup_baseline)) +

geom_histogram(position = "stack", color="black") +

geom_vline(xintercept=dat.wide$threshold_Braak_I_II_MK6240_PVC_baseline, linetype="dashed", color="black", lwd=3) +

theme_bw() +

theme(axis.line = element_line(colour = "black"),

panel.grid.major = element_blank(),

panel.grid.minor = element_blank(),

panel.border = element_blank(),

panel.background = element_blank()) +

scale_fill_brewer(palette = "Spectral") +

theme(axis.text = element_text(color="black")) +

scale_y_continuous(expand = expansion(mult = c(0, 0.05)), limits=c(0,13)) +

scale_x_continuous(limits=c(0,10)) +

theme(plot.margin = margin(1, 2, 0.5, 0.5, "cm")) +

theme(text=element_text(size=30)) + xlab("Braak I/II MK6240 PVC SUVR") + theme(legend.position = "none")

dat.wide <- dat.wide %>% mutate(Braak_I_II_MK6240_PVC_positivity_baseline = case_when(

Braak_I_II_MK6240_PVC_baseline < threshold_Braak_I_II_MK6240_PVC_baseline ~ "Tau Negative",

Braak_I_II_MK6240_PVC_baseline >= threshold_Braak_I_II_MK6240_PVC_baseline ~ "Tau Positive"))

dat.wide$Braak_I_II_MK6240_PVC_positivity_baseline <- as.factor(dat.wide$Braak_I_II_MK6240_PVC_positivity_baseline)

# 2SD + mean for middle Braak

mean_Braak_III_IV_MK6240_PVC_baseline <- mean(dat.wide_controls$Braak_III_IV_MK6240_PVC_baseline,na.rm=TRUE)

sd_Braak_III_IV_MK6240_PVC_baseline <- sd(dat.wide_controls$Braak_III_IV_MK6240_PVC_baseline,na.rm=TRUE)

dat.wide$threshold_Braak_III_IV_MK6240_PVC_baseline <- mean_Braak_III_IV_MK6240_PVC_baseline + (2 * sd_Braak_III_IV_MK6240_PVC_baseline)

ggplot(dat.wide, aes(x = Braak_III_IV_MK6240_PVC_baseline, fill = subgroup_baseline, colour = subgroup_baseline)) +

geom_histogram(position = "stack", color="black") +

geom_vline(xintercept=dat.wide$threshold_Braak_III_IV_MK6240_PVC_baseline, linetype="dashed", color="black", lwd=3) +

theme_bw() +

theme(axis.line = element_line(colour = "black"),

panel.grid.major = element_blank(),

panel.grid.minor = element_blank(),

panel.border = element_blank(),

panel.background = element_blank()) +

scale_fill_brewer(palette = "Spectral") +

theme(axis.text = element_text(color="black")) +

scale_y_continuous(expand = expansion(mult = c(0, 0.05)), limits=c(0,13)) +

scale_x_continuous(limits=c(0,10)) +

theme(plot.margin = margin(1, 2, 0.5, 0.5, "cm")) +

theme(text=element_text(size=30)) + xlab("Braak III/IV MK6240 PVC SUVR") + theme(legend.position = "none")

dat.wide <- dat.wide %>% mutate(Braak_III_IV_MK6240_PVC_positivity_baseline = case_when(

Braak_III_IV_MK6240_PVC_baseline < threshold_Braak_III_IV_MK6240_PVC_baseline ~ "Tau Negative",

Braak_III_IV_MK6240_PVC_baseline >= threshold_Braak_III_IV_MK6240_PVC_baseline ~ "Tau Positive"))

dat.wide$Braak_III_IV_MK6240_PVC_positivity_baseline <- as.factor(dat.wide$Braak_III_IV_MK6240_PVC_positivity_baseline)

# 2SD + mean for late Braak

mean_Braak_V_VI_MK6240_PVC_baseline <- mean(dat.wide_controls$Braak_V_VI_MK6240_PVC_baseline,na.rm=TRUE)

sd_Braak_V_VI_MK6240_PVC_baseline <- sd(dat.wide_controls$Braak_V_VI_MK6240_PVC_baseline,na.rm=TRUE)

dat.wide$threshold_Braak_V_VI_MK6240_PVC_baseline <- mean_Braak_V_VI_MK6240_PVC_baseline + (2 * sd_Braak_V_VI_MK6240_PVC_baseline)

ggplot(dat.wide, aes(x = Braak_V_VI_MK6240_PVC_baseline, fill = subgroup_baseline, colour = subgroup_baseline)) +

geom_histogram(position = "stack", color="black") +

geom_vline(xintercept=dat.wide$threshold_Braak_V_VI_MK6240_PVC_baseline, linetype="dashed", color="black", lwd=3) +

theme_bw() +

theme(axis.line = element_line(colour = "black"),

panel.grid.major = element_blank(),

panel.grid.minor = element_blank(),

panel.border = element_blank(),

panel.background = element_blank()) +

scale_fill_brewer(palette = "Spectral") +

theme(axis.text = element_text(color="black")) +

scale_y_continuous(expand = expansion(mult = c(0, 0.05)), limits=c(0,13)) +

scale_x_continuous(limits=c(0,10)) +

theme(plot.margin = margin(1, 2, 0.5, 0.5, "cm")) +

theme(text=element_text(size=30)) + xlab("Braak V/VI MK6240 PVC SUVR") + theme(legend.position = "none")

dat.wide <- dat.wide %>% mutate(Braak_V_VI_MK6240_PVC_positivity_baseline = case_when(

Braak_V_VI_MK6240_PVC_baseline < threshold_Braak_V_VI_MK6240_PVC_baseline ~ "Tau Negative",

Braak_V_VI_MK6240_PVC_baseline >= threshold_Braak_V_VI_MK6240_PVC_baseline ~ "Tau Positive"))

dat.wide$Braak_V_VI_MK6240_PVC_positivity_baseline <- as.factor(dat.wide$Braak_V_VI_MK6240_PVC_positivity_baseline)

# mean for TSPO, adjusted for TSPO Affinity

# Composite = prefrontal, middle inferior temporal gyrus, superior temporal cortex, cingulate gyrus, inferior parietal cortex, superior parietal cortex

Composite_ER176_PVC_TSPO_Controls_lm <- lm(Composite_ER176_PVC_baseline ~ TSPO_Affinity, data=dat.wide_controls)

Composite_ER176_PVC_TSPO_Controls_lm_summary <- summary(Composite_ER176_PVC_TSPO_Controls_lm)

dat.wide$threshold_Composite_ER176_PVC_baseline_HAB <- Composite_ER176_PVC_TSPO_Controls_lm_summary[[4]][[1]]

dat.wide$threshold_Composite_ER176_PVC_baseline_MAB <- Composite_ER176_PVC_TSPO_Controls_lm_summary[[4]][[1]] +

Composite_ER176_PVC_TSPO_Controls_lm_summary[[4]][[3]]

dat.wide$threshold_Composite_ER176_PVC_baseline_LAB <- Composite_ER176_PVC_TSPO_Controls_lm_summary[[4]][[1]] +

Composite_ER176_PVC_TSPO_Controls_lm_summary[[4]][[2]]

# match colors to be the same as tau

Spectral_colors <- RColorBrewer::brewer.pal(10,"Spectral")[1:10]

HAB_Spectral_colors <- Spectral_colors[c(1,2,3,5,6,7,8,10)]

ggplot(filter(dat.wide, TSPO_Affinity == "High Affinity" & is.na(subgroup_baseline) == 0),

aes(x = Composite_ER176_PVC_baseline, fill = subgroup_baseline, colour = subgroup_baseline)) +

geom_histogram(position = "stack", color="black") +

geom_vline(xintercept=dat.wide$threshold_Composite_ER176_PVC_baseline_HAB, linetype="dashed", color="black", lwd=3) +

theme_bw() +

theme(axis.line = element_line(colour = "black"),

panel.grid.major = element_blank(),

panel.grid.minor = element_blank(),

panel.border = element_blank(),

panel.background = element_blank()) +

scale_fill_manual(values=HAB_Spectral_colors) +

theme(axis.text = element_text(color="black")) +

scale_y_continuous(expand = expansion(mult = c(0, 0.05)), limits=c(0,6)) +

scale_x_continuous(limits=c(0.5,2)) +

theme(plot.margin = margin(1, 2, 0.5, 0.5, "cm")) +

theme(text=element_text(size=28)) + xlab("Composite ER176 PVC SUVR [HAB]") + theme(legend.position = "none")

MAB_Spectral_colors <- Spectral_colors[c(1,2,3,4,7,8,10)]

ggplot(filter(dat.wide, TSPO_Affinity == "Mixed"),

aes(x = Composite_ER176_PVC_baseline, fill = subgroup_baseline, colour = subgroup_baseline)) +

geom_histogram(position = "stack", color="black") +

geom_vline(xintercept=dat.wide$threshold_Composite_ER176_PVC_baseline_MAB, linetype="dashed", color="black", lwd=3) +

theme_bw() +

theme(axis.line = element_line(colour = "black"),

panel.grid.major = element_blank(),

panel.grid.minor = element_blank(),

panel.border = element_blank(),

panel.background = element_blank()) +

scale_fill_manual(values=MAB_Spectral_colors) +

theme(axis.text = element_text(color="black")) +

scale_y_continuous(expand = expansion(mult = c(0, 0.05)), limits=c(0,6)) +

scale_x_continuous(limits=c(0.5,2)) +

theme(plot.margin = margin(1, 2, 0.5, 0.5, "cm")) +

theme(text=element_text(size=28)) + xlab("Composite ER176 PVC SUVR [MAB]") + theme(legend.position = "none")

LAB_Spectral_colors <- Spectral_colors[c(1,7,8,10)]

ggplot(filter(dat.wide, TSPO_Affinity == "Low Affinity"),

aes(x = Composite_ER176_PVC_baseline, fill = subgroup_baseline, colour = subgroup_baseline)) +

geom_histogram(position = "stack", color="black") +

geom_vline(xintercept=dat.wide$threshold_Composite_ER176_PVC_baseline_LAB, linetype="dashed", color="black", lwd=3) +

theme_bw() +

theme(axis.line = element_line(colour = "black"),

panel.grid.major = element_blank(),

panel.grid.minor = element_blank(),

panel.border = element_blank(),

panel.background = element_blank()) +

scale_fill_manual(values=LAB_Spectral_colors) +

theme(axis.text = element_text(color="black")) +

scale_y_continuous(expand = expansion(mult = c(0, 0.05)), limits=c(0,6)) +

scale_x_continuous(limits=c(0.5,2)) +

theme(plot.margin = margin(1, 2, 0.5, 0.5, "cm")) +

theme(text=element_text(size=28)) + xlab("Composite ER176 PVC SUVR [LAB]") + theme(legend.position = "none")

dat.wide <- dat.wide %>% mutate(Composite_ER176_PVC_positivity_baseline_TSPO = case_when(

TSPO_Affinity == "High Affinity" & Composite_ER176_PVC_baseline < threshold_Composite_ER176_PVC_baseline_HAB ~ "TSPO Negative",

TSPO_Affinity == "High Affinity" & Composite_ER176_PVC_baseline >= threshold_Composite_ER176_PVC_baseline_HAB ~ "TSPO Positive",

TSPO_Affinity == "Mixed" & Composite_ER176_PVC_baseline < threshold_Composite_ER176_PVC_baseline_MAB ~ "TSPO Negative",

TSPO_Affinity == "Mixed" & Composite_ER176_PVC_baseline >= threshold_Composite_ER176_PVC_baseline_MAB ~ "TSPO Positive",

TSPO_Affinity == "Low Affinity" & Composite_ER176_PVC_baseline < threshold_Composite_ER176_PVC_baseline_LAB ~ "TSPO Negative",

TSPO_Affinity == "Low Affinity" & Composite_ER176_PVC_baseline >= threshold_Composite_ER176_PVC_baseline_LAB ~ "TSPO Positive",

))

dat.wide$Composite_ER176_PVC_positivity_baseline_TSPO <- as.factor(dat.wide$Composite_ER176_PVC_positivity_baseline_TSPO)

# specify covariates

cov.list = c('Age_baseline', 'Sex', 'BMI_baseline',

'Visual_Read_FBB_baseline', 'Status_baseline',

'group_baseline', 'subgroup_baseline',

'APOE_Genotype', 'APOE4', 'TSPO_Affinity', 'MMSE_baseline',

'Delayed_Episodic_Memory_Z_baseline',

'Immediate_Episodic_Memory_Z_baseline',

'Attention_Z_baseline',

'Executive_Z_baseline',

'Language_Z_baseline',

'Visuospatial_Z_baseline', 'ICV_baseline',

'Composite_ER176_PVC_positivity_baseline_TSPO',

'Braak_I_II_MK6240_PVC_positivity_baseline',

'Diagnosis_Age','Disease_Duration')

# convert to long format

tmp.er176 <- dat.wide %>%

select(ID, cov.list, contains('ER176_PVC_baseline')) %>%

melt(., id = c('ID',cov.list)) %>%

rename(ER176=value) %>%

mutate(roi = gsub('_ER176_PVC_baseline','',variable)) %>%

select(-variable)

tmp.tau <- dat.wide %>%

select(ID, cov.list, contains('_MK6240_PVC_baseline')) %>%

melt(., id = c('ID',cov.list)) %>%

rename(MK6240=value) %>%

mutate(roi = gsub('_MK6240_PVC_baseline','',variable)) %>%

select(-variable)

tmp.amyloid <- dat.wide %>%

select(ID, cov.list, contains('_FBB_baseline')) %>%

melt(., id = c('ID',cov.list)) %>%

rename(FBB=value) %>%

#mutate(FBB = as.numeric(as.character(FBB))) %>%

mutate(roi = gsub('_FBB_baseline','',variable)) %>%

select(-variable)

tmp.volume <- dat.wide %>%

select(ID, cov.list, contains('_Volume_MK6240_baseline')) %>%

melt(., id = c('ID',cov.list)) %>%

rename(Volume_MK6240=value) %>%

mutate(roi = gsub('_Volume_MK6240_baseline','',variable)) %>%

select(-variable)

# 13 a priori regions

roilist = c('Prefrontal','MiddleInfTempGyrus','SuperiorTemp','G_Cing',

'Hippocampus','Amygdala','FusiformGyrus','SuperiorParietal','InferiorParietal',

'LingualGyrus','Striatum','Insula','EntorhinalCortex_FS')

analysis.dat.long <-tmp.er176 %>%

left_join(., tmp.tau) %>%

left_join(., tmp.amyloid) %>%

left_join(., tmp.volume) %>%

select(ID, roi, everything(.)) %>%

arrange(ID, roi) %>%

filter(roi %in% roilist)

# summarize

analysis.dat.long_TSPO <- analysis.dat.long[complete.cases(analysis.dat.long$TSPO_Affinity),]

summary(tableby( Status_baseline~ FBB + MK6240 + ER176 + Volume_MK6240, strata = roi, test=FALSE,

numeric.stats = c("N", "meansd"), numeric.simplify=TRUE,

analysis.dat.long_TSPO))

table(analysis.dat.long_TSPO$roi)

# set reference levels

dat.wide <- within(dat.wide, TSPO_Affinity <- relevel(factor(TSPO_Affinity), ref = "Low Affinity"))

dat.wide <- within(dat.wide, APOE4 <- relevel(factor(APOE4), ref = "Non-Carrier"))

dat.wide <- within(dat.wide, Status_baseline <- relevel(factor(Status_baseline), ref = "Control"))

analysis.dat.long <- within(analysis.dat.long, roi <- relevel(factor(roi), ref = "Striatum"))

analysis.dat.long <- within(analysis.dat.long, TSPO_Affinity <- relevel(factor(TSPO_Affinity), ref = "Low Affinity"))

analysis.dat.long <- within(analysis.dat.long, APOE4 <- relevel(factor(APOE4), ref = "Non-Carrier"))

analysis.dat.long <- within(analysis.dat.long, Status_baseline <- relevel(factor(Status_baseline), ref = "Control"))

## for exploratory analyses, subset the data

#analysis.dat.long <- analysis.dat.long[analysis.dat.long$group_baseline == "A- Control" | analysis.dat.long$group_baseline == "A+ ADRD",]

#dat.wide <- dat.wide[dat.wide$group_baseline == "A- Control" | dat.wide$group_baseline == "A+ ADRD",]

#analysis.dat.long <- analysis.dat.long[analysis.dat.long$Visual_Read_FBB_baseline=="Negative",]

#dat.wide <- dat.wide[dat.wide$Visual_Read_FBB_baseline=="Negative",]

#table(analysis.dat.long$group_baseline)

#table(dat.wide$group_baseline)

# complete cases by FBB and TSPO Affinity

dat.wide_cc <- dat.wide[complete.cases(dat.wide[,c("Insula_FBB_baseline","TSPO_Affinity")]),]

table(dat.wide_cc$Status_baseline)

# 25 ADRD, 21 Controls

### Hypothesis 1: colocalization

## TSPO

analysis.dat.long_TSPO <- analysis.dat.long[complete.cases(analysis.dat.long[c("ER176", "Status_baseline", "roi",

"TSPO_Affinity","Age_baseline","Sex","BMI_baseline","APOE4")]),]

unique(analysis.dat.long_TSPO$ID)

TSPO_Status_lm_full3<-lm(ER176 ~ Status_baseline*roi + TSPO_Affinity + Age_baseline + Sex + BMI_baseline + APOE4,

analysis.dat.long %>% filter(roi %in% roilist))

TSPO_Status_lm_full3_summary<-summary(TSPO_Status_lm_full3)

TSPO_Status_lm_full3_mean <- emmeans(TSPO_Status_lm_full3, spec = pairwise ~ Status_baseline | roi,

weights="proportional",adjust="mvt") %>% summary(infer=TRUE)

# save as data frame to extract estimates

TSPO_Status_lm_full3_mean_df <- as.data.frame(TSPO_Status_lm_full3_mean)

# tspo std

pwpm(emmeans(TSPO_Status_lm_full3, spec = pairwise ~ Status_baseline | roi,

weights="proportional",adjust="mvt"))

TSPO_Status_lm_full3_std_mean <- as.data.frame(eff_size(emmeans(TSPO_Status_lm_full3, spec = pairwise ~ Status_baseline | roi,

weights="proportional",adjust="mvt"), sigma = sigma(TSPO_Status_lm_full3), edf = TSPO_Status_lm_full3_summary[[7]][[2]]))

TSPO_Status_lm_full3_std_mean_df <- as.data.frame(TSPO_Status_lm_full3_std_mean)

# std ADRD - Control

ER176_PVC_individual_cortical_regions_std_Difference = data.frame(

region = c("medial orbitofrontal", "lateral orbitofrontal","pars opercularis", "pars orbitalis", "pars triangularis", "frontal pole", "rostral middle frontal", "caudal middle frontal",

"entorhinal",

"inferior temporal", "middle temporal",

"superior temporal", "bankssts", "transverse temporal",

"caudal anterior cingulate", "rostral anterior cingulate","isthmus cingulate", "posterior cingulate",

"superior parietal",

"inferior parietal",

"fusiform",

"lingual",

"insula"),

mean = -1 * c(rep(TSPO_Status_lm_full3_std_mean_df[(TSPO_Status_lm_full3_std_mean_df$roi == "Prefrontal" & TSPO_Status_lm_full3_std_mean_df$contrast == "(Control - ADRD)"), "effect.size"],8),

rep(TSPO_Status_lm_full3_std_mean_df[(TSPO_Status_lm_full3_std_mean_df$roi == "EntorhinalCortex_FS" & TSPO_Status_lm_full3_std_mean_df$contrast == "(Control - ADRD)"), "effect.size"],1),

rep(TSPO_Status_lm_full3_std_mean_df[(TSPO_Status_lm_full3_std_mean_df$roi == "MiddleInfTempGyrus" & TSPO_Status_lm_full3_std_mean_df$contrast == "(Control - ADRD)"), "effect.size"],2),

rep(TSPO_Status_lm_full3_std_mean_df[(TSPO_Status_lm_full3_std_mean_df$roi == "SuperiorTemp" & TSPO_Status_lm_full3_std_mean_df$contrast == "(Control - ADRD)"), "effect.size"],3),

rep(TSPO_Status_lm_full3_std_mean_df[(TSPO_Status_lm_full3_std_mean_df$roi == "G_Cing" & TSPO_Status_lm_full3_std_mean_df$contrast == "(Control - ADRD)"), "effect.size"],4),

rep(TSPO_Status_lm_full3_std_mean_df[(TSPO_Status_lm_full3_std_mean_df$roi == "SuperiorParietal" & TSPO_Status_lm_full3_std_mean_df$contrast == "(Control - ADRD)"), "effect.size"],1),

rep(TSPO_Status_lm_full3_std_mean_df[(TSPO_Status_lm_full3_std_mean_df$roi == "InferiorParietal" & TSPO_Status_lm_full3_std_mean_df$contrast == "(Control - ADRD)"), "effect.size"],1),

rep(TSPO_Status_lm_full3_std_mean_df[(TSPO_Status_lm_full3_std_mean_df$roi == "FusiformGyrus" & TSPO_Status_lm_full3_std_mean_df$contrast == "(Control - ADRD)"), "effect.size"],1),

rep(TSPO_Status_lm_full3_std_mean_df[(TSPO_Status_lm_full3_std_mean_df$roi == "LingualGyrus" & TSPO_Status_lm_full3_std_mean_df$contrast == "(Control - ADRD)"), "effect.size"],1),

rep(TSPO_Status_lm_full3_std_mean_df[(TSPO_Status_lm_full3_std_mean_df$roi == "Insula" & TSPO_Status_lm_full3_std_mean_df$contrast == "(Control - ADRD)"), "effect.size"],1)),

stringsAsFactors = FALSE)

ER176_PVC_individual_cortical_regions_std_Difference_p <- ggseg(.data=ER176_PVC_individual_cortical_regions_std_Difference, colour="white", mapping=aes(fill=mean), position="stacked", hemi="left") +

theme_void() +

ggtitle(sprintf("TSPO")) +

theme(plot.title = element_text(hjust = 0.5)) +

theme(text = element_text(size = 30)) +

scale_fill_distiller(palette = "Spectral", direction=-1,

breaks=c(-3,0,3),limits=c(-3,3),

name="") + theme(legend.position = "bottom") + theme(plot.margin = margin(t=0, r=0, b=0, l=0, "pt"))

ER176_PVC_individual_subcortical_regions_std_Difference = data.frame(

region = c("hippocampus",

"amygdala",

"caudate","putamen"),

mean = -1 * c(rep(TSPO_Status_lm_full3_std_mean_df[(TSPO_Status_lm_full3_std_mean_df$roi == "Hippocampus" & TSPO_Status_lm_full3_std_mean_df$contrast == "(Control - ADRD)"), "effect.size"],1),

rep(TSPO_Status_lm_full3_std_mean_df[(TSPO_Status_lm_full3_std_mean_df$roi == "Amygdala" & TSPO_Status_lm_full3_std_mean_df$contrast == "(Control - ADRD)"), "effect.size"],1),

rep(TSPO_Status_lm_full3_std_mean_df[(TSPO_Status_lm_full3_std_mean_df$roi == "Striatum" & TSPO_Status_lm_full3_std_mean_df$contrast == "(Control - ADRD)"), "effect.size"],2)),

Status = c(rep("Controls",4),rep("ADRD",4)),

stringsAsFactors = FALSE)

ER176_PVC_individual_subcortical_regions_std_Difference_p <- ggplot(ER176_PVC_individual_subcortical_regions_std_Difference) +

geom_brain(atlas=aseg, side="coronal", colour="white", mapping=aes(fill=mean)) +

theme_void() +

scale_fill_distiller(palette = "Spectral", direction=-1,

breaks=c(-3,0,3),limits=c(-3,3),

name="") + theme(legend.position = "none") + theme(plot.margin = margin(t=0, r=0, b=0, l=0, "pt"))

ER176_PVC_individual_regions_std_Difference_p <- ER176_PVC_individual_cortical_regions_std_Difference_p / ER176_PVC_individual_subcortical_regions_std_Difference_p + plot_layout(heights = c(1.25,1))

ER176_PVC_individual_regions_std_Difference_p

# std forest plot for ER176_PVC

dat_forest_ER176_PVC_std_by_Status <- data.frame(matrix(ncol = 1, nrow = 13))

dat_forest_ER176_PVC_std_by_Status$model <- c("Prefrontal Cortex","Insula",

"Cingulate Gyrus","Fusiform Gyrus","Lingual Gyrus",

"Entorhinal Cortex",

"Middle Inferior Temporal Gyrus","Superior Temporal Gyrus",

"Inferior Parietal Cortex","Superior Parietal Cortex",

"Amygdala","Hippocampus","Striatum")

dat_forest_ER176_PVC_std_by_Status$model <- factor(dat_forest_ER176_PVC_by_Status$model,

levels=c("Prefrontal Cortex","Insula",

"Cingulate Gyrus","Fusiform Gyrus","Lingual Gyrus",

"Entorhinal Cortex",

"Middle Inferior Temporal Gyrus","Superior Temporal Gyrus",

"Inferior Parietal Cortex","Superior Parietal Cortex",

"Amygdala","Hippocampus","Striatum"))

dat_forest_ER176_PVC_std_by_Status$estimate <- c(TSPO_Status_lm_full3_std_mean_df[(TSPO_Status_lm_full3_std_mean_df$roi == "Prefrontal" & TSPO_Status_lm_full3_std_mean_df$contrast == "(Control - ADRD)"), "effect.size"],

TSPO_Status_lm_full3_std_mean_df[(TSPO_Status_lm_full3_std_mean_df$roi == "Insula" & TSPO_Status_lm_full3_std_mean_df$contrast == "(Control - ADRD)"), "effect.size"],

TSPO_Status_lm_full3_std_mean_df[(TSPO_Status_lm_full3_std_mean_df$roi == "G_Cing" & TSPO_Status_lm_full3_std_mean_df$contrast == "(Control - ADRD)"), "effect.size"],

TSPO_Status_lm_full3_std_mean_df[(TSPO_Status_lm_full3_std_mean_df$roi == "FusiformGyrus" & TSPO_Status_lm_full3_std_mean_df$contrast == "(Control - ADRD)"), "effect.size"],

TSPO_Status_lm_full3_std_mean_df[(TSPO_Status_lm_full3_std_mean_df$roi == "LingualGyrus" & TSPO_Status_lm_full3_std_mean_df$contrast == "(Control - ADRD)"), "effect.size"],

TSPO_Status_lm_full3_std_mean_df[(TSPO_Status_lm_full3_std_mean_df$roi == "EntorhinalCortex_FS" & TSPO_Status_lm_full3_std_mean_df$contrast == "(Control - ADRD)"), "effect.size"],

TSPO_Status_lm_full3_std_mean_df[(TSPO_Status_lm_full3_std_mean_df$roi == "MiddleInfTempGyrus" & TSPO_Status_lm_full3_std_mean_df$contrast == "(Control - ADRD)"), "effect.size"],

TSPO_Status_lm_full3_std_mean_df[(TSPO_Status_lm_full3_std_mean_df$roi == "SuperiorTemp" & TSPO_Status_lm_full3_std_mean_df$contrast == "(Control - ADRD)"), "effect.size"],

TSPO_Status_lm_full3_std_mean_df[(TSPO_Status_lm_full3_std_mean_df$roi == "InferiorParietal" & TSPO_Status_lm_full3_std_mean_df$contrast == "(Control - ADRD)"), "effect.size"],

TSPO_Status_lm_full3_std_mean_df[(TSPO_Status_lm_full3_std_mean_df$roi == "SuperiorParietal" & TSPO_Status_lm_full3_std_mean_df$contrast == "(Control - ADRD)"), "effect.size"],

TSPO_Status_lm_full3_std_mean_df[(TSPO_Status_lm_full3_std_mean_df$roi == "Amygdala" & TSPO_Status_lm_full3_std_mean_df$contrast == "(Control - ADRD)"), "effect.size"],

TSPO_Status_lm_full3_std_mean_df[(TSPO_Status_lm_full3_std_mean_df$roi == "Hippocampus" & TSPO_Status_lm_full3_std_mean_df$contrast == "(Control - ADRD)"), "effect.size"],

TSPO_Status_lm_full3_std_mean_df[(TSPO_Status_lm_full3_std_mean_df$roi == "Striatum" & TSPO_Status_lm_full3_std_mean_df$contrast == "(Control - ADRD)"), "effect.size"]) * -1

dat_forest_ER176_PVC_std_by_Status$low_CI <- c(TSPO_Status_lm_full3_std_mean_df[(TSPO_Status_lm_full3_std_mean_df$roi == "Prefrontal" & TSPO_Status_lm_full3_std_mean_df$contrast == "(Control - ADRD)"), "lower.CL"],

TSPO_Status_lm_full3_std_mean_df[(TSPO_Status_lm_full3_std_mean_df$roi == "Insula" & TSPO_Status_lm_full3_std_mean_df$contrast == "(Control - ADRD)"), "lower.CL"],

TSPO_Status_lm_full3_std_mean_df[(TSPO_Status_lm_full3_std_mean_df$roi == "G_Cing" & TSPO_Status_lm_full3_std_mean_df$contrast == "(Control - ADRD)"), "lower.CL"],

TSPO_Status_lm_full3_std_mean_df[(TSPO_Status_lm_full3_std_mean_df$roi == "FusiformGyrus" & TSPO_Status_lm_full3_std_mean_df$contrast == "(Control - ADRD)"), "lower.CL"],

TSPO_Status_lm_full3_std_mean_df[(TSPO_Status_lm_full3_std_mean_df$roi == "LingualGyrus" & TSPO_Status_lm_full3_std_mean_df$contrast == "(Control - ADRD)"), "lower.CL"],

TSPO_Status_lm_full3_std_mean_df[(TSPO_Status_lm_full3_std_mean_df$roi == "EntorhinalCortex_FS" & TSPO_Status_lm_full3_std_mean_df$contrast == "(Control - ADRD)"), "lower.CL"],

TSPO_Status_lm_full3_std_mean_df[(TSPO_Status_lm_full3_std_mean_df$roi == "MiddleInfTempGyrus" & TSPO_Status_lm_full3_std_mean_df$contrast == "(Control - ADRD)"), "lower.CL"],

TSPO_Status_lm_full3_std_mean_df[(TSPO_Status_lm_full3_std_mean_df$roi == "SuperiorTemp" & TSPO_Status_lm_full3_std_mean_df$contrast == "(Control - ADRD)"), "lower.CL"],

TSPO_Status_lm_full3_std_mean_df[(TSPO_Status_lm_full3_std_mean_df$roi == "InferiorParietal" & TSPO_Status_lm_full3_std_mean_df$contrast == "(Control - ADRD)"), "lower.CL"],

TSPO_Status_lm_full3_std_mean_df[(TSPO_Status_lm_full3_std_mean_df$roi == "SuperiorParietal" & TSPO_Status_lm_full3_std_mean_df$contrast == "(Control - ADRD)"), "lower.CL"],

TSPO_Status_lm_full3_std_mean_df[(TSPO_Status_lm_full3_std_mean_df$roi == "Amygdala" & TSPO_Status_lm_full3_std_mean_df$contrast == "(Control - ADRD)"), "lower.CL"],

TSPO_Status_lm_full3_std_mean_df[(TSPO_Status_lm_full3_std_mean_df$roi == "Hippocampus" & TSPO_Status_lm_full3_std_mean_df$contrast == "(Control - ADRD)"), "lower.CL"],

TSPO_Status_lm_full3_std_mean_df[(TSPO_Status_lm_full3_std_mean_df$roi == "Striatum" & TSPO_Status_lm_full3_std_mean_df$contrast == "(Control - ADRD)"), "lower.CL"]) * -1

dat_forest_ER176_PVC_std_by_Status$high_CI <- c(TSPO_Status_lm_full3_std_mean_df[(TSPO_Status_lm_full3_std_mean_df$roi == "Prefrontal" & TSPO_Status_lm_full3_std_mean_df$contrast == "(Control - ADRD)"), "upper.CL"],

TSPO_Status_lm_full3_std_mean_df[(TSPO_Status_lm_full3_std_mean_df$roi == "Insula" & TSPO_Status_lm_full3_std_mean_df$contrast == "(Control - ADRD)"), "upper.CL"],

TSPO_Status_lm_full3_std_mean_df[(TSPO_Status_lm_full3_std_mean_df$roi == "G_Cing" & TSPO_Status_lm_full3_std_mean_df$contrast == "(Control - ADRD)"), "upper.CL"],

TSPO_Status_lm_full3_std_mean_df[(TSPO_Status_lm_full3_std_mean_df$roi == "FusiformGyrus" & TSPO_Status_lm_full3_std_mean_df$contrast == "(Control - ADRD)"), "upper.CL"],

TSPO_Status_lm_full3_std_mean_df[(TSPO_Status_lm_full3_std_mean_df$roi == "LingualGyrus" & TSPO_Status_lm_full3_std_mean_df$contrast == "(Control - ADRD)"), "upper.CL"],

TSPO_Status_lm_full3_std_mean_df[(TSPO_Status_lm_full3_std_mean_df$roi == "EntorhinalCortex_FS" & TSPO_Status_lm_full3_std_mean_df$contrast == "(Control - ADRD)"), "upper.CL"],

TSPO_Status_lm_full3_std_mean_df[(TSPO_Status_lm_full3_std_mean_df$roi == "MiddleInfTempGyrus" & TSPO_Status_lm_full3_std_mean_df$contrast == "(Control - ADRD)"), "upper.CL"],

TSPO_Status_lm_full3_std_mean_df[(TSPO_Status_lm_full3_std_mean_df$roi == "SuperiorTemp" & TSPO_Status_lm_full3_std_mean_df$contrast == "(Control - ADRD)"), "upper.CL"],

TSPO_Status_lm_full3_std_mean_df[(TSPO_Status_lm_full3_std_mean_df$roi == "InferiorParietal" & TSPO_Status_lm_full3_std_mean_df$contrast == "(Control - ADRD)"), "upper.CL"],

TSPO_Status_lm_full3_std_mean_df[(TSPO_Status_lm_full3_std_mean_df$roi == "SuperiorParietal" & TSPO_Status_lm_full3_std_mean_df$contrast == "(Control - ADRD)"), "upper.CL"],

TSPO_Status_lm_full3_std_mean_df[(TSPO_Status_lm_full3_std_mean_df$roi == "Amygdala" & TSPO_Status_lm_full3_std_mean_df$contrast == "(Control - ADRD)"), "upper.CL"],

TSPO_Status_lm_full3_std_mean_df[(TSPO_Status_lm_full3_std_mean_df$roi == "Hippocampus" & TSPO_Status_lm_full3_std_mean_df$contrast == "(Control - ADRD)"), "upper.CL"],

TSPO_Status_lm_full3_std_mean_df[(TSPO_Status_lm_full3_std_mean_df$roi == "Striatum" & TSPO_Status_lm_full3_std_mean_df$contrast == "(Control - ADRD)"), "upper.CL"]) * -1

p_ER176_PVC_std_by_Status <-

dat_forest_ER176_PVC_std_by_Status |>

ggplot(aes(y = fct_rev(model))) +

theme_bw() +

geom_point(aes(x=estimate), shape=12, size=3) +

geom_linerange(aes(xmin=low_CI, xmax=high_CI)) +

geom_vline(xintercept = 0, linetype="dashed") +

labs(x="ADRD - Control", y="") +

coord_cartesian(ylim=c(1,13), xlim=c(-0.75, 3)) +

theme(text = element_text(size = 20), axis.text = element_text(color="black")) +

theme(axis.line = element_line(colour = "black"),

panel.grid.major = element_blank(),

panel.grid.minor = element_blank(),

panel.border = element_blank(),

panel.background = element_blank())

p_ER176_PVC_std_by_Status

## Tau

analysis.dat.long_Tau <- analysis.dat.long[complete.cases(analysis.dat.long[c("MK6240", "Status_baseline", "roi",

"Age_baseline","Sex","BMI_baseline","APOE4")]),]

unique(analysis.dat.long_Tau$ID)

Tau_Status_lm_full3<-lm(MK6240 ~ Status_baseline*roi + Age_baseline + Sex + BMI_baseline + APOE4,

analysis.dat.long %>% filter(roi %in% roilist))

Tau_Status_lm_full3_summary<-summary(Tau_Status_lm_full3)

Tau_Status_lm_full3_mean <- emmeans(Tau_Status_lm_full3, spec = pairwise ~ Status_baseline | roi,

weights="proportional",adjust="mvt") %>% summary(infer=TRUE)

Tau_Status_lm_full3_mean_df <- as.data.frame(Tau_Status_lm_full3_mean)

# tau std

pwpm(emmeans(Tau_Status_lm_full3, spec = pairwise ~ Status_baseline | roi,

weights="proportional",adjust="mvt"))

Tau_Status_lm_full3_std_mean <- as.data.frame(eff_size(emmeans(Tau_Status_lm_full3, spec = pairwise ~ Status_baseline | roi,

weights="proportional",adjust="mvt"), sigma = sigma(Tau_Status_lm_full3), edf = Tau_Status_lm_full3_summary[[7]][[2]]))

Tau_Status_lm_full3_std_mean_df <- as.data.frame(Tau_Status_lm_full3_std_mean)

# std ADRD - Control

MK6240_PVC_individual_cortical_regions_std_Difference = data.frame(

region = c("medial orbitofrontal", "lateral orbitofrontal","pars opercularis", "pars orbitalis", "pars triangularis", "frontal pole", "rostral middle frontal", "caudal middle frontal",

"entorhinal",

"inferior temporal", "middle temporal",

"superior temporal", "bankssts", "transverse temporal",

"caudal anterior cingulate", "rostral anterior cingulate","isthmus cingulate", "posterior cingulate",

"superior parietal",

"inferior parietal",

"fusiform",

"lingual",

"insula"),

mean = -1 * c(rep(Tau_Status_lm_full3_std_mean_df[(Tau_Status_lm_full3_std_mean_df$roi == "Prefrontal" & Tau_Status_lm_full3_std_mean_df$contrast == "(Control - ADRD)"), "effect.size"],8),

rep(Tau_Status_lm_full3_std_mean_df[(Tau_Status_lm_full3_std_mean_df$roi == "EntorhinalCortex_FS" & Tau_Status_lm_full3_std_mean_df$contrast == "(Control - ADRD)"), "effect.size"],1),

rep(Tau_Status_lm_full3_std_mean_df[(Tau_Status_lm_full3_std_mean_df$roi == "MiddleInfTempGyrus" & Tau_Status_lm_full3_std_mean_df$contrast == "(Control - ADRD)"), "effect.size"],2),

rep(Tau_Status_lm_full3_std_mean_df[(Tau_Status_lm_full3_std_mean_df$roi == "SuperiorTemp" & Tau_Status_lm_full3_std_mean_df$contrast == "(Control - ADRD)"), "effect.size"],3),

rep(Tau_Status_lm_full3_std_mean_df[(Tau_Status_lm_full3_std_mean_df$roi == "G_Cing" & Tau_Status_lm_full3_std_mean_df$contrast == "(Control - ADRD)"), "effect.size"],4),

rep(Tau_Status_lm_full3_std_mean_df[(Tau_Status_lm_full3_std_mean_df$roi == "SuperiorParietal" & Tau_Status_lm_full3_std_mean_df$contrast == "(Control - ADRD)"), "effect.size"],1),

rep(Tau_Status_lm_full3_std_mean_df[(Tau_Status_lm_full3_std_mean_df$roi == "InferiorParietal" & Tau_Status_lm_full3_std_mean_df$contrast == "(Control - ADRD)"), "effect.size"],1),

rep(Tau_Status_lm_full3_std_mean_df[(Tau_Status_lm_full3_std_mean_df$roi == "FusiformGyrus" & Tau_Status_lm_full3_std_mean_df$contrast == "(Control - ADRD)"), "effect.size"],1),

rep(Tau_Status_lm_full3_std_mean_df[(Tau_Status_lm_full3_std_mean_df$roi == "LingualGyrus" & Tau_Status_lm_full3_std_mean_df$contrast == "(Control - ADRD)"), "effect.size"],1),

rep(Tau_Status_lm_full3_std_mean_df[(Tau_Status_lm_full3_std_mean_df$roi == "Insula" & Tau_Status_lm_full3_std_mean_df$contrast == "(Control - ADRD)"), "effect.size"],1)),

stringsAsFactors = FALSE)

MK6240_PVC_individual_cortical_regions_std_Difference_p <- ggseg(.data=MK6240_PVC_individual_cortical_regions_std_Difference, colour="white", mapping=aes(fill=mean), position="stacked", hemi="left") +

theme_void() +

ggtitle(sprintf("Tau")) +

theme(plot.title = element_text(hjust = 0.5)) +

theme(text = element_text(size = 30)) +

scale_fill_distiller(palette = "Spectral", direction=-1,

breaks=c(-3,0,3),limits=c(-3,3),

name="") + theme(legend.position = "bottom") + theme(plot.margin = margin(t=0, r=0, b=0, l=0, "pt"))

MK6240_PVC_individual_subcortical_regions_std_Difference = data.frame(

region = c("hippocampus",

"amygdala",

"caudate","putamen"),

mean = -1 * c(rep(Tau_Status_lm_full3_std_mean_df[(Tau_Status_lm_full3_std_mean_df$roi == "Hippocampus" & Tau_Status_lm_full3_std_mean_df$contrast == "(Control - ADRD)"), "effect.size"],1),

rep(Tau_Status_lm_full3_std_mean_df[(Tau_Status_lm_full3_std_mean_df$roi == "Amygdala" & Tau_Status_lm_full3_std_mean_df$contrast == "(Control - ADRD)"), "effect.size"],1),

rep(Tau_Status_lm_full3_std_mean_df[(Tau_Status_lm_full3_std_mean_df$roi == "Striatum" & Tau_Status_lm_full3_std_mean_df$contrast == "(Control - ADRD)"), "effect.size"],2)),

Status = c(rep("Controls",4),rep("ADRD",4)),

stringsAsFactors = FALSE)

MK6240_PVC_individual_subcortical_regions_std_Difference_p <- ggplot(MK6240_PVC_individual_subcortical_regions_std_Difference) +

geom_brain(atlas=aseg, side="coronal", colour="white", mapping=aes(fill=mean)) +

theme_void() +

scale_fill_distiller(palette = "Spectral", direction=-1,

breaks=c(-3,0,3),limits=c(-3,3),

name="") + theme(legend.position = "none") + theme(plot.margin = margin(t=0, r=0, b=0, l=0, "pt"))

MK6240_PVC_individual_regions_std_Difference_p <- MK6240_PVC_individual_cortical_regions_std_Difference_p / MK6240_PVC_individual_subcortical_regions_std_Difference_p + plot_layout(heights = c(1.25,1))

MK6240_PVC_individual_regions_std_Difference_p

# std forest plot for MK6240_PVC

dat_forest_MK6240_PVC_std_by_Status <- data.frame(matrix(ncol = 1, nrow = 13))

dat_forest_MK6240_PVC_std_by_Status$model <- c("Prefrontal Cortex","Insula",

"Cingulate Gyrus","Fusiform Gyrus","Lingual Gyrus",

"Entorhinal Cortex",

"Middle Inferior Temporal Gyrus","Superior Temporal Gyrus",

"Inferior Parietal Cortex","Superior Parietal Cortex",

"Amygdala","Hippocampus","Striatum")

dat_forest_MK6240_PVC_std_by_Status$model <- factor(dat_forest_MK6240_PVC_by_Status$model,

levels=c("Prefrontal Cortex","Insula",

"Cingulate Gyrus","Fusiform Gyrus","Lingual Gyrus",

"Entorhinal Cortex",

"Middle Inferior Temporal Gyrus","Superior Temporal Gyrus",

"Inferior Parietal Cortex","Superior Parietal Cortex",

"Amygdala","Hippocampus","Striatum"))

dat_forest_MK6240_PVC_std_by_Status$estimate <- c(Tau_Status_lm_full3_std_mean_df[(Tau_Status_lm_full3_std_mean_df$roi == "Prefrontal" & Tau_Status_lm_full3_std_mean_df$contrast == "(Control - ADRD)"), "effect.size"],

Tau_Status_lm_full3_std_mean_df[(Tau_Status_lm_full3_std_mean_df$roi == "Insula" & Tau_Status_lm_full3_std_mean_df$contrast == "(Control - ADRD)"), "effect.size"],

Tau_Status_lm_full3_std_mean_df[(Tau_Status_lm_full3_std_mean_df$roi == "G_Cing" & Tau_Status_lm_full3_std_mean_df$contrast == "(Control - ADRD)"), "effect.size"],

Tau_Status_lm_full3_std_mean_df[(Tau_Status_lm_full3_std_mean_df$roi == "FusiformGyrus" & Tau_Status_lm_full3_std_mean_df$contrast == "(Control - ADRD)"), "effect.size"],

Tau_Status_lm_full3_std_mean_df[(Tau_Status_lm_full3_std_mean_df$roi == "LingualGyrus" & Tau_Status_lm_full3_std_mean_df$contrast == "(Control - ADRD)"), "effect.size"],

Tau_Status_lm_full3_std_mean_df[(Tau_Status_lm_full3_std_mean_df$roi == "EntorhinalCortex_FS" & Tau_Status_lm_full3_std_mean_df$contrast == "(Control - ADRD)"), "effect.size"],

Tau_Status_lm_full3_std_mean_df[(Tau_Status_lm_full3_std_mean_df$roi == "MiddleInfTempGyrus" & Tau_Status_lm_full3_std_mean_df$contrast == "(Control - ADRD)"), "effect.size"],

Tau_Status_lm_full3_std_mean_df[(Tau_Status_lm_full3_std_mean_df$roi == "SuperiorTemp" & Tau_Status_lm_full3_std_mean_df$contrast == "(Control - ADRD)"), "effect.size"],

Tau_Status_lm_full3_std_mean_df[(Tau_Status_lm_full3_std_mean_df$roi == "InferiorParietal" & Tau_Status_lm_full3_std_mean_df$contrast == "(Control - ADRD)"), "effect.size"],

Tau_Status_lm_full3_std_mean_df[(Tau_Status_lm_full3_std_mean_df$roi == "SuperiorParietal" & Tau_Status_lm_full3_std_mean_df$contrast == "(Control - ADRD)"), "effect.size"],

Tau_Status_lm_full3_std_mean_df[(Tau_Status_lm_full3_std_mean_df$roi == "Amygdala" & Tau_Status_lm_full3_std_mean_df$contrast == "(Control - ADRD)"), "effect.size"],

Tau_Status_lm_full3_std_mean_df[(Tau_Status_lm_full3_std_mean_df$roi == "Hippocampus" & Tau_Status_lm_full3_std_mean_df$contrast == "(Control - ADRD)"), "effect.size"],

Tau_Status_lm_full3_std_mean_df[(Tau_Status_lm_full3_std_mean_df$roi == "Striatum" & Tau_Status_lm_full3_std_mean_df$contrast == "(Control - ADRD)"), "effect.size"]) * -1

dat_forest_MK6240_PVC_std_by_Status$low_CI <- c(Tau_Status_lm_full3_std_mean_df[(Tau_Status_lm_full3_std_mean_df$roi == "Prefrontal" & Tau_Status_lm_full3_std_mean_df$contrast == "(Control - ADRD)"), "lower.CL"],

Tau_Status_lm_full3_std_mean_df[(Tau_Status_lm_full3_std_mean_df$roi == "Insula" & Tau_Status_lm_full3_std_mean_df$contrast == "(Control - ADRD)"), "lower.CL"],

Tau_Status_lm_full3_std_mean_df[(Tau_Status_lm_full3_std_mean_df$roi == "G_Cing" & Tau_Status_lm_full3_std_mean_df$contrast == "(Control - ADRD)"), "lower.CL"],

Tau_Status_lm_full3_std_mean_df[(Tau_Status_lm_full3_std_mean_df$roi == "FusiformGyrus" & Tau_Status_lm_full3_std_mean_df$contrast == "(Control - ADRD)"), "lower.CL"],

Tau_Status_lm_full3_std_mean_df[(Tau_Status_lm_full3_std_mean_df$roi == "LingualGyrus" & Tau_Status_lm_full3_std_mean_df$contrast == "(Control - ADRD)"), "lower.CL"],

Tau_Status_lm_full3_std_mean_df[(Tau_Status_lm_full3_std_mean_df$roi == "EntorhinalCortex_FS" & Tau_Status_lm_full3_std_mean_df$contrast == "(Control - ADRD)"), "lower.CL"],

Tau_Status_lm_full3_std_mean_df[(Tau_Status_lm_full3_std_mean_df$roi == "MiddleInfTempGyrus" & Tau_Status_lm_full3_std_mean_df$contrast == "(Control - ADRD)"), "lower.CL"],

Tau_Status_lm_full3_std_mean_df[(Tau_Status_lm_full3_std_mean_df$roi == "SuperiorTemp" & Tau_Status_lm_full3_std_mean_df$contrast == "(Control - ADRD)"), "lower.CL"],

Tau_Status_lm_full3_std_mean_df[(Tau_Status_lm_full3_std_mean_df$roi == "InferiorParietal" & Tau_Status_lm_full3_std_mean_df$contrast == "(Control - ADRD)"), "lower.CL"],

Tau_Status_lm_full3_std_mean_df[(Tau_Status_lm_full3_std_mean_df$roi == "SuperiorParietal" & Tau_Status_lm_full3_std_mean_df$contrast == "(Control - ADRD)"), "lower.CL"],

Tau_Status_lm_full3_std_mean_df[(Tau_Status_lm_full3_std_mean_df$roi == "Amygdala" & Tau_Status_lm_full3_std_mean_df$contrast == "(Control - ADRD)"), "lower.CL"],

Tau_Status_lm_full3_std_mean_df[(Tau_Status_lm_full3_std_mean_df$roi == "Hippocampus" & Tau_Status_lm_full3_std_mean_df$contrast == "(Control - ADRD)"), "lower.CL"],

Tau_Status_lm_full3_std_mean_df[(Tau_Status_lm_full3_std_mean_df$roi == "Striatum" & Tau_Status_lm_full3_std_mean_df$contrast == "(Control - ADRD)"), "lower.CL"]) * -1

dat_forest_MK6240_PVC_std_by_Status$high_CI <- c(Tau_Status_lm_full3_std_mean_df[(Tau_Status_lm_full3_std_mean_df$roi == "Prefrontal" & Tau_Status_lm_full3_std_mean_df$contrast == "(Control - ADRD)"), "upper.CL"],

Tau_Status_lm_full3_std_mean_df[(Tau_Status_lm_full3_std_mean_df$roi == "Insula" & Tau_Status_lm_full3_std_mean_df$contrast == "(Control - ADRD)"), "upper.CL"],

Tau_Status_lm_full3_std_mean_df[(Tau_Status_lm_full3_std_mean_df$roi == "G_Cing" & Tau_Status_lm_full3_std_mean_df$contrast == "(Control - ADRD)"), "upper.CL"],

Tau_Status_lm_full3_std_mean_df[(Tau_Status_lm_full3_std_mean_df$roi == "FusiformGyrus" & Tau_Status_lm_full3_std_mean_df$contrast == "(Control - ADRD)"), "upper.CL"],

Tau_Status_lm_full3_std_mean_df[(Tau_Status_lm_full3_std_mean_df$roi == "LingualGyrus" & Tau_Status_lm_full3_std_mean_df$contrast == "(Control - ADRD)"), "upper.CL"],

Tau_Status_lm_full3_std_mean_df[(Tau_Status_lm_full3_std_mean_df$roi == "EntorhinalCortex_FS" & Tau_Status_lm_full3_std_mean_df$contrast == "(Control - ADRD)"), "upper.CL"],

Tau_Status_lm_full3_std_mean_df[(Tau_Status_lm_full3_std_mean_df$roi == "MiddleInfTempGyrus" & Tau_Status_lm_full3_std_mean_df$contrast == "(Control - ADRD)"), "upper.CL"],

Tau_Status_lm_full3_std_mean_df[(Tau_Status_lm_full3_std_mean_df$roi == "SuperiorTemp" & Tau_Status_lm_full3_std_mean_df$contrast == "(Control - ADRD)"), "upper.CL"],

Tau_Status_lm_full3_std_mean_df[(Tau_Status_lm_full3_std_mean_df$roi == "InferiorParietal" & Tau_Status_lm_full3_std_mean_df$contrast == "(Control - ADRD)"), "upper.CL"],

Tau_Status_lm_full3_std_mean_df[(Tau_Status_lm_full3_std_mean_df$roi == "SuperiorParietal" & Tau_Status_lm_full3_std_mean_df$contrast == "(Control - ADRD)"), "upper.CL"],

Tau_Status_lm_full3_std_mean_df[(Tau_Status_lm_full3_std_mean_df$roi == "Amygdala" & Tau_Status_lm_full3_std_mean_df$contrast == "(Control - ADRD)"), "upper.CL"],

Tau_Status_lm_full3_std_mean_df[(Tau_Status_lm_full3_std_mean_df$roi == "Hippocampus" & Tau_Status_lm_full3_std_mean_df$contrast == "(Control - ADRD)"), "upper.CL"],

Tau_Status_lm_full3_std_mean_df[(Tau_Status_lm_full3_std_mean_df$roi == "Striatum" & Tau_Status_lm_full3_std_mean_df$contrast == "(Control - ADRD)"), "upper.CL"]) * -1

p_MK6240_PVC_std_by_Status <-

dat_forest_MK6240_PVC_std_by_Status |>

ggplot(aes(y = fct_rev(model))) +

theme_bw() +

geom_point(aes(x=estimate), shape=12, size=3) +

geom_linerange(aes(xmin=low_CI, xmax=high_CI)) +

geom_vline(xintercept = 0, linetype="dashed") +

labs(x="ADRD - Control", y="") +

coord_cartesian(ylim=c(1,13), xlim=c(-0.75, 3)) +

theme(text = element_text(size = 20), axis.text = element_text(color="black")) +

theme(axis.line = element_line(colour = "black"),

panel.grid.major = element_blank(),

panel.grid.minor = element_blank(),

panel.border = element_blank(),

panel.background = element_blank())

p_MK6240_PVC_std_by_Status

## Amyloid

analysis.dat.long_Amyloid <- analysis.dat.long[complete.cases(analysis.dat.long[c("FBB", "Status_baseline", "roi",

"Age_baseline","Sex","BMI_baseline","APOE4")]),]

unique(analysis.dat.long_Amyloid$ID)

Amyloid_Status_lm_full3<-lm(FBB ~ Status_baseline*roi + Age_baseline + Sex + BMI_baseline + APOE4,

analysis.dat.long %>% filter(roi %in% roilist))

Amyloid_Status_lm_full3_summary<-summary(Amyloid_Status_lm_full3)

Amyloid_Status_lm_full3_mean <- emmeans(Amyloid_Status_lm_full3, spec = pairwise ~ Status_baseline | roi,

weights="proportional",adjust="mvt") %>% summary(infer=TRUE)

Amyloid_Status_lm_full3_mean_df <- as.data.frame(Amyloid_Status_lm_full3_mean)

### amyloid std

pwpm(emmeans(Amyloid_Status_lm_full3, spec = pairwise ~ Status_baseline | roi,

weights="proportional",adjust="mvt"))

Amyloid_Status_lm_full3_std_mean <- as.data.frame(eff_size(emmeans(Amyloid_Status_lm_full3, spec = pairwise ~ Status_baseline | roi,

weights="proportional",adjust="mvt"), sigma = sigma(Amyloid_Status_lm_full3), edf = Amyloid_Status_lm_full3_summary[[7]][[2]]))

Amyloid_Status_lm_full3_std_mean_df <- as.data.frame(Amyloid_Status_lm_full3_std_mean)

# std ADRD - Control

FBB_individual_cortical_regions_std_Difference = data.frame(

region = c("medial orbitofrontal", "lateral orbitofrontal","pars opercularis", "pars orbitalis", "pars triangularis", "frontal pole", "rostral middle frontal", "caudal middle frontal",

"entorhinal",

"inferior temporal", "middle temporal",

"superior temporal", "bankssts", "transverse temporal",

"caudal anterior cingulate", "rostral anterior cingulate","isthmus cingulate", "posterior cingulate",

"superior parietal",

"inferior parietal",

"fusiform",

"lingual",

"insula"),

mean = -1 * c(rep(Amyloid_Status_lm_full3_std_mean_df[(Amyloid_Status_lm_full3_std_mean_df$roi == "Prefrontal" & Amyloid_Status_lm_full3_std_mean_df$contrast == "(Control - ADRD)"), "effect.size"],8),

rep(Amyloid_Status_lm_full3_std_mean_df[(Amyloid_Status_lm_full3_std_mean_df$roi == "EntorhinalCortex_FS" & Amyloid_Status_lm_full3_std_mean_df$contrast == "(Control - ADRD)"), "effect.size"],1),

rep(Amyloid_Status_lm_full3_std_mean_df[(Amyloid_Status_lm_full3_std_mean_df$roi == "MiddleInfTempGyrus" & Amyloid_Status_lm_full3_std_mean_df$contrast == "(Control - ADRD)"), "effect.size"],2),

rep(Amyloid_Status_lm_full3_std_mean_df[(Amyloid_Status_lm_full3_std_mean_df$roi == "SuperiorTemp" & Amyloid_Status_lm_full3_std_mean_df$contrast == "(Control - ADRD)"), "effect.size"],3),

rep(Amyloid_Status_lm_full3_std_mean_df[(Amyloid_Status_lm_full3_std_mean_df$roi == "G_Cing" & Amyloid_Status_lm_full3_std_mean_df$contrast == "(Control - ADRD)"), "effect.size"],4),

rep(Amyloid_Status_lm_full3_std_mean_df[(Amyloid_Status_lm_full3_std_mean_df$roi == "SuperiorParietal" & Amyloid_Status_lm_full3_std_mean_df$contrast == "(Control - ADRD)"), "effect.size"],1),

rep(Amyloid_Status_lm_full3_std_mean_df[(Amyloid_Status_lm_full3_std_mean_df$roi == "InferiorParietal" & Amyloid_Status_lm_full3_std_mean_df$contrast == "(Control - ADRD)"), "effect.size"],1),

rep(Amyloid_Status_lm_full3_std_mean_df[(Amyloid_Status_lm_full3_std_mean_df$roi == "FusiformGyrus" & Amyloid_Status_lm_full3_std_mean_df$contrast == "(Control - ADRD)"), "effect.size"],1),

rep(Amyloid_Status_lm_full3_std_mean_df[(Amyloid_Status_lm_full3_std_mean_df$roi == "LingualGyrus" & Amyloid_Status_lm_full3_std_mean_df$contrast == "(Control - ADRD)"), "effect.size"],1),

rep(Amyloid_Status_lm_full3_std_mean_df[(Amyloid_Status_lm_full3_std_mean_df$roi == "Insula" & Amyloid_Status_lm_full3_std_mean_df$contrast == "(Control - ADRD)"), "effect.size"],1)),

stringsAsFactors = FALSE)

FBB_individual_cortical_regions_std_Difference_p <- ggseg(.data=FBB_individual_cortical_regions_std_Difference, colour="white", mapping=aes(fill=mean), position="stacked", hemi="left") +

theme_void() +

ggtitle(sprintf("Amyloid")) +

theme(plot.title = element_text(hjust = 0.5)) +

theme(text = element_text(size = 30)) +

scale_fill_distiller(palette = "Spectral", direction=-1,

breaks=c(-3,0,3),limits=c(-3,3),

name="") + theme(legend.position = "bottom") + theme(plot.margin = margin(t=0, r=0, b=0, l=0, "pt"))

FBB_individual_subcortical_regions_std_Difference = data.frame(

region = c("hippocampus",

"amygdala",

"caudate","putamen"),

mean = -1 * c(rep(Amyloid_Status_lm_full3_std_mean_df[(Amyloid_Status_lm_full3_std_mean_df$roi == "Hippocampus" & Amyloid_Status_lm_full3_std_mean_df$contrast == "(Control - ADRD)"), "effect.size"],1),

rep(Amyloid_Status_lm_full3_std_mean_df[(Amyloid_Status_lm_full3_std_mean_df$roi == "Amygdala" & Amyloid_Status_lm_full3_std_mean_df$contrast == "(Control - ADRD)"), "effect.size"],1),

rep(Amyloid_Status_lm_full3_std_mean_df[(Amyloid_Status_lm_full3_std_mean_df$roi == "Striatum" & Amyloid_Status_lm_full3_std_mean_df$contrast == "(Control - ADRD)"), "effect.size"],2)),

Status = c(rep("Controls",4),rep("ADRD",4)),

stringsAsFactors = FALSE)

FBB_individual_subcortical_regions_std_Difference_p <- ggplot(FBB_individual_subcortical_regions_std_Difference) +

geom_brain(atlas=aseg, side="coronal", colour="white", mapping=aes(fill=mean)) +

theme_void() +

scale_fill_distiller(palette = "Spectral", direction=-1,

breaks=c(-3,0,3),limits=c(-3,3),

name="") + theme(legend.position = "none") + theme(plot.margin = margin(t=0, r=0, b=0, l=0, "pt"))

FBB_individual_regions_std_Difference_p <- FBB_individual_cortical_regions_std_Difference_p / FBB_individual_subcortical_regions_std_Difference_p + plot_layout(heights = c(1.25,1))

FBB_individual_regions_std_Difference_p

# std forest plot for FBB

dat_forest_FBB_std_by_Status <- data.frame(matrix(ncol = 1, nrow = 13))

dat_forest_FBB_std_by_Status$model <- c("Prefrontal Cortex","Insula",

"Cingulate Gyrus","Fusiform Gyrus","Lingual Gyrus",

"Entorhinal Cortex",

"Middle Inferior Temporal Gyrus","Superior Temporal Gyrus",

"Inferior Parietal Cortex","Superior Parietal Cortex",

"Amygdala","Hippocampus","Striatum")

dat_forest_FBB_std_by_Status$model <- factor(dat_forest_FBB_by_Status$model,

levels=c("Prefrontal Cortex","Insula",

"Cingulate Gyrus","Fusiform Gyrus","Lingual Gyrus",

"Entorhinal Cortex",

"Middle Inferior Temporal Gyrus","Superior Temporal Gyrus",

"Inferior Parietal Cortex","Superior Parietal Cortex",

"Amygdala","Hippocampus","Striatum"))

dat_forest_FBB_std_by_Status$estimate <- c(Amyloid_Status_lm_full3_std_mean_df[(Amyloid_Status_lm_full3_std_mean_df$roi == "Prefrontal" & Amyloid_Status_lm_full3_std_mean_df$contrast == "(Control - ADRD)"), "effect.size"],

Amyloid_Status_lm_full3_std_mean_df[(Amyloid_Status_lm_full3_std_mean_df$roi == "Insula" & Amyloid_Status_lm_full3_std_mean_df$contrast == "(Control - ADRD)"), "effect.size"],

Amyloid_Status_lm_full3_std_mean_df[(Amyloid_Status_lm_full3_std_mean_df$roi == "G_Cing" & Amyloid_Status_lm_full3_std_mean_df$contrast == "(Control - ADRD)"), "effect.size"],

Amyloid_Status_lm_full3_std_mean_df[(Amyloid_Status_lm_full3_std_mean_df$roi == "FusiformGyrus" & Amyloid_Status_lm_full3_std_mean_df$contrast == "(Control - ADRD)"), "effect.size"],

Amyloid_Status_lm_full3_std_mean_df[(Amyloid_Status_lm_full3_std_mean_df$roi == "LingualGyrus" & Amyloid_Status_lm_full3_std_mean_df$contrast == "(Control - ADRD)"), "effect.size"],

Amyloid_Status_lm_full3_std_mean_df[(Amyloid_Status_lm_full3_std_mean_df$roi == "EntorhinalCortex_FS" & Amyloid_Status_lm_full3_std_mean_df$contrast == "(Control - ADRD)"), "effect.size"],

Amyloid_Status_lm_full3_std_mean_df[(Amyloid_Status_lm_full3_std_mean_df$roi == "MiddleInfTempGyrus" & Amyloid_Status_lm_full3_std_mean_df$contrast == "(Control - ADRD)"), "effect.size"],

Amyloid_Status_lm_full3_std_mean_df[(Amyloid_Status_lm_full3_std_mean_df$roi == "SuperiorTemp" & Amyloid_Status_lm_full3_std_mean_df$contrast == "(Control - ADRD)"), "effect.size"],

Amyloid_Status_lm_full3_std_mean_df[(Amyloid_Status_lm_full3_std_mean_df$roi == "InferiorParietal" & Amyloid_Status_lm_full3_std_mean_df$contrast == "(Control - ADRD)"), "effect.size"],

Amyloid_Status_lm_full3_std_mean_df[(Amyloid_Status_lm_full3_std_mean_df$roi == "SuperiorParietal" & Amyloid_Status_lm_full3_std_mean_df$contrast == "(Control - ADRD)"), "effect.size"],

Amyloid_Status_lm_full3_std_mean_df[(Amyloid_Status_lm_full3_std_mean_df$roi == "Amygdala" & Amyloid_Status_lm_full3_std_mean_df$contrast == "(Control - ADRD)"), "effect.size"],

Amyloid_Status_lm_full3_std_mean_df[(Amyloid_Status_lm_full3_std_mean_df$roi == "Hippocampus" & Amyloid_Status_lm_full3_std_mean_df$contrast == "(Control - ADRD)"), "effect.size"],

Amyloid_Status_lm_full3_std_mean_df[(Amyloid_Status_lm_full3_std_mean_df$roi == "Striatum" & Amyloid_Status_lm_full3_std_mean_df$contrast == "(Control - ADRD)"), "effect.size"]) * -1

dat_forest_FBB_std_by_Status$low_CI <- c(Amyloid_Status_lm_full3_std_mean_df[(Amyloid_Status_lm_full3_std_mean_df$roi == "Prefrontal" & Amyloid_Status_lm_full3_std_mean_df$contrast == "(Control - ADRD)"), "lower.CL"],

Amyloid_Status_lm_full3_std_mean_df[(Amyloid_Status_lm_full3_std_mean_df$roi == "Insula" & Amyloid_Status_lm_full3_std_mean_df$contrast == "(Control - ADRD)"), "lower.CL"],

Amyloid_Status_lm_full3_std_mean_df[(Amyloid_Status_lm_full3_std_mean_df$roi == "G_Cing" & Amyloid_Status_lm_full3_std_mean_df$contrast == "(Control - ADRD)"), "lower.CL"],

Amyloid_Status_lm_full3_std_mean_df[(Amyloid_Status_lm_full3_std_mean_df$roi == "FusiformGyrus" & Amyloid_Status_lm_full3_std_mean_df$contrast == "(Control - ADRD)"), "lower.CL"],

Amyloid_Status_lm_full3_std_mean_df[(Amyloid_Status_lm_full3_std_mean_df$roi == "LingualGyrus" & Amyloid_Status_lm_full3_std_mean_df$contrast == "(Control - ADRD)"), "lower.CL"],

Amyloid_Status_lm_full3_std_mean_df[(Amyloid_Status_lm_full3_std_mean_df$roi == "EntorhinalCortex_FS" & Amyloid_Status_lm_full3_std_mean_df$contrast == "(Control - ADRD)"), "lower.CL"],

Amyloid_Status_lm_full3_std_mean_df[(Amyloid_Status_lm_full3_std_mean_df$roi == "MiddleInfTempGyrus" & Amyloid_Status_lm_full3_std_mean_df$contrast == "(Control - ADRD)"), "lower.CL"],

Amyloid_Status_lm_full3_std_mean_df[(Amyloid_Status_lm_full3_std_mean_df$roi == "SuperiorTemp" & Amyloid_Status_lm_full3_std_mean_df$contrast == "(Control - ADRD)"), "lower.CL"],

Amyloid_Status_lm_full3_std_mean_df[(Amyloid_Status_lm_full3_std_mean_df$roi == "InferiorParietal" & Amyloid_Status_lm_full3_std_mean_df$contrast == "(Control - ADRD)"), "lower.CL"],

Amyloid_Status_lm_full3_std_mean_df[(Amyloid_Status_lm_full3_std_mean_df$roi == "SuperiorParietal" & Amyloid_Status_lm_full3_std_mean_df$contrast == "(Control - ADRD)"), "lower.CL"],

Amyloid_Status_lm_full3_std_mean_df[(Amyloid_Status_lm_full3_std_mean_df$roi == "Amygdala" & Amyloid_Status_lm_full3_std_mean_df$contrast == "(Control - ADRD)"), "lower.CL"],

Amyloid_Status_lm_full3_std_mean_df[(Amyloid_Status_lm_full3_std_mean_df$roi == "Hippocampus" & Amyloid_Status_lm_full3_std_mean_df$contrast == "(Control - ADRD)"), "lower.CL"],

Amyloid_Status_lm_full3_std_mean_df[(Amyloid_Status_lm_full3_std_mean_df$roi == "Striatum" & Amyloid_Status_lm_full3_std_mean_df$contrast == "(Control - ADRD)"), "lower.CL"]) * -1

dat_forest_FBB_std_by_Status$high_CI <- c(Amyloid_Status_lm_full3_std_mean_df[(Amyloid_Status_lm_full3_std_mean_df$roi == "Prefrontal" & Amyloid_Status_lm_full3_std_mean_df$contrast == "(Control - ADRD)"), "upper.CL"],

Amyloid_Status_lm_full3_std_mean_df[(Amyloid_Status_lm_full3_std_mean_df$roi == "Insula" & Amyloid_Status_lm_full3_std_mean_df$contrast == "(Control - ADRD)"), "upper.CL"],

Amyloid_Status_lm_full3_std_mean_df[(Amyloid_Status_lm_full3_std_mean_df$roi == "G_Cing" & Amyloid_Status_lm_full3_std_mean_df$contrast == "(Control - ADRD)"), "upper.CL"],

Amyloid_Status_lm_full3_std_mean_df[(Amyloid_Status_lm_full3_std_mean_df$roi == "FusiformGyrus" & Amyloid_Status_lm_full3_std_mean_df$contrast == "(Control - ADRD)"), "upper.CL"],

Amyloid_Status_lm_full3_std_mean_df[(Amyloid_Status_lm_full3_std_mean_df$roi == "LingualGyrus" & Amyloid_Status_lm_full3_std_mean_df$contrast == "(Control - ADRD)"), "upper.CL"],

Amyloid_Status_lm_full3_std_mean_df[(Amyloid_Status_lm_full3_std_mean_df$roi == "EntorhinalCortex_FS" & Amyloid_Status_lm_full3_std_mean_df$contrast == "(Control - ADRD)"), "upper.CL"],

Amyloid_Status_lm_full3_std_mean_df[(Amyloid_Status_lm_full3_std_mean_df$roi == "MiddleInfTempGyrus" & Amyloid_Status_lm_full3_std_mean_df$contrast == "(Control - ADRD)"), "upper.CL"],

Amyloid_Status_lm_full3_std_mean_df[(Amyloid_Status_lm_full3_std_mean_df$roi == "SuperiorTemp" & Amyloid_Status_lm_full3_std_mean_df$contrast == "(Control - ADRD)"), "upper.CL"],

Amyloid_Status_lm_full3_std_mean_df[(Amyloid_Status_lm_full3_std_mean_df$roi == "InferiorParietal" & Amyloid_Status_lm_full3_std_mean_df$contrast == "(Control - ADRD)"), "upper.CL"],

Amyloid_Status_lm_full3_std_mean_df[(Amyloid_Status_lm_full3_std_mean_df$roi == "SuperiorParietal" & Amyloid_Status_lm_full3_std_mean_df$contrast == "(Control - ADRD)"), "upper.CL"],

Amyloid_Status_lm_full3_std_mean_df[(Amyloid_Status_lm_full3_std_mean_df$roi == "Amygdala" & Amyloid_Status_lm_full3_std_mean_df$contrast == "(Control - ADRD)"), "upper.CL"],

Amyloid_Status_lm_full3_std_mean_df[(Amyloid_Status_lm_full3_std_mean_df$roi == "Hippocampus" & Amyloid_Status_lm_full3_std_mean_df$contrast == "(Control - ADRD)"), "upper.CL"],

Amyloid_Status_lm_full3_std_mean_df[(Amyloid_Status_lm_full3_std_mean_df$roi == "Striatum" & Amyloid_Status_lm_full3_std_mean_df$contrast == "(Control - ADRD)"), "upper.CL"]) * -1

p_FBB_std_by_Status <-

dat_forest_FBB_std_by_Status |>

ggplot(aes(y = fct_rev(model))) +

theme_bw() +

geom_point(aes(x=estimate), shape=12, size=3) +

geom_linerange(aes(xmin=low_CI, xmax=high_CI)) +

geom_vline(xintercept = 0, linetype="dashed") +

labs(x="ADRD - Control", y="") +

coord_cartesian(ylim=c(1,13), xlim=c(-0.75, 3)) +

theme(text = element_text(size = 20), axis.text = element_text(color="black")) +

theme(axis.line = element_line(colour = "black"),

panel.grid.major = element_blank(),

panel.grid.minor = element_blank(),

panel.border = element_blank(),

panel.background = element_blank())

p_FBB_std_by_Status

## Volume

analysis.dat.long_Volume <- analysis.dat.long[complete.cases(analysis.dat.long[c("Volume_MK6240", "Status_baseline", "roi",

"ICV_baseline","Age_baseline","Sex","BMI_baseline","APOE4")]),]

unique(analysis.dat.long_Volume$ID)

Volume_Status_lm_full3<-lm(Volume_MK6240 ~ Status_baseline*roi + ICV_baseline + Age_baseline + Sex + BMI_baseline + APOE4,

analysis.dat.long %>% filter(roi %in% roilist))

Volume_Status_lm_full3_summary<-summary(Volume_Status_lm_full3)

Volume_Status_lm_full3_mean <- emmeans(Volume_Status_lm_full3, spec = pairwise ~ Status_baseline | roi,

weights="proportional",adjust="mvt") %>% summary(infer=TRUE)

Volume_Status_lm_full3_mean_df <- as.data.frame(Volume_Status_lm_full3_mean)

### volume std

pwpm(emmeans(Volume_Status_lm_full3, spec = pairwise ~ Status_baseline | roi,

weights="proportional",adjust="mvt"))

Volume_Status_lm_full3_std_mean <- as.data.frame(eff_size(emmeans(Volume_Status_lm_full3, spec = pairwise ~ Status_baseline | roi,

weights="proportional",adjust="mvt"), sigma = sigma(Volume_Status_lm_full3), edf = Volume_Status_lm_full3_summary[[7]][[2]]))

Volume_Status_lm_full3_std_mean_df <- as.data.frame(Volume_Status_lm_full3_std_mean)

# std ADRD - Control (flipped)

Volume_MK6240_individual_cortical_regions_std_Difference = data.frame(

region = c("medial orbitofrontal", "lateral orbitofrontal","pars opercularis", "pars orbitalis", "pars triangularis", "frontal pole", "rostral middle frontal", "caudal middle frontal",

"entorhinal",

"inferior temporal", "middle temporal",

"superior temporal", "bankssts", "transverse temporal",

"caudal anterior cingulate", "rostral anterior cingulate","isthmus cingulate", "posterior cingulate",

"superior parietal",

"inferior parietal",

"fusiform",

"lingual",

"insula"),

mean = c(rep(Volume_Status_lm_full3_std_mean_df[(Volume_Status_lm_full3_std_mean_df$roi == "Prefrontal" & Volume_Status_lm_full3_std_mean_df$contrast == "(Control - ADRD)"), "effect.size"],8),

rep(Volume_Status_lm_full3_std_mean_df[(Volume_Status_lm_full3_std_mean_df$roi == "EntorhinalCortex_FS" & Volume_Status_lm_full3_std_mean_df$contrast == "(Control - ADRD)"), "effect.size"],1),

rep(Volume_Status_lm_full3_std_mean_df[(Volume_Status_lm_full3_std_mean_df$roi == "MiddleInfTempGyrus" & Volume_Status_lm_full3_std_mean_df$contrast == "(Control - ADRD)"), "effect.size"],2),

rep(Volume_Status_lm_full3_std_mean_df[(Volume_Status_lm_full3_std_mean_df$roi == "SuperiorTemp" & Volume_Status_lm_full3_std_mean_df$contrast == "(Control - ADRD)"), "effect.size"],3),

rep(Volume_Status_lm_full3_std_mean_df[(Volume_Status_lm_full3_std_mean_df$roi == "G_Cing" & Volume_Status_lm_full3_std_mean_df$contrast == "(Control - ADRD)"), "effect.size"],4),

rep(Volume_Status_lm_full3_std_mean_df[(Volume_Status_lm_full3_std_mean_df$roi == "SuperiorParietal" & Volume_Status_lm_full3_std_mean_df$contrast == "(Control - ADRD)"), "effect.size"],1),

rep(Volume_Status_lm_full3_std_mean_df[(Volume_Status_lm_full3_std_mean_df$roi == "InferiorParietal" & Volume_Status_lm_full3_std_mean_df$contrast == "(Control - ADRD)"), "effect.size"],1),

rep(Volume_Status_lm_full3_std_mean_df[(Volume_Status_lm_full3_std_mean_df$roi == "FusiformGyrus" & Volume_Status_lm_full3_std_mean_df$contrast == "(Control - ADRD)"), "effect.size"],1),

rep(Volume_Status_lm_full3_std_mean_df[(Volume_Status_lm_full3_std_mean_df$roi == "LingualGyrus" & Volume_Status_lm_full3_std_mean_df$contrast == "(Control - ADRD)"), "effect.size"],1),

rep(Volume_Status_lm_full3_std_mean_df[(Volume_Status_lm_full3_std_mean_df$roi == "Insula" & Volume_Status_lm_full3_std_mean_df$contrast == "(Control - ADRD)"), "effect.size"],1)),

stringsAsFactors = FALSE)

Volume_MK6240_individual_cortical_regions_std_Difference_p <- ggseg(.data=Volume_MK6240_individual_cortical_regions_std_Difference, colour="white", mapping=aes(fill=mean), position="stacked", hemi="left") +

theme_void() +

ggtitle(sprintf("Neurodegeneration")) +

theme(plot.title = element_text(hjust = 0.5)) +

theme(text = element_text(size = 30)) +

scale_fill_distiller(palette = "Spectral", direction=-1,

breaks=c(-3,0,3),limits=c(-3,3),

name="") + theme(legend.position = "bottom") + theme(plot.margin = margin(t=0, r=0, b=0, l=0, "pt"))

Volume_MK6240_individual_subcortical_regions_std_Difference = data.frame(

region = c("hippocampus",

"amygdala",

"caudate","putamen"),

mean = c(rep(Volume_Status_lm_full3_std_mean_df[(Volume_Status_lm_full3_std_mean_df$roi == "Hippocampus" & Volume_Status_lm_full3_std_mean_df$contrast == "(Control - ADRD)"), "effect.size"],1),

rep(Volume_Status_lm_full3_std_mean_df[(Volume_Status_lm_full3_std_mean_df$roi == "Amygdala" & Volume_Status_lm_full3_std_mean_df$contrast == "(Control - ADRD)"), "effect.size"],1),

rep(Volume_Status_lm_full3_std_mean_df[(Volume_Status_lm_full3_std_mean_df$roi == "Striatum" & Volume_Status_lm_full3_std_mean_df$contrast == "(Control - ADRD)"), "effect.size"],2)),

Status = c(rep("Controls",4),rep("ADRD",4)),

stringsAsFactors = FALSE)

Volume_MK6240_individual_subcortical_regions_std_Difference_p <- ggplot(Volume_MK6240_individual_subcortical_regions_std_Difference) +

geom_brain(atlas=aseg, side="coronal", colour="white", mapping=aes(fill=mean)) +

theme_void() +

scale_fill_distiller(palette = "Spectral", direction=-1,

breaks=c(-3,0,3),limits=c(-3,3),

name="") + theme(legend.position = "none") + theme(plot.margin = margin(t=0, r=0, b=0, l=0, "pt"))

Volume_MK6240_individual_regions_std_Difference_p <- Volume_MK6240_individual_cortical_regions_std_Difference_p / Volume_MK6240_individual_subcortical_regions_std_Difference_p + plot_layout(heights = c(1.25,1))

Volume_MK6240_individual_regions_std_Difference_p

# std forest plot for Volume_MK6240 (flipped)

dat_forest_Volume_MK6240_std_by_Status <- data.frame(matrix(ncol = 1, nrow = 13))

dat_forest_Volume_MK6240_std_by_Status$model <- c("Prefrontal Cortex","Insula",

"Cingulate Gyrus","Fusiform Gyrus","Lingual Gyrus",

"Entorhinal Cortex",

"Middle Inferior Temporal Gyrus","Superior Temporal Gyrus",

"Inferior Parietal Cortex","Superior Parietal Cortex",

"Amygdala","Hippocampus","Striatum")

dat_forest_Volume_MK6240_std_by_Status$model <- factor(dat_forest_Volume_MK6240_by_Status$model,

levels=c("Prefrontal Cortex","Insula",

"Cingulate Gyrus","Fusiform Gyrus","Lingual Gyrus",

"Entorhinal Cortex",

"Middle Inferior Temporal Gyrus","Superior Temporal Gyrus",

"Inferior Parietal Cortex","Superior Parietal Cortex",

"Amygdala","Hippocampus","Striatum"))

dat_forest_Volume_MK6240_std_by_Status$estimate <- c(Volume_Status_lm_full3_std_mean_df[(Volume_Status_lm_full3_std_mean_df$roi == "Prefrontal" & Volume_Status_lm_full3_std_mean_df$contrast == "(Control - ADRD)"), "effect.size"],

Volume_Status_lm_full3_std_mean_df[(Volume_Status_lm_full3_std_mean_df$roi == "Insula" & Volume_Status_lm_full3_std_mean_df$contrast == "(Control - ADRD)"), "effect.size"],

Volume_Status_lm_full3_std_mean_df[(Volume_Status_lm_full3_std_mean_df$roi == "G_Cing" & Volume_Status_lm_full3_std_mean_df$contrast == "(Control - ADRD)"), "effect.size"],

Volume_Status_lm_full3_std_mean_df[(Volume_Status_lm_full3_std_mean_df$roi == "FusiformGyrus" & Volume_Status_lm_full3_std_mean_df$contrast == "(Control - ADRD)"), "effect.size"],

Volume_Status_lm_full3_std_mean_df[(Volume_Status_lm_full3_std_mean_df$roi == "LingualGyrus" & Volume_Status_lm_full3_std_mean_df$contrast == "(Control - ADRD)"), "effect.size"],

Volume_Status_lm_full3_std_mean_df[(Volume_Status_lm_full3_std_mean_df$roi == "EntorhinalCortex_FS" & Volume_Status_lm_full3_std_mean_df$contrast == "(Control - ADRD)"), "effect.size"],

Volume_Status_lm_full3_std_mean_df[(Volume_Status_lm_full3_std_mean_df$roi == "MiddleInfTempGyrus" & Volume_Status_lm_full3_std_mean_df$contrast == "(Control - ADRD)"), "effect.size"],

Volume_Status_lm_full3_std_mean_df[(Volume_Status_lm_full3_std_mean_df$roi == "SuperiorTemp" & Volume_Status_lm_full3_std_mean_df$contrast == "(Control - ADRD)"), "effect.size"],

Volume_Status_lm_full3_std_mean_df[(Volume_Status_lm_full3_std_mean_df$roi == "InferiorParietal" & Volume_Status_lm_full3_std_mean_df$contrast == "(Control - ADRD)"), "effect.size"],

Volume_Status_lm_full3_std_mean_df[(Volume_Status_lm_full3_std_mean_df$roi == "SuperiorParietal" & Volume_Status_lm_full3_std_mean_df$contrast == "(Control - ADRD)"), "effect.size"],

Volume_Status_lm_full3_std_mean_df[(Volume_Status_lm_full3_std_mean_df$roi == "Amygdala" & Volume_Status_lm_full3_std_mean_df$contrast == "(Control - ADRD)"), "effect.size"],

Volume_Status_lm_full3_std_mean_df[(Volume_Status_lm_full3_std_mean_df$roi == "Hippocampus" & Volume_Status_lm_full3_std_mean_df$contrast == "(Control - ADRD)"), "effect.size"],

Volume_Status_lm_full3_std_mean_df[(Volume_Status_lm_full3_std_mean_df$roi == "Striatum" & Volume_Status_lm_full3_std_mean_df$contrast == "(Control - ADRD)"), "effect.size"])

dat_forest_Volume_MK6240_std_by_Status$low_CI <- c(Volume_Status_lm_full3_std_mean_df[(Volume_Status_lm_full3_std_mean_df$roi == "Prefrontal" & Volume_Status_lm_full3_std_mean_df$contrast == "(Control - ADRD)"), "lower.CL"],

Volume_Status_lm_full3_std_mean_df[(Volume_Status_lm_full3_std_mean_df$roi == "Insula" & Volume_Status_lm_full3_std_mean_df$contrast == "(Control - ADRD)"), "lower.CL"],

Volume_Status_lm_full3_std_mean_df[(Volume_Status_lm_full3_std_mean_df$roi == "G_Cing" & Volume_Status_lm_full3_std_mean_df$contrast == "(Control - ADRD)"), "lower.CL"],

Volume_Status_lm_full3_std_mean_df[(Volume_Status_lm_full3_std_mean_df$roi == "FusiformGyrus" & Volume_Status_lm_full3_std_mean_df$contrast == "(Control - ADRD)"), "lower.CL"],

Volume_Status_lm_full3_std_mean_df[(Volume_Status_lm_full3_std_mean_df$roi == "LingualGyrus" & Volume_Status_lm_full3_std_mean_df$contrast == "(Control - ADRD)"), "lower.CL"],

Volume_Status_lm_full3_std_mean_df[(Volume_Status_lm_full3_std_mean_df$roi == "EntorhinalCortex_FS" & Volume_Status_lm_full3_std_mean_df$contrast == "(Control - ADRD)"), "lower.CL"],

Volume_Status_lm_full3_std_mean_df[(Volume_Status_lm_full3_std_mean_df$roi == "MiddleInfTempGyrus" & Volume_Status_lm_full3_std_mean_df$contrast == "(Control - ADRD)"), "lower.CL"],

Volume_Status_lm_full3_std_mean_df[(Volume_Status_lm_full3_std_mean_df$roi == "SuperiorTemp" & Volume_Status_lm_full3_std_mean_df$contrast == "(Control - ADRD)"), "lower.CL"],

Volume_Status_lm_full3_std_mean_df[(Volume_Status_lm_full3_std_mean_df$roi == "InferiorParietal" & Volume_Status_lm_full3_std_mean_df$contrast == "(Control - ADRD)"), "lower.CL"],

Volume_Status_lm_full3_std_mean_df[(Volume_Status_lm_full3_std_mean_df$roi == "SuperiorParietal" & Volume_Status_lm_full3_std_mean_df$contrast == "(Control - ADRD)"), "lower.CL"],

Volume_Status_lm_full3_std_mean_df[(Volume_Status_lm_full3_std_mean_df$roi == "Amygdala" & Volume_Status_lm_full3_std_mean_df$contrast == "(Control - ADRD)"), "lower.CL"],

Volume_Status_lm_full3_std_mean_df[(Volume_Status_lm_full3_std_mean_df$roi == "Hippocampus" & Volume_Status_lm_full3_std_mean_df$contrast == "(Control - ADRD)"), "lower.CL"],

Volume_Status_lm_full3_std_mean_df[(Volume_Status_lm_full3_std_mean_df$roi == "Striatum" & Volume_Status_lm_full3_std_mean_df$contrast == "(Control - ADRD)"), "lower.CL"])

dat_forest_Volume_MK6240_std_by_Status$high_CI <- c(Volume_Status_lm_full3_std_mean_df[(Volume_Status_lm_full3_std_mean_df$roi == "Prefrontal" & Volume_Status_lm_full3_std_mean_df$contrast == "(Control - ADRD)"), "upper.CL"],

Volume_Status_lm_full3_std_mean_df[(Volume_Status_lm_full3_std_mean_df$roi == "Insula" & Volume_Status_lm_full3_std_mean_df$contrast == "(Control - ADRD)"), "upper.CL"],

Volume_Status_lm_full3_std_mean_df[(Volume_Status_lm_full3_std_mean_df$roi == "G_Cing" & Volume_Status_lm_full3_std_mean_df$contrast == "(Control - ADRD)"), "upper.CL"],

Volume_Status_lm_full3_std_mean_df[(Volume_Status_lm_full3_std_mean_df$roi == "FusiformGyrus" & Volume_Status_lm_full3_std_mean_df$contrast == "(Control - ADRD)"), "upper.CL"],

Volume_Status_lm_full3_std_mean_df[(Volume_Status_lm_full3_std_mean_df$roi == "LingualGyrus" & Volume_Status_lm_full3_std_mean_df$contrast == "(Control - ADRD)"), "upper.CL"],

Volume_Status_lm_full3_std_mean_df[(Volume_Status_lm_full3_std_mean_df$roi == "EntorhinalCortex_FS" & Volume_Status_lm_full3_std_mean_df$contrast == "(Control - ADRD)"), "upper.CL"],

Volume_Status_lm_full3_std_mean_df[(Volume_Status_lm_full3_std_mean_df$roi == "MiddleInfTempGyrus" & Volume_Status_lm_full3_std_mean_df$contrast == "(Control - ADRD)"), "upper.CL"],

Volume_Status_lm_full3_std_mean_df[(Volume_Status_lm_full3_std_mean_df$roi == "SuperiorTemp" & Volume_Status_lm_full3_std_mean_df$contrast == "(Control - ADRD)"), "upper.CL"],

Volume_Status_lm_full3_std_mean_df[(Volume_Status_lm_full3_std_mean_df$roi == "InferiorParietal" & Volume_Status_lm_full3_std_mean_df$contrast == "(Control - ADRD)"), "upper.CL"],

Volume_Status_lm_full3_std_mean_df[(Volume_Status_lm_full3_std_mean_df$roi == "SuperiorParietal" & Volume_Status_lm_full3_std_mean_df$contrast == "(Control - ADRD)"), "upper.CL"],

Volume_Status_lm_full3_std_mean_df[(Volume_Status_lm_full3_std_mean_df$roi == "Amygdala" & Volume_Status_lm_full3_std_mean_df$contrast == "(Control - ADRD)"), "upper.CL"],

Volume_Status_lm_full3_std_mean_df[(Volume_Status_lm_full3_std_mean_df$roi == "Hippocampus" & Volume_Status_lm_full3_std_mean_df$contrast == "(Control - ADRD)"), "upper.CL"],

Volume_Status_lm_full3_std_mean_df[(Volume_Status_lm_full3_std_mean_df$roi == "Striatum" & Volume_Status_lm_full3_std_mean_df$contrast == "(Control - ADRD)"), "upper.CL"])

p_Volume_MK6240_std_by_Status <-

dat_forest_Volume_MK6240_std_by_Status |>

ggplot(aes(y = fct_rev(model))) +

theme_bw() +

geom_point(aes(x=estimate), shape=12, size=3) +

geom_linerange(aes(xmin=low_CI, xmax=high_CI)) +

geom_vline(xintercept = 0, linetype="dashed") +

labs(x="Control - ADRD", y="") +

coord_cartesian(ylim=c(1,13), xlim=c(-0.75, 3)) +

theme(text = element_text(size = 20), axis.text = element_text(color="black")) +

theme(axis.line = element_line(colour = "black"),

panel.grid.major = element_blank(),

panel.grid.minor = element_blank(),

panel.border = element_blank(),

panel.background = element_blank())

p_Volume_MK6240_std_by_Status

### Hypothesis 2: associations

#https://www.aptech.com/releases/gauss18/graphics-updates/color-brewer-palettes/

all_Spectral_colors <- RColorBrewer::brewer.pal(11,"Spectral")[1:11]

max_RdGy_colors <- RColorBrewer::brewer.pal(11,"RdGy")[1]

max_PuOr_colors <- RColorBrewer::brewer.pal(11,"PuOr")[11]

roi_colors <- c(max_RdGy_colors,all_Spectral_colors,max_PuOr_colors)

analysis.dat.long$roi_order <- factor(analysis.dat.long$roi, levels=

c("Prefrontal","Insula","G_Cing","FusiformGyrus","LingualGyrus",

"EntorhinalCortex_FS","MiddleInfTempGyrus","SuperiorTemp",

"InferiorParietal","SuperiorParietal","Amygdala","Hippocampus","Striatum"))

# complete cases on Status

analysis.dat.long_cc <- analysis.dat.long[complete.cases(analysis.dat.long$Status_baseline),]

analysis.dat.long_cc %>% filter(roi %in% roilist) %>%

ggplot(., aes(y=MK6240, x=ER176)) +

theme_bw() +

geom_point(aes(fill=roi_order, color=roi_order, shape=Status_baseline),alpha=0.75, size=3) +

stat_smooth(aes(fill=roi_order, color=roi_order),method='lm', se=FALSE, alpha=0.5, size=0.5) +

scale_color_manual(values=c(roi_colors)) +

stat_smooth(method='lm', se=FALSE, size=2,color="black", lty=2) +

guides(color = guide_legend(ncol = 3))+

theme(legend.title=element_blank(), legend.position="none", legend.box="vertical") +

ylab("Tau") + xlab("TSPO") +

theme(text = element_text(size = 20), axis.text = element_text(color="black")) +

theme(axis.line = element_line(colour = "black"),

panel.grid.major = element_blank(),

panel.grid.minor = element_blank(),

panel.border = element_blank(),

panel.background = element_blank())

analysis.dat.long_cc %>% filter(roi %in% roilist) %>%

ggplot(., aes(y=FBB, x=ER176)) +

theme_bw() +

geom_point(aes(fill=roi_order, color=roi_order, shape=Status_baseline),alpha=0.75, size=3) +

stat_smooth(aes(fill=roi_order, color=roi_order),method='lm', se=FALSE, alpha=0.5, size=0.5) +

scale_color_manual(values=c(roi_colors)) +

stat_smooth(method='lm', se=FALSE, size=2,color="black", lty=2) +

guides(color = guide_legend(ncol = 3))+

theme(legend.title=element_blank(), legend.position="none", legend.box="vertical") +

ylab("Amyloid") + xlab("TSPO") +

theme(text = element_text(size = 20), axis.text = element_text(color="black")) +

theme(axis.line = element_line(colour = "black"),

panel.grid.major = element_blank(),

panel.grid.minor = element_blank(),

panel.border = element_blank(),

panel.background = element_blank())

analysis.dat.long_cc %>% filter(roi %in% roilist) %>%

ggplot(., aes(y=Volume_MK6240, x=ER176)) +

theme_bw() +

geom_point(aes(fill=roi_order, color=roi_order, shape=Status_baseline),alpha=0.75, size=3) +

stat_smooth(aes(fill=roi_order, color=roi_order),method='lm', se=FALSE, alpha=0.5, size=0.5) +

scale_color_manual(values=c(roi_colors)) +

stat_smooth(method='lm', se=FALSE, size=2,color="black", lty=2) +

guides(color = guide_legend(ncol = 3))+

theme(legend.title=element_blank(), legend.position="none", legend.box="vertical") +

ylab("Neurodegeneration") + xlab("TSPO") +

theme(text = element_text(size = 20), axis.text = element_text(color="black")) +

theme(axis.line = element_line(colour = "black"),

panel.grid.major = element_blank(),

panel.grid.minor = element_blank(),

panel.border = element_blank(),

panel.background = element_blank())

## glm

TSPO_Tau_Amyloid_Volume_lm_full4<-lm(ER176 ~ MK6240*roi + FBB*roi + Volume_MK6240*roi + ICV_baseline + TSPO_Affinity + Age_baseline + Sex + BMI_baseline + APOE4,

analysis.dat.long %>% filter(roi %in% roilist))

TSPO_Tau_Amyloid_Volume_lm_full4_summary<-summary(TSPO_Tau_Amyloid_Volume_lm_full4)

TSPO_Tau_lm_full4_slopes <- emtrends(TSPO_Tau_Amyloid_Volume_lm_full4, ~ roi, var = "MK6240")

TSPO_Tau_lm_full4_contrast <- contrast(TSPO_Tau_lm_full4_slopes,

method = list("Striatum" = c(1, rep(0,12)),

"Amygdala" = c(rep(0,1),1,rep(0,11)),

"EntorhinalCortex_FS" = c(rep(0,2),1,rep(0,10)),

"FusiformGyrus" = c(rep(0,3),1,rep(0,9)),

"G_Cing" = c(rep(0,4),1,rep(0,8)),

"Hippocampus" = c(rep(0,5),1,rep(0,7)),

"InferiorParietal" = c(rep(0,6),1,rep(0,6)),

"Insula" = c(rep(0,7),1,rep(0,5)),

"LingualGyrus" = c(rep(0,8),1,rep(0,4)),

"MiddleInfTempGyrus" = c(rep(0,9),1,rep(0,3)),

"Prefrontal" = c(rep(0,10),1,rep(0,2)),

"SuperiorParietal" = c(rep(0,11),1,rep(0,1)),

"SuperiorTemp" = c(rep(0,12),1)),

weights="proportional",adjust="mvt") %>%

summary(infer=TRUE)

TSPO_Amyloid_lm_full4_slopes <- emtrends(TSPO_Tau_Amyloid_Volume_lm_full4, ~ roi, var = "FBB")

TSPO_Amyloid_lm_full4_contrast <- contrast(TSPO_Amyloid_lm_full4_slopes,

method = list("Striatum" = c(1, rep(0,12)),

"Amygdala" = c(rep(0,1),1,rep(0,11)),

"EntorhinalCortex_FS" = c(rep(0,2),1,rep(0,10)),

"FusiformGyrus" = c(rep(0,3),1,rep(0,9)),

"G_Cing" = c(rep(0,4),1,rep(0,8)),

"Hippocampus" = c(rep(0,5),1,rep(0,7)),

"InferiorParietal" = c(rep(0,6),1,rep(0,6)),

"Insula" = c(rep(0,7),1,rep(0,5)),

"LingualGyrus" = c(rep(0,8),1,rep(0,4)),

"MiddleInfTempGyrus" = c(rep(0,9),1,rep(0,3)),

"Prefrontal" = c(rep(0,10),1,rep(0,2)),

"SuperiorParietal" = c(rep(0,11),1,rep(0,1)),

"SuperiorTemp" = c(rep(0,12),1)),

weights="proportional",adjust="mvt") %>%

summary(infer=TRUE)

TSPO_Volume_lm_full4_slopes <- emtrends(TSPO_Tau_Amyloid_Volume_lm_full4, ~ roi, var = "Volume_MK6240")

TSPO_Volume_lm_full4_contrast <- contrast(TSPO_Volume_lm_full4_slopes,

method = list("Striatum" = c(1, rep(0,12)),

"Amygdala" = c(rep(0,1),1,rep(0,11)),

"EntorhinalCortex_FS" = c(rep(0,2),1,rep(0,10)),

"FusiformGyrus" = c(rep(0,3),1,rep(0,9)),

"G_Cing" = c(rep(0,4),1,rep(0,8)),

"Hippocampus" = c(rep(0,5),1,rep(0,7)),

"InferiorParietal" = c(rep(0,6),1,rep(0,6)),

"Insula" = c(rep(0,7),1,rep(0,5)),

"LingualGyrus" = c(rep(0,8),1,rep(0,4)),

"MiddleInfTempGyrus" = c(rep(0,9),1,rep(0,3)),

"Prefrontal" = c(rep(0,10),1,rep(0,2)),

"SuperiorParietal" = c(rep(0,11),1,rep(0,1)),

"SuperiorTemp" = c(rep(0,12),1)),

weights="proportional",adjust="mvt") %>%

summary(infer=TRUE)

# std

TSPO_Tau_lm_full4_std_contrast <- as.data.frame(eff_size(emtrends(TSPO_Tau_Amyloid_Volume_lm_full4, ~roi, var = "MK6240", weights="proportional",adjust="mvt"),

sigma = sigma(TSPO_Tau_Amyloid_Volume_lm_full4), edf = TSPO_Tau_Amyloid_Volume_lm_full4_summary[[7]][[2]], method=list("Striatum" = c(1, rep(0,12)),

"Amygdala" = c(rep(0,1),1,rep(0,11)),

"EntorhinalCortex_FS" = c(rep(0,2),1,rep(0,10)),

"FusiformGyrus" = c(rep(0,3),1,rep(0,9)),

"G_Cing" = c(rep(0,4),1,rep(0,8)),

"Hippocampus" = c(rep(0,5),1,rep(0,7)),

"InferiorParietal" = c(rep(0,6),1,rep(0,6)),

"Insula" = c(rep(0,7),1,rep(0,5)),

"LingualGyrus" = c(rep(0,8),1,rep(0,4)),

"MiddleInfTempGyrus" = c(rep(0,9),1,rep(0,3)),

"Prefrontal" = c(rep(0,10),1,rep(0,2)),

"SuperiorParietal" = c(rep(0,11),1,rep(0,1)),

"SuperiorTemp" = c(rep(0,12),1))))

TSPO_Amyloid_lm_full4_std_contrast <- as.data.frame(eff_size(emtrends(TSPO_Tau_Amyloid_Volume_lm_full4, ~roi, var = "FBB", weights="proportional",adjust="mvt"),

sigma = sigma(TSPO_Tau_Amyloid_Volume_lm_full4), edf = TSPO_Tau_Amyloid_Volume_lm_full4_summary[[7]][[2]], method=list("Striatum" = c(1, rep(0,12)),

"Amygdala" = c(rep(0,1),1,rep(0,11)),

"EntorhinalCortex_FS" = c(rep(0,2),1,rep(0,10)),

"FusiformGyrus" = c(rep(0,3),1,rep(0,9)),

"G_Cing" = c(rep(0,4),1,rep(0,8)),

"Hippocampus" = c(rep(0,5),1,rep(0,7)),

"InferiorParietal" = c(rep(0,6),1,rep(0,6)),

"Insula" = c(rep(0,7),1,rep(0,5)),

"LingualGyrus" = c(rep(0,8),1,rep(0,4)),

"MiddleInfTempGyrus" = c(rep(0,9),1,rep(0,3)),

"Prefrontal" = c(rep(0,10),1,rep(0,2)),

"SuperiorParietal" = c(rep(0,11),1,rep(0,1)),

"SuperiorTemp" = c(rep(0,12),1))))

TSPO_Volume_lm_full4_std_contrast <- as.data.frame(eff_size(emtrends(TSPO_Tau_Amyloid_Volume_lm_full4, ~roi, var = "Volume_MK6240", weights="proportional",adjust="mvt"),

sigma = sigma(TSPO_Tau_Amyloid_Volume_lm_full4), edf = TSPO_Tau_Amyloid_Volume_lm_full4_summary[[7]][[2]], method=list("Striatum" = c(1, rep(0,12)),

"Amygdala" = c(rep(0,1),1,rep(0,11)),

"EntorhinalCortex_FS" = c(rep(0,2),1,rep(0,10)),

"FusiformGyrus" = c(rep(0,3),1,rep(0,9)),

"G_Cing" = c(rep(0,4),1,rep(0,8)),

"Hippocampus" = c(rep(0,5),1,rep(0,7)),

"InferiorParietal" = c(rep(0,6),1,rep(0,6)),

"Insula" = c(rep(0,7),1,rep(0,5)),

"LingualGyrus" = c(rep(0,8),1,rep(0,4)),

"MiddleInfTempGyrus" = c(rep(0,9),1,rep(0,3)),

"Prefrontal" = c(rep(0,10),1,rep(0,2)),

"SuperiorParietal" = c(rep(0,11),1,rep(0,1)),

"SuperiorTemp" = c(rep(0,12),1))))

### Hypothesis 3: mediations

# set seed

set.seed(1234)

# create individual variables for each level

dat.wide$TSPO_Affinity_Mixed <- as.numeric(dat.wide$TSPO_Affinity == "Mixed")

dat.wide$TSPO_Affinity_High <- as.numeric(dat.wide$TSPO_Affinity == "High Affinity")

dat.wide$APOE4_Carrier <- as.numeric(dat.wide$APOE4 == "Carrier")

# scale continuous variables prior to models for standardization

dat.wide$Age_baseline_scale = scale(dat.wide$Age_baseline)

dat.wide$BMI_baseline_scale = scale(dat.wide$BMI_baseline)

dat.wide$ICV_baseline_scale <- scale(dat.wide$ICV_baseline)

dat.wide$Braak_I_II_MK6240_PVC_baseline_scale = scale(dat.wide$Braak_I_II_MK6240_PVC_baseline)

dat.wide$Braak_III_IV_MK6240_PVC_baseline_scale = scale(dat.wide$Braak_III_IV_MK6240_PVC_baseline)

dat.wide$Braak_V_VI_MK6240_PVC_baseline_scale = scale(dat.wide$Braak_V_VI_MK6240_PVC_baseline)

dat.wide$Composite_FBB_baseline_scale = scale(dat.wide$Composite_FBB_baseline)

dat.wide$Composite_ER176_PVC_baseline_scale = scale(dat.wide$Composite_ER176_PVC_baseline)

dat.wide$Striatum_Volume_MK6240_baseline_scale <- scale(dat.wide$Striatum_Volume_MK6240_baseline)

dat.wide$Amygdala_Volume_MK6240_baseline_scale <- scale(dat.wide$Amygdala_Volume_MK6240_baseline)

dat.wide$EntorhinalCortex_FS_Volume_MK6240_baseline_scale <- scale(dat.wide$EntorhinalCortex_FS_Volume_MK6240_baseline)

dat.wide$FusiformGyrus_Volume_MK6240_baseline_scale <- scale(dat.wide$FusiformGyrus_Volume_MK6240_baseline)

dat.wide$G_Cing_Volume_MK6240_baseline_scale <- scale(dat.wide$G_Cing_Volume_MK6240_baseline)

dat.wide$Hippocampus_Volume_MK6240_baseline_scale <- scale(dat.wide$Hippocampus_Volume_MK6240_baseline)

dat.wide$InferiorParietal_Volume_MK6240_baseline_scale <- scale(dat.wide$InferiorParietal_Volume_MK6240_baseline)

dat.wide$Insula_Volume_MK6240_baseline_scale <- scale(dat.wide$Insula_Volume_MK6240_baseline)

dat.wide$LingualGyrus_Volume_MK6240_baseline_scale <- scale(dat.wide$LingualGyrus_Volume_MK6240_baseline)

dat.wide$MiddleInfTempGyrus_Volume_MK6240_baseline_scale <- scale(dat.wide$MiddleInfTempGyrus_Volume_MK6240_baseline)

dat.wide$Prefrontal_Volume_MK6240_baseline_scale <- scale(dat.wide$Prefrontal_Volume_MK6240_baseline)

dat.wide$SuperiorParietal_Volume_MK6240_baseline_scale <- scale(dat.wide$SuperiorParietal_Volume_MK6240_baseline)

dat.wide$SuperiorTemp_Volume_MK6240_baseline_scale <- scale(dat.wide$SuperiorTemp_Volume_MK6240_baseline)

dat.wide$Striatum_ER176_PVC_baseline_scale <- scale(dat.wide$Striatum_ER176_PVC_baseline)

dat.wide$Amygdala_ER176_PVC_baseline_scale <- scale(dat.wide$Amygdala_ER176_PVC_baseline)

dat.wide$EntorhinalCortex_FS_ER176_PVC_baseline_scale <- scale(dat.wide$EntorhinalCortex_FS_ER176_PVC_baseline)

dat.wide$FusiformGyrus_ER176_PVC_baseline_scale <- scale(dat.wide$FusiformGyrus_ER176_PVC_baseline)

dat.wide$G_Cing_ER176_PVC_baseline_scale <- scale(dat.wide$G_Cing_ER176_PVC_baseline)

dat.wide$Hippocampus_ER176_PVC_baseline_scale <- scale(dat.wide$Hippocampus_ER176_PVC_baseline)

dat.wide$InferiorParietal_ER176_PVC_baseline_scale <- scale(dat.wide$InferiorParietal_ER176_PVC_baseline)

dat.wide$Insula_ER176_PVC_baseline_scale <- scale(dat.wide$Insula_ER176_PVC_baseline)

dat.wide$LingualGyrus_ER176_PVC_baseline_scale <- scale(dat.wide$LingualGyrus_ER176_PVC_baseline)

dat.wide$MiddleInfTempGyrus_ER176_PVC_baseline_scale <- scale(dat.wide$MiddleInfTempGyrus_ER176_PVC_baseline)

dat.wide$Prefrontal_ER176_PVC_baseline_scale <- scale(dat.wide$Prefrontal_ER176_PVC_baseline)

dat.wide$SuperiorParietal_ER176_PVC_baseline_scale <- scale(dat.wide$SuperiorParietal_ER176_PVC_baseline)

dat.wide$SuperiorTemp_ER176_PVC_baseline_scale <- scale(dat.wide$SuperiorTemp_ER176_PVC_baseline)

dat.wide$Striatum_MK6240_PVC_baseline_scale <- scale(dat.wide$Striatum_MK6240_PVC_baseline)

dat.wide$Amygdala_MK6240_PVC_baseline_scale <- scale(dat.wide$Amygdala_MK6240_PVC_baseline)

dat.wide$EntorhinalCortex_FS_MK6240_PVC_baseline_scale <- scale(dat.wide$EntorhinalCortex_FS_MK6240_PVC_baseline)

dat.wide$FusiformGyrus_MK6240_PVC_baseline_scale <- scale(dat.wide$FusiformGyrus_MK6240_PVC_baseline)

dat.wide$G_Cing_MK6240_PVC_baseline_scale <- scale(dat.wide$G_Cing_MK6240_PVC_baseline)

dat.wide$Hippocampus_MK6240_PVC_baseline_scale <- scale(dat.wide$Hippocampus_MK6240_PVC_baseline)

dat.wide$InferiorParietal_MK6240_PVC_baseline_scale <- scale(dat.wide$InferiorParietal_MK6240_PVC_baseline)

dat.wide$Insula_MK6240_PVC_baseline_scale <- scale(dat.wide$Insula_MK6240_PVC_baseline)

dat.wide$LingualGyrus_MK6240_PVC_baseline_scale <- scale(dat.wide$LingualGyrus_MK6240_PVC_baseline)

dat.wide$MiddleInfTempGyrus_MK6240_PVC_baseline_scale <- scale(dat.wide$MiddleInfTempGyrus_MK6240_PVC_baseline)

dat.wide$Prefrontal_MK6240_PVC_baseline_scale <- scale(dat.wide$Prefrontal_MK6240_PVC_baseline)

dat.wide$SuperiorParietal_MK6240_PVC_baseline_scale <- scale(dat.wide$SuperiorParietal_MK6240_PVC_baseline)

dat.wide$SuperiorTemp_MK6240_PVC_baseline_scale <- scale(dat.wide$SuperiorTemp_MK6240_PVC_baseline)

dat.wide$Striatum_FBB_baseline_scale <- scale(dat.wide$Striatum_FBB_baseline)

dat.wide$Amygdala_FBB_baseline_scale <- scale(dat.wide$Amygdala_FBB_baseline)

dat.wide$EntorhinalCortex_FS_FBB_baseline_scale <- scale(dat.wide$EntorhinalCortex_FS_FBB_baseline)

dat.wide$FusiformGyrus_FBB_baseline_scale <- scale(dat.wide$FusiformGyrus_FBB_baseline)

dat.wide$G_Cing_FBB_baseline_scale <- scale(dat.wide$G_Cing_FBB_baseline)

dat.wide$Hippocampus_FBB_baseline_scale <- scale(dat.wide$Hippocampus_FBB_baseline)

dat.wide$InferiorParietal_FBB_baseline_scale <- scale(dat.wide$InferiorParietal_FBB_baseline)

dat.wide$Insula_FBB_baseline_scale <- scale(dat.wide$Insula_FBB_baseline)

dat.wide$LingualGyrus_FBB_baseline_scale <- scale(dat.wide$LingualGyrus_FBB_baseline)

dat.wide$MiddleInfTempGyrus_FBB_baseline_scale <- scale(dat.wide$MiddleInfTempGyrus_FBB_baseline)

dat.wide$Prefrontal_FBB_baseline_scale <- scale(dat.wide$Prefrontal_FBB_baseline)

dat.wide$SuperiorParietal_FBB_baseline_scale <- scale(dat.wide$SuperiorParietal_FBB_baseline)

dat.wide$SuperiorTemp_FBB_baseline_scale <- scale(dat.wide$SuperiorTemp_FBB_baseline)

## sample size per mediation

# A -> TSPO -> T

dat.wide_ATSPOT <- dat.wide[complete.cases(dat.wide[c("Striatum_ER176_baseline", "Striatum_MK6240_baseline", "Striatum_FBB_baseline",

"TSPO_Affinity","Age_baseline","Sex","BMI_baseline","APOE4")]),]

unique(dat.wide_ATSPOT$ID)

# T -> TSPO -> T

dat.wide_TTSPOT <- dat.wide[complete.cases(dat.wide[c("Striatum_ER176_baseline", "Striatum_MK6240_baseline",

"TSPO_Affinity","Age_baseline","Sex","BMI_baseline","APOE4")]),]

unique(dat.wide_TTSPOT$ID)

# T -> TSPO -> N

dat.wide_TTSPON <- dat.wide[complete.cases(dat.wide[c("Striatum_ER176_baseline", "Striatum_MK6240_baseline", "Striatum_Volume_MK6240_baseline",

"ICV_baseline","TSPO_Affinity","Age_baseline","Sex","BMI_baseline","APOE4")]),]

unique(dat.wide_TTSPON$ID)

## mediations

# A -> TSPO -> T std

Striatum_A_T_full3_TSPO_mediation_std =

"

#Regressions

# specify direct effect, path c' (Y~X)

Striatum_MK6240_PVC_baseline_scale ~ c*Striatum_FBB_baseline_scale + Age_baseline_scale + Sex + BMI_baseline_scale + APOE4_Carrier

# path a (M~X)

Striatum_ER176_PVC_baseline_scale ~ a*Striatum_FBB_baseline_scale + TSPO_Affinity_Mixed + TSPO_Affinity_High + Age_baseline_scale + Sex + BMI_baseline_scale + APOE4_Carrier

# path b (Y~M)

Striatum_MK6240_PVC_baseline_scale ~ b*Striatum_ER176_PVC_baseline_scale + APOE4_Carrier

#Defined Parameters:

mediation := a*b

total := c+(a*b)

"

fit_Striatum_A_T_full3_TSPO_mediation_std<-sem(Striatum_A_T_full3_TSPO_mediation_std,

dat.wide)

fit_Striatum_A_T_full3_TSPO_mediation_std_summary <- summary(fit_Striatum_A_T_full3_TSPO_mediation_std,

fit.measures=TRUE,standardized=TRUE,rsquare=TRUE)

fit_Striatum_A_T_full3_TSPO_mediation_std_parameters <- parameterEstimates(fit_Striatum_A_T_full3_TSPO_mediation_std,

ci=TRUE,level=0.95,boot.ci.type="perc",standardized=TRUE)

Amygdala_A_T_full3_TSPO_mediation_std =

"

#Regressions

# specify direct effect, path c' (Y~X)

Amygdala_MK6240_PVC_baseline_scale ~ c*Amygdala_FBB_baseline_scale + Age_baseline_scale + Sex + BMI_baseline_scale + APOE4_Carrier

# path a (M~X)

Amygdala_ER176_PVC_baseline_scale ~ a*Amygdala_FBB_baseline_scale + TSPO_Affinity_Mixed + TSPO_Affinity_High + Age_baseline_scale + Sex + BMI_baseline_scale + APOE4_Carrier

# path b (Y~M)

Amygdala_MK6240_PVC_baseline_scale ~ b*Amygdala_ER176_PVC_baseline_scale + APOE4_Carrier

#Defined Parameters:

mediation := a*b

total := c+(a*b)

"

fit_Amygdala_A_T_full3_TSPO_mediation_std<-sem(Amygdala_A_T_full3_TSPO_mediation_std,

dat.wide)

fit_Amygdala_A_T_full3_TSPO_mediation_std_summary <- summary(fit_Amygdala_A_T_full3_TSPO_mediation_std,

fit.measures=TRUE,standardized=TRUE,rsquare=TRUE)

fit_Amygdala_A_T_full3_TSPO_mediation_std_parameters <- parameterEstimates(fit_Amygdala_A_T_full3_TSPO_mediation_std,

ci=TRUE,level=0.95,boot.ci.type="perc",standardized=TRUE)

EntorhinalCortex_FS_A_T_full3_TSPO_mediation_std =

"

#Regressions

# specify direct effect, path c' (Y~X)

EntorhinalCortex_FS_MK6240_PVC_baseline_scale ~ c*EntorhinalCortex_FS_FBB_baseline_scale + Age_baseline_scale + Sex + BMI_baseline_scale + APOE4_Carrier

# path a (M~X)

EntorhinalCortex_FS_ER176_PVC_baseline_scale ~ a*EntorhinalCortex_FS_FBB_baseline_scale + TSPO_Affinity_Mixed + TSPO_Affinity_High + Age_baseline_scale + Sex + BMI_baseline_scale + APOE4_Carrier

# path b (Y~M)

EntorhinalCortex_FS_MK6240_PVC_baseline_scale ~ b*EntorhinalCortex_FS_ER176_PVC_baseline_scale + APOE4_Carrier

#Defined Parameters:

mediation := a*b

total := c+(a*b)

"

fit_EntorhinalCortex_FS_A_T_full3_TSPO_mediation_std<-sem(EntorhinalCortex_FS_A_T_full3_TSPO_mediation_std,

dat.wide)

fit_EntorhinalCortex_FS_A_T_full3_TSPO_mediation_std_summary <- summary(fit_EntorhinalCortex_FS_A_T_full3_TSPO_mediation_std,

fit.measures=TRUE,standardized=TRUE,rsquare=TRUE)

fit_EntorhinalCortex_FS_A_T_full3_TSPO_mediation_std_parameters <- parameterEstimates(fit_EntorhinalCortex_FS_A_T_full3_TSPO_mediation_std,

ci=TRUE,level=0.95,boot.ci.type="perc",standardized=TRUE)

FusiformGyrus_A_T_full3_TSPO_mediation_std =

"

#Regressions

# specify direct effect, path c' (Y~X)

FusiformGyrus_MK6240_PVC_baseline_scale ~ c*FusiformGyrus_FBB_baseline_scale + Age_baseline_scale + Sex + BMI_baseline_scale + APOE4_Carrier

# path a (M~X)

FusiformGyrus_ER176_PVC_baseline_scale ~ a*FusiformGyrus_FBB_baseline_scale + TSPO_Affinity_Mixed + TSPO_Affinity_High + Age_baseline_scale + Sex + BMI_baseline_scale + APOE4_Carrier

# path b (Y~M)

FusiformGyrus_MK6240_PVC_baseline_scale ~ b*FusiformGyrus_ER176_PVC_baseline_scale + APOE4_Carrier

#Defined Parameters:

mediation := a*b

total := c+(a*b)

"

fit_FusiformGyrus_A_T_full3_TSPO_mediation_std<-sem(FusiformGyrus_A_T_full3_TSPO_mediation_std,

dat.wide)

fit_FusiformGyrus_A_T_full3_TSPO_mediation_std_summary <- summary(fit_FusiformGyrus_A_T_full3_TSPO_mediation_std,

fit.measures=TRUE,standardized=TRUE,rsquare=TRUE)

fit_FusiformGyrus_A_T_full3_TSPO_mediation_std_parameters <- parameterEstimates(fit_FusiformGyrus_A_T_full3_TSPO_mediation_std,

ci=TRUE,level=0.95,boot.ci.type="perc",standardized=TRUE)

G_Cing_A_T_full3_TSPO_mediation_std =

"

#Regressions

# specify direct effect, path c' (Y~X)

G_Cing_MK6240_PVC_baseline_scale ~ c*G_Cing_FBB_baseline_scale + Age_baseline_scale + Sex + BMI_baseline_scale + APOE4_Carrier

# path a (M~X)

G_Cing_ER176_PVC_baseline_scale ~ a*G_Cing_FBB_baseline_scale + TSPO_Affinity_Mixed + TSPO_Affinity_High + Age_baseline_scale + Sex + BMI_baseline_scale + APOE4_Carrier

# path b (Y~M)

G_Cing_MK6240_PVC_baseline_scale ~ b*G_Cing_ER176_PVC_baseline_scale + APOE4_Carrier

#Defined Parameters:

mediation := a*b

total := c+(a*b)

"

fit_G_Cing_A_T_full3_TSPO_mediation_std<-sem(G_Cing_A_T_full3_TSPO_mediation_std,

dat.wide)

fit_G_Cing_A_T_full3_TSPO_mediation_std_summary <- summary(fit_G_Cing_A_T_full3_TSPO_mediation_std,

fit.measures=TRUE,standardized=TRUE,rsquare=TRUE)

fit_G_Cing_A_T_full3_TSPO_mediation_std_parameters <- parameterEstimates(fit_G_Cing_A_T_full3_TSPO_mediation_std,

ci=TRUE,level=0.95,boot.ci.type="perc",standardized=TRUE)

Hippocampus_A_T_full3_TSPO_mediation_std =

"

#Regressions

# specify direct effect, path c' (Y~X)

Hippocampus_MK6240_PVC_baseline_scale ~ c*Hippocampus_FBB_baseline_scale + Age_baseline_scale + Sex + BMI_baseline_scale + APOE4_Carrier

# path a (M~X)

Hippocampus_ER176_PVC_baseline_scale ~ a*Hippocampus_FBB_baseline_scale + TSPO_Affinity_Mixed + TSPO_Affinity_High + Age_baseline_scale + Sex + BMI_baseline_scale + APOE4_Carrier

# path b (Y~M)

Hippocampus_MK6240_PVC_baseline_scale ~ b*Hippocampus_ER176_PVC_baseline_scale + APOE4_Carrier

#Defined Parameters:

mediation := a*b

total := c+(a*b)

"

fit_Hippocampus_A_T_full3_TSPO_mediation_std<-sem(Hippocampus_A_T_full3_TSPO_mediation_std,

dat.wide)

fit_Hippocampus_A_T_full3_TSPO_mediation_std_summary <- summary(fit_Hippocampus_A_T_full3_TSPO_mediation_std,

fit.measures=TRUE,standardized=TRUE,rsquare=TRUE)

fit_Hippocampus_A_T_full3_TSPO_mediation_std_parameters <- parameterEstimates(fit_Hippocampus_A_T_full3_TSPO_mediation_std,

ci=TRUE,level=0.95,boot.ci.type="perc",standardized=TRUE)

InferiorParietal_A_T_full3_TSPO_mediation_std =

"

#Regressions

# specify direct effect, path c' (Y~X)

InferiorParietal_MK6240_PVC_baseline_scale ~ c*InferiorParietal_FBB_baseline_scale + Age_baseline_scale + Sex + BMI_baseline_scale + APOE4_Carrier

# path a (M~X)

InferiorParietal_ER176_PVC_baseline_scale ~ a*InferiorParietal_FBB_baseline_scale + TSPO_Affinity_Mixed + TSPO_Affinity_High + Age_baseline_scale + Sex + BMI_baseline_scale + APOE4_Carrier

# path b (Y~M)

InferiorParietal_MK6240_PVC_baseline_scale ~ b*InferiorParietal_ER176_PVC_baseline_scale + APOE4_Carrier

#Defined Parameters:

mediation := a*b

total := c+(a*b)

"

fit_InferiorParietal_A_T_full3_TSPO_mediation_std<-sem(InferiorParietal_A_T_full3_TSPO_mediation_std,

dat.wide)

fit_InferiorParietal_A_T_full3_TSPO_mediation_std_summary <- summary(fit_InferiorParietal_A_T_full3_TSPO_mediation_std,

fit.measures=TRUE,standardized=TRUE,rsquare=TRUE)

fit_InferiorParietal_A_T_full3_TSPO_mediation_std_parameters <- parameterEstimates(fit_InferiorParietal_A_T_full3_TSPO_mediation_std,

ci=TRUE,level=0.95,boot.ci.type="perc",standardized=TRUE)

Insula_A_T_full3_TSPO_mediation_std =

"

#Regressions

# specify direct effect, path c' (Y~X)

Insula_MK6240_PVC_baseline_scale ~ c*Insula_FBB_baseline_scale + Age_baseline_scale + Sex + BMI_baseline_scale + APOE4_Carrier

# path a (M~X)

Insula_ER176_PVC_baseline_scale ~ a*Insula_FBB_baseline_scale + TSPO_Affinity_Mixed + TSPO_Affinity_High + Age_baseline_scale + Sex + BMI_baseline_scale + APOE4_Carrier

# path b (Y~M)

Insula_MK6240_PVC_baseline_scale ~ b*Insula_ER176_PVC_baseline_scale + APOE4_Carrier

#Defined Parameters:

mediation := a*b

total := c+(a*b)

"

fit_Insula_A_T_full3_TSPO_mediation_std<-sem(Insula_A_T_full3_TSPO_mediation_std,

dat.wide)

fit_Insula_A_T_full3_TSPO_mediation_std_summary <- summary(fit_Insula_A_T_full3_TSPO_mediation_std,

fit.measures=TRUE,standardized=TRUE,rsquare=TRUE)

fit_Insula_A_T_full3_TSPO_mediation_std_parameters <- parameterEstimates(fit_Insula_A_T_full3_TSPO_mediation_std,

ci=TRUE,level=0.95,boot.ci.type="perc",standardized=TRUE)

LingualGyrus_A_T_full3_TSPO_mediation_std =

"

#Regressions

# specify direct effect, path c' (Y~X)

LingualGyrus_MK6240_PVC_baseline_scale ~ c*LingualGyrus_FBB_baseline_scale + Age_baseline_scale + Sex + BMI_baseline_scale + APOE4_Carrier

# path a (M~X)

LingualGyrus_ER176_PVC_baseline_scale ~ a*LingualGyrus_FBB_baseline_scale + TSPO_Affinity_Mixed + TSPO_Affinity_High + Age_baseline_scale + Sex + BMI_baseline_scale + APOE4_Carrier

# path b (Y~M)

LingualGyrus_MK6240_PVC_baseline_scale ~ b*LingualGyrus_ER176_PVC_baseline_scale + APOE4_Carrier

#Defined Parameters:

mediation := a*b

total := c+(a*b)

"

fit_LingualGyrus_A_T_full3_TSPO_mediation_std<-sem(LingualGyrus_A_T_full3_TSPO_mediation_std,

dat.wide)

fit_LingualGyrus_A_T_full3_TSPO_mediation_std_summary <- summary(fit_LingualGyrus_A_T_full3_TSPO_mediation_std,

fit.measures=TRUE,standardized=TRUE,rsquare=TRUE)

fit_LingualGyrus_A_T_full3_TSPO_mediation_std_parameters <- parameterEstimates(fit_LingualGyrus_A_T_full3_TSPO_mediation_std,

ci=TRUE,level=0.95,boot.ci.type="perc",standardized=TRUE)

MiddleInfTempGyrus_A_T_full3_TSPO_mediation_std =

"

#Regressions

# specify direct effect, path c' (Y~X)

MiddleInfTempGyrus_MK6240_PVC_baseline_scale ~ c*MiddleInfTempGyrus_FBB_baseline_scale + Age_baseline_scale + Sex + BMI_baseline_scale + APOE4_Carrier

# path a (M~X)

MiddleInfTempGyrus_ER176_PVC_baseline_scale ~ a*MiddleInfTempGyrus_FBB_baseline_scale + TSPO_Affinity_Mixed + TSPO_Affinity_High + Age_baseline_scale + Sex + BMI_baseline_scale + APOE4_Carrier

# path b (Y~M)

MiddleInfTempGyrus_MK6240_PVC_baseline_scale ~ b*MiddleInfTempGyrus_ER176_PVC_baseline_scale + APOE4_Carrier

#Defined Parameters:

mediation := a*b

total := c+(a*b)

"

fit_MiddleInfTempGyrus_A_T_full3_TSPO_mediation_std<-sem(MiddleInfTempGyrus_A_T_full3_TSPO_mediation_std,

dat.wide)

fit_MiddleInfTempGyrus_A_T_full3_TSPO_mediation_std_summary <- summary(fit_MiddleInfTempGyrus_A_T_full3_TSPO_mediation_std,

fit.measures=TRUE,standardized=TRUE,rsquare=TRUE)

fit_MiddleInfTempGyrus_A_T_full3_TSPO_mediation_std_parameters <- parameterEstimates(fit_MiddleInfTempGyrus_A_T_full3_TSPO_mediation_std,

ci=TRUE,level=0.95,boot.ci.type="perc",standardized=TRUE)

Prefrontal_A_T_full3_TSPO_mediation_std =

"

#Regressions

# specify direct effect, path c' (Y~X)

Prefrontal_MK6240_PVC_baseline_scale ~ c*Prefrontal_FBB_baseline_scale + Age_baseline_scale + Sex + BMI_baseline_scale + APOE4_Carrier

# path a (M~X)

Prefrontal_ER176_PVC_baseline_scale ~ a*Prefrontal_FBB_baseline_scale + TSPO_Affinity_Mixed + TSPO_Affinity_High + Age_baseline_scale + Sex + BMI_baseline_scale + APOE4_Carrier

# path b (Y~M)

Prefrontal_MK6240_PVC_baseline_scale ~ b*Prefrontal_ER176_PVC_baseline_scale + APOE4_Carrier

#Defined Parameters:

mediation := a*b

total := c+(a*b)

"

fit_Prefrontal_A_T_full3_TSPO_mediation_std<-sem(Prefrontal_A_T_full3_TSPO_mediation_std,

dat.wide)

fit_Prefrontal_A_T_full3_TSPO_mediation_std_summary <- summary(fit_Prefrontal_A_T_full3_TSPO_mediation_std,

fit.measures=TRUE,standardized=TRUE,rsquare=TRUE)

fit_Prefrontal_A_T_full3_TSPO_mediation_std_parameters <- parameterEstimates(fit_Prefrontal_A_T_full3_TSPO_mediation_std,

ci=TRUE,level=0.95,boot.ci.type="perc",standardized=TRUE)

SuperiorParietal_A_T_full3_TSPO_mediation_std =

"

#Regressions

# specify direct effect, path c' (Y~X)

SuperiorParietal_MK6240_PVC_baseline_scale ~ c*SuperiorParietal_FBB_baseline_scale + Age_baseline_scale + Sex + BMI_baseline_scale + APOE4_Carrier

# path a (M~X)

SuperiorParietal_ER176_PVC_baseline_scale ~ a*SuperiorParietal_FBB_baseline_scale + TSPO_Affinity_Mixed + TSPO_Affinity_High + Age_baseline_scale + Sex + BMI_baseline_scale + APOE4_Carrier

# path b (Y~M)

SuperiorParietal_MK6240_PVC_baseline_scale ~ b*SuperiorParietal_ER176_PVC_baseline_scale + APOE4_Carrier

#Defined Parameters:

mediation := a*b

total := c+(a*b)

"

fit_SuperiorParietal_A_T_full3_TSPO_mediation_std<-sem(SuperiorParietal_A_T_full3_TSPO_mediation_std,

dat.wide)

fit_SuperiorParietal_A_T_full3_TSPO_mediation_std_summary <- summary(fit_SuperiorParietal_A_T_full3_TSPO_mediation_std,

fit.measures=TRUE,standardized=TRUE,rsquare=TRUE)

fit_SuperiorParietal_A_T_full3_TSPO_mediation_std_parameters <- parameterEstimates(fit_SuperiorParietal_A_T_full3_TSPO_mediation_std,

ci=TRUE,level=0.95,boot.ci.type="perc",standardized=TRUE)

SuperiorTemp_A_T_full3_TSPO_mediation_std =

"

#Regressions

# specify direct effect, path c' (Y~X)

SuperiorTemp_MK6240_PVC_baseline_scale ~ c*SuperiorTemp_FBB_baseline_scale + Age_baseline_scale + Sex + BMI_baseline_scale + APOE4_Carrier

# path a (M~X)

SuperiorTemp_ER176_PVC_baseline_scale ~ a*SuperiorTemp_FBB_baseline_scale + TSPO_Affinity_Mixed + TSPO_Affinity_High + Age_baseline_scale + Sex + BMI_baseline_scale + APOE4_Carrier

# path b (Y~M)

SuperiorTemp_MK6240_PVC_baseline_scale ~ b*SuperiorTemp_ER176_PVC_baseline_scale + APOE4_Carrier

#Defined Parameters:

mediation := a*b

total := c+(a*b)

"

fit_SuperiorTemp_A_T_full3_TSPO_mediation_std<-sem(SuperiorTemp_A_T_full3_TSPO_mediation_std,

dat.wide)

fit_SuperiorTemp_A_T_full3_TSPO_mediation_std_summary <- summary(fit_SuperiorTemp_A_T_full3_TSPO_mediation_std,

fit.measures=TRUE,standardized=TRUE,rsquare=TRUE)

fit_SuperiorTemp_A_T_full3_TSPO_mediation_std_parameters <- parameterEstimates(fit_SuperiorTemp_A_T_full3_TSPO_mediation_std,

ci=TRUE,level=0.95,boot.ci.type="perc",standardized=TRUE)

## adjust pvalues

AT_rois <- c("Prefrontal","Insula","G_Cing","FusiformGyrus","LingualGyrus",

"EntorhinalCortex_FS","MiddleInfTempGyrus","SuperiorTemp",

"InferiorParietal","SuperiorParietal",

"Amygdala","Hippocampus","Striatum")

# Amyloid to TPSO

AT_TSPO_mediation_A_p <- c(fit_Prefrontal_A_T_full3_TSPO_mediation_parameters[fit_Prefrontal_A_T_full3_TSPO_mediation_parameters$label=="a","pvalue"],

fit_Insula_A_T_full3_TSPO_mediation_parameters[fit_Insula_A_T_full3_TSPO_mediation_parameters$label=="a","pvalue"],

fit_G_Cing_A_T_full3_TSPO_mediation_parameters[fit_G_Cing_A_T_full3_TSPO_mediation_parameters$label=="a","pvalue"],

fit_FusiformGyrus_A_T_full3_TSPO_mediation_parameters[fit_FusiformGyrus_A_T_full3_TSPO_mediation_parameters$label=="a","pvalue"],

fit_LingualGyrus_A_T_full3_TSPO_mediation_parameters[fit_LingualGyrus_A_T_full3_TSPO_mediation_parameters$label=="a","pvalue"],

fit_EntorhinalCortex_FS_A_T_full3_TSPO_mediation_parameters[fit_EntorhinalCortex_FS_A_T_full3_TSPO_mediation_parameters$label=="a","pvalue"],

fit_MiddleInfTempGyrus_A_T_full3_TSPO_mediation_parameters[fit_MiddleInfTempGyrus_A_T_full3_TSPO_mediation_parameters$label=="a","pvalue"],

fit_SuperiorTemp_A_T_full3_TSPO_mediation_parameters[fit_SuperiorTemp_A_T_full3_TSPO_mediation_parameters$label=="a","pvalue"],

fit_InferiorParietal_A_T_full3_TSPO_mediation_parameters[fit_InferiorParietal_A_T_full3_TSPO_mediation_parameters$label=="a","pvalue"],

fit_SuperiorParietal_A_T_full3_TSPO_mediation_parameters[fit_SuperiorParietal_A_T_full3_TSPO_mediation_parameters$label=="a","pvalue"],

fit_Amygdala_A_T_full3_TSPO_mediation_parameters[fit_Amygdala_A_T_full3_TSPO_mediation_parameters$label=="a","pvalue"],

fit_Hippocampus_A_T_full3_TSPO_mediation_parameters[fit_Hippocampus_A_T_full3_TSPO_mediation_parameters$label=="a","pvalue"],

fit_Striatum_A_T_full3_TSPO_mediation_parameters[fit_Striatum_A_T_full3_TSPO_mediation_parameters$label=="a","pvalue"])

AT_TSPO_mediation_A_p_adjusted <- p.adjust(AT_TSPO_mediation_A_p, method = "BH", n = length(AT_TSPO_mediation_A_p))

AT_TSPO_mediation_A_p_adjusted_roi <- cbind(AT_rois,AT_TSPO_mediation_A_p_adjusted)

AT_TSPO_mediation_A_p_adjusted_df <- as.data.frame(AT_TSPO_mediation_A_p_adjusted_roi)

# TSPO to Tau

AT_TSPO_mediation_B_p <- c(fit_Prefrontal_A_T_full3_TSPO_mediation_parameters[fit_Prefrontal_A_T_full3_TSPO_mediation_parameters$label=="b","pvalue"],

fit_Insula_A_T_full3_TSPO_mediation_parameters[fit_Insula_A_T_full3_TSPO_mediation_parameters$label=="b","pvalue"],

fit_G_Cing_A_T_full3_TSPO_mediation_parameters[fit_G_Cing_A_T_full3_TSPO_mediation_parameters$label=="b","pvalue"],

fit_FusiformGyrus_A_T_full3_TSPO_mediation_parameters[fit_FusiformGyrus_A_T_full3_TSPO_mediation_parameters$label=="b","pvalue"],

fit_LingualGyrus_A_T_full3_TSPO_mediation_parameters[fit_LingualGyrus_A_T_full3_TSPO_mediation_parameters$label=="b","pvalue"],

fit_EntorhinalCortex_FS_A_T_full3_TSPO_mediation_parameters[fit_EntorhinalCortex_FS_A_T_full3_TSPO_mediation_parameters$label=="b","pvalue"],

fit_MiddleInfTempGyrus_A_T_full3_TSPO_mediation_parameters[fit_MiddleInfTempGyrus_A_T_full3_TSPO_mediation_parameters$label=="b","pvalue"],

fit_SuperiorTemp_A_T_full3_TSPO_mediation_parameters[fit_SuperiorTemp_A_T_full3_TSPO_mediation_parameters$label=="b","pvalue"],

fit_InferiorParietal_A_T_full3_TSPO_mediation_parameters[fit_InferiorParietal_A_T_full3_TSPO_mediation_parameters$label=="b","pvalue"],

fit_SuperiorParietal_A_T_full3_TSPO_mediation_parameters[fit_SuperiorParietal_A_T_full3_TSPO_mediation_parameters$label=="b","pvalue"],

fit_Amygdala_A_T_full3_TSPO_mediation_parameters[fit_Amygdala_A_T_full3_TSPO_mediation_parameters$label=="b","pvalue"],

fit_Hippocampus_A_T_full3_TSPO_mediation_parameters[fit_Hippocampus_A_T_full3_TSPO_mediation_parameters$label=="b","pvalue"],

fit_Striatum_A_T_full3_TSPO_mediation_parameters[fit_Striatum_A_T_full3_TSPO_mediation_parameters$label=="b","pvalue"])

AT_TSPO_mediation_B_p_adjusted <- p.adjust(AT_TSPO_mediation_B_p, method = "BH", n = length(AT_TSPO_mediation_B_p))

AT_TSPO_mediation_B_p_adjusted_roi <- cbind(AT_rois,AT_TSPO_mediation_B_p_adjusted)

AT_TSPO_mediation_B_p_adjusted_df <- as.data.frame(AT_TSPO_mediation_B_p_adjusted_roi)

# Amyloid to Tau

AT_TSPO_mediation_C_p <- c(fit_Prefrontal_A_T_full3_TSPO_mediation_parameters[fit_Prefrontal_A_T_full3_TSPO_mediation_parameters$label=="c","pvalue"],

fit_Insula_A_T_full3_TSPO_mediation_parameters[fit_Insula_A_T_full3_TSPO_mediation_parameters$label=="c","pvalue"],

fit_G_Cing_A_T_full3_TSPO_mediation_parameters[fit_G_Cing_A_T_full3_TSPO_mediation_parameters$label=="c","pvalue"],

fit_FusiformGyrus_A_T_full3_TSPO_mediation_parameters[fit_FusiformGyrus_A_T_full3_TSPO_mediation_parameters$label=="c","pvalue"],

fit_LingualGyrus_A_T_full3_TSPO_mediation_parameters[fit_LingualGyrus_A_T_full3_TSPO_mediation_parameters$label=="c","pvalue"],

fit_EntorhinalCortex_FS_A_T_full3_TSPO_mediation_parameters[fit_EntorhinalCortex_FS_A_T_full3_TSPO_mediation_parameters$label=="c","pvalue"],

fit_MiddleInfTempGyrus_A_T_full3_TSPO_mediation_parameters[fit_MiddleInfTempGyrus_A_T_full3_TSPO_mediation_parameters$label=="c","pvalue"],

fit_SuperiorTemp_A_T_full3_TSPO_mediation_parameters[fit_SuperiorTemp_A_T_full3_TSPO_mediation_parameters$label=="c","pvalue"],

fit_InferiorParietal_A_T_full3_TSPO_mediation_parameters[fit_InferiorParietal_A_T_full3_TSPO_mediation_parameters$label=="c","pvalue"],

fit_SuperiorParietal_A_T_full3_TSPO_mediation_parameters[fit_SuperiorParietal_A_T_full3_TSPO_mediation_parameters$label=="c","pvalue"],

fit_Amygdala_A_T_full3_TSPO_mediation_parameters[fit_Amygdala_A_T_full3_TSPO_mediation_parameters$label=="c","pvalue"],

fit_Hippocampus_A_T_full3_TSPO_mediation_parameters[fit_Hippocampus_A_T_full3_TSPO_mediation_parameters$label=="c","pvalue"],

fit_Striatum_A_T_full3_TSPO_mediation_parameters[fit_Striatum_A_T_full3_TSPO_mediation_parameters$label=="c","pvalue"])

AT_TSPO_mediation_C_p_adjusted <- p.adjust(AT_TSPO_mediation_C_p, method = "BH", n = length(AT_TSPO_mediation_C_p))

AT_TSPO_mediation_C_p_adjusted_roi <- cbind(AT_rois,AT_TSPO_mediation_C_p_adjusted)

AT_TSPO_mediation_C_p_adjusted_df <- as.data.frame(AT_TSPO_mediation_C_p_adjusted_roi)

# Amyloid to TSPO to Tau

AT_TSPO_mediation_Cprime_p <- c(fit_Prefrontal_A_T_full3_TSPO_mediation_parameters[fit_Prefrontal_A_T_full3_TSPO_mediation_parameters$label=="mediation","pvalue"],

fit_Insula_A_T_full3_TSPO_mediation_parameters[fit_Insula_A_T_full3_TSPO_mediation_parameters$label=="mediation","pvalue"],

fit_G_Cing_A_T_full3_TSPO_mediation_parameters[fit_G_Cing_A_T_full3_TSPO_mediation_parameters$label=="mediation","pvalue"],

fit_FusiformGyrus_A_T_full3_TSPO_mediation_parameters[fit_FusiformGyrus_A_T_full3_TSPO_mediation_parameters$label=="mediation","pvalue"],

fit_LingualGyrus_A_T_full3_TSPO_mediation_parameters[fit_LingualGyrus_A_T_full3_TSPO_mediation_parameters$label=="mediation","pvalue"],

fit_EntorhinalCortex_FS_A_T_full3_TSPO_mediation_parameters[fit_EntorhinalCortex_FS_A_T_full3_TSPO_mediation_parameters$label=="mediation","pvalue"],

fit_MiddleInfTempGyrus_A_T_full3_TSPO_mediation_parameters[fit_MiddleInfTempGyrus_A_T_full3_TSPO_mediation_parameters$label=="mediation","pvalue"],

fit_SuperiorTemp_A_T_full3_TSPO_mediation_parameters[fit_SuperiorTemp_A_T_full3_TSPO_mediation_parameters$label=="mediation","pvalue"],

fit_InferiorParietal_A_T_full3_TSPO_mediation_parameters[fit_InferiorParietal_A_T_full3_TSPO_mediation_parameters$label=="mediation","pvalue"],

fit_SuperiorParietal_A_T_full3_TSPO_mediation_parameters[fit_SuperiorParietal_A_T_full3_TSPO_mediation_parameters$label=="mediation","pvalue"],

fit_Amygdala_A_T_full3_TSPO_mediation_parameters[fit_Amygdala_A_T_full3_TSPO_mediation_parameters$label=="mediation","pvalue"],

fit_Hippocampus_A_T_full3_TSPO_mediation_parameters[fit_Hippocampus_A_T_full3_TSPO_mediation_parameters$label=="mediation","pvalue"],

fit_Striatum_A_T_full3_TSPO_mediation_parameters[fit_Striatum_A_T_full3_TSPO_mediation_parameters$label=="mediation","pvalue"])

AT_TSPO_mediation_Cprime_p_adjusted <- p.adjust(AT_TSPO_mediation_Cprime_p, method = "BH", n = length(AT_TSPO_mediation_Cprime_p))

AT_TSPO_mediation_Cprime_p_adjusted_roi <- cbind(AT_rois,AT_TSPO_mediation_Cprime_p_adjusted)

AT_TSPO_mediation_Cprime_p_adjusted_df <- as.data.frame(AT_TSPO_mediation_Cprime_p_adjusted_roi)

# Total

AT_TSPO_mediation_Total_p <- c(fit_Prefrontal_A_T_full3_TSPO_mediation_parameters[fit_Prefrontal_A_T_full3_TSPO_mediation_parameters$label=="total","pvalue"],

fit_Insula_A_T_full3_TSPO_mediation_parameters[fit_Insula_A_T_full3_TSPO_mediation_parameters$label=="total","pvalue"],

fit_G_Cing_A_T_full3_TSPO_mediation_parameters[fit_G_Cing_A_T_full3_TSPO_mediation_parameters$label=="total","pvalue"],

fit_FusiformGyrus_A_T_full3_TSPO_mediation_parameters[fit_FusiformGyrus_A_T_full3_TSPO_mediation_parameters$label=="total","pvalue"],

fit_LingualGyrus_A_T_full3_TSPO_mediation_parameters[fit_LingualGyrus_A_T_full3_TSPO_mediation_parameters$label=="total","pvalue"],

fit_EntorhinalCortex_FS_A_T_full3_TSPO_mediation_parameters[fit_EntorhinalCortex_FS_A_T_full3_TSPO_mediation_parameters$label=="total","pvalue"],

fit_MiddleInfTempGyrus_A_T_full3_TSPO_mediation_parameters[fit_MiddleInfTempGyrus_A_T_full3_TSPO_mediation_parameters$label=="total","pvalue"],

fit_SuperiorTemp_A_T_full3_TSPO_mediation_parameters[fit_SuperiorTemp_A_T_full3_TSPO_mediation_parameters$label=="total","pvalue"],

fit_InferiorParietal_A_T_full3_TSPO_mediation_parameters[fit_InferiorParietal_A_T_full3_TSPO_mediation_parameters$label=="total","pvalue"],

fit_SuperiorParietal_A_T_full3_TSPO_mediation_parameters[fit_SuperiorParietal_A_T_full3_TSPO_mediation_parameters$label=="total","pvalue"],

fit_Amygdala_A_T_full3_TSPO_mediation_parameters[fit_Amygdala_A_T_full3_TSPO_mediation_parameters$label=="total","pvalue"],

fit_Hippocampus_A_T_full3_TSPO_mediation_parameters[fit_Hippocampus_A_T_full3_TSPO_mediation_parameters$label=="total","pvalue"],

fit_Striatum_A_T_full3_TSPO_mediation_parameters[fit_Striatum_A_T_full3_TSPO_mediation_parameters$label=="total","pvalue"])

AT_TSPO_mediation_Total_p_adjusted <- p.adjust(AT_TSPO_mediation_Total_p, method = "BH", n = length(AT_TSPO_mediation_Total_p))

AT_TSPO_mediation_Total_p_adjusted_roi <- cbind(AT_rois,AT_TSPO_mediation_Total_p_adjusted)

AT_TSPO_mediation_Total_p_adjusted_df <- as.data.frame(AT_TSPO_mediation_Total_p_adjusted_roi)

## Tau spreading

# early T -> TSPO -> mid T std

early_to_middle_Braak_full3_TSPO_mediation_std =

"

#Regressions

# specify direct effect, path c'

Braak_III_IV_MK6240_PVC_baseline_scale ~ c*Braak_I_II_MK6240_PVC_baseline_scale + Age_baseline_scale + Sex + BMI_baseline_scale + APOE4_Carrier

# path a

Composite_ER176_PVC_baseline_scale ~ a*Braak_I_II_MK6240_PVC_baseline_scale + TSPO_Affinity_Mixed + TSPO_Affinity_High + Age_baseline_scale + Sex + BMI_baseline_scale + APOE4_Carrier

# path b

Braak_III_IV_MK6240_PVC_baseline_scale ~ b*Composite_ER176_PVC_baseline_scale + APOE4_Carrier

#Defined Parameters:

mediation := a*b

total := c+(a*b)

"

fit_early_to_middle_Braak_full3_TSPO_mediation_std<-sem(early_to_middle_Braak_full3_TSPO_mediation_std,

dat.wide)

fit_early_to_middle_Braak_full3_TSPO_mediation_std_summary <- summary(fit_early_to_middle_Braak_full3_TSPO_mediation_std,

fit.measures=TRUE,standardized=TRUE,rsquare=TRUE)

fit_early_to_middle_Braak_full3_TSPO_mediation_std_parameters <- parameterEstimates(fit_early_to_middle_Braak_full3_TSPO_mediation_std,

ci=TRUE,level=0.95,boot.ci.type="perc", standardized=TRUE)

# mid T -> TSPO -> late T std

middle_to_late_Braak_full3_TSPO_mediation_std =

"

#Regressions

# specify direct effect, path c'

Braak_V_VI_MK6240_PVC_baseline_scale ~ c*Braak_III_IV_MK6240_PVC_baseline_scale + Age_baseline_scale + Sex + BMI_baseline_scale + APOE4_Carrier

# path a

Composite_ER176_PVC_baseline_scale ~ a*Braak_III_IV_MK6240_PVC_baseline_scale + TSPO_Affinity_Mixed + TSPO_Affinity_High + Age_baseline_scale + Sex + BMI_baseline_scale + APOE4_Carrier

# path b

Braak_V_VI_MK6240_PVC_baseline_scale ~ b*Composite_ER176_PVC_baseline_scale + APOE4_Carrier

#Defined Parameters:

mediation := a*b

total := c+(a*b)

"

fit_middle_to_late_Braak_full3_TSPO_mediation_std<-sem(middle_to_late_Braak_full3_TSPO_mediation_std,

dat.wide)

fit_middle_to_late_Braak_full3_TSPO_mediation_std_summary <- summary(fit_middle_to_late_Braak_full3_TSPO_mediation_std,

fit.measures=TRUE,standardized=TRUE,rsquare=TRUE)

fit_middle_to_late_Braak_full3_TSPO_mediation_std_parameters <- parameterEstimates(fit_middle_to_late_Braak_full3_TSPO_mediation_std,

ci=TRUE,level=0.95,boot.ci.type="perc", standardized=TRUE)

## adjust pvalues

TT_rois <- c("early_to_middle","middle_to_late")

# Tau to TSPO

TT_TSPO_mediation_A_p <- c(fit_early_to_middle_Braak_full3_TSPO_mediation_parameters[fit_early_to_middle_Braak_full3_TSPO_mediation_parameters$label=="a","pvalue"],

fit_middle_to_late_Braak_full3_TSPO_mediation_parameters[fit_middle_to_late_Braak_full3_TSPO_mediation_parameters$label=="a","pvalue"])

TT_TSPO_mediation_A_p_adjusted <- p.adjust(TT_TSPO_mediation_A_p, method = "BH", n = length(TT_TSPO_mediation_A_p))

TT_TSPO_mediation_A_p_adjusted_roi <- cbind(TT_rois,TT_TSPO_mediation_A_p_adjusted)

TT_TSPO_mediation_A_p_adjusted_df <- as.data.frame(TT_TSPO_mediation_A_p_adjusted_roi)

# TSPO to Tau

TT_TSPO_mediation_B_p <- c(fit_early_to_middle_Braak_full3_TSPO_mediation_parameters[fit_early_to_middle_Braak_full3_TSPO_mediation_parameters$label=="b","pvalue"],

fit_middle_to_late_Braak_full3_TSPO_mediation_parameters[fit_middle_to_late_Braak_full3_TSPO_mediation_parameters$label=="b","pvalue"])

TT_TSPO_mediation_B_p_adjusted <- p.adjust(TT_TSPO_mediation_B_p, method = "BH", n = length(TT_TSPO_mediation_B_p))

TT_TSPO_mediation_B_p_adjusted_roi <- cbind(TT_rois,TT_TSPO_mediation_B_p_adjusted)

TT_TSPO_mediation_B_p_adjusted_df <- as.data.frame(TT_TSPO_mediation_B_p_adjusted_roi)

# Tau to Tau

TT_TSPO_mediation_C_p <- c(fit_early_to_middle_Braak_full3_TSPO_mediation_parameters[fit_early_to_middle_Braak_full3_TSPO_mediation_parameters$label=="c","pvalue"],

fit_middle_to_late_Braak_full3_TSPO_mediation_parameters[fit_middle_to_late_Braak_full3_TSPO_mediation_parameters$label=="c","pvalue"])

TT_TSPO_mediation_C_p_adjusted <- p.adjust(TT_TSPO_mediation_C_p, method = "BH", n = length(TT_TSPO_mediation_C_p))

TT_TSPO_mediation_C_p_adjusted_roi <- cbind(TT_rois,TT_TSPO_mediation_C_p_adjusted)

TT_TSPO_mediation_C_p_adjusted_df <- as.data.frame(TT_TSPO_mediation_C_p_adjusted_roi)

# Tau to TSPO to Tau

TT_TSPO_mediation_Cprime_p <- c(fit_early_to_middle_Braak_full3_TSPO_mediation_parameters[fit_early_to_middle_Braak_full3_TSPO_mediation_parameters$label=="mediation","pvalue"],

fit_middle_to_late_Braak_full3_TSPO_mediation_parameters[fit_middle_to_late_Braak_full3_TSPO_mediation_parameters$label=="mediation","pvalue"])

TT_TSPO_mediation_Cprime_p_adjusted <- p.adjust(TT_TSPO_mediation_Cprime_p, method = "BH", n = length(TT_TSPO_mediation_Cprime_p))

TT_TSPO_mediation_Cprime_p_adjusted_roi <- cbind(TT_rois,TT_TSPO_mediation_Cprime_p_adjusted)

TT_TSPO_mediation_Cprime_p_adjusted_df <- as.data.frame(TT_TSPO_mediation_Cprime_p_adjusted_roi)

# Total

TT_TSPO_mediation_Total_p <- c(fit_early_to_middle_Braak_full3_TSPO_mediation_parameters[fit_early_to_middle_Braak_full3_TSPO_mediation_parameters$label=="total","pvalue"],

fit_middle_to_late_Braak_full3_TSPO_mediation_parameters[fit_middle_to_late_Braak_full3_TSPO_mediation_parameters$label=="total","pvalue"])

TT_TSPO_mediation_Total_p_adjusted <- p.adjust(TT_TSPO_mediation_Total_p, method = "BH", n = length(TT_TSPO_mediation_Total_p))

TT_TSPO_mediation_Total_p_adjusted_roi <- cbind(TT_rois,TT_TSPO_mediation_Total_p_adjusted)

TT_TSPO_mediation_Total_p_adjusted_df <- as.data.frame(TT_TSPO_mediation_Total_p_adjusted_roi)

# Regional T -> N std

Striatum_T_N_full3_TSPO_mediation_std =

"

#Regressions

# specify direct effect, path c'

Striatum_Volume_MK6240_baseline_scale ~ c*Striatum_MK6240_PVC_baseline_scale + ICV_baseline_scale + Age_baseline_scale + Sex + BMI_baseline_scale + APOE4_Carrier

# path a

Striatum_ER176_PVC_baseline_scale ~ a*Striatum_MK6240_PVC_baseline_scale + TSPO_Affinity_Mixed + TSPO_Affinity_High + Age_baseline_scale + Sex + BMI_baseline_scale + APOE4_Carrier

# path b

Striatum_Volume_MK6240_baseline_scale ~ b*Striatum_ER176_PVC_baseline_scale + APOE4_Carrier

#Defined Parameters:

mediation := a*b

total := c+(a*b)

"

fit_Striatum_T_N_full3_TSPO_mediation_std<-sem(Striatum_T_N_full3_TSPO_mediation_std,

dat.wide)

fit_Striatum_T_N_full3_TSPO_mediation_std_summary <- summary(fit_Striatum_T_N_full3_TSPO_mediation_std,

fit.measures=TRUE,standardized=TRUE,rsquare=TRUE)

fit_Striatum_T_N_full3_TSPO_mediation_std_parameters <- parameterEstimates(fit_Striatum_T_N_full3_TSPO_mediation_std,

ci=TRUE,level=0.95,boot.ci.type="perc",standardized=TRUE)

Amygdala_T_N_full3_TSPO_mediation_std =

"

#Regressions

# specify direct effect, path c'

Amygdala_Volume_MK6240_baseline_scale ~ c*Amygdala_MK6240_PVC_baseline_scale + ICV_baseline_scale + Age_baseline_scale + Sex + BMI_baseline_scale + APOE4_Carrier

# path a

Amygdala_ER176_PVC_baseline_scale ~ a*Amygdala_MK6240_PVC_baseline_scale + TSPO_Affinity_Mixed + TSPO_Affinity_High + Age_baseline_scale + Sex + BMI_baseline_scale + APOE4_Carrier

# path b

Amygdala_Volume_MK6240_baseline_scale ~ b*Amygdala_ER176_PVC_baseline_scale + APOE4_Carrier

#Defined Parameters:

mediation := a*b

total := c+(a*b)

"

fit_Amygdala_T_N_full3_TSPO_mediation_std<-sem(Amygdala_T_N_full3_TSPO_mediation_std,

dat.wide)

fit_Amygdala_T_N_full3_TSPO_mediation_std_summary <- summary(fit_Amygdala_T_N_full3_TSPO_mediation_std,

fit.measures=TRUE,standardized=TRUE,rsquare=TRUE)

fit_Amygdala_T_N_full3_TSPO_mediation_std_parameters <- parameterEstimates(fit_Amygdala_T_N_full3_TSPO_mediation_std,

ci=TRUE,level=0.95,boot.ci.type="perc",standardized=TRUE)

EntorhinalCortex_FS_T_N_full3_TSPO_mediation_std =

"

#Regressions

# specify direct effect, path c'

EntorhinalCortex_FS_Volume_MK6240_baseline_scale ~ c*EntorhinalCortex_FS_MK6240_PVC_baseline_scale + ICV_baseline_scale + Age_baseline_scale + Sex + BMI_baseline_scale + APOE4_Carrier

# path a

EntorhinalCortex_FS_ER176_PVC_baseline_scale ~ a*EntorhinalCortex_FS_MK6240_PVC_baseline_scale + TSPO_Affinity_Mixed + TSPO_Affinity_High + Age_baseline_scale + Sex + BMI_baseline_scale + APOE4_Carrier

# path b

EntorhinalCortex_FS_Volume_MK6240_baseline_scale ~ b*EntorhinalCortex_FS_ER176_PVC_baseline_scale + APOE4_Carrier

#Defined Parameters:

mediation := a*b

total := c+(a*b)

"

fit_EntorhinalCortex_FS_T_N_full3_TSPO_mediation_std<-sem(EntorhinalCortex_FS_T_N_full3_TSPO_mediation_std,

dat.wide)

fit_EntorhinalCortex_FS_T_N_full3_TSPO_mediation_std_summary <- summary(fit_EntorhinalCortex_FS_T_N_full3_TSPO_mediation_std,

fit.measures=TRUE,standardized=TRUE,rsquare=TRUE)

fit_EntorhinalCortex_FS_T_N_full3_TSPO_mediation_std_parameters <- parameterEstimates(fit_EntorhinalCortex_FS_T_N_full3_TSPO_mediation_std,

ci=TRUE,level=0.95,boot.ci.type="perc",standardized=TRUE)

FusiformGyrus_T_N_full3_TSPO_mediation_std =

"

#Regressions

# specify direct effect, path c'

FusiformGyrus_Volume_MK6240_baseline_scale ~ c*FusiformGyrus_MK6240_PVC_baseline_scale + ICV_baseline_scale + Age_baseline_scale + Sex + BMI_baseline_scale + APOE4_Carrier

# path a

FusiformGyrus_ER176_PVC_baseline_scale ~ a*FusiformGyrus_MK6240_PVC_baseline_scale + TSPO_Affinity_Mixed + TSPO_Affinity_High + Age_baseline_scale + Sex + BMI_baseline_scale + APOE4_Carrier

# path b

FusiformGyrus_Volume_MK6240_baseline_scale ~ b*FusiformGyrus_ER176_PVC_baseline_scale + APOE4_Carrier

#Defined Parameters:

mediation := a*b

total := c+(a*b)

"

fit_FusiformGyrus_T_N_full3_TSPO_mediation_std<-sem(FusiformGyrus_T_N_full3_TSPO_mediation_std,

dat.wide)

fit_FusiformGyrus_T_N_full3_TSPO_mediation_std_summary <- summary(fit_FusiformGyrus_T_N_full3_TSPO_mediation_std,

fit.measures=TRUE,standardized=TRUE,rsquare=TRUE)

fit_FusiformGyrus_T_N_full3_TSPO_mediation_std_parameters <- parameterEstimates(fit_FusiformGyrus_T_N_full3_TSPO_mediation_std,

ci=TRUE,level=0.95,boot.ci.type="perc",standardized=TRUE)

G_Cing_T_N_full3_TSPO_mediation_std =

"

#Regressions

# specify direct effect, path c'

G_Cing_Volume_MK6240_baseline_scale ~ c*G_Cing_MK6240_PVC_baseline_scale + ICV_baseline_scale + Age_baseline_scale + Sex + BMI_baseline_scale + APOE4_Carrier

# path a

G_Cing_ER176_PVC_baseline_scale ~ a*G_Cing_MK6240_PVC_baseline_scale + TSPO_Affinity_Mixed + TSPO_Affinity_High + Age_baseline_scale + Sex + BMI_baseline_scale + APOE4_Carrier

# path b

G_Cing_Volume_MK6240_baseline_scale ~ b*G_Cing_ER176_PVC_baseline_scale + APOE4_Carrier

#Defined Parameters:

mediation := a*b

total := c+(a*b)

"

fit_G_Cing_T_N_full3_TSPO_mediation_std<-sem(G_Cing_T_N_full3_TSPO_mediation_std,

dat.wide)

fit_G_Cing_T_N_full3_TSPO_mediation_std_summary <- summary(fit_G_Cing_T_N_full3_TSPO_mediation_std,

fit.measures=TRUE,standardized=TRUE,rsquare=TRUE)

fit_G_Cing_T_N_full3_TSPO_mediation_std_parameters <- parameterEstimates(fit_G_Cing_T_N_full3_TSPO_mediation_std,

ci=TRUE,level=0.95,boot.ci.type="perc",standardized=TRUE)

Hippocampus_T_N_full3_TSPO_mediation_std =

"

#Regressions

# specify direct effect, path c'

Hippocampus_Volume_MK6240_baseline_scale ~ c*Hippocampus_MK6240_PVC_baseline_scale + ICV_baseline_scale + Age_baseline_scale + Sex + BMI_baseline_scale + APOE4_Carrier

# path a

Hippocampus_ER176_PVC_baseline_scale ~ a*Hippocampus_MK6240_PVC_baseline_scale + TSPO_Affinity_Mixed + TSPO_Affinity_High + Age_baseline_scale + Sex + BMI_baseline_scale + APOE4_Carrier

# path b

Hippocampus_Volume_MK6240_baseline_scale ~ b*Hippocampus_ER176_PVC_baseline_scale + APOE4_Carrier

#Defined Parameters:

mediation := a*b

total := c+(a*b)

"

fit_Hippocampus_T_N_full3_TSPO_mediation_std<-sem(Hippocampus_T_N_full3_TSPO_mediation_std,

dat.wide)

fit_Hippocampus_T_N_full3_TSPO_mediation_std_summary <- summary(fit_Hippocampus_T_N_full3_TSPO_mediation_std,

fit.measures=TRUE,standardized=TRUE,rsquare=TRUE)

fit_Hippocampus_T_N_full3_TSPO_mediation_std_parameters <- parameterEstimates(fit_Hippocampus_T_N_full3_TSPO_mediation_std,

ci=TRUE,level=0.95,boot.ci.type="perc",standardized=TRUE)

InferiorParietal_T_N_full3_TSPO_mediation_std =

"

#Regressions

# specify direct effect, path c'

InferiorParietal_Volume_MK6240_baseline_scale ~ c*InferiorParietal_MK6240_PVC_baseline_scale + ICV_baseline_scale + Age_baseline_scale + Sex + BMI_baseline_scale + APOE4_Carrier

# path a

InferiorParietal_ER176_PVC_baseline_scale ~ a*InferiorParietal_MK6240_PVC_baseline_scale + TSPO_Affinity_Mixed + TSPO_Affinity_High + Age_baseline_scale + Sex + BMI_baseline_scale + APOE4_Carrier

# path b

InferiorParietal_Volume_MK6240_baseline_scale ~ b*InferiorParietal_ER176_PVC_baseline_scale + APOE4_Carrier

#Defined Parameters:

mediation := a*b

total := c+(a*b)

"

fit_InferiorParietal_T_N_full3_TSPO_mediation_std<-sem(InferiorParietal_T_N_full3_TSPO_mediation_std,

dat.wide)

fit_InferiorParietal_T_N_full3_TSPO_mediation_std_summary <- summary(fit_InferiorParietal_T_N_full3_TSPO_mediation_std,

fit.measures=TRUE,standardized=TRUE,rsquare=TRUE)

fit_InferiorParietal_T_N_full3_TSPO_mediation_std_parameters <- parameterEstimates(fit_InferiorParietal_T_N_full3_TSPO_mediation_std,

ci=TRUE,level=0.95,boot.ci.type="perc",standardized=TRUE)

Insula_T_N_full3_TSPO_mediation_std =

"

#Regressions

# specify direct effect, path c'

Insula_Volume_MK6240_baseline_scale ~ c*Insula_MK6240_PVC_baseline_scale + ICV_baseline_scale + Age_baseline_scale + Sex + BMI_baseline_scale + APOE4_Carrier

# path a

Insula_ER176_PVC_baseline_scale ~ a*Insula_MK6240_PVC_baseline_scale + TSPO_Affinity_Mixed + TSPO_Affinity_High + Age_baseline_scale + Sex + BMI_baseline_scale + APOE4_Carrier

# path b

Insula_Volume_MK6240_baseline_scale ~ b*Insula_ER176_PVC_baseline_scale + APOE4_Carrier

#Defined Parameters:

mediation := a*b

total := c+(a*b)

"

fit_Insula_T_N_full3_TSPO_mediation_std<-sem(Insula_T_N_full3_TSPO_mediation_std,

dat.wide)

fit_Insula_T_N_full3_TSPO_mediation_std_summary <- summary(fit_Insula_T_N_full3_TSPO_mediation_std,

fit.measures=TRUE,standardized=TRUE,rsquare=TRUE)

fit_Insula_T_N_full3_TSPO_mediation_std_parameters <- parameterEstimates(fit_Insula_T_N_full3_TSPO_mediation_std,

ci=TRUE,level=0.95,boot.ci.type="perc",standardized=TRUE)

LingualGyrus_T_N_full3_TSPO_mediation_std =

"

#Regressions

# specify direct effect, path c'

LingualGyrus_Volume_MK6240_baseline_scale ~ c*LingualGyrus_MK6240_PVC_baseline_scale + ICV_baseline_scale + Age_baseline_scale + Sex + BMI_baseline_scale + APOE4_Carrier

# path a

LingualGyrus_ER176_PVC_baseline_scale ~ a*LingualGyrus_MK6240_PVC_baseline_scale + TSPO_Affinity_Mixed + TSPO_Affinity_High + Age_baseline_scale + Sex + BMI_baseline_scale + APOE4_Carrier

# path b

LingualGyrus_Volume_MK6240_baseline_scale ~ b*LingualGyrus_ER176_PVC_baseline_scale + APOE4_Carrier

#Defined Parameters:

mediation := a*b

total := c+(a*b)

"

fit_LingualGyrus_T_N_full3_TSPO_mediation_std<-sem(LingualGyrus_T_N_full3_TSPO_mediation_std,

dat.wide)

fit_LingualGyrus_T_N_full3_TSPO_mediation_std_summary <- summary(fit_LingualGyrus_T_N_full3_TSPO_mediation_std,

fit.measures=TRUE,standardized=TRUE,rsquare=TRUE)

fit_LingualGyrus_T_N_full3_TSPO_mediation_std_parameters <- parameterEstimates(fit_LingualGyrus_T_N_full3_TSPO_mediation_std,

ci=TRUE,level=0.95,boot.ci.type="perc",standardized=TRUE)

MiddleInfTempGyrus_T_N_full3_TSPO_mediation_std =

"

#Regressions

# specify direct effect, path c'

MiddleInfTempGyrus_Volume_MK6240_baseline_scale ~ c*MiddleInfTempGyrus_MK6240_PVC_baseline_scale + ICV_baseline_scale + Age_baseline_scale + Sex + BMI_baseline_scale + APOE4_Carrier

# path a

MiddleInfTempGyrus_ER176_PVC_baseline_scale ~ a*MiddleInfTempGyrus_MK6240_PVC_baseline_scale + TSPO_Affinity_Mixed + TSPO_Affinity_High + Age_baseline_scale + Sex + BMI_baseline_scale + APOE4_Carrier

# path b

MiddleInfTempGyrus_Volume_MK6240_baseline_scale ~ b*MiddleInfTempGyrus_ER176_PVC_baseline_scale + APOE4_Carrier

#Defined Parameters:

mediation := a*b

total := c+(a*b)

"

fit_MiddleInfTempGyrus_T_N_full3_TSPO_mediation_std<-sem(MiddleInfTempGyrus_T_N_full3_TSPO_mediation_std,

dat.wide)

fit_MiddleInfTempGyrus_T_N_full3_TSPO_mediation_std_summary <- summary(fit_MiddleInfTempGyrus_T_N_full3_TSPO_mediation_std,

fit.measures=TRUE,standardized=TRUE,rsquare=TRUE)

fit_MiddleInfTempGyrus_T_N_full3_TSPO_mediation_std_parameters <- parameterEstimates(fit_MiddleInfTempGyrus_T_N_full3_TSPO_mediation_std,

ci=TRUE,level=0.95,boot.ci.type="perc",standardized=TRUE)

Prefrontal_T_N_full3_TSPO_mediation_std =

"

#Regressions

# specify direct effect, path c'

Prefrontal_Volume_MK6240_baseline_scale ~ c*Prefrontal_MK6240_PVC_baseline_scale + ICV_baseline_scale + Age_baseline_scale + Sex + BMI_baseline_scale + APOE4_Carrier

# path a

Prefrontal_ER176_PVC_baseline_scale ~ a*Prefrontal_MK6240_PVC_baseline_scale + TSPO_Affinity_Mixed + TSPO_Affinity_High + Age_baseline_scale + Sex + BMI_baseline_scale + APOE4_Carrier

# path b

Prefrontal_Volume_MK6240_baseline_scale ~ b*Prefrontal_ER176_PVC_baseline_scale + APOE4_Carrier

#Defined Parameters:

mediation := a*b

total := c+(a*b)

"

fit_Prefrontal_T_N_full3_TSPO_mediation_std<-sem(Prefrontal_T_N_full3_TSPO_mediation_std,

dat.wide)

fit_Prefrontal_T_N_full3_TSPO_mediation_std_summary <- summary(fit_Prefrontal_T_N_full3_TSPO_mediation_std,

fit.measures=TRUE,standardized=TRUE,rsquare=TRUE)

fit_Prefrontal_T_N_full3_TSPO_mediation_std_parameters <- parameterEstimates(fit_Prefrontal_T_N_full3_TSPO_mediation_std,

ci=TRUE,level=0.95,boot.ci.type="perc",standardized=TRUE)

SuperiorParietal_T_N_full3_TSPO_mediation_std =

"

#Regressions

# specify direct effect, path c'

SuperiorParietal_Volume_MK6240_baseline_scale ~ c*SuperiorParietal_MK6240_PVC_baseline_scale + ICV_baseline_scale + Age_baseline_scale + Sex + BMI_baseline_scale + APOE4_Carrier

# path a

SuperiorParietal_ER176_PVC_baseline_scale ~ a*SuperiorParietal_MK6240_PVC_baseline_scale + TSPO_Affinity_Mixed + TSPO_Affinity_High + Age_baseline_scale + Sex + BMI_baseline_scale + APOE4_Carrier

# path b

SuperiorParietal_Volume_MK6240_baseline_scale ~ b*SuperiorParietal_ER176_PVC_baseline_scale + APOE4_Carrier

#Defined Parameters:

mediation := a*b

total := c+(a*b)

"

fit_SuperiorParietal_T_N_full3_TSPO_mediation_std<-sem(SuperiorParietal_T_N_full3_TSPO_mediation_std,

dat.wide)

fit_SuperiorParietal_T_N_full3_TSPO_mediation_std_summary <- summary(fit_SuperiorParietal_T_N_full3_TSPO_mediation_std,

fit.measures=TRUE,standardized=TRUE,rsquare=TRUE)

fit_SuperiorParietal_T_N_full3_TSPO_mediation_std_parameters <- parameterEstimates(fit_SuperiorParietal_T_N_full3_TSPO_mediation_std,

ci=TRUE,level=0.95,boot.ci.type="perc",standardized=TRUE)

SuperiorTemp_T_N_full3_TSPO_mediation_std =

"

#Regressions

# specify direct effect, path c'

SuperiorTemp_Volume_MK6240_baseline_scale ~ c*SuperiorTemp_MK6240_PVC_baseline_scale + ICV_baseline_scale + Age_baseline_scale + Sex + BMI_baseline_scale + APOE4_Carrier

# path a

SuperiorTemp_ER176_PVC_baseline_scale ~ a*SuperiorTemp_MK6240_PVC_baseline_scale + TSPO_Affinity_Mixed + TSPO_Affinity_High + Age_baseline_scale + Sex + BMI_baseline_scale + APOE4_Carrier

# path b

SuperiorTemp_Volume_MK6240_baseline_scale ~ b*SuperiorTemp_ER176_PVC_baseline_scale + APOE4_Carrier

#Defined Parameters:

mediation := a*b

total := c+(a*b)

"

fit_SuperiorTemp_T_N_full3_TSPO_mediation_std<-sem(SuperiorTemp_T_N_full3_TSPO_mediation_std,

dat.wide)

fit_SuperiorTemp_T_N_full3_TSPO_mediation_std_summary <- summary(fit_SuperiorTemp_T_N_full3_TSPO_mediation_std,

fit.measures=TRUE,standardized=TRUE,rsquare=TRUE)

fit_SuperiorTemp_T_N_full3_TSPO_mediation_std_parameters <- parameterEstimates(fit_SuperiorTemp_T_N_full3_TSPO_mediation_std,

ci=TRUE,level=0.95,boot.ci.type="perc",standardized=TRUE)

## adjust p values

TN_rois <- c("Prefrontal","Insula","G_Cing","FusiformGyrus","LingualGyrus",

"EntorhinalCortex_FS","MiddleInfTempGyrus","SuperiorTemp",

"InferiorParietal","SuperiorParietal",

"Amygdala","Hippocampus","Striatum")

# Tau to TSPO

TN_TSPO_mediation_A_p <- c(fit_Prefrontal_T_N_full3_TSPO_mediation_parameters[fit_Prefrontal_T_N_full3_TSPO_mediation_parameters$label=="a","pvalue"],

fit_Insula_T_N_full3_TSPO_mediation_parameters[fit_Insula_T_N_full3_TSPO_mediation_parameters$label=="a","pvalue"],

fit_G_Cing_T_N_full3_TSPO_mediation_parameters[fit_G_Cing_T_N_full3_TSPO_mediation_parameters$label=="a","pvalue"],

fit_FusiformGyrus_T_N_full3_TSPO_mediation_parameters[fit_FusiformGyrus_T_N_full3_TSPO_mediation_parameters$label=="a","pvalue"],

fit_LingualGyrus_T_N_full3_TSPO_mediation_parameters[fit_LingualGyrus_T_N_full3_TSPO_mediation_parameters$label=="a","pvalue"],

fit_EntorhinalCortex_FS_T_N_full3_TSPO_mediation_parameters[fit_EntorhinalCortex_FS_T_N_full3_TSPO_mediation_parameters$label=="a","pvalue"],

fit_MiddleInfTempGyrus_T_N_full3_TSPO_mediation_parameters[fit_MiddleInfTempGyrus_T_N_full3_TSPO_mediation_parameters$label=="a","pvalue"],

fit_SuperiorTemp_T_N_full3_TSPO_mediation_parameters[fit_SuperiorTemp_T_N_full3_TSPO_mediation_parameters$label=="a","pvalue"],

fit_InferiorParietal_T_N_full3_TSPO_mediation_parameters[fit_InferiorParietal_T_N_full3_TSPO_mediation_parameters$label=="a","pvalue"],

fit_SuperiorParietal_T_N_full3_TSPO_mediation_parameters[fit_SuperiorParietal_T_N_full3_TSPO_mediation_parameters$label=="a","pvalue"],

fit_Amygdala_T_N_full3_TSPO_mediation_parameters[fit_Amygdala_T_N_full3_TSPO_mediation_parameters$label=="a","pvalue"],

fit_Hippocampus_T_N_full3_TSPO_mediation_parameters[fit_Hippocampus_T_N_full3_TSPO_mediation_parameters$label=="a","pvalue"],

fit_Striatum_T_N_full3_TSPO_mediation_parameters[fit_Striatum_T_N_full3_TSPO_mediation_parameters$label=="a","pvalue"])

TN_TSPO_mediation_A_p_adjusted <- p.adjust(TN_TSPO_mediation_A_p, method = "BH", n = length(TN_TSPO_mediation_A_p))

TN_TSPO_mediation_A_p_adjusted_roi <- cbind(TN_rois,TN_TSPO_mediation_A_p_adjusted)

TN_TSPO_mediation_A_p_adjusted_df <- as.data.frame(TN_TSPO_mediation_A_p_adjusted_roi)

# TSPO to Neurodegeneration

TN_TSPO_mediation_B_p <- c(fit_Prefrontal_T_N_full3_TSPO_mediation_parameters[fit_Prefrontal_T_N_full3_TSPO_mediation_parameters$label=="b","pvalue"],

fit_Insula_T_N_full3_TSPO_mediation_parameters[fit_Insula_T_N_full3_TSPO_mediation_parameters$label=="b","pvalue"],

fit_G_Cing_T_N_full3_TSPO_mediation_parameters[fit_G_Cing_T_N_full3_TSPO_mediation_parameters$label=="b","pvalue"],

fit_FusiformGyrus_T_N_full3_TSPO_mediation_parameters[fit_FusiformGyrus_T_N_full3_TSPO_mediation_parameters$label=="b","pvalue"],

fit_LingualGyrus_T_N_full3_TSPO_mediation_parameters[fit_LingualGyrus_T_N_full3_TSPO_mediation_parameters$label=="b","pvalue"],

fit_EntorhinalCortex_FS_T_N_full3_TSPO_mediation_parameters[fit_EntorhinalCortex_FS_T_N_full3_TSPO_mediation_parameters$label=="b","pvalue"],

fit_MiddleInfTempGyrus_T_N_full3_TSPO_mediation_parameters[fit_MiddleInfTempGyrus_T_N_full3_TSPO_mediation_parameters$label=="b","pvalue"],

fit_SuperiorTemp_T_N_full3_TSPO_mediation_parameters[fit_SuperiorTemp_T_N_full3_TSPO_mediation_parameters$label=="b","pvalue"],

fit_InferiorParietal_T_N_full3_TSPO_mediation_parameters[fit_InferiorParietal_T_N_full3_TSPO_mediation_parameters$label=="b","pvalue"],

fit_SuperiorParietal_T_N_full3_TSPO_mediation_parameters[fit_SuperiorParietal_T_N_full3_TSPO_mediation_parameters$label=="b","pvalue"],

fit_Amygdala_T_N_full3_TSPO_mediation_parameters[fit_Amygdala_T_N_full3_TSPO_mediation_parameters$label=="b","pvalue"],

fit_Hippocampus_T_N_full3_TSPO_mediation_parameters[fit_Hippocampus_T_N_full3_TSPO_mediation_parameters$label=="b","pvalue"],

fit_Striatum_T_N_full3_TSPO_mediation_parameters[fit_Striatum_T_N_full3_TSPO_mediation_parameters$label=="b","pvalue"])

TN_TSPO_mediation_B_p_adjusted <- p.adjust(TN_TSPO_mediation_B_p, method = "BH", n = length(TN_TSPO_mediation_B_p))

TN_TSPO_mediation_B_p_adjusted_roi <- cbind(TN_rois,TN_TSPO_mediation_B_p_adjusted)

TN_TSPO_mediation_B_p_adjusted_df <- as.data.frame(TN_TSPO_mediation_B_p_adjusted_roi)

# Tau to Neurodegeneration

TN_TSPO_mediation_C_p <- c(fit_Prefrontal_T_N_full3_TSPO_mediation_parameters[fit_Prefrontal_T_N_full3_TSPO_mediation_parameters$label=="c","pvalue"],

fit_Insula_T_N_full3_TSPO_mediation_parameters[fit_Insula_T_N_full3_TSPO_mediation_parameters$label=="c","pvalue"],

fit_G_Cing_T_N_full3_TSPO_mediation_parameters[fit_G_Cing_T_N_full3_TSPO_mediation_parameters$label=="c","pvalue"],

fit_FusiformGyrus_T_N_full3_TSPO_mediation_parameters[fit_FusiformGyrus_T_N_full3_TSPO_mediation_parameters$label=="c","pvalue"],

fit_LingualGyrus_T_N_full3_TSPO_mediation_parameters[fit_LingualGyrus_T_N_full3_TSPO_mediation_parameters$label=="c","pvalue"],

fit_EntorhinalCortex_FS_T_N_full3_TSPO_mediation_parameters[fit_EntorhinalCortex_FS_T_N_full3_TSPO_mediation_parameters$label=="c","pvalue"],

fit_MiddleInfTempGyrus_T_N_full3_TSPO_mediation_parameters[fit_MiddleInfTempGyrus_T_N_full3_TSPO_mediation_parameters$label=="c","pvalue"],

fit_SuperiorTemp_T_N_full3_TSPO_mediation_parameters[fit_SuperiorTemp_T_N_full3_TSPO_mediation_parameters$label=="c","pvalue"],

fit_InferiorParietal_T_N_full3_TSPO_mediation_parameters[fit_InferiorParietal_T_N_full3_TSPO_mediation_parameters$label=="c","pvalue"],

fit_SuperiorParietal_T_N_full3_TSPO_mediation_parameters[fit_SuperiorParietal_T_N_full3_TSPO_mediation_parameters$label=="c","pvalue"],

fit_Amygdala_T_N_full3_TSPO_mediation_parameters[fit_Amygdala_T_N_full3_TSPO_mediation_parameters$label=="c","pvalue"],

fit_Hippocampus_T_N_full3_TSPO_mediation_parameters[fit_Hippocampus_T_N_full3_TSPO_mediation_parameters$label=="c","pvalue"],

fit_Striatum_T_N_full3_TSPO_mediation_parameters[fit_Striatum_T_N_full3_TSPO_mediation_parameters$label=="c","pvalue"])

TN_TSPO_mediation_C_p_adjusted <- p.adjust(TN_TSPO_mediation_C_p, method = "BH", n = length(TN_TSPO_mediation_C_p))

TN_TSPO_mediation_C_p_adjusted_roi <- cbind(TN_rois,TN_TSPO_mediation_C_p_adjusted)

TN_TSPO_mediation_C_p_adjusted_df <- as.data.frame(TN_TSPO_mediation_C_p_adjusted_roi)

# Tau to TSPO to Neurodegeneration

TN_TSPO_mediation_Cprime_p <- c(fit_Prefrontal_T_N_full3_TSPO_mediation_parameters[fit_Prefrontal_T_N_full3_TSPO_mediation_parameters$label=="mediation","pvalue"],

fit_Insula_T_N_full3_TSPO_mediation_parameters[fit_Insula_T_N_full3_TSPO_mediation_parameters$label=="mediation","pvalue"],

fit_G_Cing_T_N_full3_TSPO_mediation_parameters[fit_G_Cing_T_N_full3_TSPO_mediation_parameters$label=="mediation","pvalue"],

fit_FusiformGyrus_T_N_full3_TSPO_mediation_parameters[fit_FusiformGyrus_T_N_full3_TSPO_mediation_parameters$label=="mediation","pvalue"],

fit_LingualGyrus_T_N_full3_TSPO_mediation_parameters[fit_LingualGyrus_T_N_full3_TSPO_mediation_parameters$label=="mediation","pvalue"],

fit_EntorhinalCortex_FS_T_N_full3_TSPO_mediation_parameters[fit_EntorhinalCortex_FS_T_N_full3_TSPO_mediation_parameters$label=="mediation","pvalue"],

fit_MiddleInfTempGyrus_T_N_full3_TSPO_mediation_parameters[fit_MiddleInfTempGyrus_T_N_full3_TSPO_mediation_parameters$label=="mediation","pvalue"],

fit_SuperiorTemp_T_N_full3_TSPO_mediation_parameters[fit_SuperiorTemp_T_N_full3_TSPO_mediation_parameters$label=="mediation","pvalue"],

fit_InferiorParietal_T_N_full3_TSPO_mediation_parameters[fit_InferiorParietal_T_N_full3_TSPO_mediation_parameters$label=="mediation","pvalue"],

fit_SuperiorParietal_T_N_full3_TSPO_mediation_parameters[fit_SuperiorParietal_T_N_full3_TSPO_mediation_parameters$label=="mediation","pvalue"],

fit_Amygdala_T_N_full3_TSPO_mediation_parameters[fit_Amygdala_T_N_full3_TSPO_mediation_parameters$label=="mediation","pvalue"],

fit_Hippocampus_T_N_full3_TSPO_mediation_parameters[fit_Hippocampus_T_N_full3_TSPO_mediation_parameters$label=="mediation","pvalue"],

fit_Striatum_T_N_full3_TSPO_mediation_parameters[fit_Striatum_T_N_full3_TSPO_mediation_parameters$label=="mediation","pvalue"])

TN_TSPO_mediation_Cprime_p_adjusted <- p.adjust(TN_TSPO_mediation_Cprime_p, method = "BH", n = length(TN_TSPO_mediation_Cprime_p))

TN_TSPO_mediation_Cprime_p_adjusted_roi <- cbind(TN_rois,TN_TSPO_mediation_Cprime_p_adjusted)

TN_TSPO_mediation_Cprime_p_adjusted_df <- as.data.frame(TN_TSPO_mediation_Cprime_p_adjusted_roi)

# Total

TN_TSPO_mediation_Total_p <- c(fit_Prefrontal_T_N_full3_TSPO_mediation_parameters[fit_Prefrontal_T_N_full3_TSPO_mediation_parameters$label=="total","pvalue"],

fit_Insula_T_N_full3_TSPO_mediation_parameters[fit_Insula_T_N_full3_TSPO_mediation_parameters$label=="total","pvalue"],

fit_G_Cing_T_N_full3_TSPO_mediation_parameters[fit_G_Cing_T_N_full3_TSPO_mediation_parameters$label=="total","pvalue"],

fit_FusiformGyrus_T_N_full3_TSPO_mediation_parameters[fit_FusiformGyrus_T_N_full3_TSPO_mediation_parameters$label=="total","pvalue"],

fit_LingualGyrus_T_N_full3_TSPO_mediation_parameters[fit_LingualGyrus_T_N_full3_TSPO_mediation_parameters$label=="total","pvalue"],

fit_EntorhinalCortex_FS_T_N_full3_TSPO_mediation_parameters[fit_EntorhinalCortex_FS_T_N_full3_TSPO_mediation_parameters$label=="total","pvalue"],

fit_MiddleInfTempGyrus_T_N_full3_TSPO_mediation_parameters[fit_MiddleInfTempGyrus_T_N_full3_TSPO_mediation_parameters$label=="total","pvalue"],

fit_SuperiorTemp_T_N_full3_TSPO_mediation_parameters[fit_SuperiorTemp_T_N_full3_TSPO_mediation_parameters$label=="total","pvalue"],

fit_InferiorParietal_T_N_full3_TSPO_mediation_parameters[fit_InferiorParietal_T_N_full3_TSPO_mediation_parameters$label=="total","pvalue"],

fit_SuperiorParietal_T_N_full3_TSPO_mediation_parameters[fit_SuperiorParietal_T_N_full3_TSPO_mediation_parameters$label=="total","pvalue"],

fit_Amygdala_T_N_full3_TSPO_mediation_parameters[fit_Amygdala_T_N_full3_TSPO_mediation_parameters$label=="total","pvalue"],

fit_Hippocampus_T_N_full3_TSPO_mediation_parameters[fit_Hippocampus_T_N_full3_TSPO_mediation_parameters$label=="total","pvalue"],

fit_Striatum_T_N_full3_TSPO_mediation_parameters[fit_Striatum_T_N_full3_TSPO_mediation_parameters$label=="total","pvalue"])

TN_TSPO_mediation_Total_p_adjusted <- p.adjust(TN_TSPO_mediation_Total_p, method = "BH", n = length(TN_TSPO_mediation_Total_p))

TN_TSPO_mediation_Total_p_adjusted_roi <- cbind(TN_rois,TN_TSPO_mediation_Total_p_adjusted)

TN_TSPO_mediation_Total_p_adjusted_df <- as.data.frame(TN_TSPO_mediation_Total_p_adjusted_roi)

## opposite mediations

# Regional A -> T std

Striatum_A_T_full3_TSPO_mediation_std_flipped =

"

#Regressions

# specify direct effect, path c' (Y~X)

Striatum_MK6240_PVC_baseline_scale ~ c*Striatum_ER176_PVC_baseline_scale + TSPO_Affinity_Mixed + TSPO_Affinity_High + Age_baseline_scale + Sex + BMI_baseline_scale + APOE4_Carrier

# path a (M~X)

Striatum_FBB_baseline_scale ~ a*Striatum_ER176_PVC_baseline_scale + TSPO_Affinity_Mixed + TSPO_Affinity_High + Age_baseline_scale + Sex + BMI_baseline_scale + APOE4_Carrier

# path b (Y~M)

Striatum_MK6240_PVC_baseline_scale ~ b*Striatum_FBB_baseline_scale + APOE4_Carrier

#Defined Parameters:

mediation := a*b

total := c+(a*b)

"

fit_Striatum_A_T_full3_TSPO_mediation_std_flipped<-sem(Striatum_A_T_full3_TSPO_mediation_std_flipped,

dat.wide)

fit_Striatum_A_T_full3_TSPO_mediation_std_flipped_summary <- summary(fit_Striatum_A_T_full3_TSPO_mediation_std_flipped,

fit.measures=TRUE,standardized=TRUE,rsquare=TRUE)

fit_Striatum_A_T_full3_TSPO_mediation_std_flipped_parameters <- parameterEstimates(fit_Striatum_A_T_full3_TSPO_mediation_std_flipped,

ci=TRUE,level=0.95,boot.ci.type="perc",standardized=TRUE)

Amygdala_A_T_full3_TSPO_mediation_std_flipped =

"

#Regressions

# specify direct effect, path c' (Y~X)

Amygdala_MK6240_PVC_baseline_scale ~ c*Amygdala_ER176_PVC_baseline_scale + TSPO_Affinity_Mixed + TSPO_Affinity_High + Age_baseline_scale + Sex + BMI_baseline_scale + APOE4_Carrier

# path a (M~X)

Amygdala_FBB_baseline_scale ~ a*Amygdala_ER176_PVC_baseline_scale + TSPO_Affinity_Mixed + TSPO_Affinity_High + Age_baseline_scale + Sex + BMI_baseline_scale + APOE4_Carrier

# path b (Y~M)

Amygdala_MK6240_PVC_baseline_scale ~ b*Amygdala_FBB_baseline_scale + APOE4_Carrier

#Defined Parameters:

mediation := a*b

total := c+(a*b)

"

fit_Amygdala_A_T_full3_TSPO_mediation_std_flipped<-sem(Amygdala_A_T_full3_TSPO_mediation_std_flipped,

dat.wide)

fit_Amygdala_A_T_full3_TSPO_mediation_std_flipped_summary <- summary(fit_Amygdala_A_T_full3_TSPO_mediation_std_flipped,

fit.measures=TRUE,standardized=TRUE,rsquare=TRUE)

fit_Amygdala_A_T_full3_TSPO_mediation_std_flipped_parameters <- parameterEstimates(fit_Amygdala_A_T_full3_TSPO_mediation_std_flipped,

ci=TRUE,level=0.95,boot.ci.type="perc",standardized=TRUE)

EntorhinalCortex_FS_A_T_full3_TSPO_mediation_std_flipped =

"

#Regressions

# specify direct effect, path c' (Y~X)

EntorhinalCortex_FS_MK6240_PVC_baseline_scale ~ c*EntorhinalCortex_FS_ER176_PVC_baseline_scale + TSPO_Affinity_Mixed + TSPO_Affinity_High + Age_baseline_scale + Sex + BMI_baseline_scale + APOE4_Carrier

# path a (M~X)

EntorhinalCortex_FS_FBB_baseline_scale ~ a*EntorhinalCortex_FS_ER176_PVC_baseline_scale + TSPO_Affinity_Mixed + TSPO_Affinity_High + Age_baseline_scale + Sex + BMI_baseline_scale + APOE4_Carrier

# path b (Y~M)

EntorhinalCortex_FS_MK6240_PVC_baseline_scale ~ b*EntorhinalCortex_FS_FBB_baseline_scale + APOE4_Carrier

#Defined Parameters:

mediation := a*b

total := c+(a*b)

"

fit_EntorhinalCortex_FS_A_T_full3_TSPO_mediation_std_flipped<-sem(EntorhinalCortex_FS_A_T_full3_TSPO_mediation_std_flipped,

dat.wide)

fit_EntorhinalCortex_FS_A_T_full3_TSPO_mediation_std_flipped_summary <- summary(fit_EntorhinalCortex_FS_A_T_full3_TSPO_mediation_std_flipped,

fit.measures=TRUE,standardized=TRUE,rsquare=TRUE)

fit_EntorhinalCortex_FS_A_T_full3_TSPO_mediation_std_flipped_parameters <- parameterEstimates(fit_EntorhinalCortex_FS_A_T_full3_TSPO_mediation_std_flipped,

ci=TRUE,level=0.95,boot.ci.type="perc",standardized=TRUE)

FusiformGyrus_A_T_full3_TSPO_mediation_std_flipped =

"

#Regressions

# specify direct effect, path c' (Y~X)

FusiformGyrus_MK6240_PVC_baseline_scale ~ c*FusiformGyrus_ER176_PVC_baseline_scale + TSPO_Affinity_Mixed + TSPO_Affinity_High + Age_baseline_scale + Sex + BMI_baseline_scale + APOE4_Carrier

# path a (M~X)

FusiformGyrus_FBB_baseline_scale ~ a*FusiformGyrus_ER176_PVC_baseline_scale + TSPO_Affinity_Mixed + TSPO_Affinity_High + Age_baseline_scale + Sex + BMI_baseline_scale + APOE4_Carrier

# path b (Y~M)

FusiformGyrus_MK6240_PVC_baseline_scale ~ b*FusiformGyrus_FBB_baseline_scale + APOE4_Carrier

#Defined Parameters:

mediation := a*b

total := c+(a*b)

"

fit_FusiformGyrus_A_T_full3_TSPO_mediation_std_flipped<-sem(FusiformGyrus_A_T_full3_TSPO_mediation_std_flipped,

dat.wide)

fit_FusiformGyrus_A_T_full3_TSPO_mediation_std_flipped_summary <- summary(fit_FusiformGyrus_A_T_full3_TSPO_mediation_std_flipped,

fit.measures=TRUE,standardized=TRUE,rsquare=TRUE)

fit_FusiformGyrus_A_T_full3_TSPO_mediation_std_flipped_parameters <- parameterEstimates(fit_FusiformGyrus_A_T_full3_TSPO_mediation_std_flipped,

ci=TRUE,level=0.95,boot.ci.type="perc",standardized=TRUE)

G_Cing_A_T_full3_TSPO_mediation_std_flipped =

"

#Regressions

# specify direct effect, path c' (Y~X)

G_Cing_MK6240_PVC_baseline_scale ~ c*G_Cing_ER176_PVC_baseline_scale + TSPO_Affinity_Mixed + TSPO_Affinity_High + Age_baseline_scale + Sex + BMI_baseline_scale + APOE4_Carrier

# path a (M~X)

G_Cing_FBB_baseline_scale ~ a*G_Cing_ER176_PVC_baseline_scale + TSPO_Affinity_Mixed + TSPO_Affinity_High + Age_baseline_scale + Sex + BMI_baseline_scale + APOE4_Carrier

# path b (Y~M)

G_Cing_MK6240_PVC_baseline_scale ~ b*G_Cing_FBB_baseline_scale + APOE4_Carrier

#Defined Parameters:

mediation := a*b

total := c+(a*b)

"

fit_G_Cing_A_T_full3_TSPO_mediation_std_flipped<-sem(G_Cing_A_T_full3_TSPO_mediation_std_flipped,

dat.wide)

fit_G_Cing_A_T_full3_TSPO_mediation_std_flipped_summary <- summary(fit_G_Cing_A_T_full3_TSPO_mediation_std_flipped,

fit.measures=TRUE,standardized=TRUE,rsquare=TRUE)

fit_G_Cing_A_T_full3_TSPO_mediation_std_flipped_parameters <- parameterEstimates(fit_G_Cing_A_T_full3_TSPO_mediation_std_flipped,

ci=TRUE,level=0.95,boot.ci.type="perc",standardized=TRUE)

Hippocampus_A_T_full3_TSPO_mediation_std_flipped =

"

#Regressions

# specify direct effect, path c' (Y~X)

Hippocampus_MK6240_PVC_baseline_scale ~ c*Hippocampus_ER176_PVC_baseline_scale + TSPO_Affinity_Mixed + TSPO_Affinity_High + Age_baseline_scale + Sex + BMI_baseline_scale + APOE4_Carrier

# path a (M~X)

Hippocampus_FBB_baseline_scale ~ a*Hippocampus_ER176_PVC_baseline_scale + TSPO_Affinity_Mixed + TSPO_Affinity_High + Age_baseline_scale + Sex + BMI_baseline_scale + APOE4_Carrier

# path b (Y~M)

Hippocampus_MK6240_PVC_baseline_scale ~ b*Hippocampus_FBB_baseline_scale + APOE4_Carrier

#Defined Parameters:

mediation := a*b

total := c+(a*b)

"

fit_Hippocampus_A_T_full3_TSPO_mediation_std_flipped<-sem(Hippocampus_A_T_full3_TSPO_mediation_std_flipped,

dat.wide)

fit_Hippocampus_A_T_full3_TSPO_mediation_std_flipped_summary <- summary(fit_Hippocampus_A_T_full3_TSPO_mediation_std_flipped,

fit.measures=TRUE,standardized=TRUE,rsquare=TRUE)

fit_Hippocampus_A_T_full3_TSPO_mediation_std_flipped_parameters <- parameterEstimates(fit_Hippocampus_A_T_full3_TSPO_mediation_std_flipped,

ci=TRUE,level=0.95,boot.ci.type="perc",standardized=TRUE)

InferiorParietal_A_T_full3_TSPO_mediation_std_flipped =

"

#Regressions

# specify direct effect, path c' (Y~X)

InferiorParietal_MK6240_PVC_baseline_scale ~ c*InferiorParietal_ER176_PVC_baseline_scale + TSPO_Affinity_Mixed + TSPO_Affinity_High + Age_baseline_scale + Sex + BMI_baseline_scale + APOE4_Carrier

# path a (M~X)

InferiorParietal_FBB_baseline_scale ~ a*InferiorParietal_ER176_PVC_baseline_scale + TSPO_Affinity_Mixed + TSPO_Affinity_High + Age_baseline_scale + Sex + BMI_baseline_scale + APOE4_Carrier

# path b (Y~M)

InferiorParietal_MK6240_PVC_baseline_scale ~ b*InferiorParietal_FBB_baseline_scale + APOE4_Carrier

#Defined Parameters:

mediation := a*b

total := c+(a*b)

"

fit_InferiorParietal_A_T_full3_TSPO_mediation_std_flipped<-sem(InferiorParietal_A_T_full3_TSPO_mediation_std_flipped,

dat.wide)

fit_InferiorParietal_A_T_full3_TSPO_mediation_std_flipped_summary <- summary(fit_InferiorParietal_A_T_full3_TSPO_mediation_std_flipped,

fit.measures=TRUE,standardized=TRUE,rsquare=TRUE)

fit_InferiorParietal_A_T_full3_TSPO_mediation_std_flipped_parameters <- parameterEstimates(fit_InferiorParietal_A_T_full3_TSPO_mediation_std_flipped,

ci=TRUE,level=0.95,boot.ci.type="perc",standardized=TRUE)

Insula_A_T_full3_TSPO_mediation_std_flipped =

"

#Regressions

# specify direct effect, path c' (Y~X)

Insula_MK6240_PVC_baseline_scale ~ c*Insula_ER176_PVC_baseline_scale + TSPO_Affinity_Mixed + TSPO_Affinity_High + Age_baseline_scale + Sex + BMI_baseline_scale + APOE4_Carrier

# path a (M~X)

Insula_FBB_baseline_scale ~ a*Insula_ER176_PVC_baseline_scale + TSPO_Affinity_Mixed + TSPO_Affinity_High + Age_baseline_scale + Sex + BMI_baseline_scale + APOE4_Carrier

# path b (Y~M)

Insula_MK6240_PVC_baseline_scale ~ b*Insula_FBB_baseline_scale + APOE4_Carrier

#Defined Parameters:

mediation := a*b

total := c+(a*b)

"

fit_Insula_A_T_full3_TSPO_mediation_std_flipped<-sem(Insula_A_T_full3_TSPO_mediation_std_flipped,

dat.wide)

fit_Insula_A_T_full3_TSPO_mediation_std_flipped_summary <- summary(fit_Insula_A_T_full3_TSPO_mediation_std_flipped,

fit.measures=TRUE,standardized=TRUE,rsquare=TRUE)

fit_Insula_A_T_full3_TSPO_mediation_std_flipped_parameters <- parameterEstimates(fit_Insula_A_T_full3_TSPO_mediation_std_flipped,

ci=TRUE,level=0.95,boot.ci.type="perc",standardized=TRUE)

LingualGyrus_A_T_full3_TSPO_mediation_std_flipped =

"

#Regressions

# specify direct effect, path c' (Y~X)

LingualGyrus_MK6240_PVC_baseline_scale ~ c*LingualGyrus_ER176_PVC_baseline_scale + TSPO_Affinity_Mixed + TSPO_Affinity_High + Age_baseline_scale + Sex + BMI_baseline_scale + APOE4_Carrier

# path a (M~X)

LingualGyrus_FBB_baseline_scale ~ a*LingualGyrus_ER176_PVC_baseline_scale + TSPO_Affinity_Mixed + TSPO_Affinity_High + Age_baseline_scale + Sex + BMI_baseline_scale + APOE4_Carrier

# path b (Y~M)

LingualGyrus_MK6240_PVC_baseline_scale ~ b*LingualGyrus_FBB_baseline_scale + APOE4_Carrier

#Defined Parameters:

mediation := a*b

total := c+(a*b)

"

fit_LingualGyrus_A_T_full3_TSPO_mediation_std_flipped<-sem(LingualGyrus_A_T_full3_TSPO_mediation_std_flipped,

dat.wide)

fit_LingualGyrus_A_T_full3_TSPO_mediation_std_flipped_summary <- summary(fit_LingualGyrus_A_T_full3_TSPO_mediation_std_flipped,

fit.measures=TRUE,standardized=TRUE,rsquare=TRUE)

fit_LingualGyrus_A_T_full3_TSPO_mediation_std_flipped_parameters <- parameterEstimates(fit_LingualGyrus_A_T_full3_TSPO_mediation_std_flipped,

ci=TRUE,level=0.95,boot.ci.type="perc",standardized=TRUE)

MiddleInfTempGyrus_A_T_full3_TSPO_mediation_std_flipped =

"

#Regressions

# specify direct effect, path c' (Y~X)

MiddleInfTempGyrus_MK6240_PVC_baseline_scale ~ c*MiddleInfTempGyrus_ER176_PVC_baseline_scale + TSPO_Affinity_Mixed + TSPO_Affinity_High + Age_baseline_scale + Sex + BMI_baseline_scale + APOE4_Carrier

# path a (M~X)

MiddleInfTempGyrus_FBB_baseline_scale ~ a*MiddleInfTempGyrus_ER176_PVC_baseline_scale + TSPO_Affinity_Mixed + TSPO_Affinity_High + Age_baseline_scale + Sex + BMI_baseline_scale + APOE4_Carrier

# path b (Y~M)

MiddleInfTempGyrus_MK6240_PVC_baseline_scale ~ b*MiddleInfTempGyrus_FBB_baseline_scale + APOE4_Carrier

#Defined Parameters:

mediation := a*b

total := c+(a*b)

"

fit_MiddleInfTempGyrus_A_T_full3_TSPO_mediation_std_flipped<-sem(MiddleInfTempGyrus_A_T_full3_TSPO_mediation_std_flipped,

dat.wide)

fit_MiddleInfTempGyrus_A_T_full3_TSPO_mediation_std_flipped_summary <- summary(fit_MiddleInfTempGyrus_A_T_full3_TSPO_mediation_std_flipped,

fit.measures=TRUE,standardized=TRUE,rsquare=TRUE)

fit_MiddleInfTempGyrus_A_T_full3_TSPO_mediation_std_flipped_parameters <- parameterEstimates(fit_MiddleInfTempGyrus_A_T_full3_TSPO_mediation_std_flipped,

ci=TRUE,level=0.95,boot.ci.type="perc",standardized=TRUE)

Prefrontal_A_T_full3_TSPO_mediation_std_flipped =

"

#Regressions

# specify direct effect, path c' (Y~X)

Prefrontal_MK6240_PVC_baseline_scale ~ c*Prefrontal_ER176_PVC_baseline_scale + TSPO_Affinity_Mixed + TSPO_Affinity_High + Age_baseline_scale + Sex + BMI_baseline_scale + APOE4_Carrier

# path a (M~X)

Prefrontal_FBB_baseline_scale ~ a*Prefrontal_ER176_PVC_baseline_scale + TSPO_Affinity_Mixed + TSPO_Affinity_High + Age_baseline_scale + Sex + BMI_baseline_scale + APOE4_Carrier

# path b (Y~M)

Prefrontal_MK6240_PVC_baseline_scale ~ b*Prefrontal_FBB_baseline_scale + APOE4_Carrier

#Defined Parameters:

mediation := a*b

total := c+(a*b)

"

fit_Prefrontal_A_T_full3_TSPO_mediation_std_flipped<-sem(Prefrontal_A_T_full3_TSPO_mediation_std_flipped,

dat.wide)

fit_Prefrontal_A_T_full3_TSPO_mediation_std_flipped_summary <- summary(fit_Prefrontal_A_T_full3_TSPO_mediation_std_flipped,

fit.measures=TRUE,standardized=TRUE,rsquare=TRUE)

fit_Prefrontal_A_T_full3_TSPO_mediation_std_flipped_parameters <- parameterEstimates(fit_Prefrontal_A_T_full3_TSPO_mediation_std_flipped,

ci=TRUE,level=0.95,boot.ci.type="perc",standardized=TRUE)

SuperiorParietal_A_T_full3_TSPO_mediation_std_flipped =

"

#Regressions

# specify direct effect, path c' (Y~X)

SuperiorParietal_MK6240_PVC_baseline_scale ~ c*SuperiorParietal_ER176_PVC_baseline_scale + TSPO_Affinity_Mixed + TSPO_Affinity_High + Age_baseline_scale + Sex + BMI_baseline_scale + APOE4_Carrier

# path a (M~X)

SuperiorParietal_FBB_baseline_scale ~ a*SuperiorParietal_ER176_PVC_baseline_scale + TSPO_Affinity_Mixed + TSPO_Affinity_High + Age_baseline_scale + Sex + BMI_baseline_scale + APOE4_Carrier

# path b (Y~M)

SuperiorParietal_MK6240_PVC_baseline_scale ~ b*SuperiorParietal_FBB_baseline_scale + APOE4_Carrier

#Defined Parameters:

mediation := a*b

total := c+(a*b)

"

fit_SuperiorParietal_A_T_full3_TSPO_mediation_std_flipped<-sem(SuperiorParietal_A_T_full3_TSPO_mediation_std_flipped,

dat.wide)

fit_SuperiorParietal_A_T_full3_TSPO_mediation_std_flipped_summary <- summary(fit_SuperiorParietal_A_T_full3_TSPO_mediation_std_flipped,

fit.measures=TRUE,standardized=TRUE,rsquare=TRUE)

fit_SuperiorParietal_A_T_full3_TSPO_mediation_std_flipped_parameters <- parameterEstimates(fit_SuperiorParietal_A_T_full3_TSPO_mediation_std_flipped,

ci=TRUE,level=0.95,boot.ci.type="perc",standardized=TRUE)

SuperiorTemp_A_T_full3_TSPO_mediation_std_flipped =

"

#Regressions

# specify direct effect, path c' (Y~X)

SuperiorTemp_MK6240_PVC_baseline_scale ~ c*SuperiorTemp_ER176_PVC_baseline_scale + TSPO_Affinity_Mixed + TSPO_Affinity_High + Age_baseline_scale + Sex + BMI_baseline_scale + APOE4_Carrier

# path a (M~X)

SuperiorTemp_FBB_baseline_scale ~ a*SuperiorTemp_ER176_PVC_baseline_scale + TSPO_Affinity_Mixed + TSPO_Affinity_High + Age_baseline_scale + Sex + BMI_baseline_scale + APOE4_Carrier

# path b (Y~M)

SuperiorTemp_MK6240_PVC_baseline_scale ~ b*SuperiorTemp_FBB_baseline_scale + APOE4_Carrier

#Defined Parameters:

mediation := a*b

total := c+(a*b)

"

fit_SuperiorTemp_A_T_full3_TSPO_mediation_std_flipped<-sem(SuperiorTemp_A_T_full3_TSPO_mediation_std_flipped,

dat.wide)

fit_SuperiorTemp_A_T_full3_TSPO_mediation_std_flipped_summary <- summary(fit_SuperiorTemp_A_T_full3_TSPO_mediation_std_flipped,

fit.measures=TRUE,standardized=TRUE,rsquare=TRUE)

fit_SuperiorTemp_A_T_full3_TSPO_mediation_std_flipped_parameters <- parameterEstimates(fit_SuperiorTemp_A_T_full3_TSPO_mediation_std_flipped,

ci=TRUE,level=0.95,boot.ci.type="perc",standardized=TRUE)

## adjust pvalues

AT_rois <- c("Prefrontal","Insula","G_Cing","FusiformGyrus","LingualGyrus",

"EntorhinalCortex_FS","MiddleInfTempGyrus","SuperiorTemp",

"InferiorParietal","SuperiorParietal",

"Amygdala","Hippocampus","Striatum")

# Amyloid to TPSO

AT_TSPO_mediation_flipped_A_p <- c(fit_Prefrontal_A_T_full3_TSPO_mediation_flipped_parameters[fit_Prefrontal_A_T_full3_TSPO_mediation_flipped_parameters$label=="a","pvalue"],

fit_Insula_A_T_full3_TSPO_mediation_flipped_parameters[fit_Insula_A_T_full3_TSPO_mediation_flipped_parameters$label=="a","pvalue"],

fit_G_Cing_A_T_full3_TSPO_mediation_flipped_parameters[fit_G_Cing_A_T_full3_TSPO_mediation_flipped_parameters$label=="a","pvalue"],

fit_FusiformGyrus_A_T_full3_TSPO_mediation_flipped_parameters[fit_FusiformGyrus_A_T_full3_TSPO_mediation_flipped_parameters$label=="a","pvalue"],

fit_LingualGyrus_A_T_full3_TSPO_mediation_flipped_parameters[fit_LingualGyrus_A_T_full3_TSPO_mediation_flipped_parameters$label=="a","pvalue"],

fit_EntorhinalCortex_FS_A_T_full3_TSPO_mediation_flipped_parameters[fit_EntorhinalCortex_FS_A_T_full3_TSPO_mediation_flipped_parameters$label=="a","pvalue"],

fit_MiddleInfTempGyrus_A_T_full3_TSPO_mediation_flipped_parameters[fit_MiddleInfTempGyrus_A_T_full3_TSPO_mediation_flipped_parameters$label=="a","pvalue"],

fit_SuperiorTemp_A_T_full3_TSPO_mediation_flipped_parameters[fit_SuperiorTemp_A_T_full3_TSPO_mediation_flipped_parameters$label=="a","pvalue"],

fit_InferiorParietal_A_T_full3_TSPO_mediation_flipped_parameters[fit_InferiorParietal_A_T_full3_TSPO_mediation_flipped_parameters$label=="a","pvalue"],

fit_SuperiorParietal_A_T_full3_TSPO_mediation_flipped_parameters[fit_SuperiorParietal_A_T_full3_TSPO_mediation_flipped_parameters$label=="a","pvalue"],

fit_Amygdala_A_T_full3_TSPO_mediation_flipped_parameters[fit_Amygdala_A_T_full3_TSPO_mediation_flipped_parameters$label=="a","pvalue"],

fit_Hippocampus_A_T_full3_TSPO_mediation_flipped_parameters[fit_Hippocampus_A_T_full3_TSPO_mediation_flipped_parameters$label=="a","pvalue"],

fit_Striatum_A_T_full3_TSPO_mediation_flipped_parameters[fit_Striatum_A_T_full3_TSPO_mediation_flipped_parameters$label=="a","pvalue"])

AT_TSPO_mediation_flipped_A_p_adjusted <- p.adjust(AT_TSPO_mediation_flipped_A_p, method = "BH", n = length(AT_TSPO_mediation_flipped_A_p))

AT_TSPO_mediation_flipped_A_p_adjusted_roi <- cbind(AT_rois,AT_TSPO_mediation_flipped_A_p_adjusted)

AT_TSPO_mediation_flipped_A_p_adjusted_df <- as.data.frame(AT_TSPO_mediation_flipped_A_p_adjusted_roi)

# TSPO to Tau

AT_TSPO_mediation_flipped_B_p <- c(fit_Prefrontal_A_T_full3_TSPO_mediation_flipped_parameters[fit_Prefrontal_A_T_full3_TSPO_mediation_flipped_parameters$label=="b","pvalue"],

fit_Insula_A_T_full3_TSPO_mediation_flipped_parameters[fit_Insula_A_T_full3_TSPO_mediation_flipped_parameters$label=="b","pvalue"],

fit_G_Cing_A_T_full3_TSPO_mediation_flipped_parameters[fit_G_Cing_A_T_full3_TSPO_mediation_flipped_parameters$label=="b","pvalue"],

fit_FusiformGyrus_A_T_full3_TSPO_mediation_flipped_parameters[fit_FusiformGyrus_A_T_full3_TSPO_mediation_flipped_parameters$label=="b","pvalue"],

fit_LingualGyrus_A_T_full3_TSPO_mediation_flipped_parameters[fit_LingualGyrus_A_T_full3_TSPO_mediation_flipped_parameters$label=="b","pvalue"],

fit_EntorhinalCortex_FS_A_T_full3_TSPO_mediation_flipped_parameters[fit_EntorhinalCortex_FS_A_T_full3_TSPO_mediation_flipped_parameters$label=="b","pvalue"],

fit_MiddleInfTempGyrus_A_T_full3_TSPO_mediation_flipped_parameters[fit_MiddleInfTempGyrus_A_T_full3_TSPO_mediation_flipped_parameters$label=="b","pvalue"],

fit_SuperiorTemp_A_T_full3_TSPO_mediation_flipped_parameters[fit_SuperiorTemp_A_T_full3_TSPO_mediation_flipped_parameters$label=="b","pvalue"],

fit_InferiorParietal_A_T_full3_TSPO_mediation_flipped_parameters[fit_InferiorParietal_A_T_full3_TSPO_mediation_flipped_parameters$label=="b","pvalue"],

fit_SuperiorParietal_A_T_full3_TSPO_mediation_flipped_parameters[fit_SuperiorParietal_A_T_full3_TSPO_mediation_flipped_parameters$label=="b","pvalue"],

fit_Amygdala_A_T_full3_TSPO_mediation_flipped_parameters[fit_Amygdala_A_T_full3_TSPO_mediation_flipped_parameters$label=="b","pvalue"],

fit_Hippocampus_A_T_full3_TSPO_mediation_flipped_parameters[fit_Hippocampus_A_T_full3_TSPO_mediation_flipped_parameters$label=="b","pvalue"],

fit_Striatum_A_T_full3_TSPO_mediation_flipped_parameters[fit_Striatum_A_T_full3_TSPO_mediation_flipped_parameters$label=="b","pvalue"])

AT_TSPO_mediation_flipped_B_p_adjusted <- p.adjust(AT_TSPO_mediation_flipped_B_p, method = "BH", n = length(AT_TSPO_mediation_flipped_B_p))

AT_TSPO_mediation_flipped_B_p_adjusted_roi <- cbind(AT_rois,AT_TSPO_mediation_flipped_B_p_adjusted)

AT_TSPO_mediation_flipped_B_p_adjusted_df <- as.data.frame(AT_TSPO_mediation_flipped_B_p_adjusted_roi)

# Amyloid to Tau

AT_TSPO_mediation_flipped_C_p <- c(fit_Prefrontal_A_T_full3_TSPO_mediation_flipped_parameters[fit_Prefrontal_A_T_full3_TSPO_mediation_flipped_parameters$label=="c","pvalue"],

fit_Insula_A_T_full3_TSPO_mediation_flipped_parameters[fit_Insula_A_T_full3_TSPO_mediation_flipped_parameters$label=="c","pvalue"],

fit_G_Cing_A_T_full3_TSPO_mediation_flipped_parameters[fit_G_Cing_A_T_full3_TSPO_mediation_flipped_parameters$label=="c","pvalue"],

fit_FusiformGyrus_A_T_full3_TSPO_mediation_flipped_parameters[fit_FusiformGyrus_A_T_full3_TSPO_mediation_flipped_parameters$label=="c","pvalue"],

fit_LingualGyrus_A_T_full3_TSPO_mediation_flipped_parameters[fit_LingualGyrus_A_T_full3_TSPO_mediation_flipped_parameters$label=="c","pvalue"],

fit_EntorhinalCortex_FS_A_T_full3_TSPO_mediation_flipped_parameters[fit_EntorhinalCortex_FS_A_T_full3_TSPO_mediation_flipped_parameters$label=="c","pvalue"],

fit_MiddleInfTempGyrus_A_T_full3_TSPO_mediation_flipped_parameters[fit_MiddleInfTempGyrus_A_T_full3_TSPO_mediation_flipped_parameters$label=="c","pvalue"],

fit_SuperiorTemp_A_T_full3_TSPO_mediation_flipped_parameters[fit_SuperiorTemp_A_T_full3_TSPO_mediation_flipped_parameters$label=="c","pvalue"],

fit_InferiorParietal_A_T_full3_TSPO_mediation_flipped_parameters[fit_InferiorParietal_A_T_full3_TSPO_mediation_flipped_parameters$label=="c","pvalue"],

fit_SuperiorParietal_A_T_full3_TSPO_mediation_flipped_parameters[fit_SuperiorParietal_A_T_full3_TSPO_mediation_flipped_parameters$label=="c","pvalue"],

fit_Amygdala_A_T_full3_TSPO_mediation_flipped_parameters[fit_Amygdala_A_T_full3_TSPO_mediation_flipped_parameters$label=="c","pvalue"],

fit_Hippocampus_A_T_full3_TSPO_mediation_flipped_parameters[fit_Hippocampus_A_T_full3_TSPO_mediation_flipped_parameters$label=="c","pvalue"],

fit_Striatum_A_T_full3_TSPO_mediation_flipped_parameters[fit_Striatum_A_T_full3_TSPO_mediation_flipped_parameters$label=="c","pvalue"])

AT_TSPO_mediation_flipped_C_p_adjusted <- p.adjust(AT_TSPO_mediation_flipped_C_p, method = "BH", n = length(AT_TSPO_mediation_flipped_C_p))

AT_TSPO_mediation_flipped_C_p_adjusted_roi <- cbind(AT_rois,AT_TSPO_mediation_flipped_C_p_adjusted)

AT_TSPO_mediation_flipped_C_p_adjusted_df <- as.data.frame(AT_TSPO_mediation_flipped_C_p_adjusted_roi)

# Amyloid to TSPO to Tau

AT_TSPO_mediation_flipped_Cprime_p <- c(fit_Prefrontal_A_T_full3_TSPO_mediation_flipped_parameters[fit_Prefrontal_A_T_full3_TSPO_mediation_flipped_parameters$label=="mediation","pvalue"],

fit_Insula_A_T_full3_TSPO_mediation_flipped_parameters[fit_Insula_A_T_full3_TSPO_mediation_flipped_parameters$label=="mediation","pvalue"],

fit_G_Cing_A_T_full3_TSPO_mediation_flipped_parameters[fit_G_Cing_A_T_full3_TSPO_mediation_flipped_parameters$label=="mediation","pvalue"],

fit_FusiformGyrus_A_T_full3_TSPO_mediation_flipped_parameters[fit_FusiformGyrus_A_T_full3_TSPO_mediation_flipped_parameters$label=="mediation","pvalue"],

fit_LingualGyrus_A_T_full3_TSPO_mediation_flipped_parameters[fit_LingualGyrus_A_T_full3_TSPO_mediation_flipped_parameters$label=="mediation","pvalue"],

fit_EntorhinalCortex_FS_A_T_full3_TSPO_mediation_flipped_parameters[fit_EntorhinalCortex_FS_A_T_full3_TSPO_mediation_flipped_parameters$label=="mediation","pvalue"],

fit_MiddleInfTempGyrus_A_T_full3_TSPO_mediation_flipped_parameters[fit_MiddleInfTempGyrus_A_T_full3_TSPO_mediation_flipped_parameters$label=="mediation","pvalue"],

fit_SuperiorTemp_A_T_full3_TSPO_mediation_flipped_parameters[fit_SuperiorTemp_A_T_full3_TSPO_mediation_flipped_parameters$label=="mediation","pvalue"],

fit_InferiorParietal_A_T_full3_TSPO_mediation_flipped_parameters[fit_InferiorParietal_A_T_full3_TSPO_mediation_flipped_parameters$label=="mediation","pvalue"],

fit_SuperiorParietal_A_T_full3_TSPO_mediation_flipped_parameters[fit_SuperiorParietal_A_T_full3_TSPO_mediation_flipped_parameters$label=="mediation","pvalue"],

fit_Amygdala_A_T_full3_TSPO_mediation_flipped_parameters[fit_Amygdala_A_T_full3_TSPO_mediation_flipped_parameters$label=="mediation","pvalue"],

fit_Hippocampus_A_T_full3_TSPO_mediation_flipped_parameters[fit_Hippocampus_A_T_full3_TSPO_mediation_flipped_parameters$label=="mediation","pvalue"],

fit_Striatum_A_T_full3_TSPO_mediation_flipped_parameters[fit_Striatum_A_T_full3_TSPO_mediation_flipped_parameters$label=="mediation","pvalue"])

AT_TSPO_mediation_flipped_Cprime_p_adjusted <- p.adjust(AT_TSPO_mediation_flipped_Cprime_p, method = "BH", n = length(AT_TSPO_mediation_flipped_Cprime_p))

AT_TSPO_mediation_flipped_Cprime_p_adjusted_roi <- cbind(AT_rois,AT_TSPO_mediation_flipped_Cprime_p_adjusted)

AT_TSPO_mediation_flipped_Cprime_p_adjusted_df <- as.data.frame(AT_TSPO_mediation_flipped_Cprime_p_adjusted_roi)

# Total

AT_TSPO_mediation_flipped_Total_p <- c(fit_Prefrontal_A_T_full3_TSPO_mediation_flipped_parameters[fit_Prefrontal_A_T_full3_TSPO_mediation_flipped_parameters$label=="total","pvalue"],

fit_Insula_A_T_full3_TSPO_mediation_flipped_parameters[fit_Insula_A_T_full3_TSPO_mediation_flipped_parameters$label=="total","pvalue"],

fit_G_Cing_A_T_full3_TSPO_mediation_flipped_parameters[fit_G_Cing_A_T_full3_TSPO_mediation_flipped_parameters$label=="total","pvalue"],

fit_FusiformGyrus_A_T_full3_TSPO_mediation_flipped_parameters[fit_FusiformGyrus_A_T_full3_TSPO_mediation_flipped_parameters$label=="total","pvalue"],

fit_LingualGyrus_A_T_full3_TSPO_mediation_flipped_parameters[fit_LingualGyrus_A_T_full3_TSPO_mediation_flipped_parameters$label=="total","pvalue"],

fit_EntorhinalCortex_FS_A_T_full3_TSPO_mediation_flipped_parameters[fit_EntorhinalCortex_FS_A_T_full3_TSPO_mediation_flipped_parameters$label=="total","pvalue"],

fit_MiddleInfTempGyrus_A_T_full3_TSPO_mediation_flipped_parameters[fit_MiddleInfTempGyrus_A_T_full3_TSPO_mediation_flipped_parameters$label=="total","pvalue"],

fit_SuperiorTemp_A_T_full3_TSPO_mediation_flipped_parameters[fit_SuperiorTemp_A_T_full3_TSPO_mediation_flipped_parameters$label=="total","pvalue"],

fit_InferiorParietal_A_T_full3_TSPO_mediation_flipped_parameters[fit_InferiorParietal_A_T_full3_TSPO_mediation_flipped_parameters$label=="total","pvalue"],

fit_SuperiorParietal_A_T_full3_TSPO_mediation_flipped_parameters[fit_SuperiorParietal_A_T_full3_TSPO_mediation_flipped_parameters$label=="total","pvalue"],

fit_Amygdala_A_T_full3_TSPO_mediation_flipped_parameters[fit_Amygdala_A_T_full3_TSPO_mediation_flipped_parameters$label=="total","pvalue"],

fit_Hippocampus_A_T_full3_TSPO_mediation_flipped_parameters[fit_Hippocampus_A_T_full3_TSPO_mediation_flipped_parameters$label=="total","pvalue"],

fit_Striatum_A_T_full3_TSPO_mediation_flipped_parameters[fit_Striatum_A_T_full3_TSPO_mediation_flipped_parameters$label=="total","pvalue"])

AT_TSPO_mediation_flipped_Total_p_adjusted <- p.adjust(AT_TSPO_mediation_flipped_Total_p, method = "BH", n = length(AT_TSPO_mediation_flipped_Total_p))

AT_TSPO_mediation_flipped_Total_p_adjusted_roi <- cbind(AT_rois,AT_TSPO_mediation_flipped_Total_p_adjusted)

AT_TSPO_mediation_flipped_Total_p_adjusted_df <- as.data.frame(AT_TSPO_mediation_flipped_Total_p_adjusted_roi)

# Tau Spreading std

early_to_middle_Braak_full3_TSPO_mediation_std_flipped =

"

#Regressions

# specify direct effect, path c'

Braak_III_IV_MK6240_PVC_baseline_scale ~ c*Composite_ER176_PVC_baseline_scale + TSPO_Affinity_Mixed + TSPO_Affinity_High + Age_baseline_scale + Sex + BMI_baseline_scale + APOE4_Carrier

# path a

Braak_I_II_MK6240_PVC_baseline_scale ~ a*Composite_ER176_PVC_baseline_scale + TSPO_Affinity_Mixed + TSPO_Affinity_High + Age_baseline_scale + Sex + BMI_baseline_scale + APOE4_Carrier

# path b

Braak_III_IV_MK6240_PVC_baseline_scale ~ b*Braak_I_II_MK6240_PVC_baseline_scale + APOE4_Carrier

#Defined Parameters:

mediation := a*b

total := c+(a*b)

"

fit_early_to_middle_Braak_full3_TSPO_mediation_std_flipped<-sem(early_to_middle_Braak_full3_TSPO_mediation_std_flipped,

dat.wide)

fit_early_to_middle_Braak_full3_TSPO_mediation_std_flipped_summary <- summary(fit_early_to_middle_Braak_full3_TSPO_mediation_std_flipped,

fit.measures=TRUE,standardized=TRUE,rsquare=TRUE)

fit_early_to_middle_Braak_full3_TSPO_mediation_std_flipped_parameters <- parameterEstimates(fit_early_to_middle_Braak_full3_TSPO_mediation_std_flipped,

ci=TRUE,level=0.95,boot.ci.type="perc", standardized=TRUE)

middle_to_late_Braak_full3_TSPO_mediation_std_flipped =

"

#Regressions

# specify direct effect, path c'

Braak_V_VI_MK6240_PVC_baseline_scale ~ c*Composite_ER176_PVC_baseline_scale + TSPO_Affinity_Mixed + TSPO_Affinity_High + Age_baseline_scale + Sex + BMI_baseline_scale + APOE4_Carrier

# path a

Braak_III_IV_MK6240_PVC_baseline_scale ~ a*Composite_ER176_PVC_baseline_scale + TSPO_Affinity_Mixed + TSPO_Affinity_High + Age_baseline_scale + Sex + BMI_baseline_scale + APOE4_Carrier

# path b

Braak_V_VI_MK6240_PVC_baseline_scale ~ b*Braak_III_IV_MK6240_PVC_baseline_scale + APOE4_Carrier

#Defined Parameters:

mediation := a*b

total := c+(a*b)

"

fit_middle_to_late_Braak_full3_TSPO_mediation_std_flipped<-sem(middle_to_late_Braak_full3_TSPO_mediation_std_flipped,

dat.wide)

fit_middle_to_late_Braak_full3_TSPO_mediation_std_flipped_summary <- summary(fit_middle_to_late_Braak_full3_TSPO_mediation_std_flipped,

fit.measures=TRUE,standardized=TRUE,rsquare=TRUE)

fit_middle_to_late_Braak_full3_TSPO_mediation_std_flipped_parameters <- parameterEstimates(fit_middle_to_late_Braak_full3_TSPO_mediation_std_flipped,

ci=TRUE,level=0.95,boot.ci.type="perc", standardized=TRUE)

## adjust pvalues

TT_rois <- c("early_to_middle","middle_to_late")

# Tau to TSPO

TT_TSPO_mediation_flipped_A_p <- c(fit_early_to_middle_Braak_full3_TSPO_mediation_flipped_parameters[fit_early_to_middle_Braak_full3_TSPO_mediation_flipped_parameters$label=="a","pvalue"],

fit_middle_to_late_Braak_full3_TSPO_mediation_flipped_parameters[fit_middle_to_late_Braak_full3_TSPO_mediation_flipped_parameters$label=="a","pvalue"])

TT_TSPO_mediation_flipped_A_p_adjusted <- p.adjust(TT_TSPO_mediation_flipped_A_p, method = "BH", n = length(TT_TSPO_mediation_flipped_A_p))

TT_TSPO_mediation_flipped_A_p_adjusted_roi <- cbind(TT_rois,TT_TSPO_mediation_flipped_A_p_adjusted)

TT_TSPO_mediation_flipped_A_p_adjusted_df <- as.data.frame(TT_TSPO_mediation_flipped_A_p_adjusted_roi)

# TSPO to Tau

TT_TSPO_mediation_flipped_B_p <- c(fit_early_to_middle_Braak_full3_TSPO_mediation_flipped_parameters[fit_early_to_middle_Braak_full3_TSPO_mediation_flipped_parameters$label=="b","pvalue"],

fit_middle_to_late_Braak_full3_TSPO_mediation_flipped_parameters[fit_middle_to_late_Braak_full3_TSPO_mediation_flipped_parameters$label=="b","pvalue"])

TT_TSPO_mediation_flipped_B_p_adjusted <- p.adjust(TT_TSPO_mediation_flipped_B_p, method = "BH", n = length(TT_TSPO_mediation_flipped_B_p))

TT_TSPO_mediation_flipped_B_p_adjusted_roi <- cbind(TT_rois,TT_TSPO_mediation_flipped_B_p_adjusted)

TT_TSPO_mediation_flipped_B_p_adjusted_df <- as.data.frame(TT_TSPO_mediation_flipped_B_p_adjusted_roi)

# Tau to Tau

TT_TSPO_mediation_flipped_C_p <- c(fit_early_to_middle_Braak_full3_TSPO_mediation_flipped_parameters[fit_early_to_middle_Braak_full3_TSPO_mediation_flipped_parameters$label=="c","pvalue"],

fit_middle_to_late_Braak_full3_TSPO_mediation_flipped_parameters[fit_middle_to_late_Braak_full3_TSPO_mediation_flipped_parameters$label=="c","pvalue"])

TT_TSPO_mediation_flipped_C_p_adjusted <- p.adjust(TT_TSPO_mediation_flipped_C_p, method = "BH", n = length(TT_TSPO_mediation_flipped_C_p))

TT_TSPO_mediation_flipped_C_p_adjusted_roi <- cbind(TT_rois,TT_TSPO_mediation_flipped_C_p_adjusted)

TT_TSPO_mediation_flipped_C_p_adjusted_df <- as.data.frame(TT_TSPO_mediation_flipped_C_p_adjusted_roi)

# Tau to TSPO to Tau

TT_TSPO_mediation_flipped_Cprime_p <- c(fit_early_to_middle_Braak_full3_TSPO_mediation_flipped_parameters[fit_early_to_middle_Braak_full3_TSPO_mediation_flipped_parameters$label=="mediation","pvalue"],

fit_middle_to_late_Braak_full3_TSPO_mediation_flipped_parameters[fit_middle_to_late_Braak_full3_TSPO_mediation_flipped_parameters$label=="mediation","pvalue"])

TT_TSPO_mediation_flipped_Cprime_p_adjusted <- p.adjust(TT_TSPO_mediation_flipped_Cprime_p, method = "BH", n = length(TT_TSPO_mediation_flipped_Cprime_p))

TT_TSPO_mediation_flipped_Cprime_p_adjusted_roi <- cbind(TT_rois,TT_TSPO_mediation_flipped_Cprime_p_adjusted)

TT_TSPO_mediation_flipped_Cprime_p_adjusted_df <- as.data.frame(TT_TSPO_mediation_flipped_Cprime_p_adjusted_roi)

# Total

TT_TSPO_mediation_flipped_Total_p <- c(fit_early_to_middle_Braak_full3_TSPO_mediation_flipped_parameters[fit_early_to_middle_Braak_full3_TSPO_mediation_flipped_parameters$label=="total","pvalue"],

fit_middle_to_late_Braak_full3_TSPO_mediation_flipped_parameters[fit_middle_to_late_Braak_full3_TSPO_mediation_flipped_parameters$label=="total","pvalue"])

TT_TSPO_mediation_flipped_Total_p_adjusted <- p.adjust(TT_TSPO_mediation_flipped_Total_p, method = "BH", n = length(TT_TSPO_mediation_flipped_Total_p))

TT_TSPO_mediation_flipped_Total_p_adjusted_roi <- cbind(TT_rois,TT_TSPO_mediation_flipped_Total_p_adjusted)

TT_TSPO_mediation_flipped_Total_p_adjusted_df <- as.data.frame(TT_TSPO_mediation_flipped_Total_p_adjusted_roi)

# Regional T -> N std

Striatum_T_N_full3_TSPO_mediation_std_flipped =

"

#Regressions

# specify direct effect, path c'

Striatum_Volume_MK6240_baseline_scale ~ c*Striatum_ER176_PVC_baseline_scale + TSPO_Affinity_Mixed + TSPO_Affinity_High + ICV_baseline_scale + Age_baseline_scale + Sex + BMI_baseline_scale + APOE4_Carrier

# path a

Striatum_MK6240_PVC_baseline_scale ~ a*Striatum_ER176_PVC_baseline_scale + TSPO_Affinity_Mixed + TSPO_Affinity_High + Age_baseline_scale + Sex + BMI_baseline_scale + APOE4_Carrier

# path b

Striatum_Volume_MK6240_baseline_scale ~ b*Striatum_MK6240_PVC_baseline_scale + ICV_baseline_scale + APOE4_Carrier

#Defined Parameters:

mediation := a*b

total := c+(a*b)

"

fit_Striatum_T_N_full3_TSPO_mediation_std_flipped<-sem(Striatum_T_N_full3_TSPO_mediation_std_flipped,

dat.wide)

fit_Striatum_T_N_full3_TSPO_mediation_std_flipped_summary <- summary(fit_Striatum_T_N_full3_TSPO_mediation_std_flipped,

fit.measures=TRUE,standardized=TRUE,rsquare=TRUE)

fit_Striatum_T_N_full3_TSPO_mediation_std_flipped_parameters <- parameterEstimates(fit_Striatum_T_N_full3_TSPO_mediation_std_flipped,

ci=TRUE,level=0.95,boot.ci.type="perc",standardized=TRUE)

Amygdala_T_N_full3_TSPO_mediation_std_flipped =

"

#Regressions

# specify direct effect, path c'

Amygdala_Volume_MK6240_baseline_scale ~ c*Amygdala_ER176_PVC_baseline_scale + TSPO_Affinity_Mixed + TSPO_Affinity_High + ICV_baseline_scale + Age_baseline_scale + Sex + BMI_baseline_scale + APOE4_Carrier

# path a

Amygdala_MK6240_PVC_baseline_scale ~ a*Amygdala_ER176_PVC_baseline_scale + TSPO_Affinity_Mixed + TSPO_Affinity_High + Age_baseline_scale + Sex + BMI_baseline_scale + APOE4_Carrier

# path b

Amygdala_Volume_MK6240_baseline_scale ~ b*Amygdala_MK6240_PVC_baseline_scale + ICV_baseline_scale + APOE4_Carrier

#Defined Parameters:

mediation := a*b

total := c+(a*b)

"

fit_Amygdala_T_N_full3_TSPO_mediation_std_flipped<-sem(Amygdala_T_N_full3_TSPO_mediation_std_flipped,

dat.wide)

fit_Amygdala_T_N_full3_TSPO_mediation_std_flipped_summary <- summary(fit_Amygdala_T_N_full3_TSPO_mediation_std_flipped,

fit.measures=TRUE,standardized=TRUE,rsquare=TRUE)

fit_Amygdala_T_N_full3_TSPO_mediation_std_flipped_parameters <- parameterEstimates(fit_Amygdala_T_N_full3_TSPO_mediation_std_flipped,

ci=TRUE,level=0.95,boot.ci.type="perc",standardized=TRUE)

EntorhinalCortex_FS_T_N_full3_TSPO_mediation_std_flipped =

"

#Regressions

# specify direct effect, path c'

EntorhinalCortex_FS_Volume_MK6240_baseline_scale ~ c*EntorhinalCortex_FS_ER176_PVC_baseline_scale + TSPO_Affinity_Mixed + TSPO_Affinity_High + ICV_baseline_scale + Age_baseline_scale + Sex + BMI_baseline_scale + APOE4_Carrier

# path a

EntorhinalCortex_FS_MK6240_PVC_baseline_scale ~ a*EntorhinalCortex_FS_ER176_PVC_baseline_scale + TSPO_Affinity_Mixed + TSPO_Affinity_High + Age_baseline_scale + Sex + BMI_baseline_scale + APOE4_Carrier

# path b

EntorhinalCortex_FS_Volume_MK6240_baseline_scale ~ b*EntorhinalCortex_FS_MK6240_PVC_baseline_scale + ICV_baseline_scale + APOE4_Carrier

#Defined Parameters:

mediation := a*b

total := c+(a*b)

"

fit_EntorhinalCortex_FS_T_N_full3_TSPO_mediation_std_flipped<-sem(EntorhinalCortex_FS_T_N_full3_TSPO_mediation_std_flipped,

dat.wide)

fit_EntorhinalCortex_FS_T_N_full3_TSPO_mediation_std_flipped_summary <- summary(fit_EntorhinalCortex_FS_T_N_full3_TSPO_mediation_std_flipped,

fit.measures=TRUE,standardized=TRUE,rsquare=TRUE)

fit_EntorhinalCortex_FS_T_N_full3_TSPO_mediation_std_flipped_parameters <- parameterEstimates(fit_EntorhinalCortex_FS_T_N_full3_TSPO_mediation_std_flipped,

ci=TRUE,level=0.95,boot.ci.type="perc",standardized=TRUE)

FusiformGyrus_T_N_full3_TSPO_mediation_std_flipped =

"

#Regressions

# specify direct effect, path c'

FusiformGyrus_Volume_MK6240_baseline_scale ~ c*FusiformGyrus_ER176_PVC_baseline_scale + TSPO_Affinity_Mixed + TSPO_Affinity_High + ICV_baseline_scale + Age_baseline_scale + Sex + BMI_baseline_scale + APOE4_Carrier

# path a

FusiformGyrus_MK6240_PVC_baseline_scale ~ a*FusiformGyrus_ER176_PVC_baseline_scale + TSPO_Affinity_Mixed + TSPO_Affinity_High + Age_baseline_scale + Sex + BMI_baseline_scale + APOE4_Carrier

# path b

FusiformGyrus_Volume_MK6240_baseline_scale ~ b*FusiformGyrus_MK6240_PVC_baseline_scale + ICV_baseline_scale + APOE4_Carrier

#Defined Parameters:

mediation := a*b

total := c+(a*b)

"

fit_FusiformGyrus_T_N_full3_TSPO_mediation_std_flipped<-sem(FusiformGyrus_T_N_full3_TSPO_mediation_std_flipped,

dat.wide)

fit_FusiformGyrus_T_N_full3_TSPO_mediation_std_flipped_summary <- summary(fit_FusiformGyrus_T_N_full3_TSPO_mediation_std_flipped,

fit.measures=TRUE,standardized=TRUE,rsquare=TRUE)

fit_FusiformGyrus_T_N_full3_TSPO_mediation_std_flipped_parameters <- parameterEstimates(fit_FusiformGyrus_T_N_full3_TSPO_mediation_std_flipped,

ci=TRUE,level=0.95,boot.ci.type="perc",standardized=TRUE)

G_Cing_T_N_full3_TSPO_mediation_std_flipped =

"

#Regressions

# specify direct effect, path c'

G_Cing_Volume_MK6240_baseline_scale ~ c*G_Cing_ER176_PVC_baseline_scale + TSPO_Affinity_Mixed + TSPO_Affinity_High + ICV_baseline_scale + Age_baseline_scale + Sex + BMI_baseline_scale + APOE4_Carrier

# path a

G_Cing_MK6240_PVC_baseline_scale ~ a*G_Cing_ER176_PVC_baseline_scale + TSPO_Affinity_Mixed + TSPO_Affinity_High + Age_baseline_scale + Sex + BMI_baseline_scale + APOE4_Carrier

# path b

G_Cing_Volume_MK6240_baseline_scale ~ b*G_Cing_MK6240_PVC_baseline_scale + ICV_baseline_scale + APOE4_Carrier

#Defined Parameters:

mediation := a*b

total := c+(a*b)

"

fit_G_Cing_T_N_full3_TSPO_mediation_std_flipped<-sem(G_Cing_T_N_full3_TSPO_mediation_std_flipped,

dat.wide)

fit_G_Cing_T_N_full3_TSPO_mediation_std_flipped_summary <- summary(fit_G_Cing_T_N_full3_TSPO_mediation_std_flipped,

fit.measures=TRUE,standardized=TRUE,rsquare=TRUE)

fit_G_Cing_T_N_full3_TSPO_mediation_std_flipped_parameters <- parameterEstimates(fit_G_Cing_T_N_full3_TSPO_mediation_std_flipped,

ci=TRUE,level=0.95,boot.ci.type="perc",standardized=TRUE)

Hippocampus_T_N_full3_TSPO_mediation_std_flipped =

"

#Regressions

# specify direct effect, path c'

Hippocampus_Volume_MK6240_baseline_scale ~ c*Hippocampus_ER176_PVC_baseline_scale + TSPO_Affinity_Mixed + TSPO_Affinity_High + ICV_baseline_scale + Age_baseline_scale + Sex + BMI_baseline_scale + APOE4_Carrier

# path a

Hippocampus_MK6240_PVC_baseline_scale ~ a*Hippocampus_ER176_PVC_baseline_scale + TSPO_Affinity_Mixed + TSPO_Affinity_High + Age_baseline_scale + Sex + BMI_baseline_scale + APOE4_Carrier

# path b

Hippocampus_Volume_MK6240_baseline_scale ~ b*Hippocampus_MK6240_PVC_baseline_scale + ICV_baseline_scale + APOE4_Carrier

#Defined Parameters:

mediation := a*b

total := c+(a*b)

"

fit_Hippocampus_T_N_full3_TSPO_mediation_std_flipped<-sem(Hippocampus_T_N_full3_TSPO_mediation_std_flipped,

dat.wide)

fit_Hippocampus_T_N_full3_TSPO_mediation_std_flipped_summary <- summary(fit_Hippocampus_T_N_full3_TSPO_mediation_std_flipped,

fit.measures=TRUE,standardized=TRUE,rsquare=TRUE)

fit_Hippocampus_T_N_full3_TSPO_mediation_std_flipped_parameters <- parameterEstimates(fit_Hippocampus_T_N_full3_TSPO_mediation_std_flipped,

ci=TRUE,level=0.95,boot.ci.type="perc",standardized=TRUE)

InferiorParietal_T_N_full3_TSPO_mediation_std_flipped =

"

#Regressions

# specify direct effect, path c'

InferiorParietal_Volume_MK6240_baseline_scale ~ c*InferiorParietal_ER176_PVC_baseline_scale + TSPO_Affinity_Mixed + TSPO_Affinity_High + ICV_baseline_scale + Age_baseline_scale + Sex + BMI_baseline_scale + APOE4_Carrier

# path a

InferiorParietal_MK6240_PVC_baseline_scale ~ a*InferiorParietal_ER176_PVC_baseline_scale + TSPO_Affinity_Mixed + TSPO_Affinity_High + Age_baseline_scale + Sex + BMI_baseline_scale + APOE4_Carrier

# path b

InferiorParietal_Volume_MK6240_baseline_scale ~ b*InferiorParietal_MK6240_PVC_baseline_scale + ICV_baseline_scale + APOE4_Carrier

#Defined Parameters:

mediation := a*b

total := c+(a*b)

"

fit_InferiorParietal_T_N_full3_TSPO_mediation_std_flipped<-sem(InferiorParietal_T_N_full3_TSPO_mediation_std_flipped,

dat.wide)

fit_InferiorParietal_T_N_full3_TSPO_mediation_std_flipped_summary <- summary(fit_InferiorParietal_T_N_full3_TSPO_mediation_std_flipped,

fit.measures=TRUE,standardized=TRUE,rsquare=TRUE)

fit_InferiorParietal_T_N_full3_TSPO_mediation_std_flipped_parameters <- parameterEstimates(fit_InferiorParietal_T_N_full3_TSPO_mediation_std_flipped,

ci=TRUE,level=0.95,boot.ci.type="perc",standardized=TRUE)

Insula_T_N_full3_TSPO_mediation_std_flipped =

"

#Regressions

# specify direct effect, path c'

Insula_Volume_MK6240_baseline_scale ~ c*Insula_ER176_PVC_baseline_scale + TSPO_Affinity_Mixed + TSPO_Affinity_High + ICV_baseline_scale + Age_baseline_scale + Sex + BMI_baseline_scale + APOE4_Carrier

# path a

Insula_MK6240_PVC_baseline_scale ~ a*Insula_ER176_PVC_baseline_scale + TSPO_Affinity_Mixed + TSPO_Affinity_High + Age_baseline_scale + Sex + BMI_baseline_scale + APOE4_Carrier

# path b

Insula_Volume_MK6240_baseline_scale ~ b*Insula_MK6240_PVC_baseline_scale + ICV_baseline_scale + APOE4_Carrier

#Defined Parameters:

mediation := a*b

total := c+(a*b)

"

fit_Insula_T_N_full3_TSPO_mediation_std_flipped<-sem(Insula_T_N_full3_TSPO_mediation_std_flipped,

dat.wide)

fit_Insula_T_N_full3_TSPO_mediation_std_flipped_summary <- summary(fit_Insula_T_N_full3_TSPO_mediation_std_flipped,

fit.measures=TRUE,standardized=TRUE,rsquare=TRUE)

fit_Insula_T_N_full3_TSPO_mediation_std_flipped_parameters <- parameterEstimates(fit_Insula_T_N_full3_TSPO_mediation_std_flipped,

ci=TRUE,level=0.95,boot.ci.type="perc",standardized=TRUE)

LingualGyrus_T_N_full3_TSPO_mediation_std_flipped =

"

#Regressions

# specify direct effect, path c'

LingualGyrus_Volume_MK6240_baseline_scale ~ c*LingualGyrus_ER176_PVC_baseline_scale + TSPO_Affinity_Mixed + TSPO_Affinity_High + ICV_baseline_scale + Age_baseline_scale + Sex + BMI_baseline_scale + APOE4_Carrier

# path a

LingualGyrus_MK6240_PVC_baseline_scale ~ a*LingualGyrus_ER176_PVC_baseline_scale + TSPO_Affinity_Mixed + TSPO_Affinity_High + Age_baseline_scale + Sex + BMI_baseline_scale + APOE4_Carrier

# path b

LingualGyrus_Volume_MK6240_baseline_scale ~ b*LingualGyrus_MK6240_PVC_baseline_scale + ICV_baseline_scale + APOE4_Carrier

#Defined Parameters:

mediation := a*b

total := c+(a*b)

"

fit_LingualGyrus_T_N_full3_TSPO_mediation_std_flipped<-sem(LingualGyrus_T_N_full3_TSPO_mediation_std_flipped,

dat.wide)

fit_LingualGyrus_T_N_full3_TSPO_mediation_std_flipped_summary <- summary(fit_LingualGyrus_T_N_full3_TSPO_mediation_std_flipped,

fit.measures=TRUE,standardized=TRUE,rsquare=TRUE)

fit_LingualGyrus_T_N_full3_TSPO_mediation_std_flipped_parameters <- parameterEstimates(fit_LingualGyrus_T_N_full3_TSPO_mediation_std_flipped,

ci=TRUE,level=0.95,boot.ci.type="perc",standardized=TRUE)

MiddleInfTempGyrus_T_N_full3_TSPO_mediation_std_flipped =

"

#Regressions

# specify direct effect, path c'

MiddleInfTempGyrus_Volume_MK6240_baseline_scale ~ c*MiddleInfTempGyrus_ER176_PVC_baseline_scale + TSPO_Affinity_Mixed + TSPO_Affinity_High + ICV_baseline_scale + Age_baseline_scale + Sex + BMI_baseline_scale + APOE4_Carrier

# path a

MiddleInfTempGyrus_MK6240_PVC_baseline_scale ~ a*MiddleInfTempGyrus_ER176_PVC_baseline_scale + TSPO_Affinity_Mixed + TSPO_Affinity_High + Age_baseline_scale + Sex + BMI_baseline_scale + APOE4_Carrier

# path b

MiddleInfTempGyrus_Volume_MK6240_baseline_scale ~ b*MiddleInfTempGyrus_MK6240_PVC_baseline_scale + ICV_baseline_scale + APOE4_Carrier

#Defined Parameters:

mediation := a*b

total := c+(a*b)

"

fit_MiddleInfTempGyrus_T_N_full3_TSPO_mediation_std_flipped<-sem(MiddleInfTempGyrus_T_N_full3_TSPO_mediation_std_flipped,

dat.wide)

fit_MiddleInfTempGyrus_T_N_full3_TSPO_mediation_std_flipped_summary <- summary(fit_MiddleInfTempGyrus_T_N_full3_TSPO_mediation_std_flipped,

fit.measures=TRUE,standardized=TRUE,rsquare=TRUE)

fit_MiddleInfTempGyrus_T_N_full3_TSPO_mediation_std_flipped_parameters <- parameterEstimates(fit_MiddleInfTempGyrus_T_N_full3_TSPO_mediation_std_flipped,

ci=TRUE,level=0.95,boot.ci.type="perc",standardized=TRUE)

Prefrontal_T_N_full3_TSPO_mediation_std_flipped =

"

#Regressions

# specify direct effect, path c'

Prefrontal_Volume_MK6240_baseline_scale ~ c*Prefrontal_ER176_PVC_baseline_scale + TSPO_Affinity_Mixed + TSPO_Affinity_High + ICV_baseline_scale + Age_baseline_scale + Sex + BMI_baseline_scale + APOE4_Carrier

# path a

Prefrontal_MK6240_PVC_baseline_scale ~ a*Prefrontal_ER176_PVC_baseline_scale + TSPO_Affinity_Mixed + TSPO_Affinity_High + Age_baseline_scale + Sex + BMI_baseline_scale + APOE4_Carrier

# path b

Prefrontal_Volume_MK6240_baseline_scale ~ b*Prefrontal_MK6240_PVC_baseline_scale + ICV_baseline_scale + APOE4_Carrier

#Defined Parameters:

mediation := a*b

total := c+(a*b)

"

fit_Prefrontal_T_N_full3_TSPO_mediation_std_flipped<-sem(Prefrontal_T_N_full3_TSPO_mediation_std_flipped,

dat.wide)

fit_Prefrontal_T_N_full3_TSPO_mediation_std_flipped_summary <- summary(fit_Prefrontal_T_N_full3_TSPO_mediation_std_flipped,

fit.measures=TRUE,standardized=TRUE,rsquare=TRUE)

fit_Prefrontal_T_N_full3_TSPO_mediation_std_flipped_parameters <- parameterEstimates(fit_Prefrontal_T_N_full3_TSPO_mediation_std_flipped,

ci=TRUE,level=0.95,boot.ci.type="perc",standardized=TRUE)

SuperiorParietal_T_N_full3_TSPO_mediation_std_flipped =

"

#Regressions

# specify direct effect, path c'

SuperiorParietal_Volume_MK6240_baseline_scale ~ c*SuperiorParietal_ER176_PVC_baseline_scale + TSPO_Affinity_Mixed + TSPO_Affinity_High + ICV_baseline_scale + Age_baseline_scale + Sex + BMI_baseline_scale + APOE4_Carrier

# path a

SuperiorParietal_MK6240_PVC_baseline_scale ~ a*SuperiorParietal_ER176_PVC_baseline_scale + TSPO_Affinity_Mixed + TSPO_Affinity_High + Age_baseline_scale + Sex + BMI_baseline_scale + APOE4_Carrier

# path b

SuperiorParietal_Volume_MK6240_baseline_scale ~ b*SuperiorParietal_MK6240_PVC_baseline_scale + ICV_baseline_scale + APOE4_Carrier

#Defined Parameters:

mediation := a*b

total := c+(a*b)

"

fit_SuperiorParietal_T_N_full3_TSPO_mediation_std_flipped<-sem(SuperiorParietal_T_N_full3_TSPO_mediation_std_flipped,

dat.wide)

fit_SuperiorParietal_T_N_full3_TSPO_mediation_std_flipped_summary <- summary(fit_SuperiorParietal_T_N_full3_TSPO_mediation_std_flipped,

fit.measures=TRUE,standardized=TRUE,rsquare=TRUE)

fit_SuperiorParietal_T_N_full3_TSPO_mediation_std_flipped_parameters <- parameterEstimates(fit_SuperiorParietal_T_N_full3_TSPO_mediation_std_flipped,

ci=TRUE,level=0.95,boot.ci.type="perc",standardized=TRUE)

SuperiorTemp_T_N_full3_TSPO_mediation_std_flipped =

"

#Regressions

# specify direct effect, path c'

SuperiorTemp_Volume_MK6240_baseline_scale ~ c*SuperiorTemp_ER176_PVC_baseline_scale + TSPO_Affinity_Mixed + TSPO_Affinity_High + ICV_baseline_scale + Age_baseline_scale + Sex + BMI_baseline_scale + APOE4_Carrier

# path a

SuperiorTemp_MK6240_PVC_baseline_scale ~ a*SuperiorTemp_ER176_PVC_baseline_scale + TSPO_Affinity_Mixed + TSPO_Affinity_High + Age_baseline_scale + Sex + BMI_baseline_scale + APOE4_Carrier

# path b

SuperiorTemp_Volume_MK6240_baseline_scale ~ b*SuperiorTemp_MK6240_PVC_baseline_scale + ICV_baseline_scale + APOE4_Carrier

#Defined Parameters:

mediation := a*b

total := c+(a*b)

"

fit_SuperiorTemp_T_N_full3_TSPO_mediation_std_flipped<-sem(SuperiorTemp_T_N_full3_TSPO_mediation_std_flipped,

dat.wide)

fit_SuperiorTemp_T_N_full3_TSPO_mediation_std_flipped_summary <- summary(fit_SuperiorTemp_T_N_full3_TSPO_mediation_std_flipped,

fit.measures=TRUE,standardized=TRUE,rsquare=TRUE)

fit_SuperiorTemp_T_N_full3_TSPO_mediation_std_flipped_parameters <- parameterEstimates(fit_SuperiorTemp_T_N_full3_TSPO_mediation_std_flipped,

ci=TRUE,level=0.95,boot.ci.type="perc",standardized=TRUE)

## adjust p values

TN_rois <- c("Prefrontal","Insula","G_Cing","FusiformGyrus","LingualGyrus",

"EntorhinalCortex_FS","MiddleInfTempGyrus","SuperiorTemp",

"InferiorParietal","SuperiorParietal",

"Amygdala","Hippocampus","Striatum")

# Tau to TSPO

TN_TSPO_mediation_flipped_A_p <- c(fit_Prefrontal_T_N_full3_TSPO_mediation_flipped_parameters[fit_Prefrontal_T_N_full3_TSPO_mediation_flipped_parameters$label=="a","pvalue"],

fit_Insula_T_N_full3_TSPO_mediation_flipped_parameters[fit_Insula_T_N_full3_TSPO_mediation_flipped_parameters$label=="a","pvalue"],

fit_G_Cing_T_N_full3_TSPO_mediation_flipped_parameters[fit_G_Cing_T_N_full3_TSPO_mediation_flipped_parameters$label=="a","pvalue"],

fit_FusiformGyrus_T_N_full3_TSPO_mediation_flipped_parameters[fit_FusiformGyrus_T_N_full3_TSPO_mediation_flipped_parameters$label=="a","pvalue"],

fit_LingualGyrus_T_N_full3_TSPO_mediation_flipped_parameters[fit_LingualGyrus_T_N_full3_TSPO_mediation_flipped_parameters$label=="a","pvalue"],

fit_EntorhinalCortex_FS_T_N_full3_TSPO_mediation_flipped_parameters[fit_EntorhinalCortex_FS_T_N_full3_TSPO_mediation_flipped_parameters$label=="a","pvalue"],

fit_MiddleInfTempGyrus_T_N_full3_TSPO_mediation_flipped_parameters[fit_MiddleInfTempGyrus_T_N_full3_TSPO_mediation_flipped_parameters$label=="a","pvalue"],

fit_SuperiorTemp_T_N_full3_TSPO_mediation_flipped_parameters[fit_SuperiorTemp_T_N_full3_TSPO_mediation_flipped_parameters$label=="a","pvalue"],

fit_InferiorParietal_T_N_full3_TSPO_mediation_flipped_parameters[fit_InferiorParietal_T_N_full3_TSPO_mediation_flipped_parameters$label=="a","pvalue"],

fit_SuperiorParietal_T_N_full3_TSPO_mediation_flipped_parameters[fit_SuperiorParietal_T_N_full3_TSPO_mediation_flipped_parameters$label=="a","pvalue"],

fit_Amygdala_T_N_full3_TSPO_mediation_flipped_parameters[fit_Amygdala_T_N_full3_TSPO_mediation_flipped_parameters$label=="a","pvalue"],

fit_Hippocampus_T_N_full3_TSPO_mediation_flipped_parameters[fit_Hippocampus_T_N_full3_TSPO_mediation_flipped_parameters$label=="a","pvalue"],

fit_Striatum_T_N_full3_TSPO_mediation_flipped_parameters[fit_Striatum_T_N_full3_TSPO_mediation_flipped_parameters$label=="a","pvalue"])

TN_TSPO_mediation_flipped_A_p_adjusted <- p.adjust(TN_TSPO_mediation_flipped_A_p, method = "BH", n = length(TN_TSPO_mediation_flipped_A_p))

TN_TSPO_mediation_flipped_A_p_adjusted_roi <- cbind(TN_rois,TN_TSPO_mediation_flipped_A_p_adjusted)

TN_TSPO_mediation_flipped_A_p_adjusted_df <- as.data.frame(TN_TSPO_mediation_flipped_A_p_adjusted_roi)

# TSPO to Neurodegeneration

TN_TSPO_mediation_flipped_B_p <- c(fit_Prefrontal_T_N_full3_TSPO_mediation_flipped_parameters[fit_Prefrontal_T_N_full3_TSPO_mediation_flipped_parameters$label=="b","pvalue"],

fit_Insula_T_N_full3_TSPO_mediation_flipped_parameters[fit_Insula_T_N_full3_TSPO_mediation_flipped_parameters$label=="b","pvalue"],

fit_G_Cing_T_N_full3_TSPO_mediation_flipped_parameters[fit_G_Cing_T_N_full3_TSPO_mediation_flipped_parameters$label=="b","pvalue"],

fit_FusiformGyrus_T_N_full3_TSPO_mediation_flipped_parameters[fit_FusiformGyrus_T_N_full3_TSPO_mediation_flipped_parameters$label=="b","pvalue"],

fit_LingualGyrus_T_N_full3_TSPO_mediation_flipped_parameters[fit_LingualGyrus_T_N_full3_TSPO_mediation_flipped_parameters$label=="b","pvalue"],

fit_EntorhinalCortex_FS_T_N_full3_TSPO_mediation_flipped_parameters[fit_EntorhinalCortex_FS_T_N_full3_TSPO_mediation_flipped_parameters$label=="b","pvalue"],

fit_MiddleInfTempGyrus_T_N_full3_TSPO_mediation_flipped_parameters[fit_MiddleInfTempGyrus_T_N_full3_TSPO_mediation_flipped_parameters$label=="b","pvalue"],

fit_SuperiorTemp_T_N_full3_TSPO_mediation_flipped_parameters[fit_SuperiorTemp_T_N_full3_TSPO_mediation_flipped_parameters$label=="b","pvalue"],

fit_InferiorParietal_T_N_full3_TSPO_mediation_flipped_parameters[fit_InferiorParietal_T_N_full3_TSPO_mediation_flipped_parameters$label=="b","pvalue"],

fit_SuperiorParietal_T_N_full3_TSPO_mediation_flipped_parameters[fit_SuperiorParietal_T_N_full3_TSPO_mediation_flipped_parameters$label=="b","pvalue"],

fit_Amygdala_T_N_full3_TSPO_mediation_flipped_parameters[fit_Amygdala_T_N_full3_TSPO_mediation_flipped_parameters$label=="b","pvalue"],

fit_Hippocampus_T_N_full3_TSPO_mediation_flipped_parameters[fit_Hippocampus_T_N_full3_TSPO_mediation_flipped_parameters$label=="b","pvalue"],

fit_Striatum_T_N_full3_TSPO_mediation_flipped_parameters[fit_Striatum_T_N_full3_TSPO_mediation_flipped_parameters$label=="b","pvalue"])

TN_TSPO_mediation_flipped_B_p_adjusted <- p.adjust(TN_TSPO_mediation_flipped_B_p, method = "BH", n = length(TN_TSPO_mediation_flipped_B_p))

TN_TSPO_mediation_flipped_B_p_adjusted_roi <- cbind(TN_rois,TN_TSPO_mediation_flipped_B_p_adjusted)

TN_TSPO_mediation_flipped_B_p_adjusted_df <- as.data.frame(TN_TSPO_mediation_flipped_B_p_adjusted_roi)

# Tau to Neurodegeneration

TN_TSPO_mediation_flipped_C_p <- c(fit_Prefrontal_T_N_full3_TSPO_mediation_flipped_parameters[fit_Prefrontal_T_N_full3_TSPO_mediation_flipped_parameters$label=="c","pvalue"],

fit_Insula_T_N_full3_TSPO_mediation_flipped_parameters[fit_Insula_T_N_full3_TSPO_mediation_flipped_parameters$label=="c","pvalue"],

fit_G_Cing_T_N_full3_TSPO_mediation_flipped_parameters[fit_G_Cing_T_N_full3_TSPO_mediation_flipped_parameters$label=="c","pvalue"],

fit_FusiformGyrus_T_N_full3_TSPO_mediation_flipped_parameters[fit_FusiformGyrus_T_N_full3_TSPO_mediation_flipped_parameters$label=="c","pvalue"],

fit_LingualGyrus_T_N_full3_TSPO_mediation_flipped_parameters[fit_LingualGyrus_T_N_full3_TSPO_mediation_flipped_parameters$label=="c","pvalue"],

fit_EntorhinalCortex_FS_T_N_full3_TSPO_mediation_flipped_parameters[fit_EntorhinalCortex_FS_T_N_full3_TSPO_mediation_flipped_parameters$label=="c","pvalue"],

fit_MiddleInfTempGyrus_T_N_full3_TSPO_mediation_flipped_parameters[fit_MiddleInfTempGyrus_T_N_full3_TSPO_mediation_flipped_parameters$label=="c","pvalue"],

fit_SuperiorTemp_T_N_full3_TSPO_mediation_flipped_parameters[fit_SuperiorTemp_T_N_full3_TSPO_mediation_flipped_parameters$label=="c","pvalue"],

fit_InferiorParietal_T_N_full3_TSPO_mediation_flipped_parameters[fit_InferiorParietal_T_N_full3_TSPO_mediation_flipped_parameters$label=="c","pvalue"],

fit_SuperiorParietal_T_N_full3_TSPO_mediation_flipped_parameters[fit_SuperiorParietal_T_N_full3_TSPO_mediation_flipped_parameters$label=="c","pvalue"],

fit_Amygdala_T_N_full3_TSPO_mediation_flipped_parameters[fit_Amygdala_T_N_full3_TSPO_mediation_flipped_parameters$label=="c","pvalue"],

fit_Hippocampus_T_N_full3_TSPO_mediation_flipped_parameters[fit_Hippocampus_T_N_full3_TSPO_mediation_flipped_parameters$label=="c","pvalue"],

fit_Striatum_T_N_full3_TSPO_mediation_flipped_parameters[fit_Striatum_T_N_full3_TSPO_mediation_flipped_parameters$label=="c","pvalue"])

TN_TSPO_mediation_flipped_C_p_adjusted <- p.adjust(TN_TSPO_mediation_flipped_C_p, method = "BH", n = length(TN_TSPO_mediation_flipped_C_p))

TN_TSPO_mediation_flipped_C_p_adjusted_roi <- cbind(TN_rois,TN_TSPO_mediation_flipped_C_p_adjusted)

TN_TSPO_mediation_flipped_C_p_adjusted_df <- as.data.frame(TN_TSPO_mediation_flipped_C_p_adjusted_roi)

# Tau to TSPO to Neurodegeneration

TN_TSPO_mediation_flipped_Cprime_p <- c(fit_Prefrontal_T_N_full3_TSPO_mediation_flipped_parameters[fit_Prefrontal_T_N_full3_TSPO_mediation_flipped_parameters$label=="mediation","pvalue"],

fit_Insula_T_N_full3_TSPO_mediation_flipped_parameters[fit_Insula_T_N_full3_TSPO_mediation_flipped_parameters$label=="mediation","pvalue"],

fit_G_Cing_T_N_full3_TSPO_mediation_flipped_parameters[fit_G_Cing_T_N_full3_TSPO_mediation_flipped_parameters$label=="mediation","pvalue"],

fit_FusiformGyrus_T_N_full3_TSPO_mediation_flipped_parameters[fit_FusiformGyrus_T_N_full3_TSPO_mediation_flipped_parameters$label=="mediation","pvalue"],

fit_LingualGyrus_T_N_full3_TSPO_mediation_flipped_parameters[fit_LingualGyrus_T_N_full3_TSPO_mediation_flipped_parameters$label=="mediation","pvalue"],

fit_EntorhinalCortex_FS_T_N_full3_TSPO_mediation_flipped_parameters[fit_EntorhinalCortex_FS_T_N_full3_TSPO_mediation_flipped_parameters$label=="mediation","pvalue"],

fit_MiddleInfTempGyrus_T_N_full3_TSPO_mediation_flipped_parameters[fit_MiddleInfTempGyrus_T_N_full3_TSPO_mediation_flipped_parameters$label=="mediation","pvalue"],

fit_SuperiorTemp_T_N_full3_TSPO_mediation_flipped_parameters[fit_SuperiorTemp_T_N_full3_TSPO_mediation_flipped_parameters$label=="mediation","pvalue"],

fit_InferiorParietal_T_N_full3_TSPO_mediation_flipped_parameters[fit_InferiorParietal_T_N_full3_TSPO_mediation_flipped_parameters$label=="mediation","pvalue"],

fit_SuperiorParietal_T_N_full3_TSPO_mediation_flipped_parameters[fit_SuperiorParietal_T_N_full3_TSPO_mediation_flipped_parameters$label=="mediation","pvalue"],

fit_Amygdala_T_N_full3_TSPO_mediation_flipped_parameters[fit_Amygdala_T_N_full3_TSPO_mediation_flipped_parameters$label=="mediation","pvalue"],

fit_Hippocampus_T_N_full3_TSPO_mediation_flipped_parameters[fit_Hippocampus_T_N_full3_TSPO_mediation_flipped_parameters$label=="mediation","pvalue"],

fit_Striatum_T_N_full3_TSPO_mediation_flipped_parameters[fit_Striatum_T_N_full3_TSPO_mediation_flipped_parameters$label=="mediation","pvalue"])

TN_TSPO_mediation_flipped_Cprime_p_adjusted <- p.adjust(TN_TSPO_mediation_flipped_Cprime_p, method = "BH", n = length(TN_TSPO_mediation_flipped_Cprime_p))

TN_TSPO_mediation_flipped_Cprime_p_adjusted_roi <- cbind(TN_rois,TN_TSPO_mediation_flipped_Cprime_p_adjusted)

TN_TSPO_mediation_flipped_Cprime_p_adjusted_df <- as.data.frame(TN_TSPO_mediation_flipped_Cprime_p_adjusted_roi)

# Total

TN_TSPO_mediation_flipped_Total_p <- c(fit_Prefrontal_T_N_full3_TSPO_mediation_flipped_parameters[fit_Prefrontal_T_N_full3_TSPO_mediation_flipped_parameters$label=="total","pvalue"],

fit_Insula_T_N_full3_TSPO_mediation_flipped_parameters[fit_Insula_T_N_full3_TSPO_mediation_flipped_parameters$label=="total","pvalue"],

fit_G_Cing_T_N_full3_TSPO_mediation_flipped_parameters[fit_G_Cing_T_N_full3_TSPO_mediation_flipped_parameters$label=="total","pvalue"],

fit_FusiformGyrus_T_N_full3_TSPO_mediation_flipped_parameters[fit_FusiformGyrus_T_N_full3_TSPO_mediation_flipped_parameters$label=="total","pvalue"],

fit_LingualGyrus_T_N_full3_TSPO_mediation_flipped_parameters[fit_LingualGyrus_T_N_full3_TSPO_mediation_flipped_parameters$label=="total","pvalue"],

fit_EntorhinalCortex_FS_T_N_full3_TSPO_mediation_flipped_parameters[fit_EntorhinalCortex_FS_T_N_full3_TSPO_mediation_flipped_parameters$label=="total","pvalue"],

fit_MiddleInfTempGyrus_T_N_full3_TSPO_mediation_flipped_parameters[fit_MiddleInfTempGyrus_T_N_full3_TSPO_mediation_flipped_parameters$label=="total","pvalue"],

fit_SuperiorTemp_T_N_full3_TSPO_mediation_flipped_parameters[fit_SuperiorTemp_T_N_full3_TSPO_mediation_flipped_parameters$label=="total","pvalue"],

fit_InferiorParietal_T_N_full3_TSPO_mediation_flipped_parameters[fit_InferiorParietal_T_N_full3_TSPO_mediation_flipped_parameters$label=="total","pvalue"],

fit_SuperiorParietal_T_N_full3_TSPO_mediation_flipped_parameters[fit_SuperiorParietal_T_N_full3_TSPO_mediation_flipped_parameters$label=="total","pvalue"],

fit_Amygdala_T_N_full3_TSPO_mediation_flipped_parameters[fit_Amygdala_T_N_full3_TSPO_mediation_flipped_parameters$label=="total","pvalue"],

fit_Hippocampus_T_N_full3_TSPO_mediation_flipped_parameters[fit_Hippocampus_T_N_full3_TSPO_mediation_flipped_parameters$label=="total","pvalue"],

fit_Striatum_T_N_full3_TSPO_mediation_flipped_parameters[fit_Striatum_T_N_full3_TSPO_mediation_flipped_parameters$label=="total","pvalue"])

TN_TSPO_mediation_flipped_Total_p_adjusted <- p.adjust(TN_TSPO_mediation_flipped_Total_p, method = "BH", n = length(TN_TSPO_mediation_flipped_Total_p))

TN_TSPO_mediation_flipped_Total_p_adjusted_roi <- cbind(TN_rois,TN_TSPO_mediation_flipped_Total_p_adjusted)

TN_TSPO_mediation_flipped_Total_p_adjusted_df <- as.data.frame(TN_TSPO_mediation_flipped_Total_p_adjusted_roi)

### Sensitivity: transform

## Hypothesis 1, TSPO as example

TSPO_Status_lm_full3<-glm(ER176 ~ Status_baseline*roi + TSPO_Affinity + Age_baseline + Sex + BMI_baseline + APOE4, family=Gamma(link="log"),

analysis.dat.long %>% filter(roi %in% roilist))

TSPO_Status_lm_full3_summary<-summary(TSPO_Status_lm_full3)

TSPO_Status_lm_full3_mean <- emmeans(TSPO_Status_lm_full3, spec = pairwise ~ Status_baseline | roi,

weights="proportional",adjust="mvt") %>% summary(infer=TRUE)

# save as data frame to extract estimates

TSPO_Status_lm_full3_mean_df <- as.data.frame(TSPO_Status_lm_full3_mean)

# tspo std

pwpm(emmeans(TSPO_Status_lm_full3, spec = pairwise ~ Status_baseline | roi,

weights="proportional",adjust="mvt"))

TSPO_Status_lm_full3_std_mean <- as.data.frame(eff_size(emmeans(TSPO_Status_lm_full3, spec = pairwise ~ Status_baseline | roi,

weights="proportional",adjust="mvt"), sigma = sigma(TSPO_Status_lm_full3), edf = df.residual(TSPO_Status_lm_full3)))

TSPO_Status_lm_full3_std_mean_df <- as.data.frame(TSPO_Status_lm_full3_std_mean)

## Hypothesis 2

all_lm <- (lm(ER176 ~ MK6240*roi + FBB*roi + Volume_MK6240*roi + ICV_baseline + TSPO_Affinity + Age_baseline + Sex + BMI_baseline + APOE4, family=Gamma(link="log"),

analysis.dat.long %>% filter(roi %in% roilist)))

## Hypothesis 3

# log transform all variables before mediations

dat.wide$Braak_I_II_MK6240_PVC_baseline_scale = scale(log(dat.wide$Braak_I_II_MK6240_PVC_baseline))

dat.wide$Braak_III_IV_MK6240_PVC_baseline_scale = scale(log(dat.wide$Braak_III_IV_MK6240_PVC_baseline))

dat.wide$Braak_V_VI_MK6240_PVC_baseline_scale = scale(log(dat.wide$Braak_V_VI_MK6240_PVC_baseline))

dat.wide$Composite_FBB_baseline_scale = scale(log(dat.wide$Composite_FBB_baseline))

dat.wide$Composite_ER176_PVC_baseline_scale = scale(log(dat.wide$Composite_ER176_PVC_baseline))

dat.wide$Striatum_Volume_MK6240_baseline_scale <- scale(log(dat.wide$Striatum_Volume_MK6240_baseline))

dat.wide$Amygdala_Volume_MK6240_baseline_scale <- scale(log(dat.wide$Amygdala_Volume_MK6240_baseline))

dat.wide$EntorhinalCortex_FS_Volume_MK6240_baseline_scale <- scale(log(dat.wide$EntorhinalCortex_FS_Volume_MK6240_baseline))

dat.wide$FusiformGyrus_Volume_MK6240_baseline_scale <- scale(log(dat.wide$FusiformGyrus_Volume_MK6240_baseline))

dat.wide$G_Cing_Volume_MK6240_baseline_scale <- scale(log(dat.wide$G_Cing_Volume_MK6240_baseline))

dat.wide$Hippocampus_Volume_MK6240_baseline_scale <- scale(log(dat.wide$Hippocampus_Volume_MK6240_baseline))

dat.wide$InferiorParietal_Volume_MK6240_baseline_scale <- scale(log(dat.wide$InferiorParietal_Volume_MK6240_baseline))

dat.wide$Insula_Volume_MK6240_baseline_scale <- scale(log(dat.wide$Insula_Volume_MK6240_baseline))

dat.wide$LingualGyrus_Volume_MK6240_baseline_scale <- scale(log(dat.wide$LingualGyrus_Volume_MK6240_baseline))

dat.wide$MiddleInfTempGyrus_Volume_MK6240_baseline_scale <- scale(log(dat.wide$MiddleInfTempGyrus_Volume_MK6240_baseline))

dat.wide$Prefrontal_Volume_MK6240_baseline_scale <- scale(log(dat.wide$Prefrontal_Volume_MK6240_baseline))

dat.wide$SuperiorParietal_Volume_MK6240_baseline_scale <- scale(log(dat.wide$SuperiorParietal_Volume_MK6240_baseline))

dat.wide$SuperiorTemp_Volume_MK6240_baseline_scale <- scale(log(dat.wide$SuperiorTemp_Volume_MK6240_baseline))

dat.wide$Striatum_ER176_PVC_baseline_scale <- scale(log(dat.wide$Striatum_ER176_PVC_baseline))

dat.wide$Amygdala_ER176_PVC_baseline_scale <- scale(log(dat.wide$Amygdala_ER176_PVC_baseline))

dat.wide$EntorhinalCortex_FS_ER176_PVC_baseline_scale <- scale(log(dat.wide$EntorhinalCortex_FS_ER176_PVC_baseline))

dat.wide$FusiformGyrus_ER176_PVC_baseline_scale <- scale(log(dat.wide$FusiformGyrus_ER176_PVC_baseline))

dat.wide$G_Cing_ER176_PVC_baseline_scale <- scale(log(dat.wide$G_Cing_ER176_PVC_baseline))

dat.wide$Hippocampus_ER176_PVC_baseline_scale <- scale(log(dat.wide$Hippocampus_ER176_PVC_baseline))

dat.wide$InferiorParietal_ER176_PVC_baseline_scale <- scale(log(dat.wide$InferiorParietal_ER176_PVC_baseline))

dat.wide$Insula_ER176_PVC_baseline_scale <- scale(log(dat.wide$Insula_ER176_PVC_baseline))

dat.wide$LingualGyrus_ER176_PVC_baseline_scale <- scale(log(dat.wide$LingualGyrus_ER176_PVC_baseline))

dat.wide$MiddleInfTempGyrus_ER176_PVC_baseline_scale <- scale(log(dat.wide$MiddleInfTempGyrus_ER176_PVC_baseline))

dat.wide$Prefrontal_ER176_PVC_baseline_scale <- scale(log(dat.wide$Prefrontal_ER176_PVC_baseline))

dat.wide$SuperiorParietal_ER176_PVC_baseline_scale <- scale(log(dat.wide$SuperiorParietal_ER176_PVC_baseline))

dat.wide$SuperiorTemp_ER176_PVC_baseline_scale <- scale(log(dat.wide$SuperiorTemp_ER176_PVC_baseline))

dat.wide$Striatum_MK6240_PVC_baseline_scale <- scale(log(dat.wide$Striatum_MK6240_PVC_baseline))

dat.wide$Amygdala_MK6240_PVC_baseline_scale <- scale(log(dat.wide$Amygdala_MK6240_PVC_baseline))

dat.wide$EntorhinalCortex_FS_MK6240_PVC_baseline_scale <- scale(log(dat.wide$EntorhinalCortex_FS_MK6240_PVC_baseline))

dat.wide$FusiformGyrus_MK6240_PVC_baseline_scale <- scale(log(dat.wide$FusiformGyrus_MK6240_PVC_baseline))

dat.wide$G_Cing_MK6240_PVC_baseline_scale <- scale(log(dat.wide$G_Cing_MK6240_PVC_baseline))

dat.wide$Hippocampus_MK6240_PVC_baseline_scale <- scale(log(dat.wide$Hippocampus_MK6240_PVC_baseline))

dat.wide$InferiorParietal_MK6240_PVC_baseline_scale <- scale(log(dat.wide$InferiorParietal_MK6240_PVC_baseline))

dat.wide$Insula_MK6240_PVC_baseline_scale <- scale(log(dat.wide$Insula_MK6240_PVC_baseline))

dat.wide$LingualGyrus_MK6240_PVC_baseline_scale <- scale(log(dat.wide$LingualGyrus_MK6240_PVC_baseline))

dat.wide$MiddleInfTempGyrus_MK6240_PVC_baseline_scale <- scale(log(dat.wide$MiddleInfTempGyrus_MK6240_PVC_baseline))

dat.wide$Prefrontal_MK6240_PVC_baseline_scale <- scale(log(dat.wide$Prefrontal_MK6240_PVC_baseline))

dat.wide$SuperiorParietal_MK6240_PVC_baseline_scale <- scale(log(dat.wide$SuperiorParietal_MK6240_PVC_baseline))

dat.wide$SuperiorTemp_MK6240_PVC_baseline_scale <- scale(log(dat.wide$SuperiorTemp_MK6240_PVC_baseline))

dat.wide$Striatum_FBB_baseline_scale <- scale(log(dat.wide$Striatum_FBB_baseline))

dat.wide$Amygdala_FBB_baseline_scale <- scale(log(dat.wide$Amygdala_FBB_baseline))

dat.wide$EntorhinalCortex_FS_FBB_baseline_scale <- scale(log(dat.wide$EntorhinalCortex_FS_FBB_baseline))

dat.wide$FusiformGyrus_FBB_baseline_scale <- scale(log(dat.wide$FusiformGyrus_FBB_baseline))

dat.wide$G_Cing_FBB_baseline_scale <- scale(log(dat.wide$G_Cing_FBB_baseline))

dat.wide$Hippocampus_FBB_baseline_scale <- scale(log(dat.wide$Hippocampus_FBB_baseline))

dat.wide$InferiorParietal_FBB_baseline_scale <- scale(log(dat.wide$InferiorParietal_FBB_baseline))

dat.wide$Insula_FBB_baseline_scale <- scale(log(dat.wide$Insula_FBB_baseline))

dat.wide$LingualGyrus_FBB_baseline_scale <- scale(log(dat.wide$LingualGyrus_FBB_baseline))

dat.wide$MiddleInfTempGyrus_FBB_baseline_scale <- scale(log(dat.wide$MiddleInfTempGyrus_FBB_baseline))

dat.wide$Prefrontal_FBB_baseline_scale <- scale(log(dat.wide$Prefrontal_FBB_baseline))

dat.wide$SuperiorParietal_FBB_baseline_scale <- scale(log(dat.wide$SuperiorParietal_FBB_baseline))

dat.wide$SuperiorTemp_FBB_baseline_scale <- scale(log(dat.wide$SuperiorTemp_FBB_baseline))

## Sensitivity: univariate

# Tau

TSPO_Tau_lm_full3<-lm(ER176 ~ MK6240*roi + TSPO_Affinity + Age_baseline + Sex + BMI_baseline + APOE4,

analysis.dat.long %>% filter(roi %in% roilist))

TSPO_Tau_lm_full3_summary<-summary(TSPO_Tau_lm_full3)

TSPO_Tau_lm_full3_slopes <- emtrends(TSPO_Tau_lm_full3, ~ roi, var = "MK6240")

# get adjusted pvalues

TSPO_Tau_lm_full3_contrast <- contrast(TSPO_Tau_lm_full3_slopes,

method = list("Striatum" = c(1, rep(0,12)),

"Amygdala" = c(rep(0,1),1,rep(0,11)),

"EntorhinalCortex_FS" = c(rep(0,2),1,rep(0,10)),

"FusiformGyrus" = c(rep(0,3),1,rep(0,9)),

"G_Cing" = c(rep(0,4),1,rep(0,8)),

"Hippocampus" = c(rep(0,5),1,rep(0,7)),

"InferiorParietal" = c(rep(0,6),1,rep(0,6)),

"Insula" = c(rep(0,7),1,rep(0,5)),

"LingualGyrus" = c(rep(0,8),1,rep(0,4)),

"MiddleInfTempGyrus" = c(rep(0,9),1,rep(0,3)),

"Prefrontal" = c(rep(0,10),1,rep(0,2)),

"SuperiorParietal" = c(rep(0,11),1,rep(0,1)),

"SuperiorTemp" = c(rep(0,12),1)),

weights="proportional",adjust="mvt") %>%

summary(infer=TRUE)

# Tau std

TSPO_Tau_lm_full3_std_contrast <- as.data.frame(eff_size(emtrends(TSPO_Tau_lm_full3, ~roi, var = "MK6240", weights="proportional",adjust="mvt"),

sigma = sigma(TSPO_Tau_lm_full3), edf = TSPO_Tau_lm_full3_summary[[7]][[2]], method=list("Striatum" = c(1, rep(0,12)),

"Amygdala" = c(rep(0,1),1,rep(0,11)),

"EntorhinalCortex_FS" = c(rep(0,2),1,rep(0,10)),

"FusiformGyrus" = c(rep(0,3),1,rep(0,9)),

"G_Cing" = c(rep(0,4),1,rep(0,8)),

"Hippocampus" = c(rep(0,5),1,rep(0,7)),

"InferiorParietal" = c(rep(0,6),1,rep(0,6)),

"Insula" = c(rep(0,7),1,rep(0,5)),

"LingualGyrus" = c(rep(0,8),1,rep(0,4)),

"MiddleInfTempGyrus" = c(rep(0,9),1,rep(0,3)),

"Prefrontal" = c(rep(0,10),1,rep(0,2)),

"SuperiorParietal" = c(rep(0,11),1,rep(0,1)),

"SuperiorTemp" = c(rep(0,12),1))))

# Amyloid

TSPO_Amyloid_lm_full3<-lm(ER176 ~ FBB*roi + TSPO_Affinity + Age_baseline + Sex + BMI_baseline + APOE4,

analysis.dat.long %>% filter(roi %in% roilist))

TSPO_Amyloid_lm_full3_summary<-summary(TSPO_Amyloid_lm_full3)

TSPO_Amyloid_lm_full3_slopes <- emtrends(TSPO_Amyloid_lm_full3, ~ roi, var = "FBB")

TSPO_Amyloid_lm_full3_contrast <- contrast(TSPO_Amyloid_lm_full3_slopes,

method = list("Striatum" = c(1, rep(0,12)),

"Amygdala" = c(rep(0,1),1,rep(0,11)),

"EntorhinalCortex_FS" = c(rep(0,2),1,rep(0,10)),

"FusiformGyrus" = c(rep(0,3),1,rep(0,9)),

"G_Cing" = c(rep(0,4),1,rep(0,8)),

"Hippocampus" = c(rep(0,5),1,rep(0,7)),

"InferiorParietal" = c(rep(0,6),1,rep(0,6)),

"Insula" = c(rep(0,7),1,rep(0,5)),

"LingualGyrus" = c(rep(0,8),1,rep(0,4)),

"MiddleInfTempGyrus" = c(rep(0,9),1,rep(0,3)),

"Prefrontal" = c(rep(0,10),1,rep(0,2)),

"SuperiorParietal" = c(rep(0,11),1,rep(0,1)),

"SuperiorTemp" = c(rep(0,12),1)),

weights="proportional",adjust="mvt") %>%

summary(infer=TRUE)

# Amyloid std

TSPO_Amyloid_lm_full3_std_contrast <- as.data.frame(eff_size(emtrends(TSPO_Amyloid_lm_full3, ~roi, var = "FBB", weights="proportional",adjust="mvt"),

sigma = sigma(TSPO_Amyloid_lm_full3), edf = TSPO_Amyloid_lm_full3_summary[[7]][[2]], method=list("Striatum" = c(1, rep(0,12)),

"Amygdala" = c(rep(0,1),1,rep(0,11)),

"EntorhinalCortex_FS" = c(rep(0,2),1,rep(0,10)),

"FusiformGyrus" = c(rep(0,3),1,rep(0,9)),

"G_Cing" = c(rep(0,4),1,rep(0,8)),

"Hippocampus" = c(rep(0,5),1,rep(0,7)),

"InferiorParietal" = c(rep(0,6),1,rep(0,6)),

"Insula" = c(rep(0,7),1,rep(0,5)),

"LingualGyrus" = c(rep(0,8),1,rep(0,4)),

"MiddleInfTempGyrus" = c(rep(0,9),1,rep(0,3)),

"Prefrontal" = c(rep(0,10),1,rep(0,2)),

"SuperiorParietal" = c(rep(0,11),1,rep(0,1)),

"SuperiorTemp" = c(rep(0,12),1))))

# Volume

# adjust for icv

TSPO_Volume_lm_full3<-lm(ER176 ~ Volume_MK6240*roi + ICV_baseline + TSPO_Affinity + Age_baseline + Sex + BMI_baseline + APOE4,

analysis.dat.long %>% filter(roi %in% roilist))

TSPO_Volume_lm_full3_summary<-summary(TSPO_Volume_lm_full3)

TSPO_Volume_lm_full3_slopes <- emtrends(TSPO_Volume_lm_full3, ~ roi, var = "Volume_MK6240")

TSPO_Volume_lm_full3_contrast <- contrast(TSPO_Volume_lm_full3_slopes,

method = list("Striatum" = c(1, rep(0,12)),

"Amygdala" = c(rep(0,1),1,rep(0,11)),

"EntorhinalCortex_FS" = c(rep(0,2),1,rep(0,10)),

"FusiformGyrus" = c(rep(0,3),1,rep(0,9)),

"G_Cing" = c(rep(0,4),1,rep(0,8)),

"Hippocampus" = c(rep(0,5),1,rep(0,7)),

"InferiorParietal" = c(rep(0,6),1,rep(0,6)),

"Insula" = c(rep(0,7),1,rep(0,5)),

"LingualGyrus" = c(rep(0,8),1,rep(0,4)),

"MiddleInfTempGyrus" = c(rep(0,9),1,rep(0,3)),

"Prefrontal" = c(rep(0,10),1,rep(0,2)),

"SuperiorParietal" = c(rep(0,11),1,rep(0,1)),

"SuperiorTemp" = c(rep(0,12),1)),

weights="proportional",adjust="mvt") %>%

summary(infer=TRUE)

# Volume std

TSPO_Volume_lm_full3_std_contrast <- as.data.frame(eff_size(emtrends(TSPO_Volume_lm_full3, ~roi, var = "Volume_MK6240", weights="proportional",adjust="mvt"),

sigma = sigma(TSPO_Volume_lm_full3), edf = TSPO_Volume_lm_full3_summary[[7]][[2]], method=list("Striatum" = c(1, rep(0,12)),

"Amygdala" = c(rep(0,1),1,rep(0,11)),

"EntorhinalCortex_FS" = c(rep(0,2),1,rep(0,10)),

"FusiformGyrus" = c(rep(0,3),1,rep(0,9)),

"G_Cing" = c(rep(0,4),1,rep(0,8)),

"Hippocampus" = c(rep(0,5),1,rep(0,7)),

"InferiorParietal" = c(rep(0,6),1,rep(0,6)),

"Insula" = c(rep(0,7),1,rep(0,5)),

"LingualGyrus" = c(rep(0,8),1,rep(0,4)),

"MiddleInfTempGyrus" = c(rep(0,9),1,rep(0,3)),

"Prefrontal" = c(rep(0,10),1,rep(0,2)),

"SuperiorParietal" = c(rep(0,11),1,rep(0,1)),

"SuperiorTemp" = c(rep(0,12),1))))

## Sensitivity: brain-wide

dat.wide$wm_FBB_UNC_SUVR_baseline <- (dat.wide$FBB_lh_wm_UNC_SUVR_baseline + dat.wide$FBB_rh_wm_UNC_SUVR_baseline) / 2

dat.wide$cbmwm_FBB_UNC_SUVR_baseline <- (dat.wide$FBB_lh_cbmwm_UNC_SUVR_baseline + dat.wide$FBB_rh_cbmwm_UNC_SUVR_baseline) / 2

dat.wide$cbmgm_FBB_UNC_SUVR_baseline <- (dat.wide$FBB_lh_cbmgm_UNC_SUVR_baseline + dat.wide$FBB_rh_cbmgm_UNC_SUVR_baseline) / 2

dat.wide$thalamus_FBB_UNC_SUVR_baseline <- (dat.wide$FBB_lh_thalamus_UNC_SUVR_baseline + dat.wide$FBB_rh_thalamus_UNC_SUVR_baseline) / 2

dat.wide$caudate_FBB_UNC_SUVR_baseline <- (dat.wide$FBB_lh_caudate_UNC_SUVR_baseline + dat.wide$FBB_rh_caudate_UNC_SUVR_baseline) / 2

dat.wide$putamen_FBB_UNC_SUVR_baseline <- (dat.wide$FBB_lh_putamen_UNC_SUVR_baseline + dat.wide$FBB_rh_putamen_UNC_SUVR_baseline) / 2

dat.wide$pallidum_FBB_UNC_SUVR_baseline <- (dat.wide$FBB_lh_pallidum_UNC_SUVR_baseline + dat.wide$FBB_rh_pallidum_UNC_SUVR_baseline) / 2

dat.wide$hippocampus_FBB_UNC_SUVR_baseline <- (dat.wide$FBB_lh_hippocampus_UNC_SUVR_baseline + dat.wide$FBB_rh_hippocampus_UNC_SUVR_baseline) / 2

dat.wide$amygdala_FBB_UNC_SUVR_baseline <- (dat.wide$FBB_lh_amygdala_UNC_SUVR_baseline + dat.wide$FBB_rh_amygdala_UNC_SUVR_baseline) / 2

dat.wide$accumbens_FBB_UNC_SUVR_baseline <- (dat.wide$FBB_lh_accumbens_UNC_SUVR_baseline + dat.wide$FBB_rh_accumbens_UNC_SUVR_baseline) / 2

dat.wide$choroid_FBB_UNC_SUVR_baseline <- (dat.wide$FBB_lh_choroid_UNC_SUVR_baseline + dat.wide$FBB_rh_choroid_UNC_SUVR_baseline) / 2

dat.wide$bankssts_FBB_UNC_SUVR_baseline <- (dat.wide$FBB_lh_bankssts_UNC_SUVR_baseline + dat.wide$FBB_rh_bankssts_UNC_SUVR_baseline) / 2

dat.wide$caudalanteriorcingulate_FBB_UNC_SUVR_baseline <- (dat.wide$FBB_lh_caudalanteriorcingulate_UNC_SUVR_baseline + dat.wide$FBB_rh_caudalanteriorcingulate_UNC_SUVR_baseline) / 2

dat.wide$caudalmiddlefrontal_FBB_UNC_SUVR_baseline <- (dat.wide$FBB_lh_caudalmiddlefrontal_UNC_SUVR_baseline + dat.wide$FBB_rh_caudalmiddlefrontal_UNC_SUVR_baseline) / 2

dat.wide$cuneus_FBB_UNC_SUVR_baseline <- (dat.wide$FBB_lh_cuneus_UNC_SUVR_baseline + dat.wide$FBB_rh_cuneus_UNC_SUVR_baseline) / 2

dat.wide$entorhinal_FBB_UNC_SUVR_baseline <- (dat.wide$FBB_lh_entorhinal_UNC_SUVR_baseline + dat.wide$FBB_rh_entorhinal_UNC_SUVR_baseline) / 2

dat.wide$fusiform_FBB_UNC_SUVR_baseline <- (dat.wide$FBB_lh_fusiform_UNC_SUVR_baseline + dat.wide$FBB_rh_fusiform_UNC_SUVR_baseline) / 2

dat.wide$inferiorparietal_FBB_UNC_SUVR_baseline <- (dat.wide$FBB_lh_inferiorparietal_UNC_SUVR_baseline + dat.wide$FBB_rh_inferiorparietal_UNC_SUVR_baseline) / 2

dat.wide$inferiortemporal_FBB_UNC_SUVR_baseline <- (dat.wide$FBB_lh_inferiortemporal_UNC_SUVR_baseline + dat.wide$FBB_rh_inferiortemporal_UNC_SUVR_baseline) / 2

dat.wide$isthmuscingulate_FBB_UNC_SUVR_baseline <- (dat.wide$FBB_lh_isthmuscingulate_UNC_SUVR_baseline + dat.wide$FBB_rh_isthmuscingulate_UNC_SUVR_baseline) / 2

dat.wide$lateraloccipital_FBB_UNC_SUVR_baseline <- (dat.wide$FBB_lh_lateraloccipital_UNC_SUVR_baseline + dat.wide$FBB_rh_lateraloccipital_UNC_SUVR_baseline) / 2

dat.wide$lateralorbitofrontal_FBB_UNC_SUVR_baseline <- (dat.wide$FBB_lh_lateralorbitofrontal_UNC_SUVR_baseline + dat.wide$FBB_rh_lateralorbitofrontal_UNC_SUVR_baseline) / 2

dat.wide$lingual_FBB_UNC_SUVR_baseline <- (dat.wide$FBB_lh_lingual_UNC_SUVR_baseline + dat.wide$FBB_rh_lingual_UNC_SUVR_baseline) / 2

dat.wide$medialorbitofrontal_FBB_UNC_SUVR_baseline <- (dat.wide$FBB_lh_medialorbitofrontal_UNC_SUVR_baseline + dat.wide$FBB_rh_medialorbitofrontal_UNC_SUVR_baseline) / 2

dat.wide$lingual_FBB_UNC_SUVR_baseline <- (dat.wide$FBB_lh_lingual_UNC_SUVR_baseline + dat.wide$FBB_rh_lingual_UNC_SUVR_baseline) / 2

dat.wide$medialorbitofrontal_FBB_UNC_SUVR_baseline <- (dat.wide$FBB_lh_medialorbitofrontal_UNC_SUVR_baseline + dat.wide$FBB_rh_medialorbitofrontal_UNC_SUVR_baseline) / 2

dat.wide$middletemporal_FBB_UNC_SUVR_baseline <- (dat.wide$FBB_lh_middletemporal_UNC_SUVR_baseline + dat.wide$FBB_rh_middletemporal_UNC_SUVR_baseline) / 2

dat.wide$parahippocampal_FBB_UNC_SUVR_baseline <- (dat.wide$FBB_lh_parahippocampal_UNC_SUVR_baseline + dat.wide$FBB_rh_parahippocampal_UNC_SUVR_baseline) / 2

dat.wide$paracentral_FBB_UNC_SUVR_baseline <- (dat.wide$FBB_lh_paracentral_UNC_SUVR_baseline + dat.wide$FBB_rh_paracentral_UNC_SUVR_baseline) / 2

dat.wide$parsopercularis_FBB_UNC_SUVR_baseline <- (dat.wide$FBB_lh_parsopercularis_UNC_SUVR_baseline + dat.wide$FBB_rh_parsopercularis_UNC_SUVR_baseline) / 2

dat.wide$parsorbitalis_FBB_UNC_SUVR_baseline <- (dat.wide$FBB_lh_parsorbitalis_UNC_SUVR_baseline + dat.wide$FBB_rh_parsorbitalis_UNC_SUVR_baseline) / 2

dat.wide$parstriangularis_FBB_UNC_SUVR_baseline <- (dat.wide$FBB_lh_parstriangularis_UNC_SUVR_baseline + dat.wide$FBB_rh_parstriangularis_UNC_SUVR_baseline) / 2

dat.wide$pericalcarine_FBB_UNC_SUVR_baseline <- (dat.wide$FBB_lh_pericalcarine_UNC_SUVR_baseline + dat.wide$FBB_rh_pericalcarine_UNC_SUVR_baseline) / 2

dat.wide$postcentral_FBB_UNC_SUVR_baseline <- (dat.wide$FBB_lh_postcentral_UNC_SUVR_baseline + dat.wide$FBB_rh_postcentral_UNC_SUVR_baseline) / 2

dat.wide$posteriorcingulate_FBB_UNC_SUVR_baseline <- (dat.wide$FBB_lh_posteriorcingulate_UNC_SUVR_baseline + dat.wide$FBB_rh_posteriorcingulate_UNC_SUVR_baseline) / 2

dat.wide$precentral_FBB_UNC_SUVR_baseline <- (dat.wide$FBB_lh_precentral_UNC_SUVR_baseline + dat.wide$FBB_rh_precentral_UNC_SUVR_baseline) / 2

dat.wide$precuneus_FBB_UNC_SUVR_baseline <- (dat.wide$FBB_lh_precuneus_UNC_SUVR_baseline + dat.wide$FBB_rh_precuneus_UNC_SUVR_baseline) / 2

dat.wide$rostralanteriorcingulate_FBB_UNC_SUVR_baseline <- (dat.wide$FBB_lh_rostralanteriorcingulate_UNC_SUVR_baseline + dat.wide$FBB_rh_rostralanteriorcingulate_UNC_SUVR_baseline) / 2

dat.wide$rostralmiddlefrontal_FBB_UNC_SUVR_baseline <- (dat.wide$FBB_lh_rostralmiddlefrontal_UNC_SUVR_baseline + dat.wide$FBB_rh_rostralmiddlefrontal_UNC_SUVR_baseline) / 2

dat.wide$superiorfrontal_FBB_UNC_SUVR_baseline <- (dat.wide$FBB_lh_superiorfrontal_UNC_SUVR_baseline + dat.wide$FBB_rh_superiorfrontal_UNC_SUVR_baseline) / 2

dat.wide$superiorparietal_FBB_UNC_SUVR_baseline <- (dat.wide$FBB_lh_superiorparietal_UNC_SUVR_baseline + dat.wide$FBB_rh_superiorparietal_UNC_SUVR_baseline) / 2

dat.wide$superiortemporal_FBB_UNC_SUVR_baseline <- (dat.wide$FBB_lh_superiortemporal_UNC_SUVR_baseline + dat.wide$FBB_rh_superiortemporal_UNC_SUVR_baseline) / 2

dat.wide$supramarginal_FBB_UNC_SUVR_baseline <- (dat.wide$FBB_lh_supramarginal_UNC_SUVR_baseline + dat.wide$FBB_rh_supramarginal_UNC_SUVR_baseline) / 2

dat.wide$frontalpole_FBB_UNC_SUVR_baseline <- (dat.wide$FBB_lh_frontalpole_UNC_SUVR_baseline + dat.wide$FBB_rh_frontalpole_UNC_SUVR_baseline) / 2

dat.wide$temporalpole_FBB_UNC_SUVR_baseline <- (dat.wide$FBB_lh_temporalpole_UNC_SUVR_baseline + dat.wide$FBB_rh_temporalpole_UNC_SUVR_baseline) / 2

dat.wide$transversetemporal_FBB_UNC_SUVR_baseline <- (dat.wide$FBB_lh_transversetemporal_UNC_SUVR_baseline + dat.wide$FBB_rh_transversetemporal_UNC_SUVR_baseline) / 2

dat.wide$insula_FBB_UNC_SUVR_baseline <- (dat.wide$FBB_lh_insula_UNC_SUVR_baseline + dat.wide$FBB_rh_insula_UNC_SUVR_baseline) / 2

dat.wide$brainstem_FBB_UNC_SUVR_baseline <- dat.wide$FBB_brainstem_UNC_SUVR_baseline

dat.wide$cc_posterior_FBB_UNC_SUVR_baseline <- dat.wide$FBB_cc_posterior_UNC_SUVR_baseline

dat.wide$cc_mid_posterior_FBB_UNC_SUVR_baseline <- dat.wide$FBB_cc_mid_posterior_UNC_SUVR_baseline

dat.wide$cc_central_FBB_UNC_SUVR_baseline <- dat.wide$FBB_cc_central_UNC_SUVR_baseline

dat.wide$cc_mid_anterior_FBB_UNC_SUVR_baseline <- dat.wide$FBB_cc_mid_anterior_UNC_SUVR_baseline

dat.wide$cc_anterior_FBB_UNC_SUVR_baseline <- dat.wide$FBB_cc_anterior_UNC_SUVR_baseline

dat.wide$wm_MK6240_PVC_SUVR_baseline <- (dat.wide$MK6240_lh_wm_PVC_SUVR_baseline + dat.wide$MK6240_rh_wm_PVC_SUVR_baseline) / 2

dat.wide$cbmwm_MK6240_PVC_SUVR_baseline <- (dat.wide$MK6240_lh_cbmwm_PVC_SUVR_baseline + dat.wide$MK6240_rh_cbmwm_PVC_SUVR_baseline) / 2

dat.wide$cbmgm_MK6240_PVC_SUVR_baseline <- (dat.wide$MK6240_lh_cbmgm_PVC_SUVR_baseline + dat.wide$MK6240_rh_cbmgm_PVC_SUVR_baseline) / 2

dat.wide$thalamus_MK6240_PVC_SUVR_baseline <- (dat.wide$MK6240_lh_thalamus_PVC_SUVR_baseline + dat.wide$MK6240_rh_thalamus_PVC_SUVR_baseline) / 2

dat.wide$caudate_MK6240_PVC_SUVR_baseline <- (dat.wide$MK6240_lh_caudate_PVC_SUVR_baseline + dat.wide$MK6240_rh_caudate_PVC_SUVR_baseline) / 2

dat.wide$putamen_MK6240_PVC_SUVR_baseline <- (dat.wide$MK6240_lh_putamen_PVC_SUVR_baseline + dat.wide$MK6240_rh_putamen_PVC_SUVR_baseline) / 2

dat.wide$pallidum_MK6240_PVC_SUVR_baseline <- (dat.wide$MK6240_lh_pallidum_PVC_SUVR_baseline + dat.wide$MK6240_rh_pallidum_PVC_SUVR_baseline) / 2

dat.wide$hippocampus_MK6240_PVC_SUVR_baseline <- (dat.wide$MK6240_lh_hippocampus_PVC_SUVR_baseline + dat.wide$MK6240_rh_hippocampus_PVC_SUVR_baseline) / 2

dat.wide$amygdala_MK6240_PVC_SUVR_baseline <- (dat.wide$MK6240_lh_amygdala_PVC_SUVR_baseline + dat.wide$MK6240_rh_amygdala_PVC_SUVR_baseline) / 2

dat.wide$accumbens_MK6240_PVC_SUVR_baseline <- (dat.wide$MK6240_lh_accumbens_PVC_SUVR_baseline + dat.wide$MK6240_rh_accumbens_PVC_SUVR_baseline) / 2

dat.wide$choroid_MK6240_PVC_SUVR_baseline <- (dat.wide$MK6240_lh_choroid_PVC_SUVR_baseline + dat.wide$MK6240_rh_choroid_PVC_SUVR_baseline) / 2

dat.wide$bankssts_MK6240_PVC_SUVR_baseline <- (dat.wide$MK6240_lh_bankssts_PVC_SUVR_baseline + dat.wide$MK6240_rh_bankssts_PVC_SUVR_baseline) / 2

dat.wide$caudalanteriorcingulate_MK6240_PVC_SUVR_baseline <- (dat.wide$MK6240_lh_caudalanteriorcingulate_PVC_SUVR_baseline + dat.wide$MK6240_rh_caudalanteriorcingulate_PVC_SUVR_baseline) / 2

dat.wide$caudalmiddlefrontal_MK6240_PVC_SUVR_baseline <- (dat.wide$MK6240_lh_caudalmiddlefrontal_PVC_SUVR_baseline + dat.wide$MK6240_rh_caudalmiddlefrontal_PVC_SUVR_baseline) / 2

dat.wide$cuneus_MK6240_PVC_SUVR_baseline <- (dat.wide$MK6240_lh_cuneus_PVC_SUVR_baseline + dat.wide$MK6240_rh_cuneus_PVC_SUVR_baseline) / 2

dat.wide$entorhinal_MK6240_PVC_SUVR_baseline <- (dat.wide$MK6240_lh_entorhinal_PVC_SUVR_baseline + dat.wide$MK6240_rh_entorhinal_PVC_SUVR_baseline) / 2

dat.wide$fusiform_MK6240_PVC_SUVR_baseline <- (dat.wide$MK6240_lh_fusiform_PVC_SUVR_baseline + dat.wide$MK6240_rh_fusiform_PVC_SUVR_baseline) / 2

dat.wide$inferiorparietal_MK6240_PVC_SUVR_baseline <- (dat.wide$MK6240_lh_inferiorparietal_PVC_SUVR_baseline + dat.wide$MK6240_rh_inferiorparietal_PVC_SUVR_baseline) / 2

dat.wide$inferiortemporal_MK6240_PVC_SUVR_baseline <- (dat.wide$MK6240_lh_inferiortemporal_PVC_SUVR_baseline + dat.wide$MK6240_rh_inferiortemporal_PVC_SUVR_baseline) / 2

dat.wide$isthmuscingulate_MK6240_PVC_SUVR_baseline <- (dat.wide$MK6240_lh_isthmuscingulate_PVC_SUVR_baseline + dat.wide$MK6240_rh_isthmuscingulate_PVC_SUVR_baseline) / 2

dat.wide$lateraloccipital_MK6240_PVC_SUVR_baseline <- (dat.wide$MK6240_lh_lateraloccipital_PVC_SUVR_baseline + dat.wide$MK6240_rh_lateraloccipital_PVC_SUVR_baseline) / 2

dat.wide$lateralorbitofrontal_MK6240_PVC_SUVR_baseline <- (dat.wide$MK6240_lh_lateralorbitofrontal_PVC_SUVR_baseline + dat.wide$MK6240_rh_lateralorbitofrontal_PVC_SUVR_baseline) / 2

dat.wide$lingual_MK6240_PVC_SUVR_baseline <- (dat.wide$MK6240_lh_lingual_PVC_SUVR_baseline + dat.wide$MK6240_rh_lingual_PVC_SUVR_baseline) / 2

dat.wide$medialorbitofrontal_MK6240_PVC_SUVR_baseline <- (dat.wide$MK6240_lh_medialorbitofrontal_PVC_SUVR_baseline + dat.wide$MK6240_rh_medialorbitofrontal_PVC_SUVR_baseline) / 2

dat.wide$lingual_MK6240_PVC_SUVR_baseline <- (dat.wide$MK6240_lh_lingual_PVC_SUVR_baseline + dat.wide$MK6240_rh_lingual_PVC_SUVR_baseline) / 2

dat.wide$medialorbitofrontal_MK6240_PVC_SUVR_baseline <- (dat.wide$MK6240_lh_medialorbitofrontal_PVC_SUVR_baseline + dat.wide$MK6240_rh_medialorbitofrontal_PVC_SUVR_baseline) / 2

dat.wide$middletemporal_MK6240_PVC_SUVR_baseline <- (dat.wide$MK6240_lh_middletemporal_PVC_SUVR_baseline + dat.wide$MK6240_rh_middletemporal_PVC_SUVR_baseline) / 2

dat.wide$parahippocampal_MK6240_PVC_SUVR_baseline <- (dat.wide$MK6240_lh_parahippocampal_PVC_SUVR_baseline + dat.wide$MK6240_rh_parahippocampal_PVC_SUVR_baseline) / 2

dat.wide$paracentral_MK6240_PVC_SUVR_baseline <- (dat.wide$MK6240_lh_paracentral_PVC_SUVR_baseline + dat.wide$MK6240_rh_paracentral_PVC_SUVR_baseline) / 2

dat.wide$parsopercularis_MK6240_PVC_SUVR_baseline <- (dat.wide$MK6240_lh_parsopercularis_PVC_SUVR_baseline + dat.wide$MK6240_rh_parsopercularis_PVC_SUVR_baseline) / 2

dat.wide$parsorbitalis_MK6240_PVC_SUVR_baseline <- (dat.wide$MK6240_lh_parsorbitalis_PVC_SUVR_baseline + dat.wide$MK6240_rh_parsorbitalis_PVC_SUVR_baseline) / 2

dat.wide$parstriangularis_MK6240_PVC_SUVR_baseline <- (dat.wide$MK6240_lh_parstriangularis_PVC_SUVR_baseline + dat.wide$MK6240_rh_parstriangularis_PVC_SUVR_baseline) / 2

dat.wide$pericalcarine_MK6240_PVC_SUVR_baseline <- (dat.wide$MK6240_lh_pericalcarine_PVC_SUVR_baseline + dat.wide$MK6240_rh_pericalcarine_PVC_SUVR_baseline) / 2

dat.wide$postcentral_MK6240_PVC_SUVR_baseline <- (dat.wide$MK6240_lh_postcentral_PVC_SUVR_baseline + dat.wide$MK6240_rh_postcentral_PVC_SUVR_baseline) / 2

dat.wide$posteriorcingulate_MK6240_PVC_SUVR_baseline <- (dat.wide$MK6240_lh_posteriorcingulate_PVC_SUVR_baseline + dat.wide$MK6240_rh_posteriorcingulate_PVC_SUVR_baseline) / 2

dat.wide$precentral_MK6240_PVC_SUVR_baseline <- (dat.wide$MK6240_lh_precentral_PVC_SUVR_baseline + dat.wide$MK6240_rh_precentral_PVC_SUVR_baseline) / 2

dat.wide$precuneus_MK6240_PVC_SUVR_baseline <- (dat.wide$MK6240_lh_precuneus_PVC_SUVR_baseline + dat.wide$MK6240_rh_precuneus_PVC_SUVR_baseline) / 2

dat.wide$rostralanteriorcingulate_MK6240_PVC_SUVR_baseline <- (dat.wide$MK6240_lh_rostralanteriorcingulate_PVC_SUVR_baseline + dat.wide$MK6240_rh_rostralanteriorcingulate_PVC_SUVR_baseline) / 2

dat.wide$rostralmiddlefrontal_MK6240_PVC_SUVR_baseline <- (dat.wide$MK6240_lh_rostralmiddlefrontal_PVC_SUVR_baseline + dat.wide$MK6240_rh_rostralmiddlefrontal_PVC_SUVR_baseline) / 2

dat.wide$superiorfrontal_MK6240_PVC_SUVR_baseline <- (dat.wide$MK6240_lh_superiorfrontal_PVC_SUVR_baseline + dat.wide$MK6240_rh_superiorfrontal_PVC_SUVR_baseline) / 2

dat.wide$superiorparietal_MK6240_PVC_SUVR_baseline <- (dat.wide$MK6240_lh_superiorparietal_PVC_SUVR_baseline + dat.wide$MK6240_rh_superiorparietal_PVC_SUVR_baseline) / 2

dat.wide$superiortemporal_MK6240_PVC_SUVR_baseline <- (dat.wide$MK6240_lh_superiortemporal_PVC_SUVR_baseline + dat.wide$MK6240_rh_superiortemporal_PVC_SUVR_baseline) / 2

dat.wide$supramarginal_MK6240_PVC_SUVR_baseline <- (dat.wide$MK6240_lh_supramarginal_PVC_SUVR_baseline + dat.wide$MK6240_rh_supramarginal_PVC_SUVR_baseline) / 2

dat.wide$frontalpole_MK6240_PVC_SUVR_baseline <- (dat.wide$MK6240_lh_frontalpole_PVC_SUVR_baseline + dat.wide$MK6240_rh_frontalpole_PVC_SUVR_baseline) / 2

dat.wide$temporalpole_MK6240_PVC_SUVR_baseline <- (dat.wide$MK6240_lh_temporalpole_PVC_SUVR_baseline + dat.wide$MK6240_rh_temporalpole_PVC_SUVR_baseline) / 2

dat.wide$transversetemporal_MK6240_PVC_SUVR_baseline <- (dat.wide$MK6240_lh_transversetemporal_PVC_SUVR_baseline + dat.wide$MK6240_rh_transversetemporal_PVC_SUVR_baseline) / 2

dat.wide$insula_MK6240_PVC_SUVR_baseline <- (dat.wide$MK6240_lh_insula_PVC_SUVR_baseline + dat.wide$MK6240_rh_insula_PVC_SUVR_baseline) / 2

dat.wide$brainstem_MK6240_PVC_SUVR_baseline <- dat.wide$MK6240_brainstem_PVC_SUVR_baseline

dat.wide$cc_posterior_MK6240_PVC_SUVR_baseline <- dat.wide$MK6240_cc_posterior_PVC_SUVR_baseline

dat.wide$cc_mid_posterior_MK6240_PVC_SUVR_baseline <- dat.wide$MK6240_cc_mid_posterior_PVC_SUVR_baseline

dat.wide$cc_central_MK6240_PVC_SUVR_baseline <- dat.wide$MK6240_cc_central_PVC_SUVR_baseline

dat.wide$cc_mid_anterior_MK6240_PVC_SUVR_baseline <- dat.wide$MK6240_cc_mid_anterior_PVC_SUVR_baseline

dat.wide$cc_anterior_MK6240_PVC_SUVR_baseline <- dat.wide$MK6240_cc_anterior_PVC_SUVR_baseline

dat.wide$wm_ER176_PVC_SUVR_baseline <- (dat.wide$ER176_lh_wm_PVC_SUVR_baseline + dat.wide$ER176_rh_wm_PVC_SUVR_baseline) / 2

dat.wide$cbmwm_ER176_PVC_SUVR_baseline <- (dat.wide$ER176_lh_cbmwm_PVC_SUVR_baseline + dat.wide$ER176_rh_cbmwm_PVC_SUVR_baseline) / 2

dat.wide$cbmgm_ER176_PVC_SUVR_baseline <- (dat.wide$ER176_lh_cbmgm_PVC_SUVR_baseline + dat.wide$ER176_rh_cbmgm_PVC_SUVR_baseline) / 2

dat.wide$thalamus_ER176_PVC_SUVR_baseline <- (dat.wide$ER176_lh_thalamus_PVC_SUVR_baseline + dat.wide$ER176_rh_thalamus_PVC_SUVR_baseline) / 2

dat.wide$caudate_ER176_PVC_SUVR_baseline <- (dat.wide$ER176_lh_caudate_PVC_SUVR_baseline + dat.wide$ER176_rh_caudate_PVC_SUVR_baseline) / 2

dat.wide$putamen_ER176_PVC_SUVR_baseline <- (dat.wide$ER176_lh_putamen_PVC_SUVR_baseline + dat.wide$ER176_rh_putamen_PVC_SUVR_baseline) / 2

dat.wide$pallidum_ER176_PVC_SUVR_baseline <- (dat.wide$ER176_lh_pallidum_PVC_SUVR_baseline + dat.wide$ER176_rh_pallidum_PVC_SUVR_baseline) / 2

dat.wide$hippocampus_ER176_PVC_SUVR_baseline <- (dat.wide$ER176_lh_hippocampus_PVC_SUVR_baseline + dat.wide$ER176_rh_hippocampus_PVC_SUVR_baseline) / 2

dat.wide$amygdala_ER176_PVC_SUVR_baseline <- (dat.wide$ER176_lh_amygdala_PVC_SUVR_baseline + dat.wide$ER176_rh_amygdala_PVC_SUVR_baseline) / 2

dat.wide$accumbens_ER176_PVC_SUVR_baseline <- (dat.wide$ER176_lh_accumbens_PVC_SUVR_baseline + dat.wide$ER176_rh_accumbens_PVC_SUVR_baseline) / 2

dat.wide$choroid_ER176_PVC_SUVR_baseline <- (dat.wide$ER176_lh_choroid_PVC_SUVR_baseline + dat.wide$ER176_rh_choroid_PVC_SUVR_baseline) / 2

dat.wide$bankssts_ER176_PVC_SUVR_baseline <- (dat.wide$ER176_lh_bankssts_PVC_SUVR_baseline + dat.wide$ER176_rh_bankssts_PVC_SUVR_baseline) / 2

dat.wide$caudalanteriorcingulate_ER176_PVC_SUVR_baseline <- (dat.wide$ER176_lh_caudalanteriorcingulate_PVC_SUVR_baseline + dat.wide$ER176_rh_caudalanteriorcingulate_PVC_SUVR_baseline) / 2

dat.wide$caudalmiddlefrontal_ER176_PVC_SUVR_baseline <- (dat.wide$ER176_lh_caudalmiddlefrontal_PVC_SUVR_baseline + dat.wide$ER176_rh_caudalmiddlefrontal_PVC_SUVR_baseline) / 2

dat.wide$cuneus_ER176_PVC_SUVR_baseline <- (dat.wide$ER176_lh_cuneus_PVC_SUVR_baseline + dat.wide$ER176_rh_cuneus_PVC_SUVR_baseline) / 2

dat.wide$entorhinal_ER176_PVC_SUVR_baseline <- (dat.wide$ER176_lh_entorhinal_PVC_SUVR_baseline + dat.wide$ER176_rh_entorhinal_PVC_SUVR_baseline) / 2

dat.wide$fusiform_ER176_PVC_SUVR_baseline <- (dat.wide$ER176_lh_fusiform_PVC_SUVR_baseline + dat.wide$ER176_rh_fusiform_PVC_SUVR_baseline) / 2

dat.wide$inferiorparietal_ER176_PVC_SUVR_baseline <- (dat.wide$ER176_lh_inferiorparietal_PVC_SUVR_baseline + dat.wide$ER176_rh_inferiorparietal_PVC_SUVR_baseline) / 2

dat.wide$inferiortemporal_ER176_PVC_SUVR_baseline <- (dat.wide$ER176_lh_inferiortemporal_PVC_SUVR_baseline + dat.wide$ER176_rh_inferiortemporal_PVC_SUVR_baseline) / 2

dat.wide$isthmuscingulate_ER176_PVC_SUVR_baseline <- (dat.wide$ER176_lh_isthmuscingulate_PVC_SUVR_baseline + dat.wide$ER176_rh_isthmuscingulate_PVC_SUVR_baseline) / 2

dat.wide$lateraloccipital_ER176_PVC_SUVR_baseline <- (dat.wide$ER176_lh_lateraloccipital_PVC_SUVR_baseline + dat.wide$ER176_rh_lateraloccipital_PVC_SUVR_baseline) / 2

dat.wide$lateralorbitofrontal_ER176_PVC_SUVR_baseline <- (dat.wide$ER176_lh_lateralorbitofrontal_PVC_SUVR_baseline + dat.wide$ER176_rh_lateralorbitofrontal_PVC_SUVR_baseline) / 2

dat.wide$lingual_ER176_PVC_SUVR_baseline <- (dat.wide$ER176_lh_lingual_PVC_SUVR_baseline + dat.wide$ER176_rh_lingual_PVC_SUVR_baseline) / 2

dat.wide$medialorbitofrontal_ER176_PVC_SUVR_baseline <- (dat.wide$ER176_lh_medialorbitofrontal_PVC_SUVR_baseline + dat.wide$ER176_rh_medialorbitofrontal_PVC_SUVR_baseline) / 2

dat.wide$lingual_ER176_PVC_SUVR_baseline <- (dat.wide$ER176_lh_lingual_PVC_SUVR_baseline + dat.wide$ER176_rh_lingual_PVC_SUVR_baseline) / 2

dat.wide$medialorbitofrontal_ER176_PVC_SUVR_baseline <- (dat.wide$ER176_lh_medialorbitofrontal_PVC_SUVR_baseline + dat.wide$ER176_rh_medialorbitofrontal_PVC_SUVR_baseline) / 2

dat.wide$middletemporal_ER176_PVC_SUVR_baseline <- (dat.wide$ER176_lh_middletemporal_PVC_SUVR_baseline + dat.wide$ER176_rh_middletemporal_PVC_SUVR_baseline) / 2

dat.wide$parahippocampal_ER176_PVC_SUVR_baseline <- (dat.wide$ER176_lh_parahippocampal_PVC_SUVR_baseline + dat.wide$ER176_rh_parahippocampal_PVC_SUVR_baseline) / 2

dat.wide$paracentral_ER176_PVC_SUVR_baseline <- (dat.wide$ER176_lh_paracentral_PVC_SUVR_baseline + dat.wide$ER176_rh_paracentral_PVC_SUVR_baseline) / 2

dat.wide$parsopercularis_ER176_PVC_SUVR_baseline <- (dat.wide$ER176_lh_parsopercularis_PVC_SUVR_baseline + dat.wide$ER176_rh_parsopercularis_PVC_SUVR_baseline) / 2

dat.wide$parsorbitalis_ER176_PVC_SUVR_baseline <- (dat.wide$ER176_lh_parsorbitalis_PVC_SUVR_baseline + dat.wide$ER176_rh_parsorbitalis_PVC_SUVR_baseline) / 2

dat.wide$parstriangularis_ER176_PVC_SUVR_baseline <- (dat.wide$ER176_lh_parstriangularis_PVC_SUVR_baseline + dat.wide$ER176_rh_parstriangularis_PVC_SUVR_baseline) / 2

dat.wide$pericalcarine_ER176_PVC_SUVR_baseline <- (dat.wide$ER176_lh_pericalcarine_PVC_SUVR_baseline + dat.wide$ER176_rh_pericalcarine_PVC_SUVR_baseline) / 2

dat.wide$postcentral_ER176_PVC_SUVR_baseline <- (dat.wide$ER176_lh_postcentral_PVC_SUVR_baseline + dat.wide$ER176_rh_postcentral_PVC_SUVR_baseline) / 2

dat.wide$posteriorcingulate_ER176_PVC_SUVR_baseline <- (dat.wide$ER176_lh_posteriorcingulate_PVC_SUVR_baseline + dat.wide$ER176_rh_posteriorcingulate_PVC_SUVR_baseline) / 2

dat.wide$precentral_ER176_PVC_SUVR_baseline <- (dat.wide$ER176_lh_precentral_PVC_SUVR_baseline + dat.wide$ER176_rh_precentral_PVC_SUVR_baseline) / 2

dat.wide$precuneus_ER176_PVC_SUVR_baseline <- (dat.wide$ER176_lh_precuneus_PVC_SUVR_baseline + dat.wide$ER176_rh_precuneus_PVC_SUVR_baseline) / 2

dat.wide$rostralanteriorcingulate_ER176_PVC_SUVR_baseline <- (dat.wide$ER176_lh_rostralanteriorcingulate_PVC_SUVR_baseline + dat.wide$ER176_rh_rostralanteriorcingulate_PVC_SUVR_baseline) / 2

dat.wide$rostralmiddlefrontal_ER176_PVC_SUVR_baseline <- (dat.wide$ER176_lh_rostralmiddlefrontal_PVC_SUVR_baseline + dat.wide$ER176_rh_rostralmiddlefrontal_PVC_SUVR_baseline) / 2

dat.wide$superiorfrontal_ER176_PVC_SUVR_baseline <- (dat.wide$ER176_lh_superiorfrontal_PVC_SUVR_baseline + dat.wide$ER176_rh_superiorfrontal_PVC_SUVR_baseline) / 2

dat.wide$superiorparietal_ER176_PVC_SUVR_baseline <- (dat.wide$ER176_lh_superiorparietal_PVC_SUVR_baseline + dat.wide$ER176_rh_superiorparietal_PVC_SUVR_baseline) / 2

dat.wide$superiortemporal_ER176_PVC_SUVR_baseline <- (dat.wide$ER176_lh_superiortemporal_PVC_SUVR_baseline + dat.wide$ER176_rh_superiortemporal_PVC_SUVR_baseline) / 2

dat.wide$supramarginal_ER176_PVC_SUVR_baseline <- (dat.wide$ER176_lh_supramarginal_PVC_SUVR_baseline + dat.wide$ER176_rh_supramarginal_PVC_SUVR_baseline) / 2

dat.wide$frontalpole_ER176_PVC_SUVR_baseline <- (dat.wide$ER176_lh_frontalpole_PVC_SUVR_baseline + dat.wide$ER176_rh_frontalpole_PVC_SUVR_baseline) / 2

dat.wide$temporalpole_ER176_PVC_SUVR_baseline <- (dat.wide$ER176_lh_temporalpole_PVC_SUVR_baseline + dat.wide$ER176_rh_temporalpole_PVC_SUVR_baseline) / 2

dat.wide$transversetemporal_ER176_PVC_SUVR_baseline <- (dat.wide$ER176_lh_transversetemporal_PVC_SUVR_baseline + dat.wide$ER176_rh_transversetemporal_PVC_SUVR_baseline) / 2

dat.wide$insula_ER176_PVC_SUVR_baseline <- (dat.wide$ER176_lh_insula_PVC_SUVR_baseline + dat.wide$ER176_rh_insula_PVC_SUVR_baseline) / 2

dat.wide$brainstem_ER176_PVC_SUVR_baseline <- dat.wide$ER176_brainstem_PVC_SUVR_baseline

dat.wide$cc_posterior_ER176_PVC_SUVR_baseline <- dat.wide$ER176_cc_posterior_PVC_SUVR_baseline

dat.wide$cc_mid_posterior_ER176_PVC_SUVR_baseline <- dat.wide$ER176_cc_mid_posterior_PVC_SUVR_baseline

dat.wide$cc_central_ER176_PVC_SUVR_baseline <- dat.wide$ER176_cc_central_PVC_SUVR_baseline

dat.wide$cc_mid_anterior_ER176_PVC_SUVR_baseline <- dat.wide$ER176_cc_mid_anterior_PVC_SUVR_baseline

dat.wide$cc_anterior_ER176_PVC_SUVR_baseline <- dat.wide$ER176_cc_anterior_PVC_SUVR_baseline

dat.wide$wm_Volume_FBB_UNC_baseline <- (dat.wide$FBB_lh_wm_UNC_vol_baseline + dat.wide$FBB_rh_wm_UNC_vol_baseline)

dat.wide$cbmwm_Volume_FBB_UNC_baseline <- (dat.wide$FBB_lh_cbmwm_UNC_vol_baseline + dat.wide$FBB_rh_cbmwm_UNC_vol_baseline)

dat.wide$cbmgm_Volume_FBB_UNC_baseline <- (dat.wide$FBB_lh_cbmgm_UNC_vol_baseline + dat.wide$FBB_rh_cbmgm_UNC_vol_baseline)

dat.wide$thalamus_Volume_FBB_UNC_baseline <- (dat.wide$FBB_lh_thalamus_UNC_vol_baseline + dat.wide$FBB_rh_thalamus_UNC_vol_baseline)

dat.wide$caudate_Volume_FBB_UNC_baseline <- (dat.wide$FBB_lh_caudate_UNC_vol_baseline + dat.wide$FBB_rh_caudate_UNC_vol_baseline)

dat.wide$putamen_Volume_FBB_UNC_baseline <- (dat.wide$FBB_lh_putamen_UNC_vol_baseline + dat.wide$FBB_rh_putamen_UNC_vol_baseline)

dat.wide$pallidum_Volume_FBB_UNC_baseline <- (dat.wide$FBB_lh_pallidum_UNC_vol_baseline + dat.wide$FBB_rh_pallidum_UNC_vol_baseline)

dat.wide$hippocampus_Volume_FBB_UNC_baseline <- (dat.wide$FBB_lh_hippocampus_UNC_vol_baseline + dat.wide$FBB_rh_hippocampus_UNC_vol_baseline)

dat.wide$amygdala_Volume_FBB_UNC_baseline <- (dat.wide$FBB_lh_amygdala_UNC_vol_baseline + dat.wide$FBB_rh_amygdala_UNC_vol_baseline)

dat.wide$accumbens_Volume_FBB_UNC_baseline <- (dat.wide$FBB_lh_accumbens_UNC_vol_baseline + dat.wide$FBB_rh_accumbens_UNC_vol_baseline)

dat.wide$choroid_Volume_FBB_UNC_baseline <- (dat.wide$FBB_lh_choroid_UNC_vol_baseline + dat.wide$FBB_rh_choroid_UNC_vol_baseline)

dat.wide$bankssts_Volume_FBB_UNC_baseline <- (dat.wide$FBB_lh_bankssts_UNC_vol_baseline + dat.wide$FBB_rh_bankssts_UNC_vol_baseline)

dat.wide$caudalanteriorcingulate_Volume_FBB_UNC_baseline <- (dat.wide$FBB_lh_caudalanteriorcingulate_UNC_vol_baseline + dat.wide$FBB_rh_caudalanteriorcingulate_UNC_vol_baseline)

dat.wide$caudalmiddlefrontal_Volume_FBB_UNC_baseline <- (dat.wide$FBB_lh_caudalmiddlefrontal_UNC_vol_baseline + dat.wide$FBB_rh_caudalmiddlefrontal_UNC_vol_baseline)

dat.wide$cuneus_Volume_FBB_UNC_baseline <- (dat.wide$FBB_lh_cuneus_UNC_vol_baseline + dat.wide$FBB_rh_cuneus_UNC_vol_baseline)

dat.wide$entorhinal_Volume_FBB_UNC_baseline <- (dat.wide$FBB_lh_entorhinal_UNC_vol_baseline + dat.wide$FBB_rh_entorhinal_UNC_vol_baseline)

dat.wide$fusiform_Volume_FBB_UNC_baseline <- (dat.wide$FBB_lh_fusiform_UNC_vol_baseline + dat.wide$FBB_rh_fusiform_UNC_vol_baseline)

dat.wide$inferiorparietal_Volume_FBB_UNC_baseline <- (dat.wide$FBB_lh_inferiorparietal_UNC_vol_baseline + dat.wide$FBB_rh_inferiorparietal_UNC_vol_baseline)

dat.wide$inferiortemporal_Volume_FBB_UNC_baseline <- (dat.wide$FBB_lh_inferiortemporal_UNC_vol_baseline + dat.wide$FBB_rh_inferiortemporal_UNC_vol_baseline)

dat.wide$isthmuscingulate_Volume_FBB_UNC_baseline <- (dat.wide$FBB_lh_isthmuscingulate_UNC_vol_baseline + dat.wide$FBB_rh_isthmuscingulate_UNC_vol_baseline)

dat.wide$lateraloccipital_Volume_FBB_UNC_baseline <- (dat.wide$FBB_lh_lateraloccipital_UNC_vol_baseline + dat.wide$FBB_rh_lateraloccipital_UNC_vol_baseline)

dat.wide$lateralorbitofrontal_Volume_FBB_UNC_baseline <- (dat.wide$FBB_lh_lateralorbitofrontal_UNC_vol_baseline + dat.wide$FBB_rh_lateralorbitofrontal_UNC_vol_baseline)

dat.wide$lingual_Volume_FBB_UNC_baseline <- (dat.wide$FBB_lh_lingual_UNC_vol_baseline + dat.wide$FBB_rh_lingual_UNC_vol_baseline)

dat.wide$medialorbitofrontal_Volume_FBB_UNC_baseline <- (dat.wide$FBB_lh_medialorbitofrontal_UNC_vol_baseline + dat.wide$FBB_rh_medialorbitofrontal_UNC_vol_baseline)

dat.wide$lingual_Volume_FBB_UNC_baseline <- (dat.wide$FBB_lh_lingual_UNC_vol_baseline + dat.wide$FBB_rh_lingual_UNC_vol_baseline)

dat.wide$medialorbitofrontal_Volume_FBB_UNC_baseline <- (dat.wide$FBB_lh_medialorbitofrontal_UNC_vol_baseline + dat.wide$FBB_rh_medialorbitofrontal_UNC_vol_baseline)

dat.wide$middletemporal_Volume_FBB_UNC_baseline <- (dat.wide$FBB_lh_middletemporal_UNC_vol_baseline + dat.wide$FBB_rh_middletemporal_UNC_vol_baseline)

dat.wide$parahippocampal_Volume_FBB_UNC_baseline <- (dat.wide$FBB_lh_parahippocampal_UNC_vol_baseline + dat.wide$FBB_rh_parahippocampal_UNC_vol_baseline)

dat.wide$paracentral_Volume_FBB_UNC_baseline <- (dat.wide$FBB_lh_paracentral_UNC_vol_baseline + dat.wide$FBB_rh_paracentral_UNC_vol_baseline)

dat.wide$parsopercularis_Volume_FBB_UNC_baseline <- (dat.wide$FBB_lh_parsopercularis_UNC_vol_baseline + dat.wide$FBB_rh_parsopercularis_UNC_vol_baseline)

dat.wide$parsorbitalis_Volume_FBB_UNC_baseline <- (dat.wide$FBB_lh_parsorbitalis_UNC_vol_baseline + dat.wide$FBB_rh_parsorbitalis_UNC_vol_baseline)

dat.wide$parstriangularis_Volume_FBB_UNC_baseline <- (dat.wide$FBB_lh_parstriangularis_UNC_vol_baseline + dat.wide$FBB_rh_parstriangularis_UNC_vol_baseline)

dat.wide$pericalcarine_Volume_FBB_UNC_baseline <- (dat.wide$FBB_lh_pericalcarine_UNC_vol_baseline + dat.wide$FBB_rh_pericalcarine_UNC_vol_baseline)

dat.wide$postcentral_Volume_FBB_UNC_baseline <- (dat.wide$FBB_lh_postcentral_UNC_vol_baseline + dat.wide$FBB_rh_postcentral_UNC_vol_baseline)

dat.wide$posteriorcingulate_Volume_FBB_UNC_baseline <- (dat.wide$FBB_lh_posteriorcingulate_UNC_vol_baseline + dat.wide$FBB_rh_posteriorcingulate_UNC_vol_baseline)

dat.wide$precentral_Volume_FBB_UNC_baseline <- (dat.wide$FBB_lh_precentral_UNC_vol_baseline + dat.wide$FBB_rh_precentral_UNC_vol_baseline)

dat.wide$precuneus_Volume_FBB_UNC_baseline <- (dat.wide$FBB_lh_precuneus_UNC_vol_baseline + dat.wide$FBB_rh_precuneus_UNC_vol_baseline)

dat.wide$rostralanteriorcingulate_Volume_FBB_UNC_baseline <- (dat.wide$FBB_lh_rostralanteriorcingulate_UNC_vol_baseline + dat.wide$FBB_rh_rostralanteriorcingulate_UNC_vol_baseline)

dat.wide$rostralmiddlefrontal_Volume_FBB_UNC_baseline <- (dat.wide$FBB_lh_rostralmiddlefrontal_UNC_vol_baseline + dat.wide$FBB_rh_rostralmiddlefrontal_UNC_vol_baseline)

dat.wide$superiorfrontal_Volume_FBB_UNC_baseline <- (dat.wide$FBB_lh_superiorfrontal_UNC_vol_baseline + dat.wide$FBB_rh_superiorfrontal_UNC_vol_baseline)

dat.wide$superiorparietal_Volume_FBB_UNC_baseline <- (dat.wide$FBB_lh_superiorparietal_UNC_vol_baseline + dat.wide$FBB_rh_superiorparietal_UNC_vol_baseline)

dat.wide$superiortemporal_Volume_FBB_UNC_baseline <- (dat.wide$FBB_lh_superiortemporal_UNC_vol_baseline + dat.wide$FBB_rh_superiortemporal_UNC_vol_baseline)

dat.wide$supramarginal_Volume_FBB_UNC_baseline <- (dat.wide$FBB_lh_supramarginal_UNC_vol_baseline + dat.wide$FBB_rh_supramarginal_UNC_vol_baseline)

dat.wide$frontalpole_Volume_FBB_UNC_baseline <- (dat.wide$FBB_lh_frontalpole_UNC_vol_baseline + dat.wide$FBB_rh_frontalpole_UNC_vol_baseline)

dat.wide$temporalpole_Volume_FBB_UNC_baseline <- (dat.wide$FBB_lh_temporalpole_UNC_vol_baseline + dat.wide$FBB_rh_temporalpole_UNC_vol_baseline)

dat.wide$transversetemporal_Volume_FBB_UNC_baseline <- (dat.wide$FBB_lh_transversetemporal_UNC_vol_baseline + dat.wide$FBB_rh_transversetemporal_UNC_vol_baseline)

dat.wide$insula_Volume_FBB_UNC_baseline <- (dat.wide$FBB_lh_insula_UNC_vol_baseline + dat.wide$FBB_rh_insula_UNC_vol_baseline)

dat.wide$brainstem_Volume_FBB_UNC_baseline <- dat.wide$FBB_brainstem_UNC_vol_baseline

dat.wide$cc_posterior_Volume_FBB_UNC_baseline <- dat.wide$FBB_cc_posterior_UNC_vol_baseline

dat.wide$cc_mid_posterior_Volume_FBB_UNC_baseline <- dat.wide$FBB_cc_mid_posterior_UNC_vol_baseline

dat.wide$cc_central_Volume_FBB_UNC_baseline <- dat.wide$FBB_cc_central_UNC_vol_baseline

dat.wide$cc_mid_anterior_Volume_FBB_UNC_baseline <- dat.wide$FBB_cc_mid_anterior_UNC_vol_baseline

dat.wide$cc_anterior_Volume_FBB_UNC_baseline <- dat.wide$FBB_cc_anterior_UNC_vol_baseline
